# Reliability of urological telesurgery compared with local surgery: multicentre randomised controlled trial

**DOI:** 10.1136/bmj-2024-083588

**Published:** 2026-01-28

**Authors:** Ye Wang, Dan Xia, Wanhai Xu, Mulati Rexiati, Wuyi Zhao, Qingbo Huang, Taoping Shi, Baojun Wang, Shuo Wang, Sheng Tai, Bingzhang Qiao, Yubai Zhang, Sunyi Ye, Xiangping Zhang, Jianle Mao, Yi Zhu, Honglei Wang, Shuangyu Ma, Cheng Yang, Weijun Fu, Tao Song, Qing Ai, Yong Song, Longhe Xu, Guoqiang Yang, Yu Gao, Shaoxi Niu, Jing Guo, Guojun Liu, Xueyuan Xiang, Chaozhao Liang, Xin Ma, Hongzhao Li, Xu Zhang

**Affiliations:** 1 Department of Urology, Chinese PLA General Hospital, Beijing, China; 2 Department of Urology, The First Affiliated Hospital, School of Medicine, Zhejiang University, Hangzhou, China; 3 Department of Urology, Harbin Medical University Cancer Hospital, Harbin, China; 4 Department of Urology, The First Affiliated Hospital of Xinjiang Medical University, Urumqi, China; 5 Shenzhen Edge Medical Co, Shenzhen, China; 6 Department of Urology, The First Affiliated Hospital of Anhui Medical University, Hefei, China; 7 Department of Anesthesiology, The Third Center of Chinese PLA General Hospital, Beijing, China

## Abstract

**Objective:**

To investigate whether the reliability of telesurgery is non-inferior to that of standard local surgery in patients undergoing urological robotic operations.

**Design:**

Multicentre, non-inferiority, randomised controlled trial.

**Setting:**

Five hospitals in China from December 2023 to June 2024.

**Participants:**

Patients scheduled to undergo radical prostatectomy or partial nephrectomy.

**Interventions:**

Patients were randomly assigned 1:1 to undergo telesurgery or local surgery.

**Main outcome measures:**

The primary outcome was the probability of success of surgery, determined by the medical team on the basis of pre-established criteria. The pre-specified non-inferiority margin was an absolute reduction in probability of 0.1. Thirteen clinical secondary outcomes were associated with the operation and early recovery, and one secondary outcome related to the workload of the medical team. Four technical secondary outcomes for the surgical system were also explored, including network latency, display latency, frame loss during telesurgery, and system malfunction. The participants were followed up at four and six weeks postoperatively for the secondary outcomes of recovery and complications.

**Results:**

A total of 72 participants were enrolled in the study and randomised 1:1 to the telesurgery group and the local surgery group for the intention-to-treat set. The median age of patients was 61.0 (interquartile range 57.5-68.0) years in the telesurgery group and 65.0 (56.5-70.0) years in the local surgery group. Telesurgery was not inferior to local surgery in terms of the probability of surgical success in the intention-to-treat population, accounting for clustering by surgeon (success probability difference 0.02 (95% credible interval −0.03 to 0.15) with bayesian posterior probability of 0.99 for non-inferiority). The telesurgery system was stable with a distance from 1000 km to 2800 km, a mean round trip network latency of 20.1-47.5 ms, and frame loss of 0-1.5 per telesurgery. Secondary outcomes, including operative basic data, complications, early recovery, oncological outcome, and medical team workload, did not differ substantially between the two groups.

**Conclusions:**

The reliability of telesurgery was non-inferior to that of local robotic surgery according to the non-inferiority margin of a 0.1 reduction in success probability.

**Trial registration:**

ChiCTR.org ChiCTR2300077721.

## Introduction

Surgery, defined as the invasive removal of lesions or reconstruction of tissues and organs,^1^ has evolved from open techniques to minimally invasive robotic assisted approaches,^2^ ^3^ ^4^ ^5^ ^6^ progressively overcoming human limitations in precision, ergonomics, and visualisation. However, one critical barrier persists: the geographical dependency of surgery. Surgeons must physically operate in the same room as the patient, leaving underserved regions, disaster zones, military environments, and space missions vulnerable to gaps in timely care.^7^ ^8^ ^9^ Telesurgery—the remote performance of robotic assisted procedures via telecommunication networks—emerges as a transformative solution. By decoupling the surgeon’s presence from the operating site, it redefines surgical accessibility.^10^ ^11^

Conventional robotic systems, although enhancing local precision, fail to overcome spatial constraints. Rural hospitals lack specialist surgeons, and disaster or warfare responders encounter logistical delays. Telesurgery overcomes these gaps by integrating three pillars: a surgeon console with haptic controls and 3D visualisation, a patient-side robotic system, and ultra low latency communication networks (for example, optical fibre dedicated lines, 5G/6G wireless network, or satellite). The surgeon’s movements are digitised, transmitted via secure networks, and executed by robotic arms with sub-millimetre accuracy. During the signal transmission of the telesurgery system, challenges such as latency, data packet loss, or misinterpretation may arise. Although these problems typically have minimal individual effects on procedural precision, whether their cumulative effects—stemming from minor discrepancies over time—could compromise the probability of surgical success and patients’ outcomes remains unclear.

Despite these prospects, the clinical validity of telesurgery remains unproved. Early milestone, such as the 2001 trans-Atlantic cholecystectomy,^12^ prioritised technical novelty over clinical rigour. Over the next 20 years, telesurgery research progressed slowly, which may have been due to the limitations of robotic systems and the instability of telecommunications. Fortunately, telesurgery, assisted by advanced telecommunication and surgical robot technology, has entered a fast developmental stage in recent years.^13^ ^14^ Telesurgery has been explored in urology,^15^ ^16^ orthopaedics,^17^ cardiovascular medicine,^18^ military medicine,^7^ and other areas, providing a meaningful exploration for the development of telesurgery. Our team conducted an exploratory trial to explore the feasibility of the different kinds of urological operations, including radical prostatectomy, partial nephrectomy, resection of adrenal tumour, and dismembered ureteroplasty of the retrocaval ureter, which covered the main types of operation on the urological system. However, previous studies, including our exploratory trial,^19^ focused on feasibility in narrow indications, and no clinical evidence has been established to support further research or the wider application of telesurgery. Therefore, we designed this randomised controlled trial in patients undergoing robotic urological procedures (radical prostatectomy or partial nephrectomy) to determine whether telesurgery is non-inferior to standard local surgery in the probability of achieving surgical success while also evaluating technical performance (for example, network latency, system stability) and secondary clinical outcomes (for example, complications, recovery, workload).

## Methods

### Determination of non-inferiority margin

As this study is the first randomised controlled trial of telesurgery, no established reports were available for reference for determining the non-inferiority margin. Firstly, we must clarify that failure of telesurgery mainly occurs when switching from telesurgery to local robotic surgery, rather than directly to laparoscopic or open surgery. The conversion risk from telesurgery to local robotic surgery is a primary consideration when determining the non-inferiority margin. At the same time, we included the probability of surgical success of the da Vinci robotic system in clinical trials at different periods as important references for determining the non-inferiority margin. The clinical reports show that the probability of success of the da Vinci robotic system ranged from 79.2% to 96.5% during different development periods.^20^ ^21^ ^22^ ^23^

After careful discussion between the clinical trial team and the engineer, we set the pre-specified non-inferiority margin as an absolute reduction in the probability of success of 0.1 at the early development period of telesurgery, considering the operation conversion and the malfunction of the telesurgery system.

### Telesurgery system structure and telesurgery pathways

**Fig 1:**
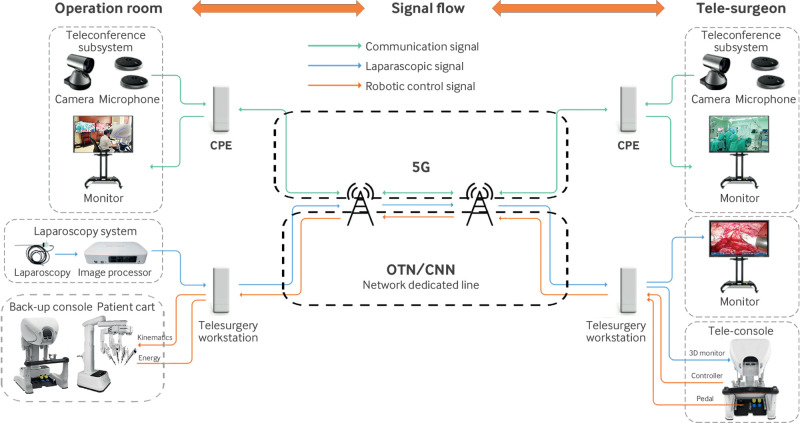
Structure and instrument arrangement of telesurgery system. Telesurgery system includes three subsystems: robot subsystem, telecommunication subsystem, and teleconference subsystem. Dedicated line and back-up were used for transporting data packages between surgeon console and patient cart through telecommunication subsystem. Back-up console was installed in operation room. 5G wireless network was used for teleconference subsystem. CNN=cloud connect network; CPE=customer premises equipment; OTN=optical transport network

The telesurgery system consisted of three main components: a robotic subsystem, a telecommunication subsystem, and a teleconference subsystem (fig 1; supplementary table A). We used a four arm, multi-port Surgical Robotic System (MP1000, Edge Medical Co, Shenzhen, China)^24^ as the robotic subsystem. The telerobotic console was installed at the first surgeon’s hospital. A robotic cart unit and a back-up local console were installed in the patient’s operation room. When the telesurgery system malfunctioned, the local surgeon would take control of the robotic cart unit via the back-up local console and complete the remaining steps of the operation. Switching from telesurgery mode to local surgery mode can be rapid.

**Fig 2:**
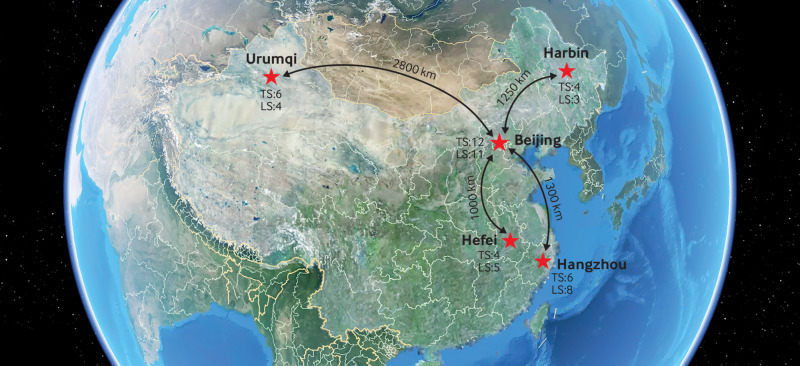
Telesurgery network. Telesurgery network between Beijing and other four sites was set up via optical transport network or cloud connect network dedicated lines. Bidirected telesurgery was performed through this network with centre of Beijing, and numbers of participants who received telesurgery (TS) or local surgery (LS) are indicated

The telecommunication subsystem was the core system for telesurgery, which was used to transport telecontrol signals from the tele-surgeon console to the robotic arms and laparoscopic image data packages from the endoscope to the surgeon’s console. The telecommunications pathways used in this trial were an optical transport network and a cloud connect network dedicated network, each with a bandwidth of 60 Mbps, provided by China Telecom Company and China Unicom Company, respectively. Telesurgery pathways were constructed between Beijing and the other four trial sites (fig 2; supplementary table A). The distance of the pathways from Urumqi, Harbin, Hangzhou, and Hefei to Beijing was about 2800 km, 1300 km, 1250 km, and 1000 km, respectively. We recorded important factors, including round trip network latency, frame loss, and display latency to monitor the quality of telesurgery and provide early warnings of potential risks, such as remote control failure or endoscope image distortion.

The teleconference subsystem was used to establish communication between the tele-surgeon and the medical assistant team. Supplementary table A gives the details of the proposed system.

### Study design

This investigator initiated, multicentre, single blind, non-inferiority, randomised controlled trial was conducted at five sites in China. The primary objective was to test the probability of success of the telesurgery and the hypothesis that telesurgery was not inferior to standard local robotic surgery. The secondary objective was to explore differences between groups in the basic operative process, patients’ recovery, and surgical system stability. The procedures used in this trial were partial nephrectomy and radical prostatectomy, which represent upper urinary tract and lower urinary tract operations, respectively. Patients were randomised centrally to receive either telesurgery or local surgery. The protocol and statistical analysis plan for the trial are available in the supplementary materials.

### Inclusion and exclusion criteria

The inclusion criteria were age 18-80 years; body mass index 18-30; diagnosis of renal tumour or prostate cancer and fit to undergo urological laparoscopic surgery, including prostatectomy and partial nephrectomy; physiological condition suitable for robot assisted laparoscopic surgery; and willingness to cooperate and complete study follow-up and related examinations. The exclusion criteria were severe cardiovascular or circulatory diseases that are not tolerable for surgery; pregnancy or lactation; history of epilepsy or mental illness; severe allergies or suspected/confirmed alcohol or drug addiction; and inability to understand the study requirements or complete the study’s follow-up schedule.

### Randomisation and masking

The participants were randomly assigned in a 1:1 ratio to undergo either telesurgery or standard local robotic surgery, using stratified randomisation with random block sizes of four. Stratification was based on the type of surgery (radical prostatectomy or partial nephrectomy), and randomisation was done separately for each stratum. Specifically, the implementation of telesurgery was linked to two hospitals, and randomisation was done at the patient-side hospital by an independent statistician using web based randomisation software (Sigma Med, China). After randomisation, patients were scheduled for surgery according to their group assignments, and the surgeon was notified to prepare for the surgery. For patients undergoing telesurgery, remote consultation was also conducted to clarify the specific details of the surgery. Participants, follow-up specialists (independent research nurses or doctors), and independent statisticians were masked to the form of surgery performed (telesurgery or local surgery). As masking of the medical team was not feasible, the determination of surgical success was conducted by the surgical team according to pre-specified criteria to minimise subjectivity. Meanwhile, the traceability of surgical video records will ensure the reliability and reproducibility of the findings. The randomisation group was not involved in assessing the primary outcomes and were masked to them. Furthermore, masked central pathologists reviewed the biopsies.

### Procedures

The management was the same for all participants, except for the form of operation, which was either telesurgery or local surgery. In this trial, four teams, including the surgeon team, telesurgery procedure planning team, telesurgery system monitoring team, and follow-up team, were set up to coordinate telesurgery arrangements, remote consultations, and other matters. For telesurgery, a remote consultation was required before the surgery. Once the telesurgery time was confirmed, the engineers were notified to prepare the telesurgery system, including necessary steps such as debugging and inspecting the remote lines.

Surgeons were required to have completed >500 robot assisted laparoscopic surgical procedures. To reduce surgical heterogeneity, all surgeons received in-person instructions from XZ. The surgical approach was selected according to the hospital’s practices and the participant’s location to enhance the generalisability of the research findings. The responsibilities and composition of the four teams and the surgical procedure details are detailed in supplementary tables D and E. Participants were assessed at baseline and at four weeks and six weeks after surgery.

### Outcomes

The primary outcome was the probability of success of surgery, which the research team defined on the basis of the characteristics of telesurgery. The success was confirmed according to the following determination points: the surgical process was carried out according to the planned steps; no obvious injury to large blood vessels or adjacent organs occurred during the surgery; no conversion of the surgical method occurred, such as switching from telesurgery to local robotic surgery or converting local robotic surgery to laparoscopic surgery or open surgery; the surgery proceeded as planned, and no postponement due to surgical system malfunction occurred. The success of each case was jointly confirmed by the medical team on the basis of these pre-specified determination points after the surgery.

The trial had 18 secondary outcomes, including overall and functional recovery, incidence of complications, oncological outcomes, medical team workload, and surgical system status. The status of the telesurgery system was monitored using software that measured network latency, display latency, and frame loss. Malfunction of the surgical system was recorded during surgery and preoperative testing.

Intraoperative blood loss, operative time, postoperative and intensive care hospital admission days, reoperation, readmission to hospital, and blood transfusion either during or after the operation, as well as mortality, were all recorded. We extracted all clinical data and intraoperative events from hospital records.

Complications were recorded during the inpatient stay and at follow-up time points according to the Clavien-Dindo Classification.^25^ Oncological outcomes included positive surgical margin status, which refers to the presence of tumour cells at the edge of the resected tissue after histopathological examination, indicating incomplete tumour removal and associated with an increased risk of local recurrence or metastasis. The quality of early recovery was assessed using a 15 item Quality of Recovery Questionnaire (QoR-15)^26^ and a 30 second chair-to-stand test. Prostate associated outcomes, including urinary control and sexual function, were assessed using the Expanded Prostate Cancer Index Composite-26 (EPIC-26).^27^ The workload of the medical team was measured by using the NASA Task Load Index.^28^

A blinded independent research nurse or doctor at each trial site ascertained postoperative secondary outcomes through medical records, in-person patient interviews, or WeChat social media.^29^. Supplementary table I provides details of the primary and secondary outcomes.

### Sample size calculation

We used a simulation method for power and sample size calculation based on related non-inferiority trials. On the basis of pre-experimental results, both telesurgery and local surgery had a probability of success of 98% or more. The unilateral α value was set at 0.025, and the power (1−β) was set at 0.8. The sample size ratio of the telesurgery and local surgery groups was 1:1. The non-inferiority margin was 0.1 (that is, a reduction in probability of success of 0.1), which the trial leaders chose as the maximum reasonable and clinically relevant limit for the outside limit of the confidence interval. Assuming a 10% attrition rate, we determined that a sample size of 34 participants per group was needed. If the dropout rate exceeded the expected 10%, resulting in insufficient valid cases, the number of participants could be appropriately increased according to the protocol.

### Statistical analysis

The trial design was a two group randomised controlled trial with three assessment points (baseline and four and six weeks after surgery). We analysed the participants according to their randomisation group. The first outcome, probability of success, was independent of the follow-up time points and was decided by the research team after the surgery. The subsequent follow-up assessments at four and six weeks were conducted exclusively for the secondary outcomes.

For descriptive statistics, we described quantitative variables as mean and standard deviation or median and interquartile range. We described categorical variables as percentages or the number of cases.^30^

For the primary outcome, we derived the differences in the probability of success between the groups along with their 95% credible interval from the posterior distributions of the bayesian mixed effect logistic regression with penalised priors accounting for clustering by surgeon, taking the intercept of surgeon as a random effect. We calculated the 95% credible intervals by using the 2.5% and 97.5% posterior quantiles and reported them accordingly. For the penalised priors, we assigned fixed effects coefficients normal priors with mean 0 and standard deviation 5, reflecting prior belief that effect sizes are centred around zero but allowing considerable uncertainty, and we used half-Cauchy priors with a scale parameter of 2 for the variance of the random effect, a commonly recommended choice that penalises excessively large variance components while remaining flexible. We did both intention-to-treat analysis and per protocol analysis. We imputed missing data for the primary outcome in the intention-to-treat analysis by using multiple imputation by logistic model of fully conditional specification, generating 50 complete datasets. The imputation models included the randomisation group variable and covariates (for example, surgeon, hospital, surgery type) to predict missing values.^31^ For each multiply imputed dataset, we fitted distinct bayesian adjustment models, generating Markov chain Monte Carlo posterior sample chains by using Stan’s Hamiltonian Monte Carlo algorithm. These posterior samples were then aggregated across all imputed datasets for each model specification to form a unified pooled posterior distribution (bayesian pooling). Finally, we derived key inferential quantities from these pooled posteriors.^32^ We analysed the posterior probabilities for non-inferiority, taking the non-inferiority value of −0.1 as the reference point. The complete methods of imputation are provided in the statistical codes in the supplementary materials.

For the secondary outcomes, we assessed differences between groups by using the mixed effect linear regression model for all continuous outcomes and bayesian mixed effect logistic regression with penalised priors for the positive surgical margin status of the tumour (binary variable). The penalised priors for bayesian mixed effect logistic regression were the same as the priors for the primary outcome. We used Fisher’s exact test to analysis the risk difference of the Clavin-Dindo complications without stratification. We assessed the 95% confidence intervals of the odds ratio or risk differences and the effect sizes of these analyses.

All tests were two tailed with significance level α=0.05. We used SAS version 9.4 or RStudio for analyses. We visualised telesurgery monitoring data by using RStudio or GraphPad Prism 8.0.

### Patient and public involvement

As patient and public involvement was not a routine practice in the areas where this trial was conducted, no patients were involved in the design or implementation of this trial, and nor did they participate in the subsequent data analysis, interpretation, or writing of the manuscript. However, all patients were aware of the trial objectives and protocols during recruitment.

## Results

### Participants

**Fig 3:**
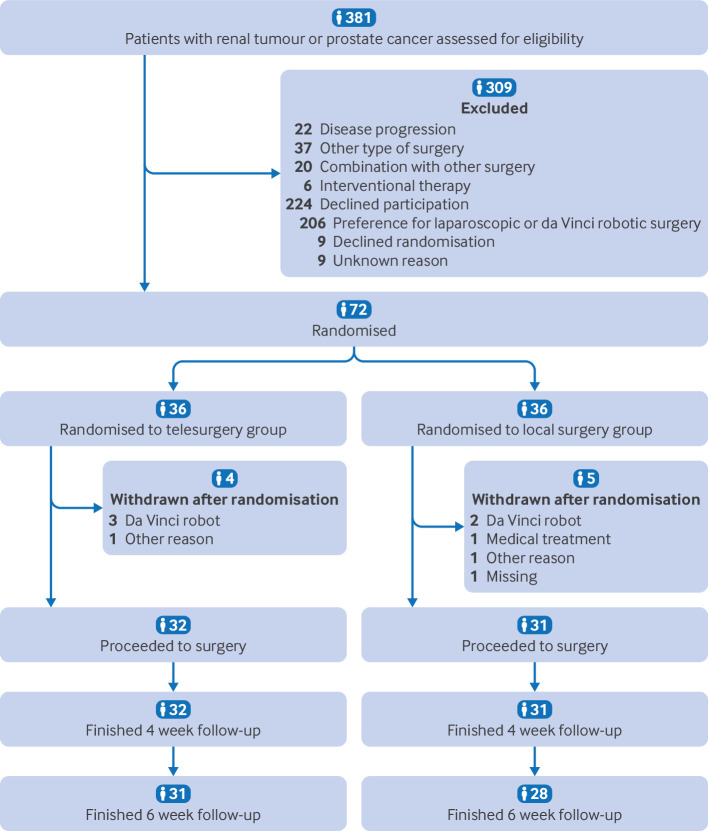
Trial profile. Patients with renal tumour or prostate cancer were randomly assigned in 1:1 ratio to undergo telesurgery or local surgery. Follow-up period applied only to patients undergoing telesurgery or local robotic surgery

The trial was conducted from December 2023 to June 2024 at five sites in China. The first surgery was performed on 20 December 2023 and the last one on 25 April 2024; the last visit by a participant was on 7 June 2024. Among the 381 patients screened, 309 were excluded and 72 participants were randomised (fig 3; supplementary figures A and B), of whom nine (12.5%) withdrew (four for telesurgery and five for local surgery). Finally, 32 participants (17 prostatectomies and 15 partial nephrectomies) underwent telesurgery, and 31 participants (16 prostatectomies and 15 partial nephrectomies) underwent local surgery. The baseline characteristics of participants in the two groups were balanced for demographic and disease related factors (table 1; supplementary tables B and C).

**Table 1:** Characteristics of participants at baseline. Values are numbers (percentages) unless stated otherwise

Characteristic	Intention-to-treat population			Per protocol population	
	Telesurgery group (n=36)	Local surgery group (n=36)		Telesurgery group (n=32)	Local surgery group (n=31)
Male sex, total	28 (78)	26 (72)		24 (75)	22 (71)
Male sex, with renal tumour	7/16 (44)	7/16 (44)		6/15 (40)	7/15 (47)
Median (IQR) age, years	61.0 (57.5-68.0)	65.0 (56.5-70.0)		61.0 (57.5-68.0)	62.0 (53.0-70.5)
Median (IQR) height, m	1.69 (1.60-1.72)	1.68 (1.63-1.75)		1.68 (1.60-1.72)	1.68 (1.62-1.75)
Median (IQR) weight, kg	70.0 (64.5-75)	70.0 (65-80.3)		70 (64.5-76.3)	70.0 (65-82)
Median (IQR) body mass index*	25.0 (23.0-27.3)	25.4 (23.4-27.6)		25.3 (23.0-27.6)	25.8 (24.2-27.7)
Tumour characteristics:†					
Prostate cancer	20 (56)	20 (56)		17 (53)	16 (52)
Renal tumour	16 (44)	16 (44)		15 (47)	15 (48)
Patient location:‡					
Beijing	12 (33)	12 (33)		12 (38)	11 (35)
Urumqi	6 (17)	6 (17)		6 (19)	4 (13)
Harbin	4 (11)	4 (11)		4 (13)	3 (10)
Hangzhou	8 (22)	8 (22)		6 (19)	8 (26)
Hefei	6 (17)	6 (17)		4 (13)	5 (16)

IQR=interquartile range.

* Weight in kilograms divided by square of height in metres.

† Renal tumour included renal cancer and renal hamartoma. See supplementary tables C and D for analysis of details of tumour characteristics. For prostate cancer, serum prostate specific antigen concentration, Gleason score, and prostate volume were reported. For renal tumours, RENAL score was reported.

‡ Telesurgery of patients located in Beijing was done by surgeons in other four trial sites. Telesurgery of patients located in Urumqi, Harbin, Hangzhou, and Hefei was done by surgeons in Beijing.

### Primary outcome

The primary outcome, probability of surgical success, was available for both trial groups. The probability of surgical success in the telesurgery and local surgery groups in the intention-to-treat population were 100% and 94.44%, respectively (table 2). In the bayesian mixed effect logistic regression analysis accounting for clustering by surgeon, the estimated difference in the probability of success in the intention-to-treat and per protocol populations was 0.02 (95% credible interval (CrI) −0.03 to 0.15; bayesian posterior probability 0.99 for non-inferiority) and 0.003 (−0.001 to 0.03; bayesian posterior probability >0.99 for non-inferiority), respectively. The lower boundaries of the 95% credible intervals were all above the pre-specified non-inferiority margin of −0.1, and posterior probabilities for non-inferiority were both higher than 0.98, indicating that telesurgery was not inferior to standard local robotic surgery with high probability. The sensitivity analysis on adjustment of other stratification also supported the non-inferiority hypothesis (supplementary table F). Only one failure was observed in the local surgery group owing to a surgical robotic malfunction. We did not analyse superiority.

**Table 2:** Trial outcomes

Outcome	Telesurgery	Local surgery	Adjusted difference or odds ratio	Adjusted effect size	P (difference in probability of success >−0.1)
**Primary outcome**					
Probability of surgical success:*			(Adjusted probability difference (95% CrI))		
No (%) in intention-to-treat population†	36/36 (100)	34/36 (94.44)	0.02 (−0.03 to 0.15)	0.02	0.99
No (%) in per protocol population	32/32 (100)	30/31 (96.77)	0.003 (−0.001 to 0.03)	0.003	>0.99
**Secondary outcomes** **‡**					
Surgery details:			(Adjusted mean difference (95% CI))		
Median (IQR) operative time, min§	151.5 (98.5-180.0)	135.0 (94.5-207.5)	13.67 (−25.22 to 29.61)	0.05	0.87
Median (IQR) warm ischemia time, min¶	16.0 (15.0-22.0)	20.0 (16.5-22.5)	2.76 (−8.01 to 3.34)	−0.32	0.41
Median (IQR) blood loss, mL	50.0 (50.0-100.0)	50.0 (50.0-100.0)	20.00 (−35.56 to 44.54)	0.06	0.82
Perioperative morbidity:			(Adjusted mean difference (95% CI) )		
Median (IQR) postoperative hospital admission , d	6.0 (5.0-6.0)	5.5 (4.3-7.0)	−0.25 (−1.43 to 0.92)	−0.11	0.67
Mean (SD) length of stay in critical care, d**	0.03 (0.18)	0.03 (0.18)	0.033 (−0.04 to 0.09)	0.21	0.48
Early recovery:					
Median (IQR) QoR-15 score:††			(Adjusted mean difference (95% CI))		
Baseline	147.5 (141.8-150.0)	148.0 (145.0-150.0)	1.60 (−5.87 to 0.53)	−0.44	0.10
4 weeks	146.0 (136.8-149.0)	145.0 (138.0-149.0)	2.07 (−5.08 to 3.22)	−0.11	0.65
6 weeks	147.0 (140.0-150.0)	149.0 (144.0-150.0)	8.18 (0.94 to 31.82)	0.53	0.06
Median (IQR) 30 second chair to stand test:‡‡			(Adjusted mean difference (95% CI))		
Baseline	15.0 (13.0-16.3)	14.0 (12.0-16.0)	1.11 (−4.07 to 0.38)	−0.42	0.10
4 weeks	15.0 (12.0-16.0)	14.0 (12.0-16.0)	1.47 (−4.73 to 1.19)	−0.31	0.24
6 weeks	14.0 (12.0-20.0)	15.0 (12.3-16.0)	1.74 (−3.99 to 2.97)	−0.08	0.77
Median (IQR) EPIC-26 score:§§			(Adjusted mean difference (95% CI))		
Baseline	52.0 (40.0-58.0)	51.0 (44.0-58.5)	6.44 (−7.78 to 18.49)	0.29	0.41
4 weeks	64.0 (56.0-70.0)	59.5 (55.0-68.0)	4.23 (−4.67 to 12.77)	0.35	0.35
6 weeks	56.0 (47.0-67.0)	59.0 (51.5-64.0)	5.72 (−2.64 to 20.74)	0.61	0.12
Task load of medical team:					
Median (IQR) NASA-TLX score:¶¶			(Adjusted mean difference (95% CI))		
Surgeon	29.0 (24.0-44.0)	48.0 (41.0-60.5)	−6.26 (−31.52 to −6.41)	−0.93	0.004
First assistant	48.0 (34.8-59.3)	40.5 (35.0-60.5)	6.28 (−13.97 to 11.23)	−0.06	0.83
Instrument nurse	48.5 (38.5-60.0)	53.5 (41.5-60.3)	−5.99 (−14.32 to 9.73)	−0.11	0.70
Clinical outcomes:			(Odds ratio (95% CI))		
No (%) positive surgical margin***	1/32 (3)	5/32 (16)	13.41 (0.80 to 575.37)	13.41	0.07
No (%) complications and adverse events†††	1/31 (3)	0	0.031 (−0.03 to 0.09)	0.03	0.61

CI=confidence interval; CrI=credible interval; EPIC-26=Expanded Prostate Cancer Index Composite-26; IQR=interquartile range; NASA-TLX=NASA Task Load Index; QoR=Quality of Recovery Questionnaire; SD=standard deviation.

* Success was defined as: surgical process is carried out according to planned steps; no obvious injury to large blood vessels or adjacent organs during surgical process; no conversion of surgical method, such as switching from telesurgery to local robotic surgery or converting local robotic surgery to laparoscopic surgery or open surgery; surgery proceeded as planned, and no postponement due to surgical system malfunction. Postponement of surgeries due to patient related factors was not considered surgical failure. Telesurgery was considered successful if it could be completed using only telesurgery system without conversion to local surgery for any reason. Sole unsuccessful case was due to malfunction of robotic system before patient was ready for anaesthesia in operating room. In this instance, external system connection cable was crushed by equipment, and manufacturer provided replacement cable 2 days later. Patient underwent surgery according to original randomised group 3 days later, and surgery was successfully performed. Total probability of success of surgery for per protocol population was 98.4%. Posterior probability of bayesian mixed effects logistic regression was for non-inferiority.

† Multiple imputation was performed for intention-to-treat (ITT) population by fully conditional specification method with 50 cycles. Main analysis of primary outcome used bayesian mixed effects logistic regression with penalised priors, accounting for clustering by surgeon as random effect within ITT framework. Per protocol analysis was also conducted.

‡ In results of secondary outcomes, mixed effects linear regression and bayesian mixed effects logistic regression with penalised priors were applied with surgeon as random effect, respectively, for continuous secondary outcomes and categorical secondary outcomes. Adjusted mean difference and odds ratio, along with their 95% CIs, were reported. For postoperative complications, owing to low frequency of positive events, odds ratio and its 95% CI were calculated using Fisher’s exact test. Effect size for continuous outcome was adjusted Cohen’s d value.

§ Operative time is total surgery time, including robotic operation time and other necessary steps, extracted from patients’ surgical records.

¶ Warm ischemia time is main factor in evaluating quality of partial nephrectomy. Two cases in telesurgery and 1 case in local surgery group were without renal artery blocking.

** One case in each group transferred to intensive care unit after surgery owing to complex underlying diseases, including respiratory system and cardiovascular disease. Both cases transferred to general ward 1 day later, and no adverse events occurred.

†† QoR-15 scale is an important measure of early postoperative health status of patients. Scores range from 0 to 150. Higher scores reflect better health status. Scale at 6 weeks was not available for four withdrawn patients.

‡‡ 30 second chair-to-stand test is an easy and effective physical functional test for populations. Test at 4 weeks was unavailable for six patients: 1 was undergoing hip joint treatment, and 5 had not been tested. Test at 6 weeks was unavailable for 6 patients: 4 had completely withdrawn, 1 was receiving hip joint treatment, and 1 had not been tested.

§§ EPIC-26 is a standardised instrument for measuring health status of patients who have received prostatectomy. It was determined only for patients with prostate cancer. Six week scale was not available for one withdrawn patient.

¶¶ NASA-TLX is a multidimensional scale designed to obtain workload estimates relating to a task, including medical work. This task load was measured immediately after the surgery.

*** Oncological outcome (positive margin rate) is to measure the precision of surgery, which is important for long term outcomes. Bayesian mixed effects logistic regression with penalised priors (surgeon as random effect) was used and P value was “two tailed weighted posterior probability” calculated from posterior sample probabilities. CI for positive margin rate was CrI.

††† Adverse events recorded using Clavien-Dindo Classification and all complications over II level are reported in this table during whole follow-up period. Only 1 patient had myocardial infarction and received coronary intervention treatment.

### Secondary outcomes

#### Telesurgery monitoring data

**Fig 4:**
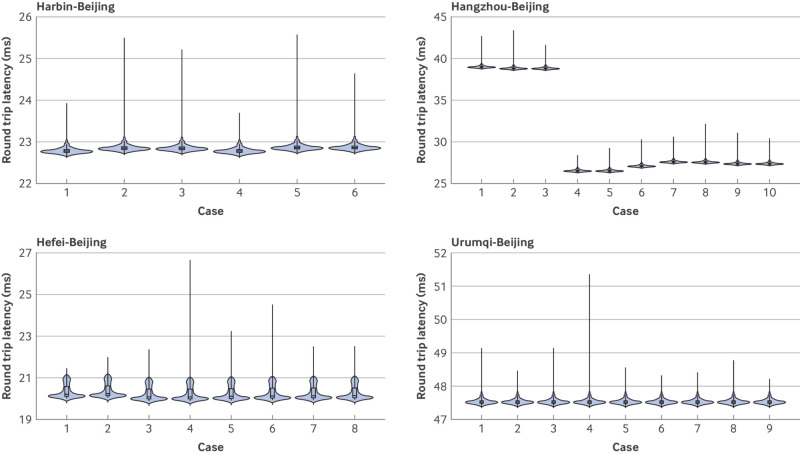
Telesurgery monitoring data. Violin plot combined with box plot for real time round trip network latency distribution of cases on four telesurgery pathways (Harbin-Beijing pathway, Hangzhou-Beijing pathway, Hefei-Beijing pathway, and Urumqi-Beijing pathway)

**Fig 5:**
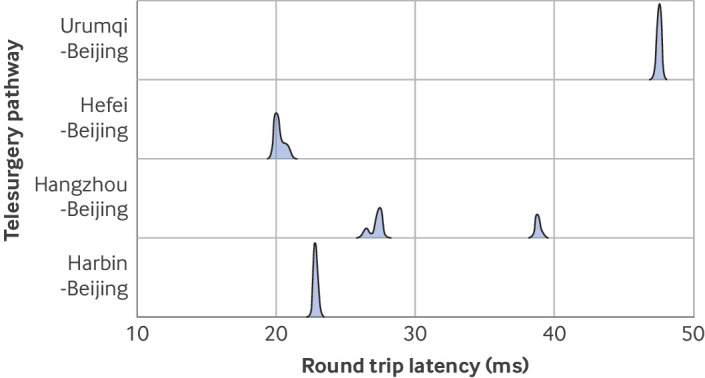
Telesurgery monitoring data. Ridgeline plot for overall view of round trip network latency distribution on four telesurgery pathways

**Fig 6:**
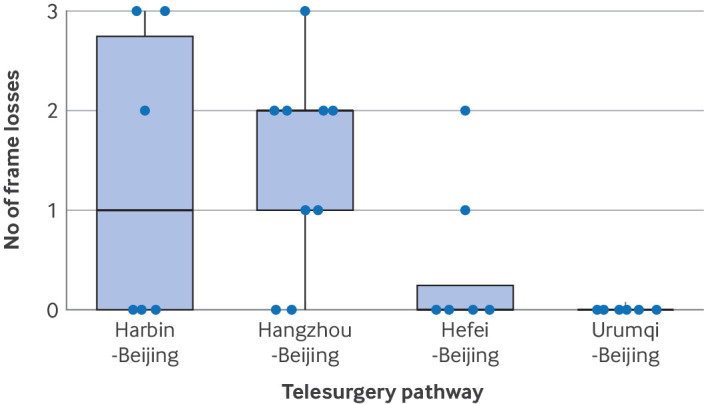
Telesurgery monitoring data. Box plot combined with scatter plot for total frame loss of cases and overall view

Telesurgery monitoring data and malfunctions in the surgical system were recorded (fig 4, fig 5, fig 6; supplementary tables G and H; supplementary figures D-O). In the telesurgery group, the round trip network latency was stable and linearly proportional to the physical distance of the telesurgery pathway (supplementary figure C). The mean round trip network latencies of Beijing-Urumqi (2800 km), Beijing-Hangzhou (1300 km), Beijing-Harbin (1250 km), and Beijing-Hefei (1000 km) telesurgery pathways were 47.5 (SD 0.08) ms, 30.6 (5.7) ms, 22.8 (0.05) ms, and 20.1 (0.06) ms, respectively. The frame loss was very low, and the mean total frame loss number of telesurgery pathways was from 0 to 1.5 per telesurgery. One preoperative malfunction event was observed in the local surgery group, resulting in the postponement of the surgery for three days, which was deemed a failure.

#### Surgery details

The median operative time of the telesurgery and local surgery groups was 151.5 (interquartile range 98.5-180.0) min and 135.0 (94.5-207.5) min, respectively, with an adjusted mean difference of 13.67 (95% CI −25.22 to 29.61; P=0.87; adjusted Cohen’s d=0.05). The warm ischaemia time for partial nephrectomy and blood loss showed no statistically significant differences between groups, considering both the P value and the effect size (table 2).

#### Perioperative morbidity

Postoperative hospital admission days in the telesurgery and local surgery groups were 6.0 (interquartile range 5.0-6.0) and 5.5 (4.3-7.0), respectively (adjusted mean difference −0.25, 95% CI −1.43 to 0.92; P=0.67; adjusted Cohen’s d=−0.11) (table 2). Only one Clavien-Dindo level III complication not directly related to the surgery was observed in the telesurgery group. The length of stay in critical care was the same for both groups 0.03 (standard deviation 0.18) days (adjusted mean difference 0.03, 95% CI −0.04 to 0.09; P=0.48; adjusted Cohen’s d=0.21). Other associated outcomes, including reoperation intervention, readmission to hospital, blood transfusion, and mortality, were not observed in this trial.

#### Early recovery

The QoR-15 scale score at baseline, four weeks, and six weeks for the telesurgery and local surgery groups were 147.5 and 148.0 (adjusted mean difference 1.60, 95% CI −5.87 to 0.53; P=0.10; adjusted Cohen’s d=−0.44), 146.0 and 145.0 (adjusted mean difference 2.07, −5.08 to 3.22; P=0.65; adjusted Cohen’s d=−0.11), and 147.00 and 149.00 (adjusted mean difference 8.18, 0.94 to 31.82; P=0.06; adjusted Cohen’s d=0.53) , respectively. The 30 second chair-to-stand test and EPIC-26 score (for patients with prostate cancer) showed no statistically significant differences between groups (table 2).

#### Oncological outcome

The positive margin rates of telesurgery and local surgery were 3% and 16%, respectively (odds ratio 13.41, 95% CrI 0.80 to 575.37; two tailed weighted posterior probability 0.07) (table 2). The positive margin was observed only in cases of prostatectomy.

#### Task load of medical team

The task load of the first assistant and instrument nurse did not differ significantly between groups (table 2). The task load scores of surgeons in the telesurgery and local surgery groups were 29.0 and 48.0, respectively (adjusted mean difference −6.26, 95% CI −31.52 to −6.41; P=0.004; adjusted Cohen’s d=−0.93).

## Discussion

In this trial, telesurgeries were successfully performed with stable latency and low frame loss, even at the longest distance of 2800 km. The lower boundary of the 95% credible intervals for the difference in the probability of success in both the intention-to-treat and per protocol analyses was higher than the pre-specified −0.1 non-inferiority margin, and posterior probabilities for non-inferiority were higher than 0.98, which both provided statistical evidence for the non-inferiority hypothesis. Except for the surgeon’s workload, early recovery, complications, and ontological outcomes showed no statistically significant differences between groups. The NASA Task Load Index is a subjective rating scale in which the scores are associated with the participants’ perceptions. As masking the medical team, including surgeons, was not feasible in this trial, bias may have been introduced into the results. The decrease in the workload of surgeons in the telesurgery group may be due to the inability to be masked. Furthermore, the positive margin rate of the telesurgery group (3.1%) was lower than that of the local surgery group (16.1%), and further research is needed to substantiate this.

### Implications of findings

In developing countries, the distribution of medical resources is uneven, and the graded medical treatment system is in its early stages. In China, high quality medical resources are concentrated in larger cities,^33^ which causes difficulties such as long waiting times for beds, long distance travel, and increased total expenses for out-of-town medical treatment. At the same time, the international community faces the dual pressure of an ageing population and a significant trend of early onset cancer,^34^ which can be expected to continue to increase the number of patients with cancer and the demand for surgical procedures.

Telesurgery is a feasible solution to these problems.^35^ Before this trial, we had conducted a small sample, single arm trial on urological telesurgery and observed the feasibility of different urological organ operations.^19^ The benefits of telesurgery are multifaceted, and this needs to be demonstrated through larger cohort studies. Only when robust evidence shows comparable probabilities of success between telesurgery and local robotic surgery can larger scale studies be supported, taking into account ethical considerations and benefits to patients. Therefore, we conducted this non-inferiority randomised controlled trial first.

### Strengths and limitations of study

Despite the ideal performance of telesurgery in this trial, several concerns and limitations remain. Firstly, clinical adoption of telesurgery remains limited, and a notable proportion of trial participants were recruited from non-local regions. A comprehensive evaluation of the benefits of telesurgery must extend beyond technical feasibility to include multidimensional considerations such as long term clinical outcomes, health economic impacts, sociological implications, medical training requirements, and patient centred humanistic factors, none of which could be accomplished in this trial.

Secondly, no articles were available for reference in determining the non-inferiority margin. The non-inferiority margin of this trial was determined by clinical experts through discussion based on the characteristics of telesurgery and relevant clinical trials on the robotic surgery. Thirdly, although the sample size was estimated on the basis of the predefined non-inferiority margin, we acknowledge that the relatively small cohort in this trial may have limited the ability to detect statistically significant differences in certain outcomes. Fourthly, the withdrawal rate was slightly high, at 12.5%, owing to patients’ insufficient confidence in the safety of domestically produced Chinese surgical robotic systems and telesurgery technology. Most of the patients who withdrew turned to using the da Vinci system, which was balanced in groups and had no significant influence on the statistical results.

### Comparison with other studies

This study was the first randomised controlled trial in the field of telesurgery to analyse the difference in reliability between· telesurgery and local surgery. Previous clinical trials have been limited to single arm uncontrolled studies or case reports, thus failing to provide robust evidence. This randomised controlled trial showed that the reliability of telesurgery was non-inferior to that of local surgery according to the non-inferiority margin of a 0.1 reduction in the probability success of, which provided stronger evidence for the further application of telesurgery.

### Conclusions

This randomised controlled trial showed that telesurgery was not inferior to conventional local robotic surgery, with a pre-specified margin of 0.1 for the probability of surgical success. We found no clear evidence of clinically important differences in the operative process, complications, or early recovery. This trial provides important evidence and reference for future larger cohort studies to explore the comprehensive benefits of telesurgery in clinical application.

## What is already known on this topic

Telesurgery has evolved over more than three decades, progressing from conceptual inception to advanced clinical exploration
However, despite the accumulation of many single arm and uncontrolled studies, robust evidence confirming its reliability remains scarce

## What this study adds

As the first randomised controlled trial in the field of telesurgery, this study establishes that its reliability is non-inferior to that of conventional local surgery
This finding provides a foundational evidence base for the design and implementation of larger scale clinical trials in the future

## Data Availability

The code used to analyse the data in the paper can be found in the supplementary materials. The data underlying the findings in this paper are openly and publicly available and can be found at Dryad: https://doi.org/10.5061/dryad.t4b8gtjg8. If you encounter problems accessing the data, please contact the corresponding author.

## Web extra

Extra material supplied by authors

Web appendix: Supplementary tables and figures

Web appendix: Trial protocol and statistical analysis plan

Web appendix: Statistical codes

## Notes

Contributors: YW, DX, WHX, MR, and WYZ are joint first authors. XZ was the chief investigator and oversaw the trial throughout. XZ and YW obtained funding. XZ, YW, XM, and HZL did the literature search, designed the study, interpreted the data, and wrote or reviewed the report. YW and WYZ were the trial coordinators (YW for clinical coordination; WYZ for surgical system coordination). XM, DX, WHX, MR, and CZL were principal investigators at each site and participated in recruitment and collection of data. ZX, XM, HZL, WHX, MR, CZL, SW, WJF, and QBH did the surgery. WYZ, XPZ, and JLM monitored the surgical robotic system, including the telesurgery system. TPS, BJW, ST, BZQ, YBZ, YSY, YZ, HLW, SYM, CY, TS, QA, YS, LHX, GQY, YG, and SXN collected the data, designed the study, and contributed to recruitment and treatment of patients. JG, GJL, and XYX collected and interpreted the data and contributed to the medical care and follow up. YW wrote the first draft of the manuscript. All authors have read and approved the final manuscript. XZ, HZL, XM, and CZL share last authorship. XZ and YW are the guarantors. The corresponding author attests that all listed authors meet authorship criteria and that no others meeting the criteria have been omitted.

Transparency: The lead authors (the manuscript’s guarantors) affirm that the manuscript is an honest, accurate, and transparent account of the study being reported; that no important aspects of the study have been omitted; and that any discrepancies from the study as planned (and, if relevant, registered) have been explained.

Dissemination to participants and related patient and public communities: The authors plan to disseminate the study and its results to research institutions, medical associations, and patient and public communities. The results will be presented at scientific meetings and conferences, as well as other opportunities for dissemination.

Provenance and peer review: Not commissioned; externally peer reviewed.

Publisher’s note: Published maps are provided without any warranty of any kind, either express or implied. BMJ remains neutral with regard to jurisdictional claims in published maps.
