# Supplementary material for: Reliability of urological telesurgery compared with local surgery: multicentre randomised controlled trial
Source: BMJ. 2026 Jan 28;392:e083588. doi: 10.1136/bmj-2024-083588 (PMC12849050; doi:10.1136/bmj-2024-083588)
Supplement: Supplementary file 1 — Web appendix: Supplementary tables and figures [file wany083588.ww1.pdf]

Supplementary Tables and Figures

Table of contents

Contents	Page
Table A. List of Telesurgery System framework details.	3
Table B Characteristics of the Baseline details of patients of renal tumor.	5
Table C. Characteristics of the Baseline details of patients of prostate cancer.	7
Table D. Surgeon and Patient location details.	8
Table E. Surgeon distribution details.	9
Table F. Sensitive analysis for primary outcome for ITT with vague prior distributions.	10
Table G. Summary for the Telesurgery monitoring data.	11
Table H. Summary Statistics for the Malfunction by Group.	14
Table I. List of Reported Outcomes Collected in This Trial.	15
Table J. Outcome Completion Summary for Per-protocol population.	18
Table K. Summary Statistics for the QoR15 Score by Group.	20
Table L. Summary Statistics for the 30 Second Chair to Stand test by Group.	21
Table M. Summary Statistics for the EPIC-26 Score by Group.	22
Table N. Summary Statistics for the surgical approach for Groups (Partial Nephrectomy and Prostatectomy)	22
Table O. Type of Complication at 4 weeks by Group.	23
Table P. Type of Complication at 6 weeks by Group.	23
Table Q. Type of Complication (Full Description during the follow-up period) by Group.	23

Table R. Summary Statistics for Oncological outcome by Group.	26
Table S. Sensitive Analysis for secondary outcomes by Group.	27
Table T. List of study team responsibility.	29
Table U. List of Investigators-Nonauthor Collaborators.	30
Figure A. Participant Flow of participants received partial nephrectomy in this trial.	32
Figure B. Participant Flow of participants received prostatectomy in this trial.	33
Figure C. Univariate linear regression of physical distance and round-trip network latency.	34
Figure D. Real-time round trip network latency between Beijing and Urumqi.	35
Figure E. Real-time display latency between Beijing and Urumqi.	36
Figure F. Real-time frame loss during the surgery between Beijing and Urumqi.	37
Figure G. Real-time round trip network latency between Beijing and Hangzhou.	38
Figure H. Real-time display latency between Beijing and Hangzhou.	39
Figure I. Real-time frame loss during the telesurgery from Beijing to Hangzhou.	40
Figure J. Real-time round trip network latency between Beijing and Harbin.	41
Figure K. Real-time display latency between Beijing and Harbin.	42
Figure L. Real-time frame loss during the telesurgery between Beijing and Harbin.	43
Figure M. Real-time round trip network latency between Beijing and Hefei.	44
Figure N. Real-time display latency between Beijing and Hefei.	45
Figure O. Real-time frame loss during the telesurgery between Beijing and Hefei.	46

Table A. List of Telesurgery System framework details.

Instrument Name (Model, Company)	Number		Functions
	Tele-Surgeon Room	Operation Room	
**Surgery robot Subsystem**			
Surgeon Console (MP1000，Edge Medical，China)	1	1	1. The console on the surgeon side is the main console, and the patient side one is the backup one;<br>2. Provide 3D endoscopic video image<br>3. Manipulate the instruments and endoscope at the surgical site
Patient Cart (MP1000，Edge Medical，China)	/	1	1. Support the instrument arms and camera arm<br>2. Perform patient-side activities
Vision System(MP1000，Edge Medical，China)	/	1	1. Connect and control endoscopes<br>2. Endoscopic image processing
**Teleconference Subsystem**			
Teleconference Box (AE600，XYLink，China)	1	2	1. Teleconference video and audio processing<br>2. Network transmission of teleconference video and audio
Teleconference Box (ME55s，XYLink，China)	1	/	1. Integrate the endoscopic image into teleconference video layout

Instrument Name (Model, Company)	Number		Functions
	Surgeon Side	Patient Side	
Camera (AC95，XYLink，China)	1	2	1. Capture surgery scene<br>2. Capture surgeon image<br>3. Capture the surgical site and arms of patient cart in the operation room
Microphone (SH26，XYLink，China)	1	1	1. Capture surgeon speaking<br>2. Capture assistant speaking<br>3. Capture the sound of energy portfolio
Screen (XR-65X91K，Sony，Japan)	1	1	1. Display teleconference video<br>2. Display teleconference audio
**Telecommunication Subsystem**			
Dedicated line (China Telecom, and China Unicom)	One dedicated line connect the tele-surgeon and patient side. OTN dedicated line*: Beijing-Urumqi, Beijing-Harbin; CCN dedicated line†: Beijing-Hangzhou, Beijing-Hefei		For the data packages transport
Telesurgery work station(TS0-001,Edge Medical，China)	1	1	1. Network transmission of control signal<br>2. Endoscopic video encoding and decoding<br>3. Network transmission of endoscopic video

[a] OTN: Optical transport network

[b] CCN: Cloud connect network

Table B. Characteristics of the Baseline details of patients of renal tumor.

Characteristic	Per-Protocal population		Intention-To-Treat population	
	Telesurgery Group (n=15)	Local Surgery Group (n=15)	Telesurgery Group (n=16)	Local Surgery Group (n=16)
**Sex，no.(%)**				
Male	7(46.7)	6(40)	8(50)	6(37.5)
Female	8(45.3)	9(60)	8(50)	10(62.5)
**Age, median (IQR), yr**	58(53.5-61)	51(48-61.5)	57（53.8-60.5）	53（48.5-65.3）
<40	2 (13.3)	1 (6.7)	2(12.5)	1(6.3)
40-59	8 (53.3)	10 (66.7)	9(56.3)	10(62.5)
≥60	5 (33.3)	4 (26.7)	5(31.2)	5(31.2)
**Height, median (IQR), m**	1.64(1.60-1.71)	1.65(1.60-1.74)	1.64(1.60-1.70)	1.65(1.60-1.74)
**Weight, median (IQR), kg**	70(64.5-77.5)	68(61.0-84.5)	70(63.5-76.3)	68.8(61.5-84.3)
**Body mass index，median (IQR) , kg/m2**	26.2(24.0-29.9)	25.4(23.3-28.3)	25.8(24.0-28.9)	26.0(23.4-28.0)
**Preoperative hemoglobin, median (IQR) , g/dL**	138(136-147)	139(128-151)	139(137-144)	139(129-150)
**Preoperative creatinine, median (IQR) , mg/dL**	67(57.5-80.1)	65(62.0-75.0)	67(59.3-78.7)	65(62.0-72.3)
**RENAL score**				
**Radius, no.(%)**				
Radius ≤ 4 cm	14 (93.3)	12 (80.0)	14(87.5)	13(81.3)
4 <Radius ≤7 cm	1 (6.7)	3 (20.0)	1(6.3)	3(18.8)
Radius≥7 cm	0	0	1(6.3)	0
**Exophytic property**				
Exophytic ≥50%	8 (53.3)	11 (73.3)	9(56.3)	11(68.8)
Exophytic <50%	5 (33.3)	4 (26.7)	5(31.3)	5(31.3)

Completely endophytic	2 (13.3)	0	2(12.5)	0
**Characteristic**	**Per-Protocal population**		**Intention-To-Treat population**	
	**Telesurgery Group (n=15)**	**Local Surgery Group (n=15)**	**Telesurgery Group (n=16)**	**Local Surgery Group (n=16)**
**Nearness of the deepest portion of the tumor to the collecting system, n（%）**				
≥7mm	4 (26.7)	11 (73.3)	4(25)	12(75)
4~7mm	7 (46.7)	2 (13.3)	8(50)	2(12.5)
≤4mm	4 (26.7)	2 (13.3)	4(25)	2(12.5)
**Anterior/Posterior descriptor，n（%）**				
A	4 (26.7)	8 (53.3)	4(25)	9(56.3)
P	9 (60.0)	5 (33.3)	10(62.5)	5(31.3)
X	2 (13.3)	2 (13.3)	2(12.5)	2(12.5)
**Location relative to the polar line**				
Entirely above the upper or below the lower polar line	9 (60.0)	7 (46.7)	9(56.3)	8(50)
Lesion crosses polar line	3 (20.0)	5 (33.3)	3(18.8)	5(31.2)
>50% of mass is across polar line	3 (20.0)	3 (20.0)	4(25)	3(18.8)
**Patient location，n(%)**				
Beijing	6 (40.0)	5（33.3)	6(37.5)	6(37.5)
Urumqi	2 (13.3)	2 (13.3)	2(12.5)	2(12.5)
Harbin	4（26.7)	2(13.3)	4(25)	4(25)
Hangzhou	2(13.3)	4（26.7)	2(12.5)	2(12.5)
Hefei	1（6.7)	2(13.3)	2(12.5)	2(12.5)

Table C. Characteristics of the Baseline details of patients of prostate cancer.

Characteristic	Per-Protocal population		Intention-To-Treat population	
	Telesurgery Group (n=17)	Local Surgery Group (n=16)	Telesurgery Group (n=20)	Local Surgery Group (n=20)
**Age，median(IQR) , yr**	66.0(60.0-68.0)	66.5(62-73.5)	65.5(60-68.3)	66.5(62-73)
40-59, no.(%)	2 (11.8)	2 (12.5)	2(10)	3(15)
≥60,no.(%)	15 (88.2)	14 (87.5)	18(90)	17(85)
**Height, median (IQR), m**	1.70 (1.68-1.73)	1.70 (1.65-1.75)	1.72 (1.68-1.73)	1.70 (16.7-1.75)
**Weight, median (IQR), kg**	70.0 (65.0-75.0)	75.0(68.0-80.2)	69.0 (65.0-75.0)	74.0 (65.0-79.4)
**Body mass index，median(IQR), kg/m2**	24.7 (22.1-27.0)	25.8(24.4-26.9)	24.1 (22.2-26.4)	25.3 (24.3-26.9)
**Preoperative hemoglobin, median (IQR), g/dL**	143 (135-146)	141 (131-147)	143 (135-154)	141 (132-147)
**Preoperative PSA, median (IQR), ng/mL**	9.7 (6.6-11.9)	10.4 (4.9-15.5)	9.2 (6.4-12.0)	10.4 (4.9-13.7)
**Volume of Prostate, median (IQR), ml**	35.5 (29.3-40.4)	26.6 (22.0-59.1)	36.3 (28.9-46.6)	34.1 (24.9-48.8)
**TNM stage**				
**T stage, no. (%)**				
T2	15 (88.2)	13 (81.4）	18(90)	17(85)
T3	2 (11.8)	3 (17.6)	2(10)	3(15)
**N stage, no. (%)**				
N0	8 (47.1）	7 (43.8)	9(45)	10(50)
N1	1 (5.9)	1 (6.3)	1(5)	1(5)
Nx	8 (47.1)	8 (50)	10(50)	9(45)
**M stage, no. (%)**				
M0	11 (64.7)	9 (56.3)	12(60)	12(60)
M1	1 (5.9)	1 (6.3)	1(5)	1(5)
MX	5 (29.4)	6 (37.5)	7(35)	7(35)
**Patient location，no.（%）**				
Beijing	6 (35.3)	6 (37.5)	6(30)	6(30)
Urumqi	4 (23.5)	2 (12.5)	4(20)	4(20)
Harbin	2 (11.8)	1 (6.3)	2(10)	2(10)
Hangzhou	2 (11.8)	4 (25)	4(20)	4(20)
Hefei	3 (17.6)	3 (18.8)	4(20)	4(20)

Table D. Surgeon and Patient location details.					
NO.	Location of Surgeon	Surgeon Name	Location of Patient	Operation	Group
1	Hefei	Chaozhao Liang	Hefei	Partial Nephrectomy	Local Sugery Group
2	Hefei	Chaozhao Liang	Hefei	Partial Nephrectomy	Local Sugery Group
3	Hefei	Baojun Wang	Hefei	Radical prostatectomy	Telesurgery Group
4	Hefei	Xu Zhang	Hefei	Radical prostatectomy	Telesurgery Group
5	Hefei	Sheng Tai	Hefei	Radical prostatectomy	Local Sugery Group
6	Beijing	Hongzhao Li	Hefei	Radical prostatectomy	Telesurgery Group
7	Beijing	Xin Ma	Hefei	Partial Nephrectomy	Telesurgery Group
8	Hefei	Chaozhao Liang	Hefei	Radical prostatectomy	Local Sugery Group
9	Hefei	Sheng Tai	Hefei	Radical prostatectomy	Local Sugery Group
10	Harbin	Wanghai Xu	Harbin	Partial Nephrectomy	Local Sugery Group
11	Beijing	Qingbo Huang	Harbin	Partial Nephrectomy	Telesurgery Group
12	Beijing	Hongzhao Li	Harbin	Radical prostatectomy	Telesurgery Group
13	Beijing	Xin Ma	Harbin	Partial Nephrectomy	Telesurgery Group
14	Harbin	Wanghai Xu	Harbin	Partial Nephrectomy	Local Sugery Group
15	Harbin	Wanghai Xu	Harbin	Radical prostatectomy	Local Sugery Group
16	Beijing	Xin Ma	Harbin	Radical prostatectomy	Telesurgery Group
17	Harbin	Wanghai Xu	Beijing	Radical prostatectomy	Telesurgery Group
18	Hefei	Chaozhao Liang	Beijing	Radical prostatectomy	Telesurgery Group
19	Beijing	Weijun Fu	Beijing	Radical prostatectomy	Local Sugery Group
20	Beijing	Xin Ma	Beijing	Partial Nephrectomy	Local Sugery Group
21	Beijing	Xin Ma	Beijing	Partial Nephrectomy	Local Sugery Group
22	Beijing	Weijun Fu	Beijing	Radical prostatectomy	Local Sugery Group
23	Harbin	Wanghai Xu	Beijing	Partial Nephrectomy	Telesurgery Group
24	Hefei	Chaozhao Liang	Beijing	Partial Nephrectomy	Telesurgery Group
25	Beijing	Weijun Fu	Beijing	Radical prostatectomy	Local Sugery Group
26	Hangzhou	Shuo Wang	Beijing	Radical prostatectomy	Telesurgery Group
27	Hefei	Chaozhao Liang	Beijing	Partial Nephrectomy	Telesurgery Group
28	Hangzhou	Shuo Wang	Beijing	Radical prostatectomy	Telesurgery Group
29	Beijing	Xin Ma	Beijing	Partial Nephrectomy	Local Sugery Group
30	Hangzhou	Shuo Wang	Beijing	Partial Nephrectomy	Telesurgery Group
31	Hangzhou	Shuo Wang	Beijing	Radical prostatectomy	Telesurgery Group
32	Urumqi	Qingbo Huang	Beijing	Partial Nephrectomy	Telesurgery Group
33	Urumqi	Qingbo Huang	Beijing	Partial Nephrectomy	Telesurgery Group
34	Beijing	Xin Ma	Beijing	Partial Nephrectomy	Local Sugery Group
35	Urumqi	Weijun Fu	Beijing	Radical prostatectomy	Telesurgery Group
36	Beijing	Xin Ma	Beijing	Partial Nephrectomy	Local Sugery Group

37	Beijing	Weijun Fu	Beijing	Radical prostatectomy	Local Sugery Group
38	Beijing	Xin Ma	Hangzhou	Partial Nephrectomy	Telesurgery Group
39	Hangzhou	Shuo Wang	Hangzhou	Partial Nephrectomy	Local Sugery Group
40	Hangzhou	Shuo Wang	Hangzhou	Partial Nephrectomy	Local Sugery Group
41	Beijing	Xin Ma	Hangzhou	Partial Nephrectomy	Telesurgery Group
42	Hangzhou	Shuo Wang	Hangzhou	Partial Nephrectomy	Local Sugery Group
43	Beijing	Xin Ma	Hangzhou	Partial Nephrectomy	Telesurgery Group
44	Hangzhou	Shuo Wang	Hangzhou	Partial Nephrectomy	Local Sugery Group
45	Beijing	Xin Ma	Hangzhou	Partial Nephrectomy	Telesurgery Group
46	Hangzhou	Shuo Wang	Hangzhou	Radical prostatectomy	Local Sugery Group
47	Beijing	Hongzhao Li	Hangzhou	Radical prostatectomy	Telesurgery Group
48	Hangzhou	Shuo Wang	Hangzhou	Radical prostatectomy	Local Sugery Group
49	Hangzhou	Shuo Wang	Hangzhou	Radical prostatectomy	Local Sugery Group
50	Beijing	Hongzhao Li	Hangzhou	Radical prostatectomy	Telesurgery Group
51	Hangzhou	Shuo Wang	Hangzhou	Radical prostatectomy	Local Sugery Group
52	Beijing	Weijun Fu	Beijing	Radical prostatectomy	Local Sugery Group
53	Urumqi	Baojun Wang	Urumqi	Radical prostatectomy	Local Sugery Group
54	Urumqi	Mulati Rexiati	Urumqi	Radical prostatectomy	Local Sugery Group
55	Beijing	Xin Ma	Urumqi	Partial Nephrectomy	Telesurgery Group
56	Beijing	Hongzhao Li	Urumqi	Radical prostatectomy	Telesurgery Group
57	Beijing	Baojun Wang	Urumqi	Radical prostatectomy	Telesurgery Group
58	Beijing	Hongzhao Li	Urumqi	Radical prostatectomy	Telesurgery Group
59	Beijing	Hongzhao Li	Urumqi	Radical prostatectomy	Telesurgery Group
60	Urumqi	Qingbo Huang	Urumqi	Partial Nephrectomy	Local Sugery Group
61	Urumqi	Qingbo Huang	Urumqi	Partial Nephrectomy	Local Sugery Group
62	Beijing	Qingbo Huang	Urumqi	Partial Nephrectomy	Telesurgery Group
63	Beijing	Xu Zhang	Beijing	Radical prostatectomy	Local Sugery Group

Table E. Surgeon distribution details.

	Telesurgery Group			Local Surgery Group		
**Surgeon**	**Total, no.(%)**	Radical prostatectomy	Partial Nephrectomy	**Total, no.(%)**	Radical prostatectomy	Partial Nephrectomy
Chaozhao Liang	**3 (9.4)**	1	2	**3 (9.7)**	1	2
Baojun Wang	**2 (6.3)**	2	0	**1 (3.2)**	1	0
Xu Zhang	**1 (3.1)**	1	0	**1 (3.2)**	1	0
Sheng Tai	**0**	0	0	**2 (6.5)**	2	0
Hongzhao Li	**7 (21.9)**	7	0	**0**	0	0
Wanghai Xu	**2 (6.3)**	1	1	**3 (9.7)**	1	2

Xin Ma	**8 (25.0)**	1	7	**5 (16.1)**	0	5
Qingbo Huang	**4 (12.5)**	0	4	**2 (6.5)**	0	2
Weijun Fu	**1 (3.1)**	1	0	**5 (16.1)**	5	0
Shuo Wang	**4 (12.5)**	3	1	**8 (25.8)**	4	4
Mulati Rexiati	**0**	0	0	**1 (3.2)**	1	0

Table F. Sensitive analysis for primary outcome for ITT with vague prior distributions.

**Success Probability of Surgery，success no./ total no. (%)**	**Telesurgery**	**Local Surgery**	**Success Probability difference**	**95% CrIs**	**P(difference in success probability > -0.1)**	**Non-inferiority hypothesis**
Half-normal priors [a]						
Adjusted for Surgeon (random)	36/36(100)	34/36(94.4)	0.034	-0.079 to 0.20	0.98388	valid
Adjusted for Hospital	36/36(100)	34/36(94.4)	0.016	-0.024 to 0.14	0.99819	valid
Adjusted for Surgery Type	36/36(100)	34/36(94.4)	0.051	-0.083 to 0.25	0.98257	valid
Exponential priors [b]						
Adjusted for Surgeon (random)	36/36(100)	34/36(94.4)	0.041	-0.077 to 0.19	0.98549	valid
Adjusted for Hospital	36/36(100)	34/36(94.4)	0.016	-0.024 to 0.12	0.99819	valid
Adjusted for Surgery Type	36/36(100)	34/36(94.4)	0.051	-0.083 to 0.25	0.98257	valid
Uniform priors[c]						
Adjusted for Surgeon (random)	36/36(100)	34/36(94.4)	0.034	-0.081 to 0.21	0.98304	valid
Adjusted for Hospital	36/36(100)	34/36(94.4)	0.016	-0.024 to 0.12	0.99819	valid
Adjusted for Surgery Type	36/36(100)	34/36(94.4)	0.051	-0.083 to 0.25	0.98257	valid

a: **Prior for Random Effects SD** : normal(0, 5).

b: **Prior for Random Effects SD** : exponential(1).

c: **Prior for Random Effects SD** : uniform(0, 10).

Table G. Summary for the Telesurgery monitoring data

Telesurgery pathway	Case NO. of Each site								
**Beijing-Urumqi**	**case 1**	**case 2**	**case 3**	**case 4**	**case 5**	**case 6**	**case 7**	**case 8**	**case 9**
Round-trip network latency,ms,median (IQR)	47.53 (47.49-47.57)	47.53 (47.49-47.58)	47.53 (47.49-47.57)	47.53 (47.49-47.57)	47.54 (47.51-47.59)	47.53 (47.50-47.58)	47.53 (47.50-47.58)	47.53 (47.50-47.58)	47.54 (47.50-47.58)
Display latency,ms,median (IQR)*	20.01 (19.99-20.03)	20.01 (20.00-20.03)	20.01 (20.00-20.04)	20.01 (20.00-20.03)	20.01 (19.99-20.03)	20.01 (19.99-20.03)	20.01 (19.99-20.03)	Missing	Missing
Frame loss,total number *	0	0	0	0	0	0	0	Missing	Missing
First surgeon location	Beijing	Beijing	Beijing	Beijing	Urumqi	Urumqi	Urumqi	Beijing	Beijing
Operation type	Prostatectomy	Partial nephrectomy	Prostatectomy	Partial nephrectomy	Prostatectomy	Partial nephrectomy	Partial nephrectomy	Prostatectomy	Prostatectomy
**Beijing-Harbin**	**case 1**	**case 2**	**case 3**	**case 4**	**case 5**	**case 6**			
Round-trip network latency,ms,median (IQR)	22.77 (22.73-22.81)	22.84 (22.80-22.88)	22.84 (22.80-22.88)	22.74 (22.73-22.82)	22.86 (22.82-22.90)	22.85 (22.82-22.90)			

Display latency,ms,median (IQR)	20.01 (19.99-20.03)	20.01 (19.98-20.03)	20.01 (20.00-20.03)	20.02 (20.00-20.03)	20.00 (19.98-20.02)	20.01 (19.98-20.03)				
Frame loss,total number(%)	1(0.040%)	0	3(0.085%)	0	0	3(0.044%)				
First surgeon location	Harbin	Harbin	Beijing	Beijing	Beijing	Beijing				
Operation type	Partial nephrectomy	Prostatectomy	Partial nephrectomy	Partial nephrectomy	Prostatectomy	Prostatectomy				
**Telesurgery pathway**	**Case NO. of Each site**									
**Beijing-Hangzhou**	**case 1**	**case 2**	**case 3**	**case 4**	**case 5**	**case 6**	**case 7**	**case 8**	**case 9**	**case 10**
Round-trip network latency,ms,median (IQR)	38.98 (38.93-39.04)	38.78 (38.73-38.84)	38.79 (38.74-38.85)	26.46 (26.40-26.53)	26.46 (26.41-26.54)	27.03 (26.98-27.09)	27.52 (27.47-27.58)	27.51 (27.45-27.59)	27.32 (27.26-27.40)	27.32 (27.26-27.40)
Display latency,ms,median (IQR)	20.01 (19.99-20.03)	20.01 (19.99-20.03)	20.01 (19.99-20.03)	20.01 (19.99-20.03)	20.01 (19.99-20.03)	20.01 (19.99-20.03)	20.01 (19.99-20.04)	20.01 (19.99-20.03)	20.01 (19.99-20.03)	20.01 (19.99-20.04)
Frame loss,total number(%)	3(0.070%)	1(0.23%)	2(0.052%)	0	0	0	3(0.077%)	2(0.049%)	2(0.092%)	1(0.024%)
First surgeon location	Hangzhou	Beijing	Hangzhou	Hangzhou	Beijing	Hangzhou	Beijing	Beijing	Beijing	Beijing
Operation type	Prostatectomy	Partial nephrectomy	Partial nephrectomy	Prostatectomy	Prostatectomy	Prostatectomy	Prostatectomy	Partial nephrectomy	Partial nephrectomy	Partial nephrectomy

Beijing-Hefei	case 1	case 2	case 3	case 4	case 5	case 6	case 7
Round-trip network latency,ms,median (IQR)	20.19 (20.09-20.59)	20.23 (20.11-20.69)	20.08 (19.96-20.49)	20.08 (19.96-20.47)	20.10 (19.99-20.54)	20.12 (20.01-20.57)	20.13 (20.01-20.57)
Display latency,ms,median (IQR)	19.99 (19.84-20.14)	20.01 (19.88-20.14)	20.01 (19.87-20.14)	20.00 (19.88-20.14)	20.02 (19.88-20.16)	20.02 (19.89-20.13)	20.01 (19.86-20.15)
Frame loss,total number	0	0	0	2(0.11%)	1(0.021%)	0	0
First surgeon location	Beijing	Beijing	Hefei	Hefei	Beijing	Beijing	Hefei
Operation type	Prostatectomy	Prostatectomy	Prostatectomy	Partial nephrectomy	Prostatectomy	Partial nephrectomy	Partial nephrectomy

Table H. Summary Statistics for the Malfunction by Group

Randomized group	Total (n=63)	Telesurgery (n=32)	Local Surgery (n=31)
**Malfunction**			
During the surgery, no.(%)	0	0	0
During the test period, no.(%)	1(1.6) *	0	1(3.3)

*The occurrence of the only one malfunction of the robotic system in the local surgery group before the patient was ready for anaesthesia in the operation room. In this instance, an external system connection cable was crushed by equipment, and the manufacturer provided a replacement cable 2 days later. Then this case received surgery according to the former randomize group, and the surgery was also performed effectively. Total malfunction rate of surgical robot system was 98.4%, including the robot system of both groups.

Table I. List of Reported Outcomes Collected in This Trial

Primary Outcome	Necessary Explanation
Probability of success of surgery	The success is confirmed according to the following determination points: (1) The surgical process is carried out according to the planned steps; (2) No obvious injury to large blood vessels or adjacent organs during the surgery; (3) No conversion of the surgical method, such as switching from telesurgery to local robotic surgery, or converting local robotic surgery to laparoscopic surgery or open surgery; (4) The surgery proceeds as planned, and no postponement due to surgical system malfunction. The success of each case will be jointly confirmed by the medical team together by these pre-specified determination points after the surgery.
**Secondary Outcomes**	**Necessary Explanation**
**Early Recovery**	
1.Overall functional recovery	QoR-15 Score (Quality of recovery 15 items score):questionnaire at baseline (pre-operative), 4 weeks, 6 weeks.
2.Overall functional recovery for prostate cancer patient	EPIC-26 Score (Expanded Prostate Cancer Index Composite-26):questionnaire at baseline (pre-operative), 4 weeks, 6 weeks.
3. Physical activity	30 Second Chair to Stand test: Number times the patient can stand from sitting in a 30 second interval. This was tested at baseline (pre-operative), 4 weeks, 6 weeks.
**Secondary Outcomes**	**Necessary Explanation**

**Surgery Details**	
4. Surgery details	Surgical details that reflect the quality of surgery were recorded. Reported metrics include intraoperative blood loss, operative time, and warm ischemia time (for partial nephrectomy). Operative time is from the establishment of the Troca channel to the completion of skin suturing, which will be obtained from the patient's medical records.
**Perioperative morbidity**	
5.Complications and adverse events	Adverse events recorded using the Clavien-Dindo classification
6.Postoperative hospitalization days	Obtained from the patient's medical records.
7.Length of days in critical care	Obtained from the patient's medical records.
8.Reoperation intervention	Obtained from the patient's medical records.
9.Re-admission to hospital	Re-admission to hospital for any reasons was recorded. And, the surgery associated condition was systemically reviewed.
10.Intra and post-operative blood transfusion rates	Obtained from the patient's medical records.
11.Mortality rate	Obtained from the patient's medical records and Postoperative follow-up.
**Oncological outcomes**	
12.Positive margin rate in the pathological specimen	According to the pathology report from two blinded pathologists in each hospital.
13.Pathological type of tumor and grading of pathological malignancy	For the renal cancer and prostate cancer participants.

Secondary Outcomes	Necessary Explanation
**Task load of medical team**	
14.NASA Task load index	The NSAS Task load index was questionnaired to the first surgeon, first assistant and instrument nurse after the surgery immediately.
**Surgery System monitoring**	
15.Round-trip network latency (For telesurgery)	The round-trip network latency is defined as the time latency of data package from sending and receiving.
16.Display latency (For telesurgery)	The display latency is defined as the time latency for coding and encoding of the endoscope image.
17.Frame loss (For telesurgery)	The frame loss is defined as data package loss during the data transport.
18.Malfunction	Malfunctions of the local surgical robot or telesurgery system were recorded. The local surgical robot or telesurgery system was systematically tested 1 day before the surgery. A malfunction of the telesurgery system is defined as the system becoming uncontrollable for any reason, such as telecommunication pathway faults, laboratory pathway faults, and robotic faults. A malfunction of a local surgical robot is defined similarly as the robot becoming uncontrollable for any reason.

Table J. Outcome Completion Summary for Per-protocol population.

Outcomes	Summary		Overall
	Telesurgery Group	Local Surgery Group	
**Primary Outcome**			
Surgery outcome record; no.(%)	32(100)	31(100)	63(100)
**Secondary Outcomes**			
**Early Recovery**			
Overall functional recovery-QoR 15 scale			
Baseline; no.(%)	32(100)	31(100)	63(100)
4-week follow up; no.(%)	32(100)	31(100)	63(100)
6-week follow up; no.(%)	31(96.9)	28(90.3)	59(93.7)
Overall functional recovery for prostate cancer patient-EPIC 26 scale			
Baseline; no.(%)	17(100)	16(100)	33(100)
4-week follow up; no.(%)	17(100)	16(100)	33(100)
6-week follow up; no.(%)	17(100)	15(93.8)	32(97.0)
Physical activity-30 Second Chair to Stand test			
Baseline; no.(%)	32(100)	31(100)	63(100)
4-week follow up; no.(%)	31(96.9)	26(83.9)	57(90.5)
6-week follow up; no.(%)	30(93.8)	23(74.2)	53(84.1)
**Surgery Details**			
Surgery details	32(100)	31(100)	63(100)
**Perioperative morbidity**			
Complications and adverse events			
4-week follow up; no.(%)	32(100)	31(100)	63(100)
6-week follow up; no.(%)	31(96.9)	28(90.3)	59(93.7)
Postoperative hospitalization days	32(100)	31(100)	63(100)
Length of days in critical care	32(100)	31(100)	63(100)
Reoperation intervention			
4-week follow up; no.(%)	32(100)	31(100)	63(100)
6-week follow up; no.(%)	31(96.9)	28(90.3)	59(93.7)
Re-admission to hospital			
4-week follow up; no.(%)	32(100)	31(100)	63(100)
6-week follow up; no.(%)	31(96.9)	28(90.3)	59(93.7)
Intra and post-operative blood transfusion rates			
4-week follow up; no.(%)	32(100)	31(100)	63(100)

6-week follow up; no.(%)	31(96.9)	28(90.3)	59(93.7)
**Outcomes**	**Summary**		**Overall**
	**Telesurgery Group**	**Local Surgery Group**	
Mortality rate			
4-week follow up; no.(%)	32(100)	31(100)	63(100)
6-week follow up; no.(%)	32(100)	31(100)	63(100)
**Oncological outcomes**			
Positive margin rate in the pathological specimen; no.(%)	32(100)	31(100)	63(100)
Pathological type of tumor and grading of pathological malignancy; no.(%)	32(100)	31(100)	63(100)
**Task load of medical team**			
NASA Task load index			
Surgeon; no.(%)	25 (78.1)	27 (87.1)	52 (82.5)
First assistant; no.(%)	28 (87.5)	28 (90.3)	56 (88.9)
Instrument nurse; no.(%)	28 (87.5)	28 (90.3)	56 (88.9)
**Surgery system monitoring**			
Round-trip network latency (For telesurgery); no.(%)	32(100)	—	—
Display latency (For telesurgery); no.(%)	30(93.8)	—	—
Frame loss (For telesurgery); no.(%)	30(93.8)	—	—
Malfunction; no.(%)	32(100)	31(100)	63(100)

Table K. Summary Statistics for the QoR15 Score by Group.

Randomized group		Telesurgery group	Local Surgery group	p-value	Adjusted Mean Difference（95% CI）
**Total**					
Baseline		147.5(141.8-150)	148(145-150)	0.10	1.60(-5.87 to 0.53)
	Missing	0	0		
4 weeks		146.0(136.8-149.0)	145 (138-149)	0.65	2.07(-5.08 to 3.22)
	Missing	0	0		
6 weeks		147.0(140.0-150.0)	149.0 (144-150)	0.064	8.18(0.94 to 31.82)
	Missing	1	4		
**Renal Tumor**					
Baseline		149（139-150）	149（146-150）	0.12	2.45(-9.02 to 1.05)
	Missing	0	0		
4 weeks		146（137.5-149)	146(139.5-149)	-0.62	2.45(-9.03 to 1.05)
	Missing	0	0		
6 weeks		148(146-150)	149.5(145-150)	0.13	18.28 (-31.69 to 43.60)
	Missing	1	3		
**Prostate cancer**					
Baseline		147(144-150)	147.5(142.5-150)	0.61	1.20(-5.13 to 3.09)
	Missing	0	0		
4 weeks		147(137-148)	145(137.8-149.3)	0.96	3.20(-6.38 to 6.67)
	Missing	0	0		
6 weeks		144（139-149）	146（140-150）	0.32	8.77(-9.17 to 26.83)
	Missing	0	1		

Table L. Summary Statistics for the 30 Second Chair to Stand test by Group

**Randomized**		**Telesurgery**	**Local Surgery**	**p-value**	**Adjusted Mean Difference (95% CI)**
**Total**					
Baseline		15（13-16.3）	14（12-16）	0.10	1.11(-4.07 to 0.38)
	Missing	0	0		
4 weeks		15（12-16）	14（12-16）	0.24	1.47(-4.73 to 1.19)
	Missing	1	5		
6 weeks		14（12-20）	15（12.3-16）	0.77	1.74(-3.99 to 2.97)
	Missing	2	8		
**Renal Tumor**					
Baseline		15(13-16.5)	15(13.5-16)	0.12	2.45(-9.02 to 1.05)
	Missing	0	0		
4 weeks		15(12-15.8)	15(13.3-16)	0.74	2.15 (-5.12 to 3.69)
	Missing	0	1		
6 weeks		16(12-20)	15.5(14.8-20)	0.59	2.48 (-3.73 to 6.47)
	Missing	2	3		
**Prostate cancer**					
Baseline		15（13-16）	14（12-15）	0.32	1.88 (-5.75 to 1.93)
	Missing	0	0		
4 weeks		14（12-16）	13（9.5-14.5）	0.21	2.05 (-6.86 to 1.56)
	Missing	0	4		
6 weeks		14（13-16）	13（11-16）	0.70	2.52(-6.17 to 4.22)
	Missing	0	5		

Table M. Summary Statistics for the EPIC-26 Score by Group

Randomized group		Telesurgery	Local Surgery	p-value	Adjusted Mean Difference (95% CI)
**Total**					
Baseline		52(40-58)	51(44-58.5)	0.41	6.44(-7.78, 18.49)
	Missing	0	0		
4 weeks		64(56-70)	59.5(55-68)	0.35	4.23(-4.67, 12.77)
	Missing	0	0		
6 weeks		56(47-67)	59(51.5-64)	0.12	5.72(-2.64, 20.74)
	Missing	0	1		
**Urinary control** [a]					
Baseline		4(4-6)	4(4-5.3)	0.93	0.064(-1.52, 1.65)
4 weeks		14(9-16)	12.5(9-15.3)	0.66	0.55 (-1.99, 3.10)
6weeks		10(6-13)	12(8.5-12.5)	0.69	0.61 (-2.46, 3.68)
**Sexual function** [b]					
Baseline		19(14-26)	20(17-25)	0.52	1.36 (-2.91, 5.63)
4 weeks		26(25-27)	26(25.8-28)	0.17	1.34 (-0.61, 3.29)
6 weeks		26(25-27)	26(24-27)	0.15	2.70 (-1.08, 6.46)

[a] Items 1 to 4 of EPIC-26 scale. [b] Items 16 to 21 of EPIC-26 scale

Table N. Summary Statistics for the surgical approach for Groups

Randomized group	Telesurgery	Local Surgery
**Partial Nephrectomy**		
Surgical approach		
Retroperitoneal, no.(%)	13(86.7)	10(66.7)
Transperitoneal, no.(%)	2(13.3)	5(33.3)
**Prostatectomy**		
Surgical approach		
Transabdominal, no.(%)	14(82.4)	12(75)
Extraperitoneal, no.(%)	3(17.6)	4(25)
Lymph node dissection		

	Yes, no.(%)	6(35.3)	7(43.8)
	No, no.(%)	11(64.7)	9(56.2)

Table O. Type of Complication at 4 weeks by Group. [a]

Randomized group	Telesurgery	Local Surgery	p-value	95% CI
65. Other (specify)	3 (9.4) [b]	1 (3.2) [c]	0.26	0.32 to 0.94

[a] All levels of complications were reported in this table.

b Perineal discomfort: 2 patients; Shoulder pain:1 patient.

[c] Lateral lower back pain:1 patient

Table P. Type of Complication at 6 weeks by Group.

Randomized group	Telesurgery	Local Surgery	p-value	95% CI
4. Cardiac - Angina	1(3.1) [a]	0	0.18	0.21 to 0.97
65. Other (specify)	3(12.5) [b]	1(6.5) [c]		

[a] One patient in telesurgery group suffered myocardial infarction, and received percutaneous coronary intervention in Beijing Anzhen Hospital.

[b] Perineal discomfort: 2 patients; Shoulder pain:1 patient.

[c] Lateral lower back pain:1 patient

Table Q. Type of Complication (Full Description during the follow-up period) by Group

Surgical complications, no.(%)	Telesurgery	Local Surgery
1. Bleeding - Anaemia requiring transfusion	0	0
2. Bleeding - Post-operative bleeding other than GI	0	0
3. Bleeding - Wound hematoma	0	0
4. Cardiac - Angina	1(3.1)[a]	0
5. Cardiac - Arrhythmia	0	0

6. Cardiac - Congestive heart failure	0	0
7. Cardiac - Hypertension	0	0
8. Cardiac - Hypotension	0	0
9. Cardiac - Myocardial infarction	0	0
10. Gastrointestinal - Anastomotic bowel leak	0	0
11. Gastrointestinal - Clostridium difficile colitis	0	0
12. Gastrointestinal - Constipation	0	0
13. Gastrointestinal - Diarrhoea	0	0
14. Gastrointestinal - Emesis	0	0
15. Gastrointestinal - Gastrointestinal bleeding	0	0
16. Gastrointestinal - Ileus	0	0
17. Gastrointestinal - Small bowel obstruction	0	0
18. Gastrointestinal - Uretero-ileal obstruction	0	0
19. Infection - Abscess	0	0
20. Infection - Fever of unknown origin	0	0
21. Infection - Systemic sepsis	0	0
22. Infection - Urosepsis	0	0
23. Infection - Urinary tract infection	0	0
24. Genitourinary - Renal failure	0	0
25. Genitourinary - Haematuria	0	0
26. Genitourinary - Stomal ischemia	0	0
27. Genitourinary - Ureteral obstruction / RUT	0	0
28. Genitourinary - Urinary leak [b]	0	0
29. Genitourinary - Urinary fistula	0	0
30. Genitourinary - Urinary retention	0	0
31. Neurological - CVA	0	0
32. Neurological - Delirium/agitation	0	0
33. Neurological - Loss of consciousness	0	0
34. Neurological - Peripheral neuropathy	0	0
35. Neurological - Seizure	0	0
36. Neurological - TIA	0	0
37. Neurological - Vertigo	0	0
38. Miscellaneous - Acidosis	0	0
**Surgical complications, no.(%)**	**Telesurgery**	**Local Surgery**
39. Miscellaneous - Decubitis ulcer	0	0
40. Miscellaneous - Dehydration	0	0
41. Miscellaneous - Lymphocele	0	0
42. Miscellaneous - Peripheral arterial ischaemia	0	0
43. Miscellaneous - Psychological illness	0	0
44. Miscellaneous - Thrombocytopenia	0	0
45. Pulmonary - Atelectasis	0	0
46. Pulmonary - Pleural effusion	0	0

47. Pulmonary - Pneumonia	0	0
48. Pulmonary - Pneumothorax	0	0
49. Pulmonary - Respiratory distress	0	0
50. Surgical - Bowel injury	0	0
51. Surgical - Incisional hernia	0	0
52. Surgical - Port-site hernia	0	0
53. Surgical - Retained foreign body	0	0
54. Surgical - Vascular injury	0	0
55. Surgical - Visceral injury	0	0
56. Thromboembolic - DVT	0	0
57. Thromboembolic - Pulmonary embolism	0	0
58. Thromboembolic - Superficial phlebitis	0	0
59. Wound - Hernia	0	0
60. Wound - Wound dehiscence deep (facial)	0	0
61. Wound - Wound dehiscence superficial	0	0
62. Wound - Wound infection	0	0
63. Wound - Wound sepsis	0	0
64. Wound - Wound seroma	0	0
65. Other (specify)	3 (9.4) [c]	1 (3.2) [d]

[a] One patient in telesurgery group suffered myocardial infarction， and received percutaneous coronary intervention in Beijing Anzhen Hospital.

[b] The urinary leak is a normal phenomenon of patients received prostatectomy. So. this complication is not recorded as the complication for prostate cancer patients, whose urinary control function can be analyzed by the EPIC-26 scale.

[c] Perineal discomfort: 2 patients; Shoulder pain:1 patient.

[d] Lateral lower back pain:1 patient

Table R. Summary Statistics for Oncological outcome details by Group

Randomized group	Telesurgery	Local Surgery	Oddis Rate （95% CI）	p-value
**Positive margin rate(total)**				
Total(%)	1(3.1)	5(16.1)		
Univariate model (group only)			0.17 (0.018-1.53)	0.11
Exact logistic regression			0.17 (0.007-1.34)	0.10
**Renal Tumor**				
**Pathological classification**				
Clear cell carcinoma, no.(%)	12 (80.0)	8 (53.3)	-	-
Chromophobe cell carcinoma, no.(%)	1 (6.7)	3 (20.0)	-	-
Oncocytic carcinoma, no.(%)	0	1 (6.7)	-	-
Hamartoma, no.(%)	1 (6.7)	2 (13.3)	-	-
Other type carcinoma, no.(%) [a]	1 (6.7)	1 (6.7)	-	-
**Prostate Cancer**				
**Gleason score**				
3+3, no.(%)	2 (11.8)	2 (12.5)	-	-
3+4, no.(%)	7 (41.2)	4 (25.0)	-	-
4+3, no.(%)	3 (17.6)	3 (18.8)	-	-
4+4, no.(%)	0	3 (18.8)	-	-
4+5, no.(%)	2 (11.8)	2 (12.5)	-	-
5+4, no.(%)	1 (5.9)	0	-	-
Unable to rate , no.(%) [b]	2 (11.8)	2 (12.5)	-	-

[a] Including TFE3-rearranged renal cell carcinomas, renal epithelioid cell tumor, juxtaglomerular cell tumor.

[b] Patient were treated with androgen-deprivation therapy, and the prostate cancer regressed and Gleason score evaluation was not possible.

Table S. Sensitive Analysis for secondary outcomes by Group

Secondary outcomes	Adjusted Risk Difference (95% CI)	P-value
**Operative time**		
Adjusted for Hospital Only	-11.88(-44.24 to 20.48)	0.47
Adjusted for Surge type Only	-10.16(-34.67 to 14.35)	0.41
Adjusted for Surgeon Only	7.85(-19.86 to 35.56)	0.57
**Warm ischemia time**		
Adjusted for Hospital Only	2.93(-8.83 to 3.30)	0.36
Adjusted for Surgeon Only	2.86(2.86 to 3.73)	0.45
**Blood loss**		
Adjusted for Hospital Only	-0.27(-39.79 to 39.25)	0.99
Adjusted for Surge type Only	3.38(-35.79 to 42.54)	0.86
Adjusted for Surgeon Only	2.22(-44.80 to 49.23)	0.92
**Postoperative hospitalization days**		
Adjusted for Hospital Only	-0.35(-1.36 to 0.66)	0.49
Adjusted for Surge type Only	-0.25(-1.44 to 0.93)	0.67
Adjusted for Surgeon Only	-0.17(-1.57 to 1.23)	0.81
**Length of days in critical care**		
Adjusted for Hospital Only	-0.0129 (-0.098 to 0.072)	0.76
Adjusted for Surge type Only	-0.0019 (-0.091 to 0.087)	0.97
Adjusted for Surgeon Only	0.028 (-0.038 to 0.095)	0.40
**Positive margin rate [a]**		
Mixed effect model (Surgeon as fixed effect)	0.23(0.024 to 2.17)	0.20
Adjusted for Hospital Only	0.28(0.050 to 1.57)	0.15
Adjusted for Surge type Only	0.193(0.028 to 1.34)	0.096
Adjusted for Surgeon Only	0.20 (0.023 to 1.71)	0.14
**QoR15 Score**		
**Baseline**		
Adjusted for Hospital Only	-2.63(-5.46 to 0.21)	0.069

Adjusted for Surge type Only	-2.14(-5.35 to 1.07)	0.19
Adjusted for Surgeon Only	-3.23(-6.79 to 0.32)	0.074
**4 weeks**		
Adjusted for Hospital Only	-1.22(-4.78 to 2.37)	0.50
Adjusted for Surge type Only	-0.91(-5.07 to 3.25)	0.66
Adjusted for Surgeon Only	-2.10(-7.04 to 2.83)	0.40
**6 weeks**		
Adjusted for Hospital Only	10.93(-4.26 to 26.13)	0.16
Adjusted for Surge type Only	12.40(-4.16 to 28.96)	0.14
Adjusted for Surgeon Only	18.88(0.94 to 36.83)	0.040
**30 Second Chair to Stand test**		
**Baseline**		
Adjusted for Hospital Only	-1.53(-3.64 to 0.59)	0.15
Adjusted for Surge type Only	-1.86(-4.09 to 0.376)	0.10
Adjusted for Surgeon Only	-2.39(-5.05 to 0.27)	0.077
**4 weeks**		
Adjusted for Hospital Only	-1.78(-4.83 to 1.27)	0.25
Adjusted for Surge type Only	-1.68(-4.64 to 1.28)	0.26
Adjusted for Surgeon Only	-1.86(-5.47 to 1.75)	0.31
**6 weeks**		
Adjusted for Hospital Only	-0.47(-3.96 to 3.01)	0.79
Adjusted for Surge type Only	-0.37(-3.72 to 2.97)	0.82
Adjusted for Surgeon Only	-0.95(-5.03 to 3.13)	0.64
**EPIC-26 Score**		
**Baseline**		
Adjusted for Hospital Only	8.28(-4.31 to 20.86)	0.19
Adjusted for Surgeon Only	16.14(-2.04 to 34.32)	0.079
**4 weeks**		
Adjusted for Hospital Only	4.11(-3.37 to 11.58)	0.27
Adjusted for Surgeon Only	8.12(-3.12 to 19.37)	0.15
**6 weeks**		
Adjusted for Hospital Only	7.92(-2.97 to 18.81)	0.15
Adjusted for Surgeon Only	14.09(-0.19 to 28.37)	0.053
**NASA-TXL Score**		
**Surgeon**		
Adjusted for Hospital Only	-20.77(-33.38 to -8.15)	0.0018
Adjusted for Surge type Only	-20.32(-32.61 to -8.03)	0.0017
Adjusted for Surgeon Only	-18.03(-32.23 to -3.82)	0.014
**First Assistant**		
Adjusted for Hospital Only	-3.32(-14.81 to 8.17)	0.56
Adjusted for Surge type Only	-1.48(-13.98 to 11.03)	0.81
Adjusted for Surgeon Only	-1.02(-15.57 to 13.53)	0.89

Instrument nurse		
Adjusted for Hospital Only	-2.72(-14.35 to 8.90)	0.64
Adjusted for Surge type Only	-0.64(-12.89 to 11.60)	0.92
Adjusted for Surgeon Only	-4.40(-17.24 to 8.45)	0.49

a.Mixed-effects logistic model with Firth's correction was used in the sensitive analysis for positive surgical margin, and the surgeon, hospital or surgery type were as the fixed effects.

Table T. List of study team responsibility

Group	Main Member*	Responsibility
Surgeon group	Xu Zhang，Hongzhao Li，Xin Ma，Weijun Fu，Baojun Wang，Qingbo Huang，Wanhai Xu，Shuo Wang，Xia Dan，Chaochao Liang，Sheng Tai，Mulati Rexiati	Responsible for preoperative consultation and surgical implementation.
Telesurgery procedure planning team	Ye Wang，Yong Song, Guoqiang Yang, Lu Tang，Yanzhong Liu, Bingzhang Qiao，Yubai Zhang，Tao Xu，Sunyi Ye，Sheng Tai，Wuyi Zhao，Xu Li，Xue Dong	Patient screening, surgical scheduling, and time coordination.
Technical Group for Telesurgery	Ye Wang，Jianchen Wang，Yuanqian Gao，Xiangping Zhang，Wuyi Zhao，Chengjun Jiang，Xiaodong Zhou，Sheng Cheng	Responsible for system maintenance and monitoring
Follow-up Group	Guojun Liu，Xuyuan Xiang，Xiaoshan Hu	Manage participant follow-up, scale survey, and clinical information collection by independent research nurses.

Table U. List of Investigators-Nonauthor Collaborators

Name	Institution	Role or Contribution
Xiaoshang Hu	Chinese PLA General Hospital	Research Nurse
Lifeng Zhang	Chinese PLA General Hospital	Research Nurse
Qiang Zhu	Chinese PLA General Hospital	Medical staff involved in patient care
Lu Tang	Chinese PLA General Hospital	Medical staff involved in patient care
Run Zhu	Chinese PLA General Hospital	Medical staff involved in patient care
Yuzhu Li	Chinese PLA General Hospital	Medical staff involved in patient care
Songliang Du	Chinese PLA General Hospital	Medical staff involved in patient care
Zhenwei Lei	Chinese PLA General Hospital	Medical staff involved in patient care
Qiming Liu	Chinese PLA General Hospital	Medical staff involved in patient care
Gang Guo	Chinese PLA General Hospital	Medical staff involved in patient care
Hongzhi Wang	The First Affiliated Hospital of Anhui Medical University	Medical staff involved in patient care
Hanjiang Xu	The First Affiliated Hospital of Anhui Medical University	Medical staff involved in patient care
Meng Zhang	The First Affiliated Hospital of Anhui Medical University	Medical staff involved in patient care
Jiabing Yue	The First Affiliated Hospital of Anhui Medical University	Medical staff involved in patient care
Bensheng Liang	The First Affiliated Hospital of Anhui Medical University	Research Nurse
Hui Zhang	The First Affiliated Hospital of Anhui Medical University	Engineer
Ziqi Wang	Harbin Medical University Cancer Hospital	Medical staff involved in patient care

Tao Xu	Harbin Medical University Cancer Hospital	Medical staff involved in patient care
Jianzhang Li	Harbin Medical University Cancer Hospital	Medical staff involved in patient care
Dexin Ding	Harbin Medical University Cancer Hospital	Medical staff involved in patient care
Huirui Zhang	Harbin Medical University Cancer Hospital	Medical staff involved in patient care
Pengyu Guo	Harbin Medical University Cancer Hospital	Medical staff involved in patient care
Zhongjie Qiao	Harbin Medical University Cancer Hospital	Medical staff involved in patient care
**Name**	**Institution**	**Role or Contribution**
Hongshuang Dai	Harbin Medical University Cancer Hospital	Medical staff involved in patient care
Siwen Yu	Harbin Medical University Cancer Hospital	Medical staff involved in patient care
Donghui Ye	The First Affiliated Hospital, School of Medicine, Zhejiang University	Medical staff involved in patient care
Angbang He	The First Affiliated Hospital, School of Medicine, Zhejiang University	Medical staff involved in patient care
Xiaolin Yao	The First Affiliated Hospital, School of Medicine, Zhejiang University	Medical staff involved in patient care
Mingna Chen	The First Affiliated Hospital, School of Medicine, Zhejiang University	Medical staff involved in patient care
Lifen Chen	The First Affiliated Hospital, School of Medicine, Zhejiang University	Medical staff involved in patient care
Abudureheman Zebibula	The First Affiliated Hospital of Xinjiang Medical University	Medical staff involved in patient care
Fang Chen	The First Affiliated Hospital of Xinjiang Medical University	Medical staff involved in patient care
Zhiqiang Zhao	The First Affiliated Hospital of Xinjiang Medical University	Medical staff involved in patient care
Zhenbin Zhang	The First Affiliated Hospital of Xinjiang Medical University	Medical staff involved in patient care
Jiangjiawei Feng	The First Affiliated Hospital of Xinjiang Medical University	Medical staff involved in patient care
Jingyan Liang	The First Affiliated Hospital of Xinjiang Medical University	Medical staff involved in patient care
Xiaodong Zhou	Shenzhen Edge Medical Co., Ltd.	Engineer
Jianle Mao	Shenzhen Edge Medical Co., Ltd.	Engineer
Sheng Chen	Shenzhen Edge Medical Co., Ltd.	Engineer
Chengjun Jiang	Shenzhen Edge Medical Co., Ltd.	Engineer

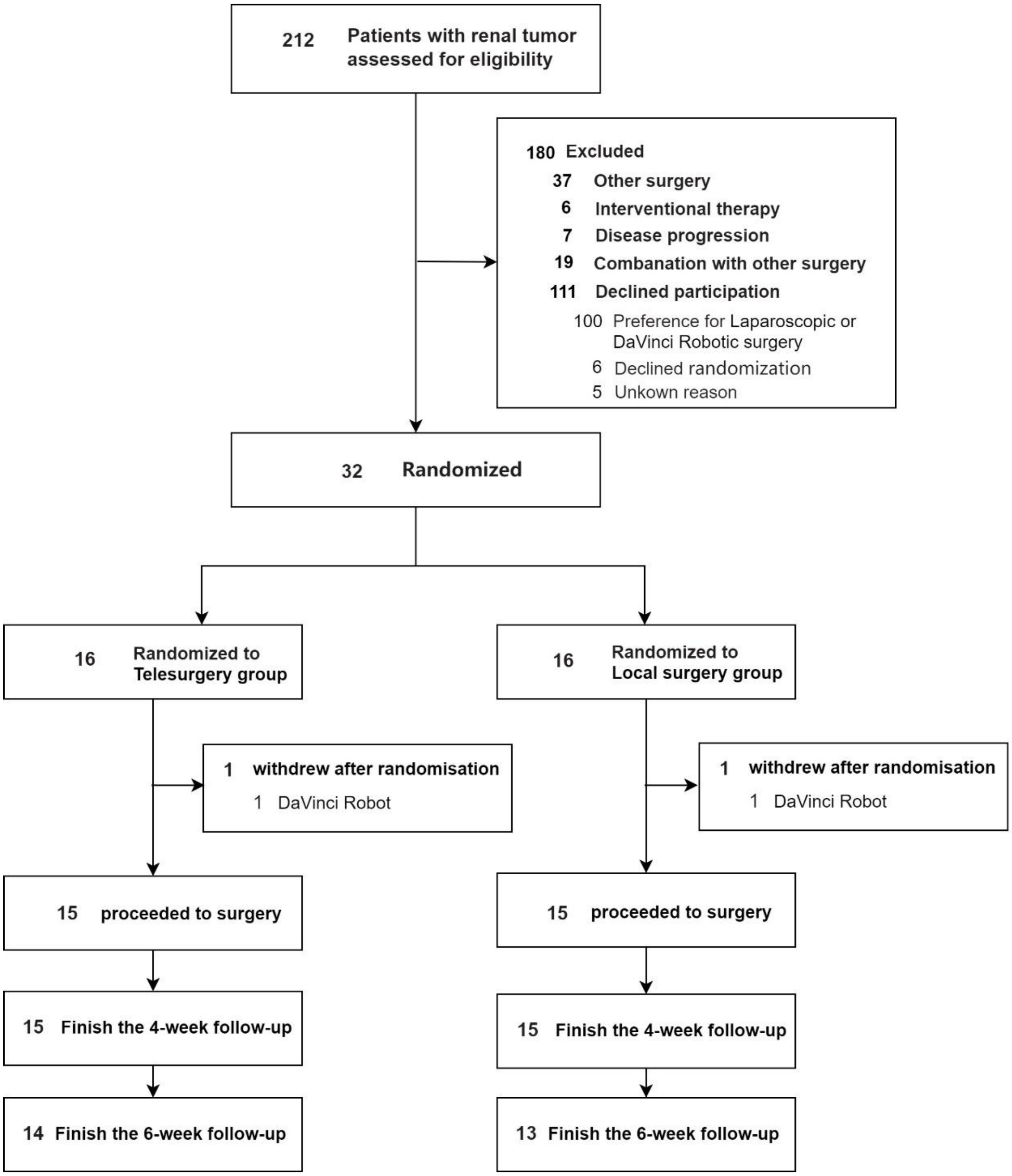

Figure A. Participant Flow of participants received partial nephrectomy in this trial.

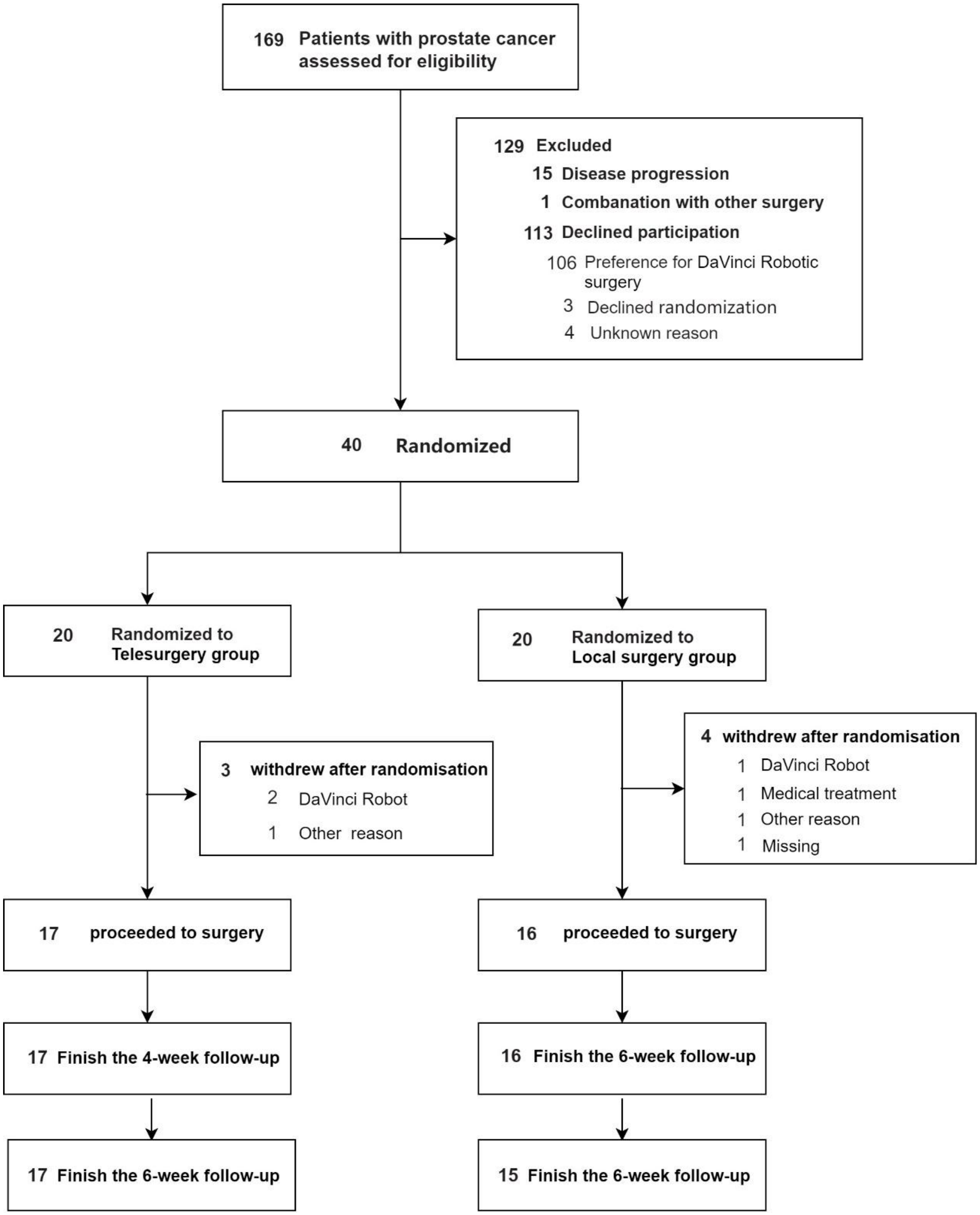

Figure B. Participant Flow of participants received prostatectomy in this trial. One patient randomized to local surgery group was missing after the randomisation.

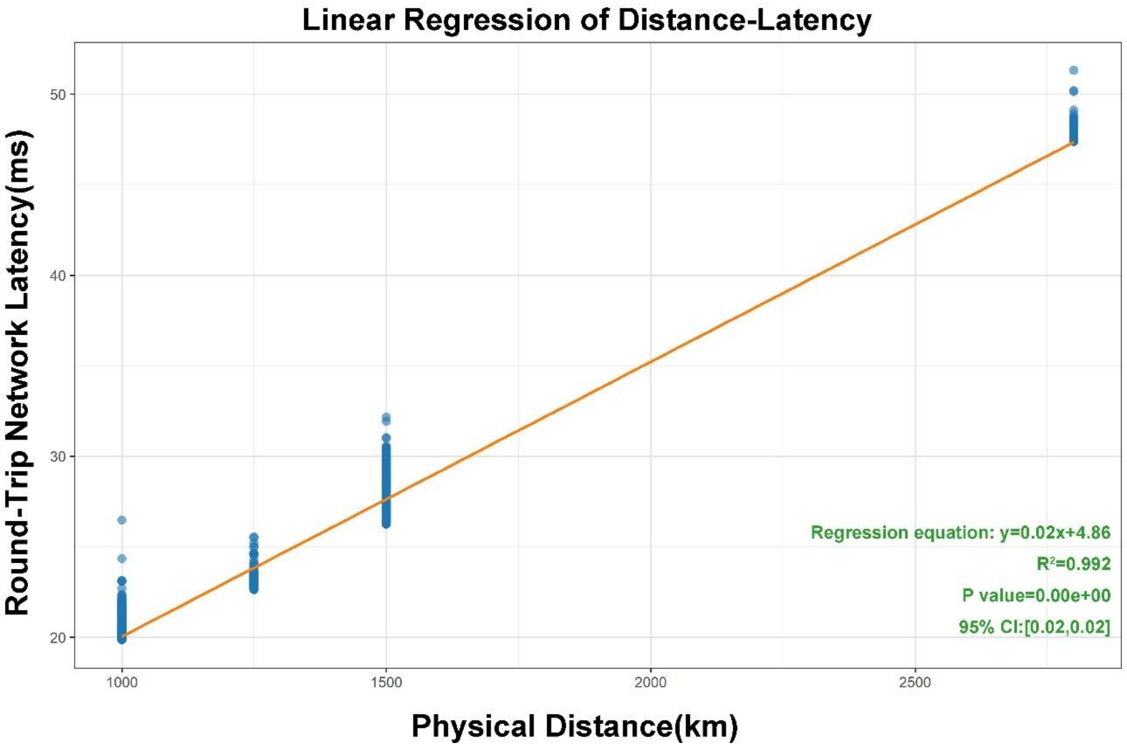

Figure C. Univariate linear regression of physical distance and round-trip network latency. This univariate linear regression equation can be used to approximately calculate the mean round-trip network latency according to the physical distance.

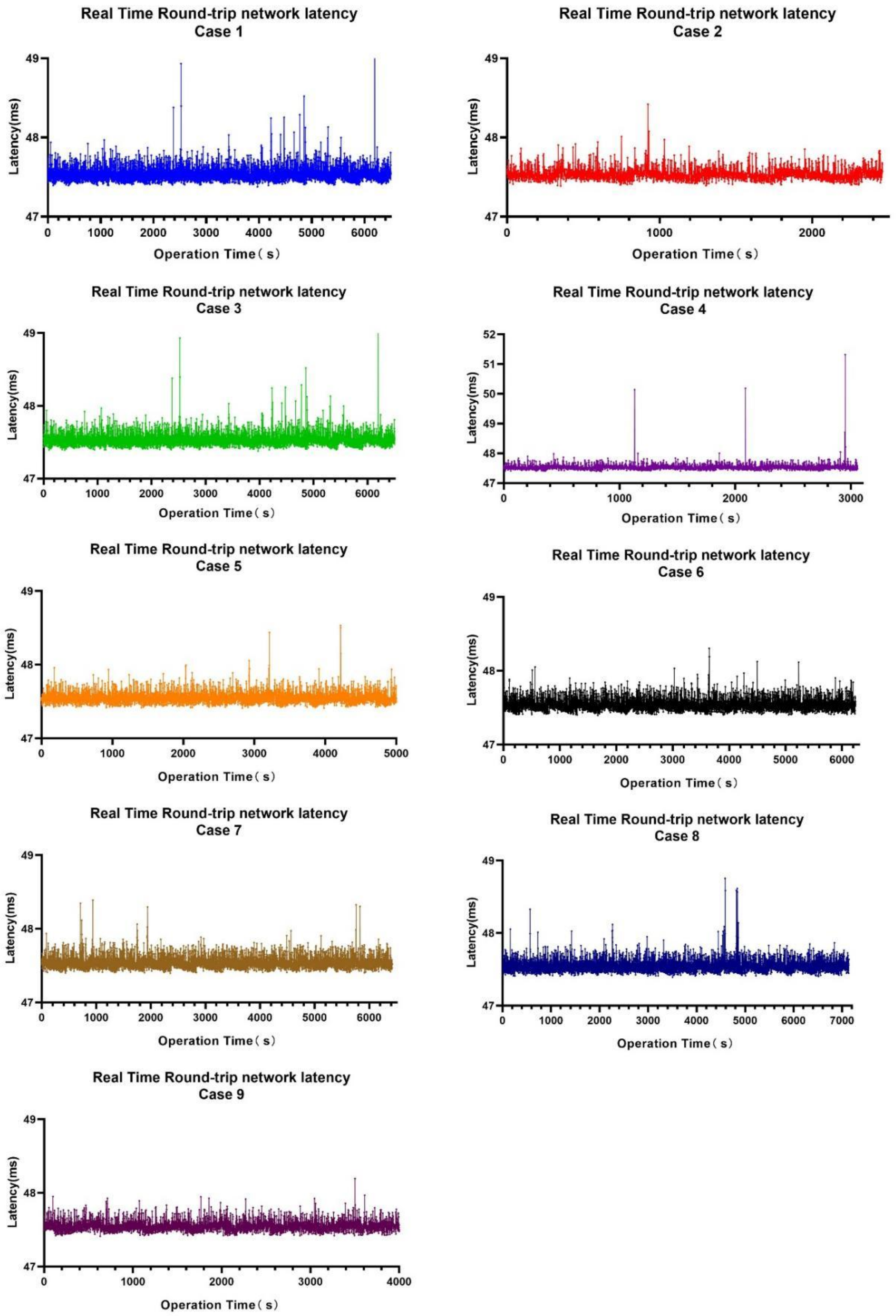

Figure D. Real-time round trip network latency from Beijing to Urumqi.

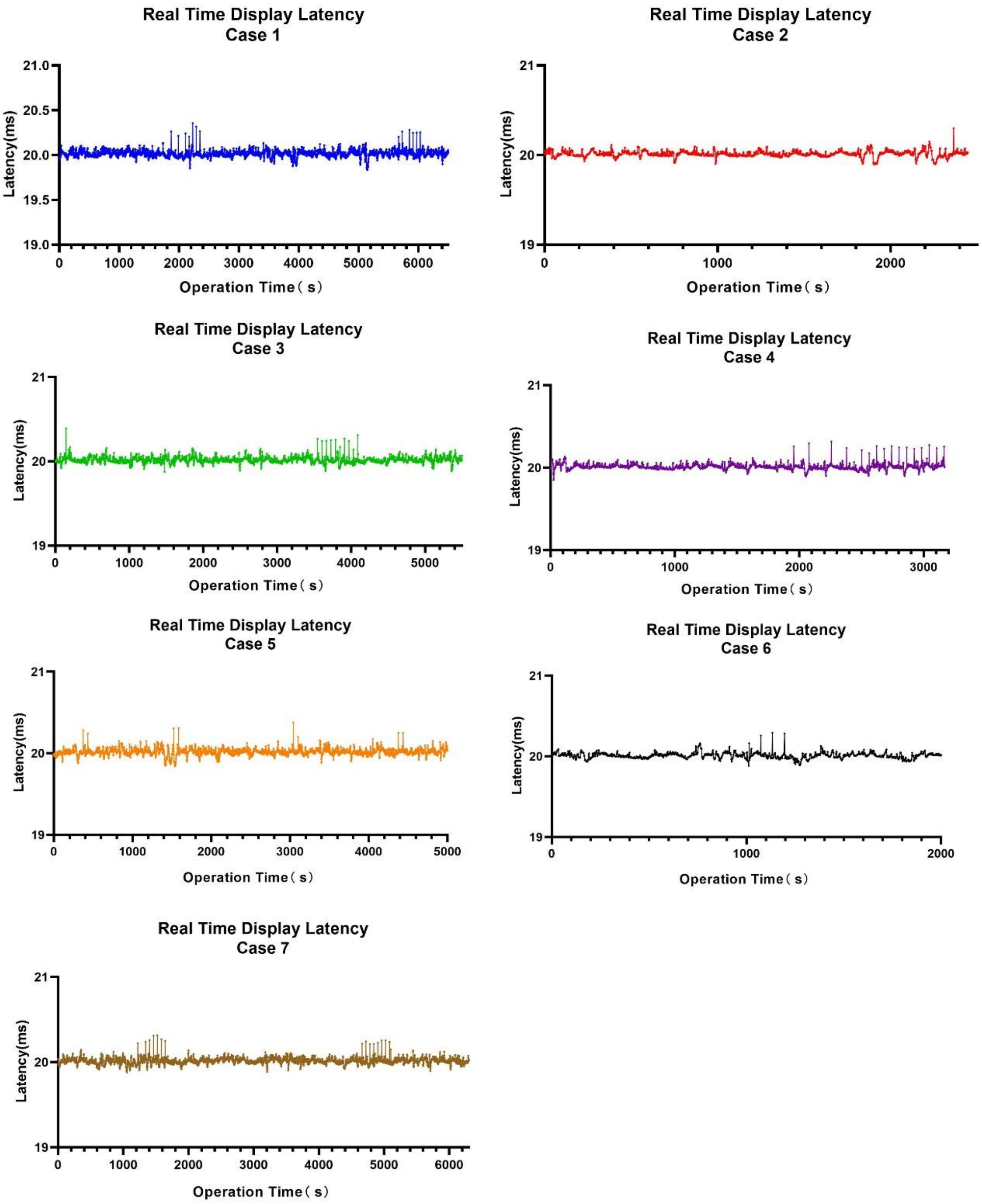

Figure E. Real-time display latency from Beijing to Urumqi. The real-time display latency of Case 8 and Case 9 were missing as incorrect settings of the recording system.

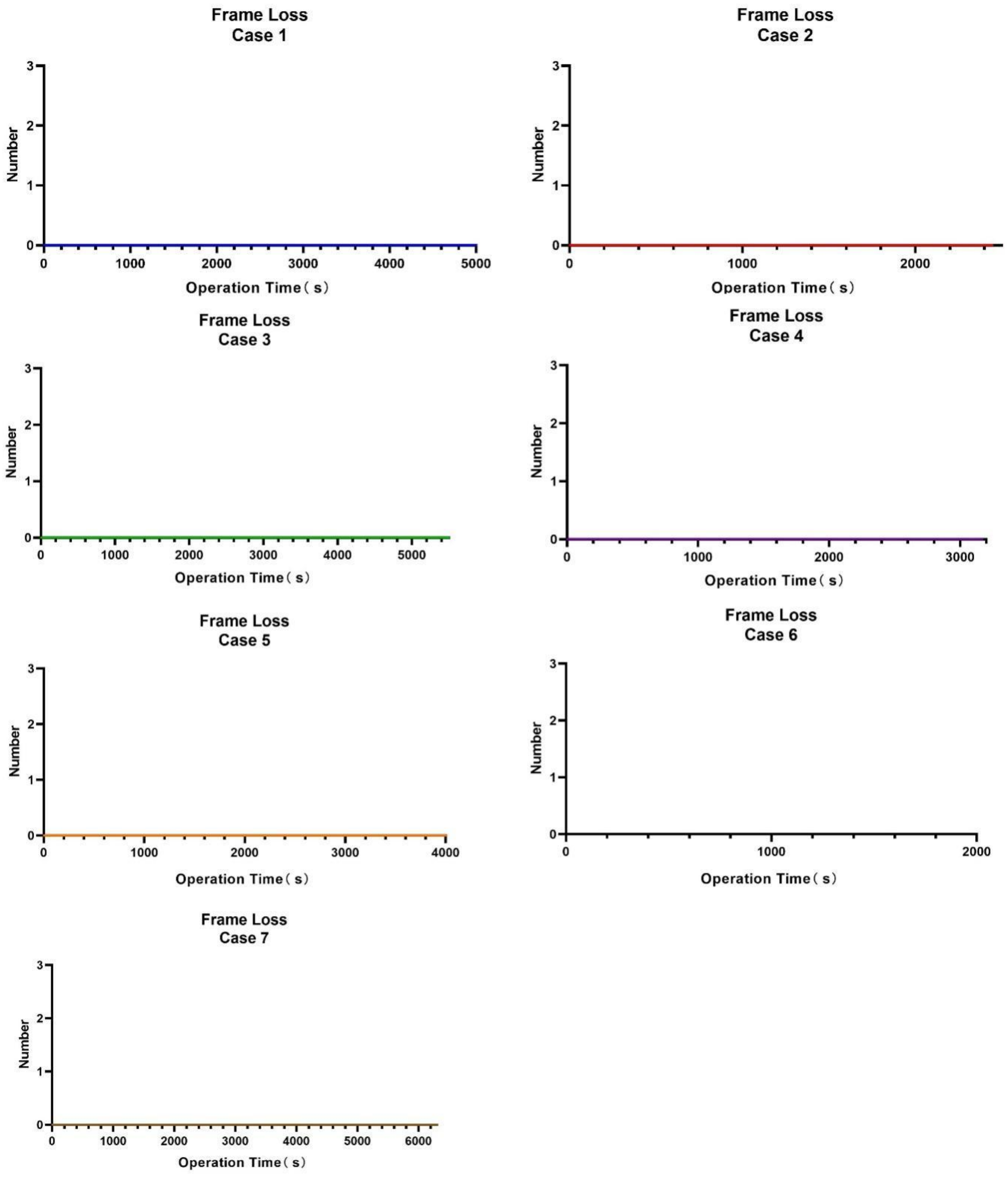

Figure F. Real-time frame loss during the surgery from Beijing to Urumqi. The frame loss of Case 8 and Case 9 were missing as incorrect settings of the recording system.

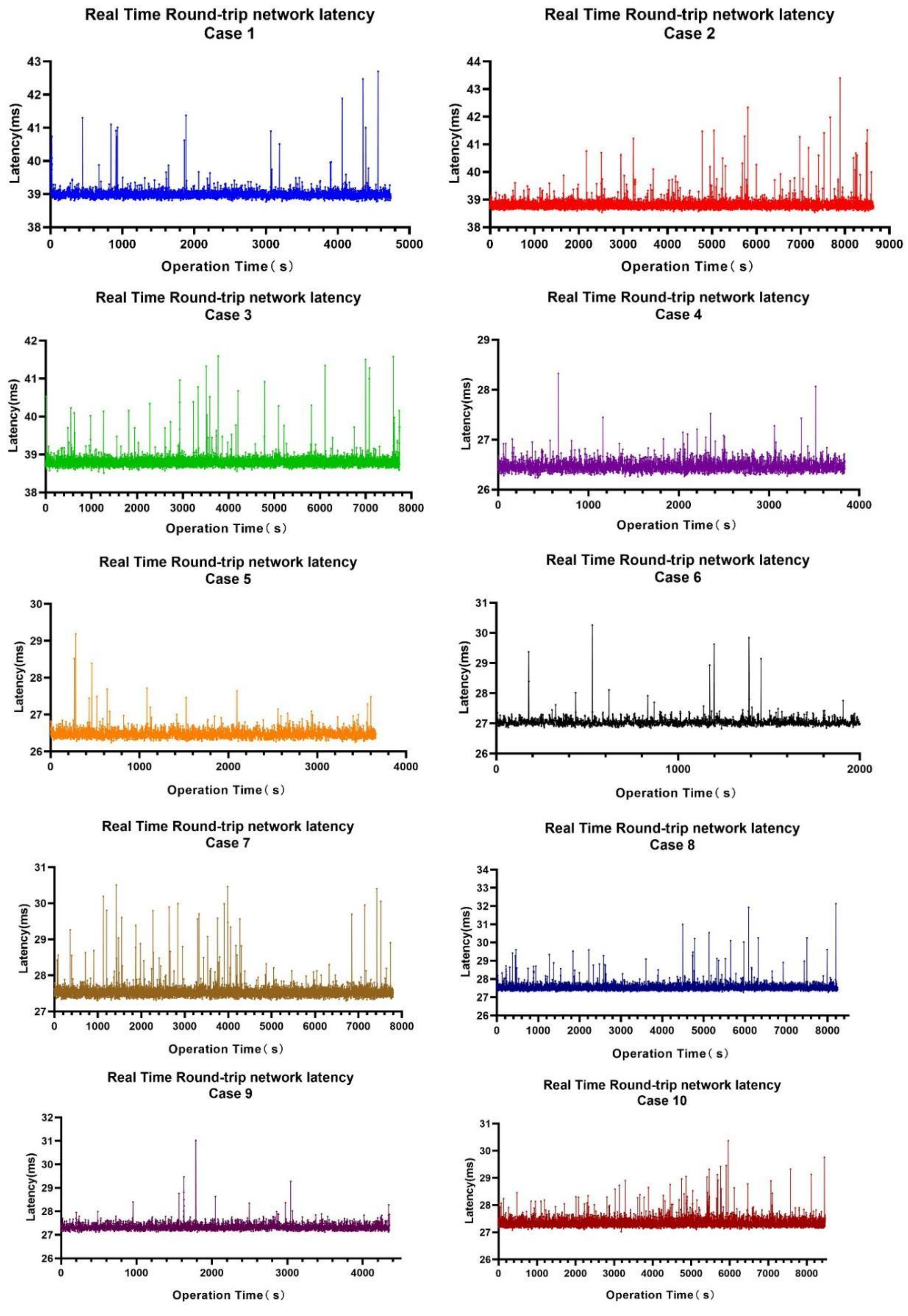

Figure G. Real-time round trip network latency from Beijing to Hangzhou.

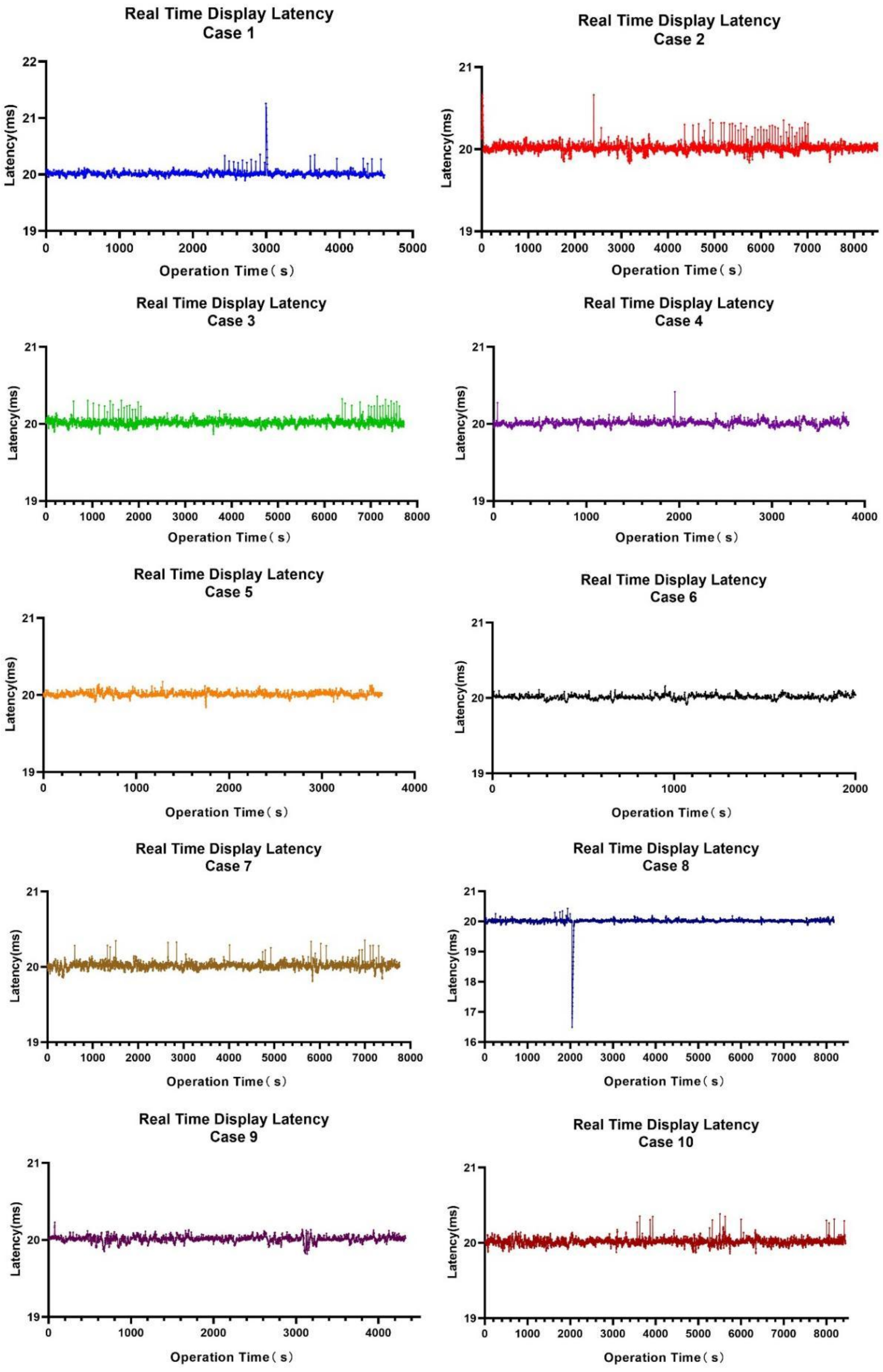

Figure H. Real-time display latency from Beijing to Hangzhou.

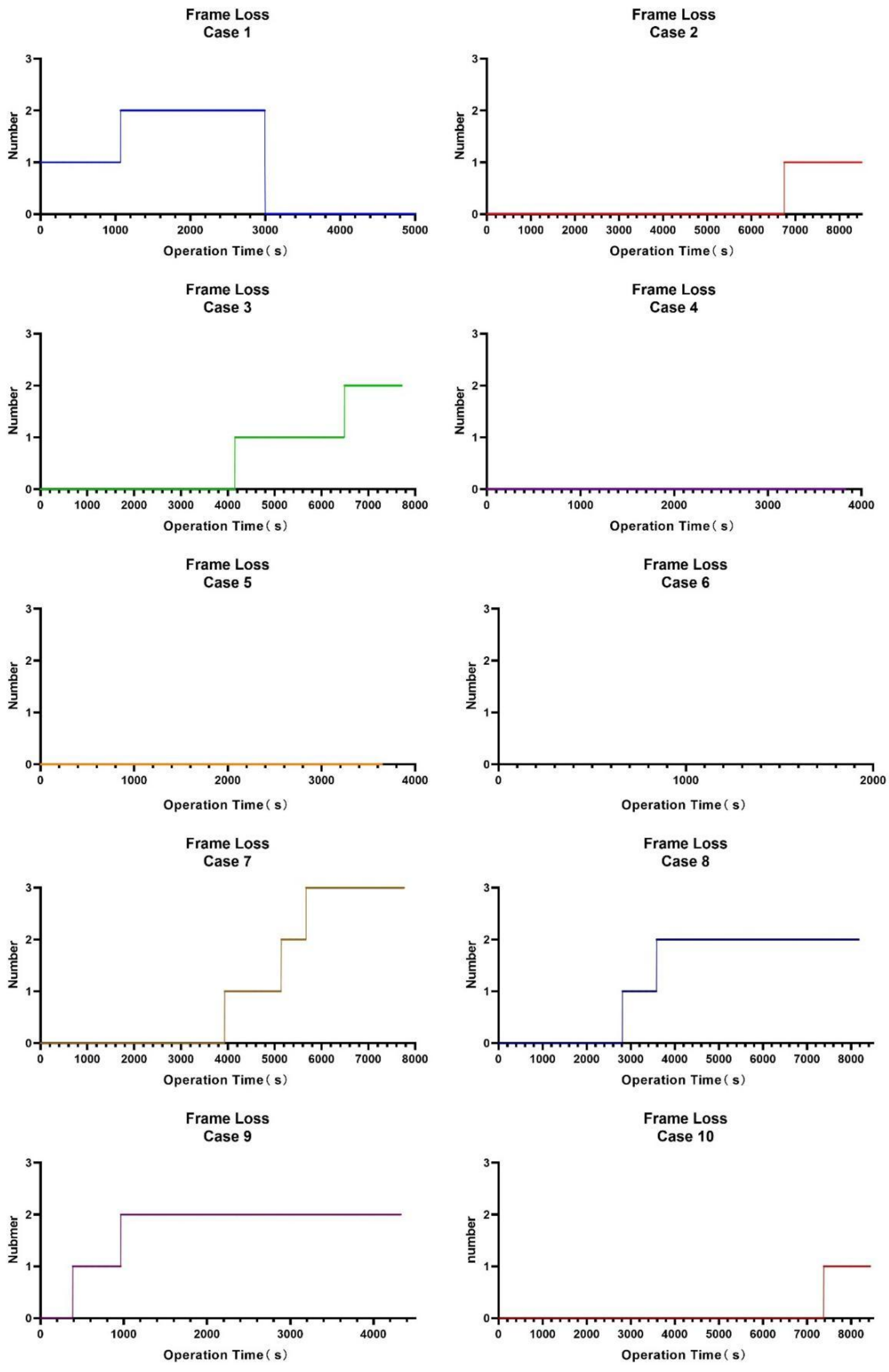

Figure I. Real-time frame loss during the telesurgery from Beijing to Hangzhou.

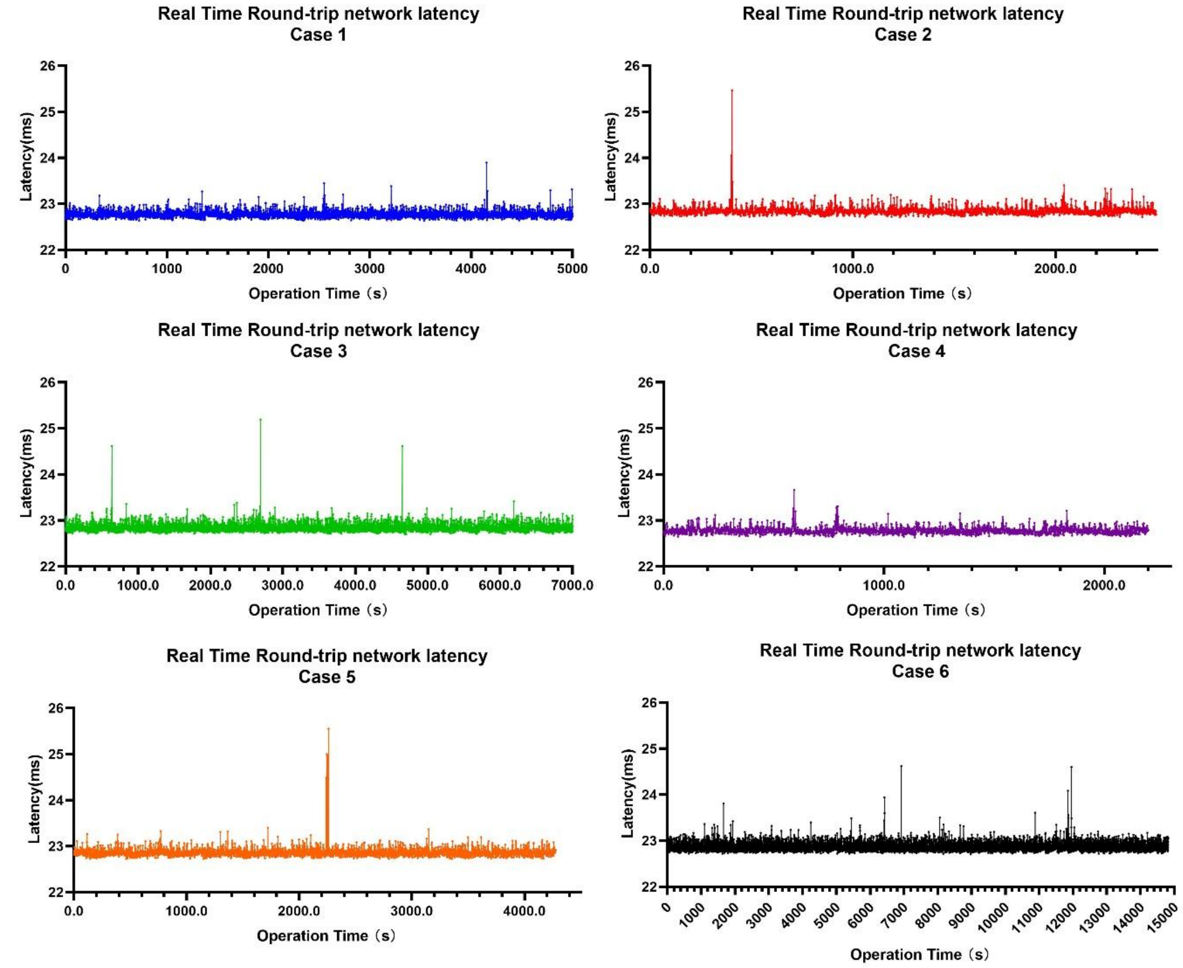

Figure J. Real-time round trip network latency from Beijing to Harbin.

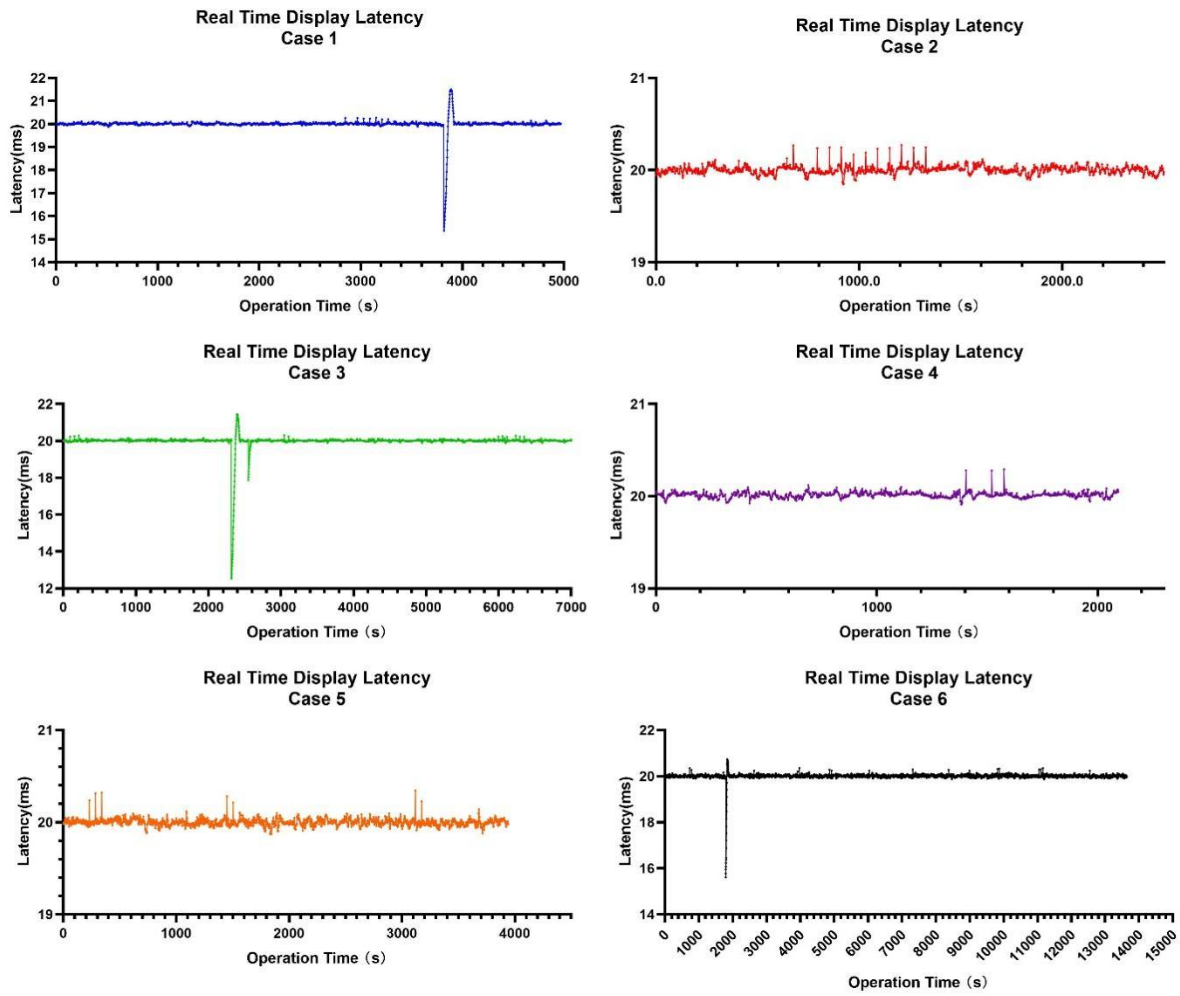

Figure K. Real-time display latency from Beijing to Harbin.

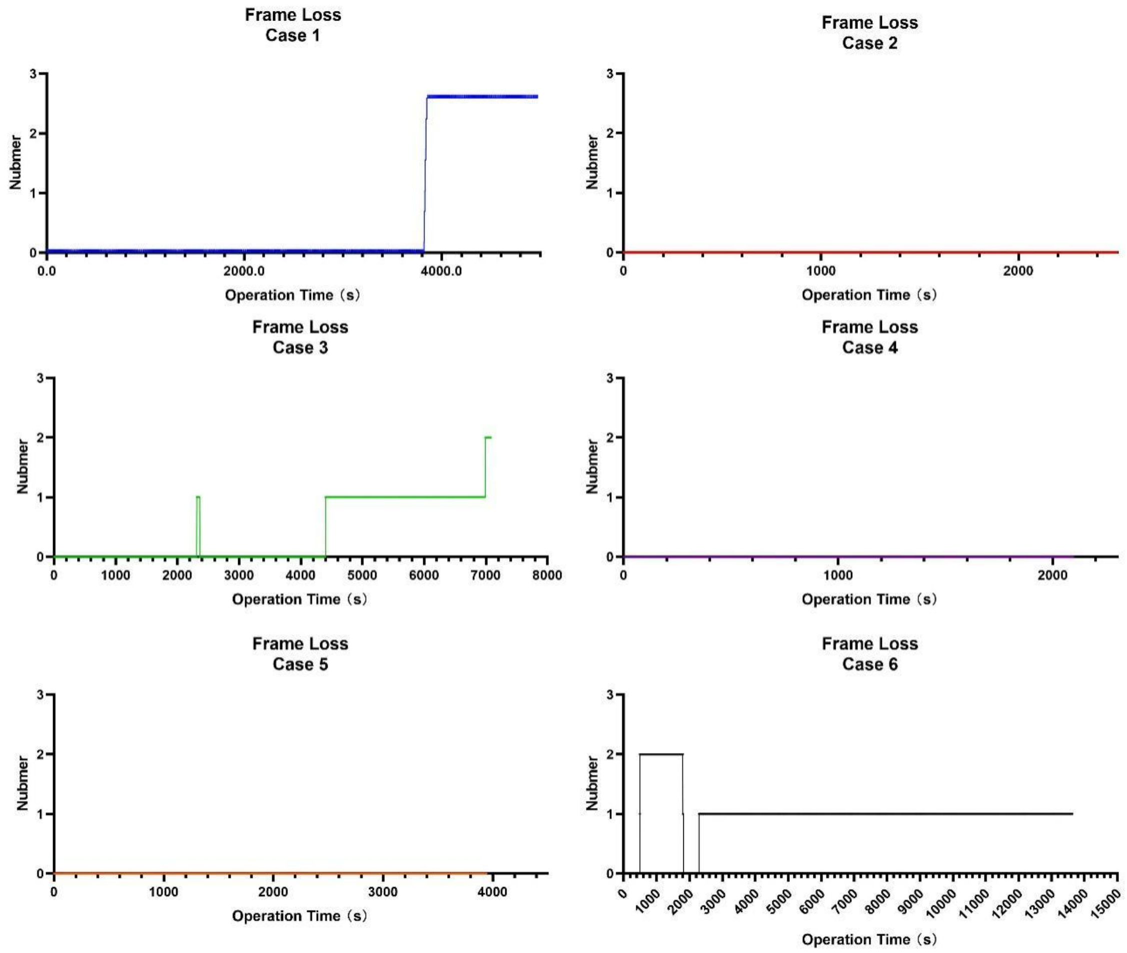

Figure L. Real-time frame loss during the telesurgery from Beijing to Harbin.

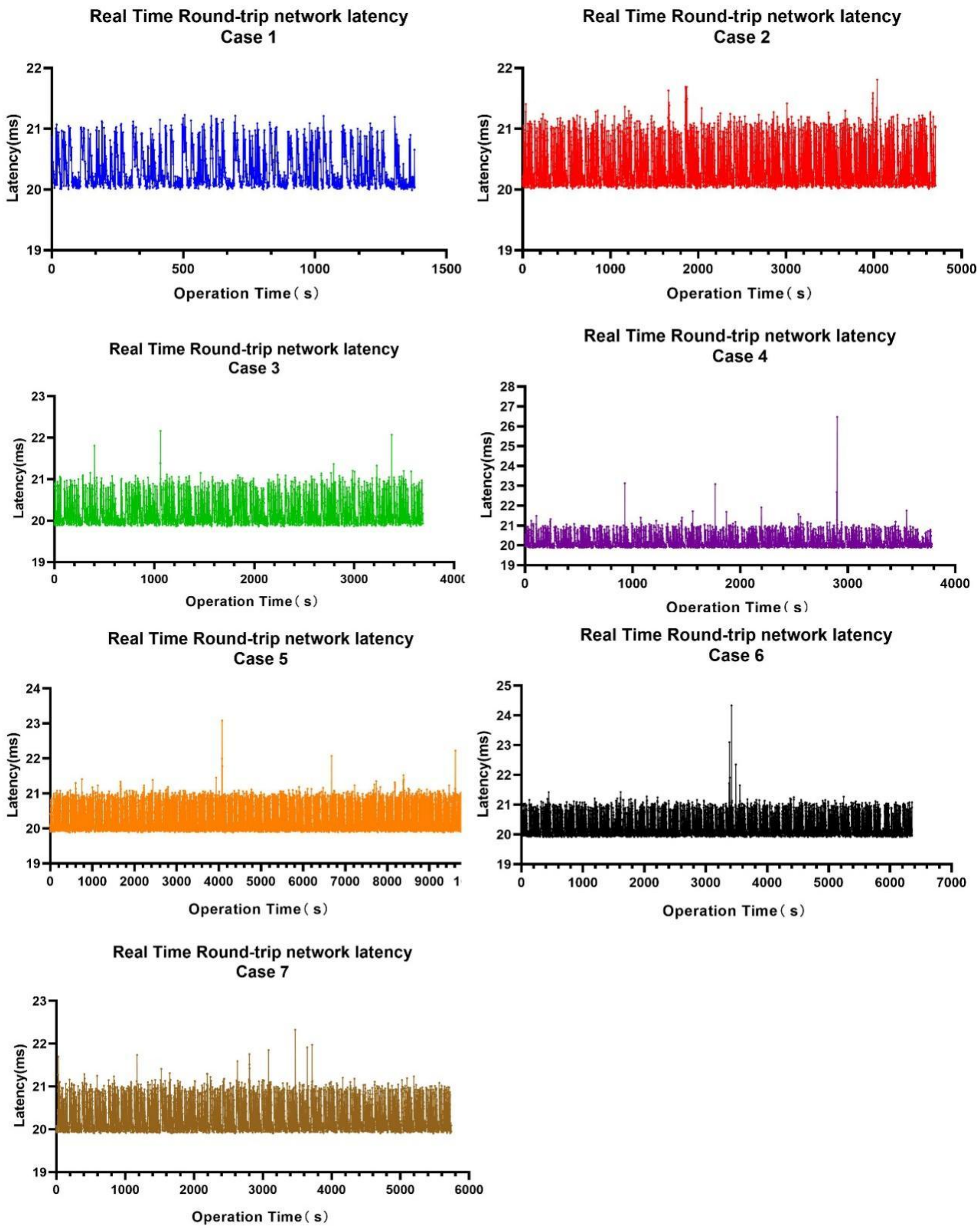

Figure M. Real-time round trip network latency from Beijing to Hefei.

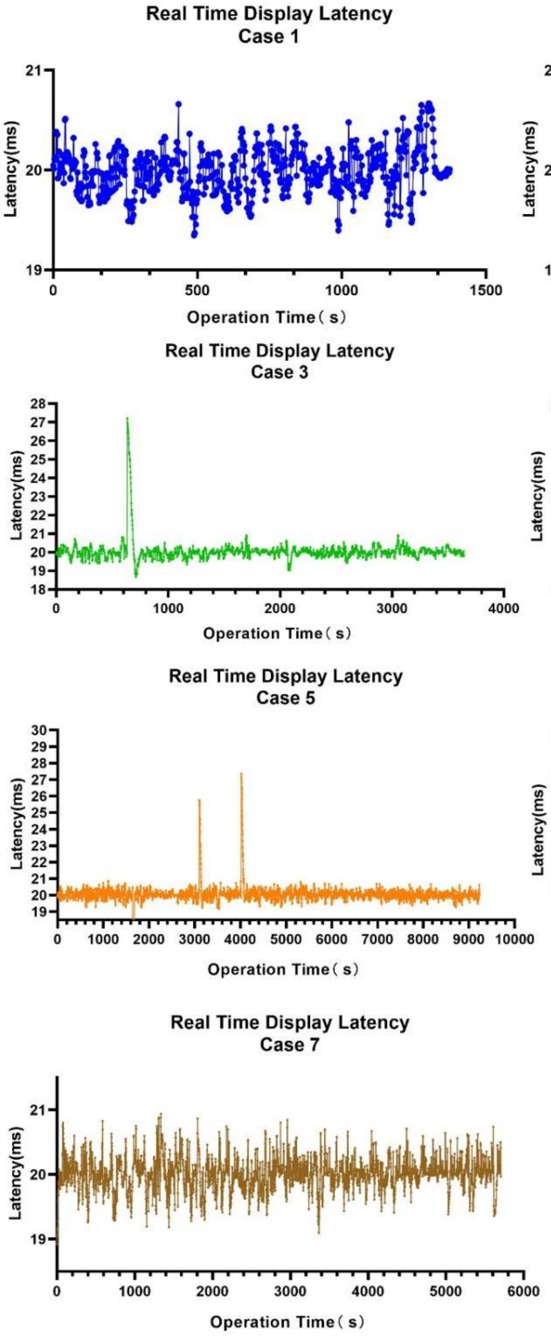

Figure N. Real-time display latency from Beijing to Hefei.

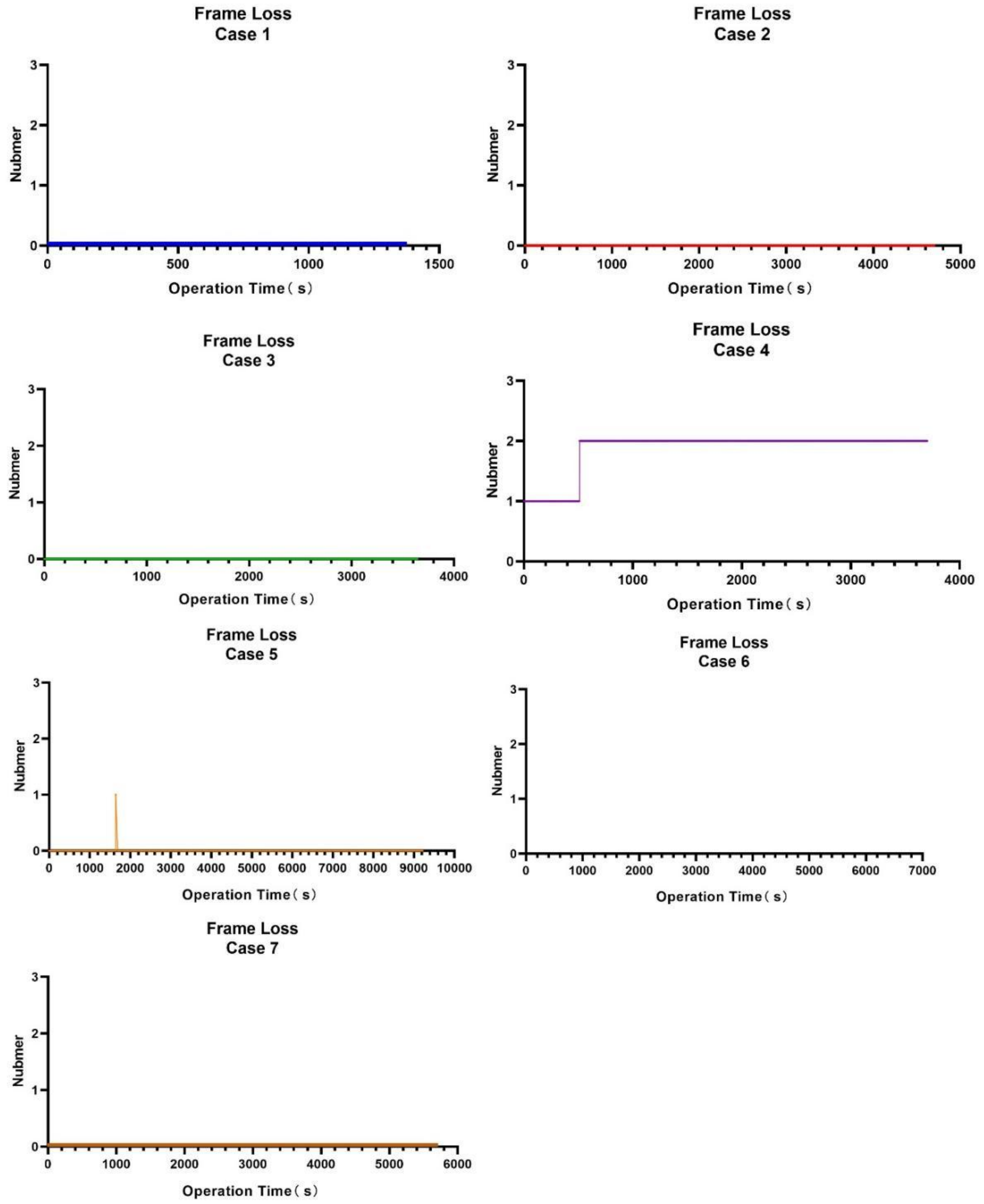

Figure O. Real-time frame loss during the telesurgery from Beijing to Hefei.
