# Supplementary material for: Reliability of urological telesurgery compared with local surgery: multicentre randomised controlled trial
Source: BMJ. 2026 Jan 28;392:e083588. doi: 10.1136/bmj-2024-083588 (PMC12849050; doi:10.1136/bmj-2024-083588)
Supplement: Supplementary file 2 — Web appendix: Trial protocol and statistical analysis plan [file wany083588.ww2.pdf]

CLINICAL STUDY PROTOCOL

**STUDY TITLE:**	A Prospective, Multicenter, Single blind, Randomized Controlled Trial on the safety and reliability of telesurgery robotic system on urology
**PROTOCOL NUMBER:**	JF-MP-IITP-007
**Chinese Clinical Trail (ChiCTR) register number：**	ChiCTR2300077721
**SPONSOR & LEADING CENTER:**	Xu Zhang, MD, Ph D.<br>Department of Urology，Chinese PLA General Hospital, Beijing, China
**PRINCIPAL INVESTIGATOR:**	Xu Zhang, MD, Ph D., Department of Urology，Chinese PLA General Hospital, Beijing, China; 28 Fuxing Road, Beijing, China, 100853<br>Tel: 86-10-66938211.
**PARITICPATING CENTERS:**	First Affiliated Hospital, Xinjiang Medical University，Urumqi, China;<br>Affiliated Cancer Hospital，Harbin Medical University，Harbin，China;<br>First Affiliated Hospital, Medical College of Zhejiang University，Hangzhou，China;<br>First Affiliated Hospital，Anhui Medical University, Hefei, China；<br>Hainan Provincial People's Hospital，Haikou, China.
**PROTOCOL VERSION/DATE:**	Version 2.0, December 8th, 2023

Protocol Signature Page

Title: A Prospective, Multicenter, Single blind, Randomized Controlled Trial on the safety and reliability of telesurgery robotic system on urology

PROTOCOL VERSION/DATE: Version 2.0, December 8th, 2023

Signed statement

1. I will strictly abide by the Declaration of Helsinki, the Quality Management Practice for Clinical Trials of Medical Devices, and relevant domestic laws and regulations to ensure the life, health, personal privacy, and dignity of test subjects.
2. I promise to abide by the code of academic ethics and ensure the authenticity, integrity, and traceability of research materials and data.
3. I ensure that there is no conflict of economic or non-economic interest between myself, the project team members, and the research, other than the normal research expenses.
4. I promise that I will not harm the interests of the subjects, and I will ensure that adequate medical services are provided to the subjects who have adverse events or serious adverse events during their participation in the study.
5. I promise to protect the privacy of subjects during or after the study, in accordance with the privacy and confidentiality provisions of relevant laws and regulations.

6. I promise to follow the signed subject consent procedure for submitting an ethical review; fully inform subjects of the risks, benefits, and alternative treatment methods of the study; obtain informed consent from subjects/legal representatives; and protect the interests of vulnerable groups.

7. I undertake to submit serious adverse event reports, protocol deviations, research progress reports, and concluding reports to the Ethics Committee in a timely manner.

8. I will provide a copy of the trial protocol and all relevant information to anyone who participates in the study under my leadership. I will discuss with them the content of the relevant materials for the clinical trial project to ensure that they fully understand and strictly implement the trial protocol.

Principal investigator:

Signature: ....

Date: ....

Protocol Amendment Form

**Protocol Title:** A Prospective, Multicenter, Single blind, Randomized Controlled Trial on the safety and reliability of telesurgery robotic system on urology	
**Protocol Number:** JF-MP-IITP-007	**Version Number:** 2.0
**Protocol Amendment Approved By:** Xu Zhang, M.D. & Ph.D.	**Amendment Date**: December 8th, 2023

Summary of Changes in Amendment 1

Number	Section Affected	Content before modification	Modified content
1	Title page	Version number: V1.0<br>Date: September 25th, 2023<br>Clinical trial institution: Chinese PLA General Hospital;<br>Affiliated Cancer Hospital,Harbin Medical University;<br>Hainan Provincial People’s Hospital.<br>Principal investigators: Xu Zhang, Wanhai Xu, Xinli Kang	Version number: V2.0<br>Date: December 8th, 2023<br>Clinical trial institution: Chinese PLA General Hospital;<br>First Affiliated Hospital, Xinjiang Medical University;<br>Affiliated Cancer Hospital,Harbin Medical University;<br>First Affiliated Hospital, Medical College of Zhejiang University;<br>First Affiliated Hospital, Anhui Medical University;<br>Hainan Provincial People’s Hospital.<br>Principal

			investigators:Xu Zhang, Wanhai Xu, Xinli Kang, Dan Xia, Chaochao Liang, Mulati Rexiati
2	Protocol Signature Page	Version number: V1.0 Date: September 25th, 2023	Version number: V2.0 Date: December 8th, 2023
3	3.1 Overall Design	The participants will be recruited from the Chinese PLA General Hospital, Harbin Medical University Affiliated Cancer Hospital, and Hainan Provincial People’s Hospital.	The participants will be recruited from the Chinese PLA General Hospital, Harbin Medical University Affiliated Tumor Hospital, the First Affiliated Hospital of Zhejiang University School of Medicine, the First Affiliated Hospital of Anhui Medical University, the First Affiliated Hospital of Xinjiang Medical University, and Hainan Provincial People’s Hospital.
4	3.4.4 Operation process of the telesurgery	In the telesurgery group, the first surgeon activates the main console and the teleconference subsystem on the surgeon's side, and the patient's side activated the teleconference system, surgical cart and the backup console of the surgery robot. Telesurgery technical teams in Beijing and Harbin/Haikou are responsible for the	In the telesurgery group, the first surgeon activates the main console and the teleconference subsystem on the surgeon's side, and the patient's side activated the teleconference system, surgical cart and the backup console of the surgery robot. Telesurgery technical teams in Beijing and Urumqi/Harbin/Hangzhou/Hefei/Haikou are responsible for the

		network connection and equipment debugging between the doctor's console and the patient's surgical platform. After the the correct connection and conditions of all the subsystem of the telesurgery, all the display screens will be turn off, and the patient will be taken to the operation room for the surgery. After the patient is anesthetized, all display screens will be turned on again.	network connection and equipment debugging between the doctor's console and the patient's surgical platform. After the the correct connection and conditions of all the subsystem of the telesurgery, all the display screens will be turn off, and the patient will be taken to the operation room for the surgery. After the patient is anesthetized, all display screens will be turned on again.
5	Header	Version number: V1.0<br>Date: September 25th, 2023	Version number: V2.0<br>Date: December 8th, 2023

TABLE OF CONTENTS

1. INTRODUCTION · · · 11

1.1 Development of Telesurgery · · · 11

1.2 Safety and reliability of Telesurgery system · · · 11

2. OUTCOMES · · · 13

2.1 Primary Outcome · · · 13

2.2 Secondary Outcomes · · · 13

2.2.1 Early recovery · · · 13

2.2.2 Perioperative morbidity · · · 14

2.2.3 Oncological outcomes · · · 15

2.2.4 Task load of medical team · · · 15

2.2.5 Telesurgery monitoring data · · · 15

3 STUDY DESIGN · · · 16

3.1 Overall Design · · · 16

3.2 Randomization · · · 17

3.3 Blinding and Unblinding · · · 18

3.4 Study Treatment · · · 18

3.4.1 Surgeon and unit accreditation · · · 18

3.4.2 Structure of Telesurgery system and protective measure · · · 20

3.4.3 Proctoring of surgery and dissemination of a standard technique · · · 20

3.4.4 Operation process of the telesurgery · · · · · · · · · · · · · · · 20

3.5 Standard Follow up for patients and medical team · · · · · · · · · · · 21

3.6 Trial Assessments · 21

3.7 End of Study · 24

4 STUDY SCHEDULE · 24

5 TABLE OF ASSESSMENT · 25

6 PATIENT SCREENING & CONSENT · · · · · · · · · · · · · · · · 26

6.1 Patient Screening · 26

6.2 Informed Consent · 27

6.3 Withdrawals · 28

7 ELIGIBILITY CRITERIA · 28

7.1 Inclusion Criteria · 29

7.2 Exclusion Criteria · 29

8 RECRUITMENT · 29

9 STATISTICAL METHODS · 30

9.1 Study Analysis · 30

9.2 Sample Size Determination · 30

9.3 Stopping Rules · 31

10 DATA COLLECTION AND HANDLING · · · · · · · · · · · · · · 32

10.1 Data Management · 33

10.2 Data Collection, Handling, and Verification · · · · · · · · · · · · · · 34

11 PEER AND REGULATORY REVIEW · · · · · · · · · · · · · · · · 34

12 ASSESSMENT AND MANAGEMENT OF RISK · · · · · · · · · · 35

12.1 Emergency plan for telesurgery · 35

12.2 Protection strategy of the telesurgery system · · · · · · · · · · · · · 35

13 RECORDING AND REPORTING OF EVENTS AND INCIDENTS · 36

13.1 Definitions of Adverse Events · 36

13.2 Procedures for Recording and Reporting Serious Adverse Events · 36

14 HUMAN SUBJECTS · 37

14.1 Ethical Considerations · 37

14.2 Communication with the Ethics Committee · · · · · · · · · · · · · 37

14.3 Informed Consent Form · 38

14.4 Subject Confidentiality · 39

15 INDEMNITY ARRANGEMENTS · · · · · · · · · · · · · · · · · · · 40

16 PUBLICATION AND DISSEMINATION POLICY · · · · · · · 40

17 FUNDING AND SUPPLY OF EQUIPMENT · · · · · · · · · · · 41

18 REFERENCES · 41

LIST OF ABBREVIATIONS

Abbreviation	Definition
CFDA	Chinese Food and Drug Administration
REC	Research Ethics committee
CRF	Case report form
ICF	Informed Consent Form
QoR-15 Score	Quality of recovery 15 items score
EPIC-26 Score	Expanded Prostate Cancer Index Composite-26
NASA	National Aeronautics and Space Administration
ICF	informed consent form
ISF	Investigator Site File
CT	Computer tomography
MR	magnetic resonance tomography
ICU	Intensive Care Unit

1. INTRODUCTION

1.1 Development of Telesurgery

Surgical technology has undergone significant advancements in recent years, progressing from its inception as open surgery to its large-scale implementation of minimally invasive endoscopic surgery and the present stage of minimally invasive endoscopic robot surgery, which is rapidly gaining popularity[1]. Surgical robots have been applied in clinics for more than 20 years, with widespread adoption and positive outcomes[2]. Compared to traditional endoscopic surgery, robotic endoscopic surgery has many advantages[3]. Rapid advances in surgical robotics and telecommunication technology have enabled real-time telesurgery, resulting in the direct control of surgical robotic systems by specialists thousands of kilometers away with virtually no operational latency[4, 5].

1.2 Safety and Reliability of the Telesurgery System

After rigorous and sufficient technical demonstrations and hundreds of animal tests, the safety, reliability and effectiveness of telesurgery have been preliminarily proved. With the assistance of the technical team of the domestic porous endoscopic surgery robot of China, led by the team of Academician Xu Zhang of the Chinese People's Liberation Army (PLA) General Hospital, the telesurgery operation network has been built, which covers most of China's territory. In Beijing and Sanya (3000km), an

explorative, small sample, single arm telesurgery clinical trial has been finished in 2023[6]. We performed telesurgical robot-assisted retrocaval ureteral repair and plastic surgery, radical nephrectomy, partial nephrectomy, radical prostatectomy, adrenal tumor resection, and other operations, all of which were successfully completed with a clear visual field, flexible operation of the robotic arm, good outcome, and no obvious signal latency feel. This preliminarily demonstrated the safety and reliability of robotic telesurgery in endoscopic urologic procedures. The present study on telesurgery systems aims to adopt a variety of technologies to ensure the smooth operation of telesurgery and that there is almost no movement latency during the operation, which can ensure the consistency of "hand-eye coordination" and the overall safety and effectiveness of telesurgery.

Telesurgery may mitigate the challenges faced by patients seeking medical treatment in different locations, rectify disparities in medical proficiency, alleviate the uneven distribution of medical resources during emergencies, and foster the provision of innovative medical and health services and the transparent exchange of medical resources[4, 7]. Reducing the disparity in medical resources across various regions, enhancing the capacity of primary medical and health services, and ensuring the equitable distribution of universal benefits are matters of utmost

importance.

2. OUTCOMES

2.1 Primary outcome:

The primary objective of this clinical trial will be to evaluate and validate the safety and efficacy of the telesurgery system in urologic surgery and compare it to local urologic surgery. The success was defined as the surgery being completed effectively as planned, without conversion to other surgical types and significant side injury or delays due to system malfunctions. Telesurgery was considered successful if it could be completed only using the telesurgery system without conversion to local surgery for any reason. The success or failure of each surgery will be jointly confirmed by the remote and local medical teams together.

2.2 Secondary outcomes:

The secondary outcomes of this study will be to investigate differences between telesurgery and local surgery in terms of complications and early recovery quality, physical functioning, early oncological outcomes, task load of the medical team, adverse events, and status or malfunction of the local surgery robotic or telesurgery system.

2.2.1 Early recovery

The following tools will be used to measure the early recovery:

1. Overall functional recovery: Quality of recovery 15-item score (QoR-

15 score) at baseline (preoperative), 4 weeks, and 6 weeks.

2. Overall functional recovery for prostate cancer patients: Expanded Prostate Cancer Index Composite-26 (EPIC-26 score) at baseline (preoperative), 4 weeks, and 6 weeks.

3. Physical activity: 30-second chair-to-stand test (number of times the patient can stand from sitting in a 30-second interval) at baseline (preoperative), 4 weeks, and 6 weeks.

2.2.2 Perioperative morbidity

To assess perioperative morbidity, the following clinical data will be collected:

1. Surgery-associated factors: intraoperative blood loss, operative time, and warm ischemia time (for partial nephrectomy).

2. Complications and adverse events: Adverse events will be recorded using the Clavien-Dindo classification at 4 weeks and 6 weeks postoperatively.

3. Postoperative hospitalization days.

4. Length of days in critical care.

5. Reoperational intervention.

6. Readmission to the hospital: Readmission to the hospital for any reason will be recorded, and the surgery-associated condition will be systemically reviewed.

7. Intra- and postoperative blood transfusion rates.

8. Mortality rate.

2.2.3 Oncological outcomes

To assess the oncological outcomes, the following pathological data will be collected:

1. Positive margin rate in the pathological specimen according to the pathology report from two blinded pathologists in each hospital.

2. Pathological type of the tumor;

3. The Gleason score and number of retrieved lymph nodes in the pathological specimen for prostatectomy with lymph node dissection only.

2.2.4 Task load of medical team

The NASA (National Aeronautics and Space Administration) Task Load Index (NASA-TLX) will be used to measure the task load of the first surgeon, first assistant, and instrument nurse immediately after the surgery in both groups.

2.2.5 Telesurgery monitoring data

To characterize the status of the telesurgery system, the following data will be collected:

1. Round-trip network latency (for telesurgery): The round-trip network latency is defined as the time latency of data packages between transmission and reception.

2. Display latency (for telesurgery): The display latency is defined as the time latency between coding and encoding the endoscope image.

3. Frame loss (for telesurgery): The frame loss is defined as the loss of data packages during data transmission.

4. Malfunction: The malfunction of the local surgical robot or telesurgery system will be recorded. The local surgical robot or telesurgery system is tested systemically one day preoperatively. A malfunction of the telesurgery system is defined as the system being out of control for any reason, such as a telecommunications pathway fault, a laboratory pathway fault, or a robotic fault. A malfunction of the local surgical robot is defined as the robot being out of control for any reason.

3 STUDY DESIGN

3.1 Overall Design

This prospective, multicenter, single-blinded, non-inferiority, randomized controlled trial will explore and compare the safety and reliability of telesurgery and local surgery for urologic procedures. The primary and secondary outcomes will be measured or recorded in the 6 weeks postoperatively.

Patients who have prostate cancer and a renal tumor, including renal cancer and a benign renal tumor, and who will undergone partial nephrectomy will be screened and asked to participate in this trial. The

participants will be randomized 1:1 to the telesurgery group or local surgery group. The follow-up time for the participants will be 6 weeks. The participants will be recruited from Chinese PLA General Hospital, Harbin Medical University Affiliated Cancer Hospital, the First Affiliated Hospital of Zhejiang University School of Medicine, the First Affiliated Hospital of Anhui Medical University, and the First Affiliated Hospital of Xinjiang Medical University. All participants will sign the informed consent form (ICF) and be screened for eligibility. Their basic information will be collected for clinical research.

3.2 Randomization

This study will adopt a random number distribution system. The random grouping table of subjects will be generated by independent statisticians (who will not participate in the clinical trial) from random coding units. The ratio of the test group to the control group will be 1:1. The seed number for generating the random table is fixed, and the process of generating the random table can be repeated. The generated random table of subjects will be imported into the random system by the random system administrator. All random numbers will not be used twice. After the successful screening of subjects, the random responsible person of each center will log in to the system and enter the basic information of subjects according to the system prompts, including the date of birth and

the name abbreviation, etc. The system will generate a unique random number for each subject and randomly allocate subjects to the groups.

3.3 Blinding and Unblinding

The patients will be blinded. Due to the significant differences in appearance between the experimental and control devices, blinding the researchers will not be possible. Blinding of the patients will be broken until this trial is finished.

3.4 Study Treatment

3.4.1 Surgeon and unit accreditation

The surgeon's experience and habits can impact various aspects of surgical performance, such as safety, operative time, and outcomes. For this trial, surgeons should complete more than 500 robot-assisted laparoscopic surgery procedures, including prostatectomy and partial nephrectomy. The surgical approach will be determined according to the protocol of the trial hospital where the patient is situated to facilitate the generalizability of the findings.

3.4.2 Structure of the telesurgery system and protective measures

The telesurgery system mainly consists of three subsystems: a surgical robotic subsystem, a telecommunications subsystem, and a teleconference subsystem. The robotic subsystem will consist of a main surgeon console

in the first surgeon’s hospital or laboratory and a patient cart in the operation room of the patients’ hospital. For safety purposes, a backup surgeon console will be positioned on the patient side in the operating room. In the event of a malfunction with the telesurgery system, the surgeon will take control of the surgery and complete the subsequent types. The telecommunication subsystem will consist of the router and communication line, which will be provided by the network operator. The teleconference subsystem, which can transmit the audio and visual input of the first surgeon and operating room, will be used for communication between the first surgeon and the assistant. The video documentation of all telesurgery procedures will be recorded by the teleconference subsystem. The video documentation of local surgery will also be recorded.

The status of the telesurgery system for the telesurgery group will be monitored by the technique group.

As a protective measure to ensure the safety of the telesurgery, the robotic subsystem has been updated. When the network latency is > 200 ms, the patient cart will stop working, and the telesurgery technique team will inspect and repair the mechanical malfunction of the system. Due to the prolonged duration required to rectify the issues, the surgeon

positioned at the patient’s side will take control of the surgery using the backup console and proceed with the remaining surgical steps.

3.4.3 Proctoring of surgery and dissemination of a standard technique

In this trial, robot-assisted laparoscopic prostatectomy and partial nephrectomy will be performed by the surgeon team. To reduce surgical heterogeneity, all procedures should adhere to standardized protocols, and all surgeons should receive in-person instruction and accreditation from Prof. Xu Zhang before the start of this trial. In prostatectomies, pelvic lymph node dissection will be carried out when the Gleason score of the prostatic biopsy is ⩾4+3. The anterior approach, which is the most common approach for prostatectomy, will be used. A transperitoneal or extraperitoneal approach can be selected by the medical team of each hospital and the first surgeon. For partial nephrectomies, the retroperitoneal approach should be the main approach.

3.4.4 Operation process of the telesurgery system

In the telesurgery group, the first surgeon will activate the main console and the teleconference subsystem on the surgeon's side, and the patient's side will activate the teleconference system, surgical cart, and backup console of the surgery robot. Telesurgery technical teams in Beijing and Urumqi/Harbin/Hangzhou/Hefei/Haikou will be responsible for the

network connection and equipment debugging between the doctor's console and the patient's surgical platform. After the correct connection and conditions of all the subsystems of the telesurgery, all the display screens will be turned off, and the patient will be taken to the operating room for the surgery. After the patient is anesthetized, all display screens will be turned on again, which is due to blind considerations.

3.5 Standard Follow-up for Patients and the Medical Team

Patients and medical team members will be followed up by the follow-up team of each center for this trial. Patients will be followed up three times, including at baseline (before the surgery), and 4 and 6 weeks after the surgery. The task load of the surgeon, first assistant, and instrument nurse will be measured immediately after the surgery. For telesurgery, the task load will be measured by the follow-up groups of the surgeon and patient sides together. Patients will continue to receive routine clinical follow-ups, and secondary outcomes will be measured up to 48 weeks.

3.6 Trial Assessments

Trial assessments will be conducted at certain time intervals (defined around the date of surgery):

(1) Baseline: Data will be recorded before the surgery.

(2) Perioperative: Operative details, task load of the medical team, and status monitoring data of the telesurgery system, including the operation

details, obvious side injury, and surgery video, and monitoring data of the telesurgery system, will be collected immediately after the surgery.

(3) In-hospital postoperative: The time to hospital discharge, length of days in critical care, reoperation intervention in the hospital, and blood transfusion rates will be recorded according to the clinical information system of each site.

(4) Four and six weeks after the surgery: The complications, tests, and questionnaires will be recorded or measured at 4 and 6 postoperatively. Further follow-up will continue to be performed at longer time points, including 12 weeks, 24 weeks, and 48 weeks.

The time point of the primary outcome is the finishing time of the surgery. The competition of surgery is defined as all the planned steps of the operation being finished without any conversion or obvious side injury. After the surgery, the blood loss, surgical approach, and any other surgical details will be recorded by the medical team.

The secondary outcome measures include the following:

QoR-15 Score: The QoR-15 score will be measured at baseline (before the surgery) and 4 and 6 weeks after surgery. The QoR-15 score is a patient-reported outcome questionnaire that measures the quality of postoperative recovery and fulfills the requirements for outcome measurement instruments in clinical trials.

EPIC-26 Score: The EPIC-26 score will be measured at baseline (before the surgery) and at 4 and 6 weeks after surgery.

30-second chair-to-stand test: The 30-second chair-to-stand test will be measured at baseline (before the surgery) and at 4 and 6 weeks after surgery.

Clavien-Dindo complication system: The Clavien-Dindo classification of surgical complications is a simple and widely used tool to assess and report postoperative complications in urologic surgery. The scores will be recorded at 4 and 6 weeks after the surgery.

Operative: Intraoperative surgical details will be recorded to compare telesurgery and local surgery. The conversion rate, operative blood transfusion rate, intraoperative blood loss, operative time, and warm ischemia time (for partial nephrectomy) will be recorded.

Postoperative: The postoperative conditions of the patients can reflect the effects and success of the surgery. Postoperative hospitalization days, length of days in critical care, reoperation intervention, the postoperative blood transfusion rate, and the mortality rate will be recorded through the clinical information system.

Oncological outcomes: The oncological outcomes for prostate cancer and renal cancer patients are key factors in the prognosis. After the surgery, the cancer sample will be viewed by two blinded pathologists.

The surgical margin rate will be focused.

3.7 End of Study

The end of study would be the last patient, last follow up clinic appointment.

4 STUDY SCHEDULE

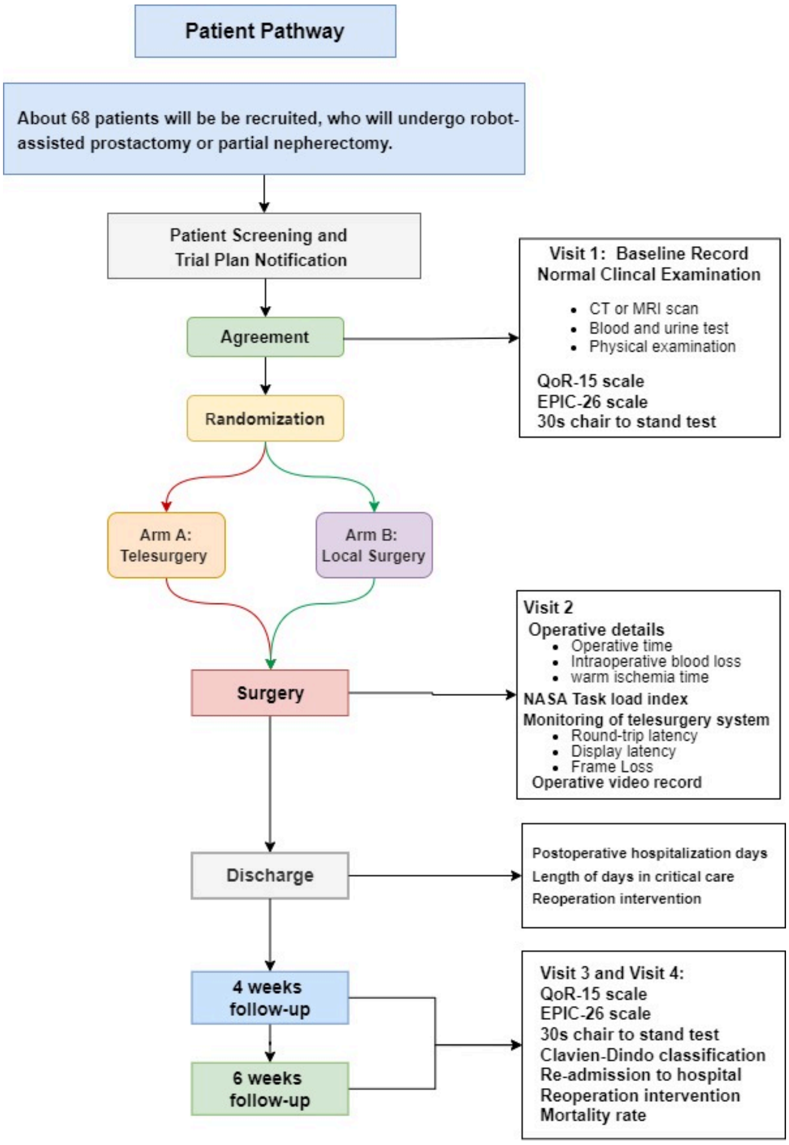

5 TABLE OF ASSESSMENTS

Items / Period	Screening	Surgery	Follow-up	
	Visit 1	Visit 2	Visit 3	Visit 4
	-7~0 day	Surgery day 0 day~3 day	4 weeks ±3 days	6 weeks ±3 days
Screening/grouping	●			
Informed consent & randomisation	●			
Fill in the basic information[1]	●			
Take medical history[1]	●			
Vital signs examination	●	●	●	●
Blood routine examination[1]	●	●	●	●
Biochemical examination[1]	●	●	●	●
Routine urine examination[1]	●			
Coagulation function[1]	●			
Preoperative pathological report[2]	●			
Electrocardiogram examination1[1]	●			
Chest CT/ X-ray[1]	●			
Lesion location CT/MR[1]	●			
Whether the operation was successful or not		●		
Operation time		●		
Blood loss volume		●		
Blood transfusion volume		●	●	
Surgery conversion		●		
Task load of the medical team		●		
Telesurgery system monitoring indicators[5]		●		
Length of days in hospital			●	
ICU time			●	
Positive margin of tumor sample [3]			●	
30 second chair to stand test [4]	●		●	●
QoR-15 scale	●		●	●
EPIC-26 Scale	●		●	●

Complication		●	●	●
Re-hospitalizations and emergency operations		●	●	●
Mobility		●	●	●
Instrument malfunction		●		
Record adverse events		●	●	●

1. These preoperative examinations will be performed by the medical system at the trial sites.

2. Preoperative pathological reports for patients with prostate cancer will be collected by the medical system at the trial sites or via paper reports from other hospitals.

3. The status of the surgical margin will be collected by the pathological report of the tumor, which is reviewed by one or two blinded pathologists.

4. The 30-s chair-to-stand test at baseline will be tested by the nurse before the surgery. In the follow-up period, this test will be performed in the outpatient service by the doctor. If the patients cannot go to the surgery hospital, this test will be performed by the follow-up nurse via WeChat (A widely popular social app in China that can perform various operations such as questionnaire investigation, voice and video).

5. The monitoring status of the telesurgery system will be collected by the telesurgery technique team.

6 PATIENT SCREENING & CONSENT

6.1 Patient Screening

During the patient screening period in each trial center, newly admitted or outpatient patients diagnosed with renal tumors or prostate cancer will be included in the screening. The patient information included in the screening will be naturally archived according to the clinical information system. Patients who meet the eligibility criteria for this clinical trial will be invited to participate.

6.2 Informed Consent

Patients who have been informed of their cancer diagnosis and approached about the suitability of robot-assisted partial nephrectomy or prostatectomy may be invited to participate in this trial.

It is the responsibility of the investigator to obtain written informed consent from (ICF) each patient prior to their entry into the study and before assessments that are not conducted as part of standard care are performed. A patient information sheet and ICF will be provided to facilitate this process. The investigator or their designee must ensure that they adequately explain to the patients the aims of the study, the study schedule, and follow-up visits, and that this study is a randomized controlled trial. At the same time, patients will be informed that the surgery will be performed by a Chinese robotic surgery system that has been registered with the Chinese Food and Drug Administration (CFDA). Patients should be informed that the surgery will be performed via telesurgery or normal local surgery.

The investigator or their designee will also inform patients that their participation is voluntary and that they can withdraw from the study at any time without it affecting their standard of care.

Patients will be given ample time and opportunity to inquire and ask questions about the trial and to decide whether or not to participate. The right of patients to refuse to participate in the trial, with or without giving

a reason, will be respected.

If a patient expresses an interest in participating in the study, they will be asked to sign and date the REC (Research Ethics Committee)-approved version of the ICF in the presence of the investigator, who will then co-sign and date the form. The patient will personally initial all boxes. A copy of the ICF will be given to the patient, a copy will be filed in the hospital notes, and the original will be placed in the investigator site file and mail to Chinese PLA General Hospital, when this trial is finished. A dated annotation will be made in the patient's medical notes stating that consent has been given, with the name of the trial and the version numbers of the ICF. Throughout the study, the patient will have the opportunity to ask questions about the study and any new information that may be relevant to the patient's continued participation.

By providing informed consent, participants will be consenting for the study to conduct follow-ups and collect their data.

6.3 Withdrawals

As participation in the study is entirely voluntary, participants may choose to discontinue at any time during this trial without penalty or loss of benefits to which they would otherwise be entitled.

7 ELIGIBILITY CRITERIA

7.1 Inclusion Criteria

Participants will have to meet the following criteria for inclusion in the study: (1) age, 18–80 years; (2) body mass index, 18–30 kg/m^2; (3) fit to undergo urologic laparoscopic surgery, including prostatectomy and partial nephrectomy; (4) physiological condition suitable for robot-assisted laparoscopic surgery; (5) willingness to cooperate and complete study follow-up and related examinations.

7.2 Exclusion Criteria

Participants who meet any of the following criteria will be excluded: (1) severe cardiovascular or circulatory diseases that are not tolerable for surgery; (2) pregnancy or lactation; (3) history of epilepsy or mental illness; (4) severe allergies or suspected/confirmed alcohol or drug addiction; (5) inability to understand study requirements or complete the study's follow-up schedule.

8 RECRUITMENT

All patients who have a diagnosis of renal tumor or prostate cancer and for whom partial nephrectomy or prostatectomy is recommended as a treatment option may be approached. It is important to keep patient withdrawals from the study to a minimum; however, a patient may withdraw from the study at any time without prejudice to their subsequent treatment.

In this randomized trial, all participants will be randomized to either

arm A (telesurgery group) or arm B (local surgery group).

9 STATISTICAL METHODS

9.1 Study Analysis

This section highlights the statistical considerations for this protocol. More details about statistical analysis are provided in a separate *Statistical Analysis Plan* document.

9.2 Sample Size Determination

According to the requirements of the "Guideline for the Design of Clinical Trials of Medical Devices" (published by CFDA), the number of trial cases was inferred based on the non-inferiority sample size calculation method, and the number of cases was calculated through joint discussion by clinical trial institutions, major researchers, and statistical analysts.

The formula is as follows：

$$n_T = n_C = \frac{\left(Z_{1-\alpha/2}+Z_{1-\beta}\right)^2 [P_C(1-P_C)+P_T(1-P_T)]}{(|D|-\Delta)^2},$$

where α = 0.025 (one-sided), β = 0.2 (80% confidence), and the non-inferiority margin Δ = 10%. Assuming that the surgical success rate of the experimental group and the control group is equal, the expected surgical success rate of the two groups is 98% (i.e., PT = PC = 98%). As the ratio between the two groups is 1:1, 31 subjects are needed in each group.

Considering that the dropout rate of subjects during the trial is about 10% or more, this study plans to enroll 68 subjects, with 34 in the experimental group and 34 in the control group. If the dropout rate exceeds the expected 10%, resulting in insufficient valid cases, the number of participants can be appropriately increased, and the number of the total valid cases should not be higher than 34 of each trial group. The increase in the number of patients enrolled should be filed with the Ethics Committee of Chinese PLA General Hospital.

Urologic surgery is divided into upper and lower urinary tract surgeries. In this clinical trial, representative surgical methods of partial nephrectomy and prostatectomy are selected for surgery. In the end, partial nephrectomy and prostatectomy in this scheme is no less than 30%, and each clinical center ensures that subjects are operated on for both partial nephrectomy and prostatectomy surgery. This information will be summarized after the end of the trial. According to the non-inferiority test, when the lower limit of the 95% confidence interval of the difference in surgical success rate between the two groups is > -10%, the non-inferiority hypothesis is established, which can be considered to meet the qualified requirements of this clinical trial.

9.3 Stopping Rules

There are no formal stopping rules for this clinical trial.

10 DATA COLLECTION AND HANDLING

10.1 Data Management

Source records are original documents, data, and records that are relevant to the clinical trial. The investigator will prepare and maintain adequate and accurate source documents, which are designed to record all observations and other pertinent data for each subject enrolled in this clinical trial. Source records will be adequate to reconstruct all data transcribed onto the CRFs (case report forms).

Participants will be given a unique random study number, and their data will be entered into this trial's database under their identification number.

After the study's database has been generated, the data will be submitted to the REC of Chinese PLA General Hospital for further review, when this trial is finished.

The clinical data, follow-up data, surgery video, and telesurgery system of the participants will be stored in the Telesurgery Research Center of Chinese PLA General Hospital. The written form will be stored in a locked filing cabinet, and electronic data will be stored in password-protected forms on the computers of the Telesurgery Research Center. The identification, screening, and enrollment logs will be stored at each trial site.

10.2 Data Collection, Handling, and Verification

Authorized site personnel will enter all required data into CRFs. The investigator will be responsible for reviewing, verifying, and approving all subject data prior to the study's completion.

Source data in clinical trials includes paper and electronic forms. Paper source data is mainly recorded on paper carriers, which can be divided into the following categories: (1) data generated by the researcher or authorized personnel of the researcher for the first time, such as inpatient and outpatient medical records; (2) data generated by the subjects themselves, such as informed consent; (3) data generated by the validated electronic clinical outcome assessment system of the subject for the first time in paper form, such as test report forms of the inspection department; (4) other data recorded by the researchers or subjects, such as original notes, memos, and other paper documents generated for the first time during the clinical trial; (5) certified copies of the above source data, such as the original data signed and approved by the main researchers in the paper case report form. Electronic source data will be attached to the computer system in electronic form and will mainly include the following categories: (1) data generated by the researcher directly input on-site during the validation of electronic data collection; (2) data generated and stored in electronic form by the validated electronic system during the

formation of test reports, inspection reports, and other processes, and obtained through the validation of system transmission. All source data/files will be reasonably preserved in accordance with relevant laws, regulations, and SOPs. For example, paper source data files should be waterproof and fireproof, thermal paper data carriers should be timely archived, and electronic source data should be stored for a certain period of time. The investigator will have control over the source data generated in their testing facility. However, when using eCRFs, the investigator will directly control the input, modification, review, and signature of source data in the eCRF/electronic database through an authorized account. When using paper case report forms, the investigator will have full control over the source data in the paper case report form and will authorize the data entry personnel to modify the source data in the electronic database through the data clarification form. Permissions will be set up for control, access, verification, and review of data files, and appropriate work will be carried out only with the approval of permission to ensure the rights and interests of subjects and avoid unauthorized changes.

11 PEER AND REGULATORY REVIEW

Obtaining funding of this trial from Chinese PLA General Hospital and National Natural Science Foundation of China and the process is

independent and sufficient rigour, which can be considered an adequate peer review.

12 ASSESSMENT AND MANAGEMENT OF RISK

The risk posed to the control arm (i.e., the local surgery group) has been determined to be no higher than that of standard medical care. However, the trial arm (i.e., the telesurgery group) may have the potential risk of a malfunction of the telesurgery system with more complex structure and subsystem.

12.1 Emergency Plan for Telesurgery

1) Network exceptions (including exceptions for image signals and control commands)

If the network is abnormal and cannot be recovered in time, the investigator will make a timely decision on whether to transfer to the local operation according to the on-site environment.

2) Abnormal operation during the procedure (such as the inability to effectively stop bleeding or perform an accurate operation)

If the network is normal but effective treatment is not possible at the patient end, the investigator will make a timely decision on whether to transfer to a local operation based on a quick discussion with the medical team.

12.2 Protection Strategy for the Telesurgery

Firstly, the two surgeon consoles will be set up on both the planned first surgeon side and the patient side. The backup surgeon console will be used for the malfunction of the telesurgery system and can be used by the surgeon on the patient side to complete all the surgery steps.

Telesurgery has been adapted to the characteristics of remote operation. The real-time network latency is monitored by the system, and the surgery robot will be locked when the network latency is > 200 ms.

13 RECORDING AND REPORTING OF EVENTS AND INCIDENTS

13.1 Definitions of Adverse Events

The Clavien-Dindo classification will be used to record the postoperative complications. All unexpected adverse events not included in this category will be recorded in the patients' medical notes. Any untoward medical occurrence in a patient will be defined as an adverse event, which may not have a causal relationship with the surgery.

A serious adverse event (SAE) is defined as any adverse event that results in death, is life-threatening, or results in any serious side injury to the participant during the surgery due to a malfunction of the telesurgery system.

13.2 Procedures for Recording and Reporting Serious Adverse Events

All SAEs will be recorded in the medical records and the CRF. SAEs will also be reported to the chief investigator within 1 hour. A SAE analysis meeting including the technique and surgeon group will review and analyze the SAEs as soon as possible. The video of the surgery and the telesurgery monitoring data will be reviewed to determine the cause of the SAE.

14 HUMAN SUBJECTS

14.1 Ethical Considerations

This clinical trial will fully be conducted in accordance with the ethical principles of the Helsinki Declaration, CFDA regulations, and REC requirements, adhering to the principle that the rights, safety, and will of the research subjects are paramount to the needs of research. Strict confidentiality measures will be taken for the research subjects, and their personal privacy will be respected to ensure that they will not suffer any discrimination or harm due to the results of the study.

14.2 Communication with the Research Ethics Committee

This protocol, the ICF, the Investigator's Brochure, and any information to be given to the subject must be submitted to a properly constituted REC by the investigator for review and approval by the REC before the study is initiated. In addition, the REC must approve all subject recruitment materials before they are used for subject recruitment. The

investigator is also responsible for promptly informing the REC of any amendments or changes to the protocol or the Investigator's Brochure. Written documentation of REC approval must be received before the amendment is implemented.

14.3 Informed Consent Form

No study procedure will be implemented prior to obtaining a signed, written ICF from the subject or the subject's legally authorized representative. REC review and approval are required for the ICF. If there are any changes to the ICF during the subjects' participation in the study, REC's written approval must be received, and subjects must consent to the revised version of the ICF before it is to be used.

Notes for the informed consent process are listed below:

(1) The researcher or the assigned responsible person will carry out the informed consent process.

(2) The informed consent process should cover every aspect related to the target subject's decision (whether to participate in the clinical study or not).

(3) Coercion, inducement, or imposition of inappropriate influence on the target subject should be avoided.

(4) The target subject will have the right to preserve their legal rights and interests.

(5) The target subject's native language will be used to provide intelligible and non-technical descriptions to facilitate their understanding.

(6) Sufficient time will be provided for the target subject to read and understand the "Informed Consent Form" and consider whether to participate in the clinical study.

(7) It should include the personal signatures of the target subject and the researcher or the assigned responsible person.

(8) The subject will be provided with a signed and dated copy of the "Informed Consent Form" or other handwritten materials.

(9) The procedure for obtaining and filling out the "Informed Consent Form" will be explained in special cases where the subject is unable to handle it themselves.

(10) Important new information will be provided to newly joined and current subjects throughout the entire clinical study process.

14.4 Subject Confidentiality

All data collected from this trial will be kept confidential to the extent required by law. Subject identification in clinical trial-related materials will primarily be based on random numbers and abbreviations of subject names in pinyin. Subjects' personal data will be recorded in a subject identification coding table, which will be kept confidential by the principal investigator. Personal information will not be disclosed without

the subject's written consent. The REC, CFDA, the Health and Health Commission Authorities of China, or the sponsor may access the personal data of subjects participating in the trial as needed, in accordance with established procedures. The content of this trial may be published, but any published content will not include any personal information about the subjects. Medical information on the subjects obtained as part of this study will be confidential and may only be disclosed to third parties as allowed by applicable regulations.

15 INDEMNITY ARRANGEMENTS

The research group will hold insurance against claims from participants for harm caused by their participation in this clinical study. The hospital will continue to have a duty of care to the participants who have sustained injuries associated with this clinical trial.

16 PUBLICATION AND DISSEMINATION POLICY

All materials and test results related to this clinical trial will be jointly owned by the researchers and the collaborators. The lead institution will have the right to publish a summary report of the clinical trial in the form of a paper, and the researchers from each participating institution will be entitled to authorship of the paper. However, consent from the collaborators must be obtained prior to publication. Before submission, any content intended for publication must be submitted to the

collaborators, who will review its accuracy, ensure that confidential information is not leaked, and supplement relevant information.

17 FUNDING AND SUPPLY OF EQUIPMENT

The study will be funded by the National Natural Science Foundation of China and the Foundation of Chinese PLA General Hospital.

The surgery robot system will be provided by EdgeMedical (Shenzhen, China). The telesurgery system will be built by the telesurgery research group of Chinese PLA General Hospital and EdgeMedical.

18 References

1. Gawande A. Two Hundred Years of Surgery. N Engl J Med. 2012;5:479.
2. Gu L, Yin C, Jia T, He K, Ma X, Zhang X. Robotic surgery in China. The Innovation 2023;4:100499.
3. Mack MJ. Minimally Invasive and Robotic Surgery. JAMA 2001;5:568-72.
4. Patel V, Marescaux J, Covas MM. The Humanitarian Impact of Telesurgery and Remote Surgery in Global Medicine. EUR UROL 2024. :S0302-2838(24)02357-1
5. Larkin M. Transatlantic, robot-assisted telesurgery deemed a success. The Lancet 2001;358:1074.
6. Wang Y, Ai Q, Zhao W, et al. Safety and Reliability of a Robot-assisted Laparoscopic Telesurgery System: Expanding Indications in Urological Surgery. EUR UROL 2024;85:506-7.
7. Choi PJ, Oskouian RJ, Tubbs RS. Telesurgery: Past, Present, and Future. Cureus 2018. 10(5):e2716

Statistical Analysis Plan (SAP)

A Prospective, Multicenter, Single blind, Randomized Controlled Trial on the safety and reliability of telesurgery robotic system on urology

**STUDY TITLE:**	A Prospective, Multicenter, Single blind, Randomized Controlled Trial on the safety and reliability of telesurgery robotic system on urology
**PROTOCOL NUMBER:**	JF-MP-IITP-007
**SPONSOR & LEADING CENTER:**	Xu Zhang, MD, Ph D., Department of Urology，Chinese PLA General Hospital, Beijing, China
**PROTOCOL VERSION/DATE:**	Version 2.0, February 28th, 2025

Signatures Page

The undersigned confirm that the following Statistical Analysis Plan has been agreed and accepted and that the Trial Statistician agrees to conduct the analysis in compliance with the approved Statistical Analysis Plan. Major deviations from the Plan will be agreed in advance before implementation. All deviations from the Plan will be explained, documented and reported accordingly.

Trial Statistician:

Signature: ....

Date: ....

Principal investigator:

Signature: ....

Date: ....

Table of Contents

1.Introduction · · · 5

2. Study Objectives · · · 5

2.1 Primary Objective · · · 5

2.2 Secondary Objectives · · · 5

3. Trial design · · · 5

3.1 Study Arms · · · 7

3.2 Sample Size · · · 7

3.3 Randomization · · · 8

4. Study Outcomes · · · 9

4.1 Primary outcome · · · 9

4.2 Secondary outcomes · · · 9

4.2.1 Early recovery · · · 10

4.2.2 Perioperative morbidity · · · 11

4.2.3 Oncological outcomes · · · 12

4.2.4 Medical team task load · · · 11

4.2.5 Telesurgery monitoring data · · · 12

5. Definitions · · · 12

5.1 Successful Completion of the Surgery · · · 12

5.2 Serious Adverse Events (SAE) · · · 14

5.3 Malfunction of the surgery system · · · 14

6. Analysis population · · · 14

6.1 Intention-to-treat (ITT) Analysis Set · · · 14

6.2 Per-Protocol Analysis Set (PPS) · · · 15

7.Interim Analysis · · · 15

8. Data analysis plan · · · 15

8.1 Recruitment and representativeness of recruited patients · · · 15

8.2 Baseline characteristics · · · 15

8.2.1 Demographics · · · 15

8.2.2 Characteristics of tumor or the target organ · · · 16

8.3 Efficacy Analysis · · · 16

8.3.1 Primary outcome analysis · · · 16

8.3.2 Second Outcomes analysis · · · 17

8.3.3 Telesurgery monitoring data analysis · · · 17

8.4 Loss to follow-up and other missing data · · · 18

8.5 Adverse event reporting · · · 18

8.6 Software · · · 28

1. Introduction

This SAP describes the statistical analyses to be performed for the data from the clinical trial entitled "A Prospective, Multicenter, Single blind, Randomized Controlled Trial on the safety and reliability of telesurgery robotic system on urology" (Protocol JF-MP-IITP-007). This clinical trial will be conducted to test the safety and reliability of telesurgery, which is a new developing field of surgery. This clinical trial will be led by Chinese PLA General Hospital.

2. Study Objects

2.1 Primary object

The primary objective of this clinical trial will be to evaluate and validate the safety and efficacy of telesurgery systems in urologic surgery compared with local surgery. The success rate of the surgery will be recorded.

2.2 Secondary objects

The secondary objective of this study will be to investigate differences between the telesurgery and local surgery groups in terms of complications and adverse events, early recovery quality, physical functioning, early oncological outcome, task load of the surgeon and medical team, and robotic or telesurgery system status or malfunction.

3. Trial design

This will be a prospective, multicenter, single-blind, non-inferiority, exploratory, randomized controlled trial aimed at exploring the safety and reliability of telesurgery.

Participants will be randomized to either the telesurgery group or the local surgery group.

There are four study periods:

(1) Screening Period;

(2) Randomization Period;

(3) Surgery Period;

(4) Follow-up Period.

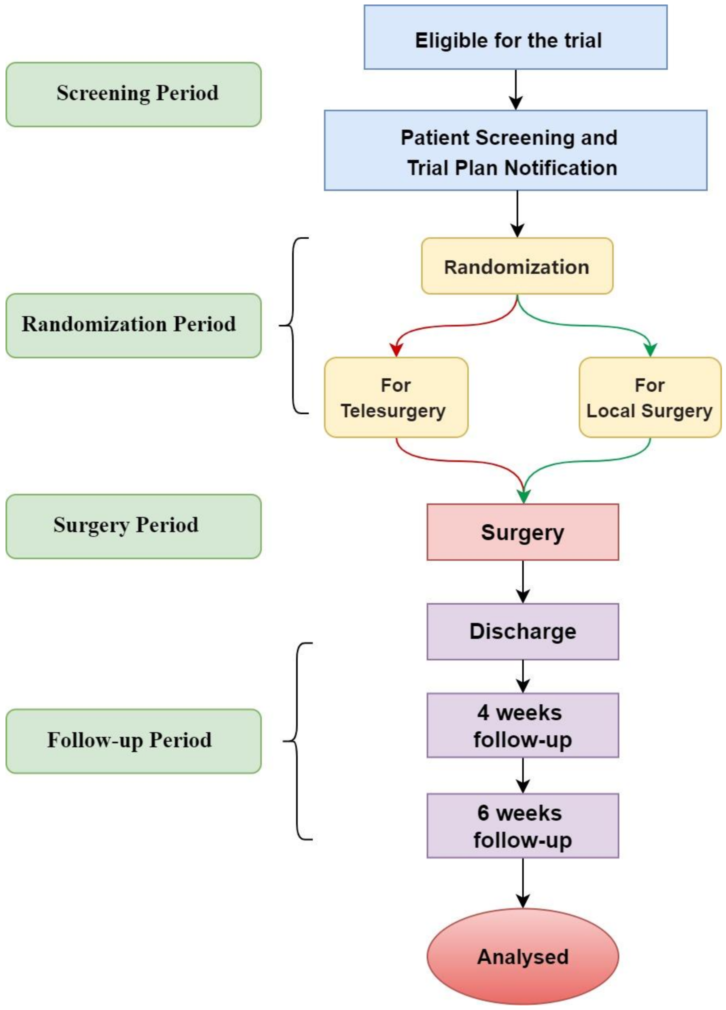

Figure 1. Schematic Flowchart of the Study Design.

3.1 Study Arms

In this randomized trial, all participants will be randomized to either arm A (telesurgery group) or arm B (local surgery group).

3.2 Sample Size

According to the requirements of the "Guideline for the Design of Clinical Trials of Medical Devices" (published by CFDA), the number of trial cases was inferred based on the non-inferiority sample size

calculation method, and the number of cases was calculated through joint discussion by clinical trial institutions, major researchers, and statistical analysts.

The formula is as follows：

$$n_T = n_C = \frac{\left(Z_{1-\alpha/2}+Z_{1-\beta}\right)^2 [P_C(1-P_C)+P_T(1-P_T)]}{(|D|-\Delta)^2},$$

where α = 0.025 (one-sided), β = 0.2 (80% confidence), and the non-inferiority margin Δ = 10%. Assuming that the surgical success rate of the experimental group and the control group is equal, the expected surgical success rate of the two groups is 98% (i.e., PT = PC = 98%). As the ratio between the two groups is 1:1, 31 subjects are needed in each group. Considering that the dropout rate of subjects during the trial is about 10% or more, this study plans to enroll 68 subjects, with 34 in the experimental group and 34 in the control group. If the dropout rate exceeds the expected 10%, resulting in insufficient valid data, the number of patients enrolled can be appropriately increased, and the proposed increase in the number of patients enrolled should be filed with the Ethics Committee of Chinese PLA General Hospital.

Urologic surgery is divided into upper and lower urinary tract surgeries. In this clinical trial, representative surgical methods of partial

nephrectomy and prostatectomy are selected for surgery. In the end, partial nephrectomy and prostatectomy in this scheme is no less than 30%, and each clinical center ensures that subjects are operated on for both partial nephrectomy and prostatectomy surgery. This information will be summarized after the end of the trial. According to the non-inferiority test, when the lower limit of the 95% confidence interval of the difference in surgical success rate between the two groups is > -10%, the non-inferiority hypothesis is established, which can be considered to meet the qualified requirements of this clinical trial.

3.3 Randomization

This study will adopt a random number distribution system. The random grouping table of subjects will be generated by independent statisticians (who will not participate in the clinical trial) from random coding units. The ratio of the test group to the control group will be 1:1. The seed number for generating the random table is fixed, and the process of generating the random table can be repeated. The generated random table of subjects will be imported into the random system by the random system administrator. All random numbers will not be used twice. After the successful screening of subjects, the random responsible person of each center will log in to the system and enter the basic information of

subjects according to the system prompts, including the date of birth and the name abbreviation, etc. The system will generate a unique random number for each subject and randomly allocate subjects to the groups. We used stratified randomization with random block sizes of four. Stratification was identified by the surgery type (prostatectomy and partial nephrectomy) with the randomization conducted separately for each stratum.

4 Outcomes

4.1 Primary outcome:

The primary objective of this clinical trial will be to evaluate and validate the safety and efficacy of the telesurgery system in urologic surgery and compare it to local urologic surgery. The success was defined as the surgery being completed effectively as planned, without conversion to other surgical types and significant side injury or delays due to system malfunctions. Telesurgery was considered successful if it could be completed only using the telesurgery system without conversion to local surgery for any reason. The success or failure of each surgery will be jointly confirmed by the remote and local medical teams together.

4.2 Secondary outcomes:

The secondary outcomes of this study will be to investigate differences

between telesurgery and local surgery in terms of complications and early recovery quality, physical functioning, early oncological outcomes, task load of the surgeon and medical team, adverse events, and status or malfunction of the local surgery robotic or telesurgery system.

4.2.1 Early recovery

The following tools will be used to measure the early recovery:

1. Overall functional recovery: Quality of recovery 15-item score (QoR-15 score) at baseline (preoperative), 4 weeks, and 6 weeks.

2. Overall functional recovery for prostate cancer patients: Expanded Prostate Cancer Index Composite-26 (EPIC-26 score) at baseline (preoperative), 4 weeks, and 6 weeks.

3. Physical activity: 30-second chair-to-stand test (number of times the patient can stand from sitting in a 30-second interval) at baseline (preoperative), 4 weeks, and 6 weeks.

4.2.2 Perioperative morbidity

To assess perioperative morbidity, the following clinical data will be collected:

1. Surgery-associated factors: intraoperative blood loss, operative time, and warm ischemia time (for partial nephrectomy).

2. Complications and adverse events: Adverse events will be recorded

using the Clavien-Dindo classification at 4 weeks and 6 weeks postoperatively.

3. Postoperative hospitalization days.

4. Length of days in critical care.

5. Reoperational intervention.

6. Readmission to the hospital: Readmission to the hospital for any reason will be recorded, and the surgery-associated condition will be systemically reviewed.

7. Intra- and postoperative blood transfusion rates.

8. Mortality rate.

4.2.3 Oncological outcomes

To assess the oncological outcomes, the following pathological data will be collected:

1. Positive margin rate in the pathological specimen according to the pathology report from two blinded pathologists in each hospital.

2. Pathological type of the tumor;

3. The Gleason score and number of retrieved lymph nodes in the pathological specimen for prostatectomy with lymph node dissection only.

4.2.4 Task load of medical team

The NASA (National Aeronautics and Space Administration) Task Load Index (NASA-TLX) will be used to measure the task load of the first surgeon, first assistant, and instrument nurse immediately after the surgery in both groups.

4.2.5 Telesurgery monitoring data

To characterize the status of the telesurgery system, the following data will be collected:

1. Round-trip network latency (for telesurgery): The round-trip network latency is defined as the time latency of data packages between transmission and reception.

2. Display latency (for telesurgery): The display latency is defined as the time latency between coding and encoding the endoscope image.

3. Frame loss (for telesurgery): The frame loss is defined as the loss of data packages during data transmission.

4. Malfunction: The malfunction of the local surgical robot or telesurgery system will be recorded. The local surgical robot or telesurgery system is tested systemically one day preoperatively. A malfunction of the telesurgery system is defined as the system being out of control for any reason, such as a telecommunications pathway fault, a laboratory pathway fault, or a robotic fault. A malfunction of the local surgical robot

is defined as the robot being out of control for any reason.

5. Definitions

5.1 Success of the surgery

The success was defined as the surgery being completed effectively as planned, without conversion to other surgical types and significant side injury or delays due to system malfunctions. Telesurgery was considered successful if it could be completed only using the telesurgery system without conversion to local surgery for any reason.

5.2 Serious adverse events

A serious adverse event is defined as any adverse event that results in death, is life-threatening, or results in any side injury to the participant during the surgery due to a malfunction of the telesurgery system.

5.3 Malfunction of the surgery system

The malfunction of the telesurgery system is defined as the system being out of control for any reason, such as a telecommunications pathway fault, a laboratory pathway fault, or a robotic fault. A malfunction of the local surgical robot is defined as the robot being out of control for any reason.

6. Analysis population

6.1 Intention-to-treat (ITT) Analysis Set

The ITT population includes all randomized participants of this trial. Missing data of the primary outcome were imputed by Multiple Imputation (e.g., fully conditional specification). Unless otherwise specified, other missing data will not be interpolated.

6.2 Per-Protocol Analysis Set (PPS)

The PPS population consists of all randomized participants who received surgery, including telesurgery and normal local surgery.

7. Interim Analysis

Not appliable.

8. Data Analysis Plan

8.1 Recruitment and representativeness of recruited patients

A CONSORT-style flow chart will be constructed based on aggregated data. This will include the following: (1) the number of eligible patients; (2) the number of patients who agree to enter the trial with a brief description of the reasons; (3) the number of patients who are randomized and undergo follow-up; (4) the number of patients who withdraw in any period of this trial; (5) the number of patients lost to follow-up; and (6) the number of patients excluded or analyzed.

8.2 Baseline characteristics

8.2.1 Demographics

A table will present the overall baseline characteristics of the patients. One table each will present the baseline characteristics of patients with renal tumors and patients with prostate cancer. The demographic data will include the age (years), sex, height (m), weight (kg), and body mass index (BMI, kg/m^2) of the patients.

8.2.2 Characteristics of tumor or the target organ

For renal tumors, the R.E.N.A.L. score will be reported. For prostate cancer, the volume of the prostate and the Gleason score will be reported. These factors, along with the BMI, can influence and reflect the difficulty level of the surgery.

8.3 Efficacy analysis

8.3.1 Primary outcome analysis

The primary outcome will be analyzed by a non-inferiority analysis. According to the medical statistics scenario, the main efficacy evaluation is based on the intention-to-treat population set and per-protocal population set. The statistical description and inference will be based on the characteristics of the data, and the appropriate descriptive indicators and hypothesis testing methods will be selected.

The primary efficacy indicator of surgical success rate uses a non-inferiority test, with the following hypotheses tested:

Null hypothesis: H0: PT – PC ≤ -△;

Alternative hypothesis: H1: PT – PC > -△.

At α = 0.025 (one-sided test) and β= 0.2 levels, the success rates of the two groups will be statistically described and compared, and the 95% confidence interval (CI) of the difference in success rates between the two groups will be calculated. If the lower limit of the 95% CI of the difference in success rates between the two groups is greater than the non-inferiority margin (-△), it can be concluded that the experimental group is non-inferior to the control group.

The difference in the primary outcome (success rate) between the telesurgery and local surgery groups will be compared using the Miettinen-Nurminen method or Newcombe-Wilson score method test, according to the success rate data. The tipping assay can be used to determine the robustness and sensitivity the statistical conclusion.

8.3.2 Secondary outcomes analysis

The differences in secondary outcomes will be compared as follows:

(1) Categorical data: logistic regression (with Firth's correction) or Exact Fisher's test；

(2) Continuous data: linear regression

The hospital or surgery type can be used as adjusting factors.

8.3.3 Telesurgery monitoring data analysis

The telesurgery monitoring data is descriptive statistic data. The median, along with the interquartile range, minimum, and maximum of different latency values, will be reported. The number and percentage of frame losses will also be reported.

8.4 Adverse event reporting

Adverse events, adverse reactions, serious adverse events, and serious adverse reactions will be summarized.

8.5 Software

Version 9.4 or greater of SAS statistical software package will be used to provide all summaries, listings, and graphs of the clinical data described in this document.

The R Studio and GraphPad Prism 8.0 will be used for the visualization of telesurgery monitoring data, including round-trip latency distribution, real-time round-trip network latency, display latency distribution, real-time display latency and frame loss.
