# Supplementary material for: Reliability of urological telesurgery compared with local surgery: multicentre randomised controlled trial
Source: BMJ. 2026 Jan 28;392:e083588. doi: 10.1136/bmj-2024-083588 (PMC12849050; doi:10.1136/bmj-2024-083588)
Supplement: Supplementary file 3 — Web appendix: Statistical codes [file wany083588.ww3.pdf]

中国人民解放军总医院
CHINESE PLA GENERAL HOSPITAL

Statistical Codes
for SAS and R language

Manuscript Title: Reliability of Urological Telesurgery compared to Local Surgery：A Multicenter， Randomized Control Trial

SPONSOR & LEADING CENTER: Xu Zhang, MD, Ph D.

Department of Urology，Chinese PLA General Hospital, Beijing, China

Table of Contents

1. Bayesian mixed-effects logistic regression with penalized priors for ITT of the primary outcome (The R Programming Language) 4

2. Bayesian mixed-effects logistic regression with penalized priors for PPS of the primary outcome (The R Programming Language) 15

3. Mixed-effects Linear regression analysis 15

3.1 Operative time 26

3.2 Warm ischemia time 31

3.3 Blood loss 35

3.4 Postoperative hospitalization days 41

3.5 Length of days in critical care 46

3.6 QoR15 Score 51

3.6.1 Total for renal tumor and prostate cancer patients 51

3.6.2 For Renal tumor patients 68

3.6.3 For Prostate cancer patients 79

3.7 '30 Second Chair to Stand test 91

3.7.1 Total for renal tumor and prostate cancer patients 91

3.7.2 For Renal tumor patients 107

3.7.3 For Prostate cancer patients 107

3.8 EPIC-26 Score 108

3.8.1 Total 108

3.8.2 For Sexual function 119

3.8.3 For Urine control 129

3.9 NASA-TXL Score 138

3.9.1 Surgeon 138

3.9.2 First Assistant 144

3.9.3 Instrument nurse 144

4. Mixed-effects Logistic regression with Firth's correction (Sensitive analysis for Positive surgical margin) 148

5. Exact Fisher's test for Clavin-Dindo Complications 153

6. Bayesian mixed-effects logistic regression (Main analysis for the Positive margin;The R Programming Language) 160

9. Univariate linear regression (The R Programming Language) 163

10. Box-plot (The R Programming Language) 165

11. Violin Plot combined with Box-plot (The R Programming Language) 166

1. Bayesian mixed-effects logistic regression with penalized priors for ITT of the primary outcome (The R Programming Language)

```
# Install necessary packages (run if not installed)
# install.packages(c("mice", "brms", "dplyr", "parallel", "bayesplot", "broom.mixed", "tidyr",
"purrr", "ggplot2", "posterior"))
# install.packages("cmdstanr") # Backend for brms, recommended to install and configure

library(mice)
library(brms)
library(dplyr)
library(parallel)
library(tidyr)
library(purrr)
library(ggplot2)
library(posterior) # For working with posterior samples

# Set non-inferiority margin
NON_INFERIORITY_MARGIN <- -0.1  # Non-inferiority margin for rate difference

# Check and configure GPU acceleration
configure_gpu_acceleration <- function() {
  # Check if cmdstanr is installed
  if (!requireNamespace("cmdstanr", quietly = TRUE)) {
    message("cmdstanr not installed. Installing now...")
    install.packages("cmdstanr",        repos        =        c("https://mc-stan.org/r-packages/",
getOption("repos")))
  }

  # Check if CUDA is available
  cuda_available <- tryCatch({
    cmdstanr::install_cmdstan(cores = parallel::detectCores(), overwrite = FALSE, cpp_options
= list("CUDA" = TRUE))
    TRUE
  }, error = function(e) FALSE)

  if (cuda_available) {
    message("CUDA detected. GPU acceleration will be used.")
    return(list(use_gpu = TRUE, backend = "cmdstanr"))
  } else {
    message("CUDA not available. Using CPU only.")
    return(list(use_gpu = FALSE, backend = "rstan"))
  }
}
```

```
# Configure GPU acceleration
gpu_config <- configure_gpu_acceleration()
use_gpu <- gpu_config$use_gpu
backend <- gpu_config$backend

# Read and prepare data
# Data details: group: 1=telesurgery group, 2=local surgery group; hospital: 1=Beijing, 2=Harbin, 3=Hangzhou, 4=Hefei, 5=Urumqi; surgery_type: 1=Radical Prostatectomy, 2=Partial Nephrectomy; surgeon codes: 1=Chaozhao Liang, 2=Baojun Wang, 3=Xu Zhang, 4=Sheng Tai, 5=Hongzhao Li, 6=Wanghai Xu, 7=Xin Ma, 8=Qingbo Huang, 9=Weijun Fu, 10=Shuo Wang, 11=Mulati Rexiati

dat_text <- "
group hospital  surgery_type surgeon success
1 4 1 1 1
1 4 1 2 1
2 4 1 3 1
2 4 1 3 1
2 1 2 4 1
2 1 2 4 1
1 1 2 5 1
1 1 1 5 1
2 2 2 5 1
2 2 2 5 1
1 2 2 4 1
1 2 2 4 1
2 1 1 6 1
1 1 2 3 1
1 1 1 3 1
2 1 1 6 1
2 4 2 3 1
2 4 2 7 1
1 4 1 8 1
2 4 1 3 1
2 1 1 1 1
2 2 1 5 1
1 2 1 8 1
1 2 1 4 1
2 1 1 6 1
1 4 2 4 1
1 1 2 3 1
1 1 1 9 1
1 3 2 4 1
```

1	1	2	9	1
1	1	1	9	1
1	3	1	8	1
2	3	1	9	1
2	3	2	9	1
1	1	1	9	1
2	1	2	4	1
2	3	1	9	1
1	3	1	8	1
2	3	1	9	1
2	3	1	9	1
1	3	2	4	1
1	3	2	4	1
2	3	2	9	1
1	5	2	4	1
1	5	1	8	1
2	3	2	9	1
2	3	2	9	1
1	3	2	4	1
2	1	1	6	1
2	5	2	10	0
2	5	2	10	1
2	1	2	10	1
1	5	1	8	1
1	5	2	4	1
2	1	2	4	1
1	1	1	6	1
1	1	2	10	1
2	1	1	2	1
1	1	2	10	1
1	5	1	4	1
1	5	1	8	1
2	5	1	11	1
2	5	1	6	1
2	2	1	5	NA
1	4	2	8	NA
1	4	1	8	NA
2	4	1	7	NA
2	1	2	4	NA
1	3	1	8	NA
2	5	1	11	NA
1	3	1	6	NA
2	5	1	11	NA"

```
dat <- read.table(text = dat_text, header = TRUE, na.strings = "NA")

dat <- dat %>%
  mutate(
    hospital = factor(hospital),
    group = factor(group, levels = 1:2, labels = c("telesurgery", "local")),
    surgery_type = factor(surgery_type, levels = 1:2, labels = c("RadicalProstatectomy",
"PartialNephrectomy")),
    surgeon = factor(surgeon),
  )

# Multiple imputation (only for 'success' variable)
ini <- mice(dat, maxit = 0, printFlag = FALSE)
pred <- ini$predictorMatrix
pred[,] <- 0
pred["success", c("hospital", "group", "surgery_type", "surgeon")] <- 1

n_imputations <- 50    # Number of imputations
n_cores <- 20          # Number of parallel processing cores

cl <- makeCluster(n_cores)
clusterExport(cl, c("dat", "pred", "mice"))
clusterEvalQ(cl, library(mice))

impute_one <- function(i) {
  set.seed(20250802 + i)
  mice(dat, m = 1, maxit = 10, predictorMatrix = pred, method = "logreg",
       printFlag = FALSE)
}

imputations_list <- parLapply(cl, 1:n_imputations, impute_one)
stopCluster(cl)

options(mc.cores = parallel::detectCores())

# Define different adjustment models (surgeon as random effect)
models <- list(
  unadjusted = list(
    name = "Unadjusted",
    formula = bf(success ~ group),
    description = "No covariates",
    random = FALSE
  ),
  adjust_surgeon = list(
```

```
    name = "Adjusted for Surgeon (random)",
    formula = bf(success ~ group + (1 | surgeon)),
    description = "Surgeon as random effect",
    random = TRUE
  ),
  adjust_hospital = list(
    name = "Adjusted for Hospital",
    formula = bf(success ~ group + hospital),
    description = "Hospital as covariate",
    random = FALSE
  ),
  adjust_surgery_type = list(
    name = "Adjusted for Surgery Type",
    formula = bf(success ~ group + surgery_type),
    description = "Surgery type as covariate",
    random = FALSE
  ),
  adjust_all = list(
    name = "Adjusted for All (surgeon random)",
    formula = bf(success ~ group + hospital + surgery_type + (1 | surgeon)),
    description = "All covariates (surgeon as random effect)",
    random = TRUE
  )
)

# Set prior distributions
fixed_prior <- prior(normal(0, 5), class = "b")
random_prior <- prior(cauchy(0, 2), class = "sd")

# Initialize storage for model results across all imputed datasets
all_models <- vector("list", length = n_imputations)

# Model fitting function with GPU acceleration
fit_model_with_gpu <- function(formula, data, priors, model_name, i, j) {
  # Set model parameters
  control_list <- list(adapt_delta = 0.95)
  stan_model_args <- NULL

  # Configure GPU acceleration
  if (use_gpu) {
    # Use OpenCL for GPU acceleration
    stan_model_args <- list(
      cpp_options = list(
        STAN_OPENCL = TRUE,
```

```
      OPENCL_DEVICE_ID = 0,
      OPENCL_PLATFORM_ID = 0
    )
  )

  # Increase iterations for complex models
  if (grepl("All", model_name)) {
    iter_val <- 3000
  } else {
    iter_val <- 2000
  }
} else {
  iter_val <- 2000
}

# Fit model
fit <- tryCatch({
  brm(
    formula = formula,
    data = data,
    family = bernoulli(),
    prior = priors,
    chains = 2,
    iter = iter_val,
    seed = 20250802 + i + j,
    control = control_list,
    refresh = 0,
    silent = TRUE,
    save_pars = save_pars(all = TRUE),
    backend = backend,
    stan_model_args = stan_model_args
  )
}, error = function(e) {
  # Fall back to CPU if GPU acceleration fails
  message("GPU acceleration failed. Falling back to CPU.")
  brm(
    formula = formula,
    data = data,
    family = bernoulli(),
    prior = priors,
    chains = 2,
    iter = 2000,
    seed = 20250802 + i + j,
    control = control_list,
```

```
      refresh = 0,
      silent = TRUE,
      save_pars = save_pars(all = TRUE)
    )
  })

  return(fit)
}

# Main loop: Fit all models
for (i in seq_len(n_imputations)) {
  dat_imp <- complete(imputations_list[[i]])
  dat_imp$success <- as.integer(dat_imp$success)

  model_results <- list()

  for (j in seq_along(models)) {
    model_name <- names(models)[j]
    model_spec <- models[[j]]

    cat("Fitting model:", model_spec$name, "for imputation", i, "\n")

    # Select priors based on model type
    current_priors <- if (model_spec$random) {
      c(fixed_prior, random_prior)
    } else {
      fixed_prior
    }

    # Fit model using GPU acceleration
    start_time <- Sys.time()
    fit <- fit_model_with_gpu(
      formula = model_spec$formula,
      data = dat_imp,
      priors = current_priors,
      model_name = model_spec$name,
      i = i,
      j = j
    )
    end_time <- Sys.time()

    # Extract posterior samples
    post_samples <- as_draws_df(fit)
```

```
    # Calculate model fitting time (minutes)
    fit_time_minutes <- as.numeric(difftime(end_time, start_time, units = "mins"))

    model_results[[model_name]] <- list(
      fit = fit,
      post_samples = post_samples,
      model_name = model_spec$name,
      description = model_spec$description,
      is_random = model_spec$random,
      fit_time = fit_time_minutes
    )

    # Print model fitting time
    cat("  Model fitting time:", round(fit_time_minutes, 2), "minutes\n")
  }

  all_models[[i]] <- model_results
  cat("Completed models for imputation:", i, "/", n_imputations, "\n")
}

# Function: Compute rate difference from posterior samples (using Bayesian pooling)
compute_rate_difference_bayes <- function(model_list, non_inferiority_margin =
NON_INFERIORITY_MARGIN) {
  # Combine posterior samples from all imputed datasets
  all_samples <- map_dfr(model_list, ~ .x$post_samples)

  # Find group effect parameter
  param_name <- grep("b_grouplocal", names(all_samples), value = TRUE)
  if (length(param_name) == 0) {
    param_name <- grep("b_group", names(all_samples), value = TRUE)[1]
  }

  if (length(param_name) == 0 || is.na(param_name)) {
    warning("Group effect parameter not found in model: ", model_list[[1]]$model_name)
    return(list(
      diff_mean = NA,
      diff_ci = c(NA, NA),
      prob_diff_positive = NA,
      non_inf_mean = NA,
      non_inf_ci = c(NA, NA),
      prob_non_inferior = NA
    ))
  }
```

```
  # Extract intercept and group effect
  intercept <- all_samples$b_Intercept
  group_effect <- all_samples[[param_name]]

  # Calculate probabilities
  p_telesurgery <- plogis(intercept)
  p_local <- plogis(intercept + group_effect)

  # Calculate rate differences
  prob_diff <- p_telesurgery - p_local
  non_inferiority_diff <- p_telesurgery - (p_local + non_inferiority_margin)

  # Calculate statistics
  diff_mean <- mean(prob_diff)
  diff_ci <- quantile(prob_diff, c(0.025, 0.975))
  prob_diff_positive <- mean(prob_diff > 0)

  # Non-inferiority statistics
  non_inf_mean <- mean(non_inferiority_diff)
  non_inf_ci <- quantile(non_inferiority_diff, c(0.025, 0.975))
  prob_non_inferior <- mean(non_inferiority_diff > 0)

  return(list(
    diff_mean = diff_mean,
    diff_ci = diff_ci,
    prob_diff_positive = prob_diff_positive,
    non_inf_mean = non_inf_mean,
    non_inf_ci = non_inf_ci,
    prob_non_inferior = prob_non_inferior
  ))
}

# Analyze results for each model (using Bayesian pooling)
final_results <- list()
total_fit_times <- list()

for (model_name in names(models)) {
  cat("\nProcessing model:", models[[model_name]]$name, "\n")

  # Extract results for this model across all imputed datasets
  model_list <- map(all_models, ~ .x[[model_name]])

  # Calculate total fitting time
  total_time <- sum(sapply(model_list, function(x) x$fit_time))
```

```
  total_fit_times[[model_name]] <- total_time
  cat("  Total fitting time:", round(total_time, 1), "minutes\n")

  # Compute Bayesian-pooled rate difference
  bayes_results <- compute_rate_difference_bayes(model_list)

  # Store results
  final_results[[model_name]] <- list(
    model_name = models[[model_name]]$name,
    description = models[[model_name]]$description,
    is_random = models[[model_name]]$random,
    rate_diff = bayes_results$diff_mean,
    rate_diff_ci = bayes_results$diff_ci,
    prob_diff_positive = bayes_results$prob_diff_positive,
    prob_non_inferior = bayes_results$prob_non_inferior,
    total_fit_time = total_time
  )
}

# Create results table
results_table <- map_dfr(final_results, function(res) {
  data.frame(
    Model = res$model_name,
    Covariates = res$description,
    Random_Effects = ifelse(res$is_random, "Yes", "No"),
    Rate_Diff = res$rate_diff,
    Rate_Diff_CI_low = res$rate_diff_ci[1],
    Rate_Diff_CI_high = res$rate_diff_ci[2],
    Prob_Rate_Diff_Positive = res$prob_diff_positive,
    Non_Inferior_Prob = res$prob_non_inferior,
    Non_Inferior = ifelse(res$prob_non_inferior > 0.975, "Yes", "No"),
    Total_Fit_Time_Min = round(res$total_fit_time, 1),
    stringsAsFactors = FALSE
  )
})

# Print results
cat("\n===== FINAL RESULTS (Bayesian Pooling with GPU Acceleration) =====\n")
print(results_table)

# Save results
write.csv(results_table, "gpu_accelerated_non_inferiority_results.csv", row.names = FALSE)

# Visualization: Forest plot of rate differences (including non-inferiority region)
```

```r
rd_plot <- ggplot(results_table, aes(x = Rate_Diff, y = Model)) +
  geom_point(aes(color = Non_Inferior), size = 3) +
  geom_errorbarh(aes(xmin = Rate_Diff_CI_low, xmax = Rate_Diff_CI_high,
                     color = Non_Inferior), height = 0.1, linewidth = 0.7) +
  geom_vline(xintercept = 0, linetype = "dashed", color = "black") +
  geom_vline(xintercept = NON_INFERIORITY_MARGIN, linetype = "dashed", color = "red",
linewidth = 1) +
  annotate("rect", xmin = NON_INFERIORITY_MARGIN, xmax =
max(results_table$Rate_Diff_CI_high, na.rm = TRUE) + 0.05,
          ymin = -Inf, ymax = Inf, alpha = 0.1, fill = "green") +
  scale_color_manual(values = c("Yes" = "blue", "No" = "gray50")) +
  labs(x = "Success Rate Difference (Telesurgery - Local)",
       y = "Adjustment Model",
       title = paste("GPU-Accelerated Non-inferiority Analysis (Margin =",
NON_INFERIORITY_MARGIN, ")"),
       subtitle = paste("GPU Acceleration:", ifelse(use_gpu, "Enabled", "Disabled")),
       color = "Non-Inferior",
       caption = paste("Non-inferiority threshold:", NON_INFERIORITY_MARGIN,
                      "\nNon-inferiority concluded if posterior probability > 97.5%")) +
  theme_minimal() +
  theme(
    plot.title = element_text(hjust = 0.5, face = "bold", size = 14),
    plot.subtitle = element_text(hjust = 0.5),
    axis.title = element_text(size = 12),
    axis.text = element_text(size = 10),
    legend.position = "bottom"
  ) +
  geom_text(aes(label = sprintf("Prob(NI): %.3f\nTime: %.1f min", Non_Inferior_Prob,
Total_Fit_Time_Min),
              x = max(results_table$Rate_Diff_CI_high, na.rm = TRUE) + 0.03),
            hjust = 0, size = 3.5, color = "darkblue")

# Save and display plot
ggsave("gpu_accelerated_non_inferiority_plot.png", rd_plot, width = 11, height = 7, dpi =
300)
print(rd_plot)

# Print final message
cat("\nGPU-accelerated analysis completed successfully!\n")
cat("Results saved to:\n")
cat("- gpu_accelerated_non_inferiority_results.csv\n")
cat("- gpu_accelerated_non_inferiority_plot.png\n")

# Print total computation time
```

```
total_time <- sum(results_table$Total_Fit_Time_Min)
cat("\nTotal computation time:", round(total_time, 1), "minutes\n")
if (use_gpu) {
  cat("GPU acceleration reduced computation time significantly.\n")
} else {
  cat("Consider enabling GPU acceleration for faster computation.\n")
}
```

2. Bayesian mixed-effects logistic regression with penalized priors for PPS of the primary outcome (The R Programming Language)

```
library(brms)
library(dplyr)
library(tibble)
library(posterior)
library(bayestestR)
library(ggplot2)
library(tidyr)
library(purrr)
library(ggpubr)

# Set non-inferiority margin
NON_INFERIORITY_MARGIN <- -0.1

# 1. Build dataset
dat_text <- "
group hospital  surgery_type surgeon success
1  4  1   1   1
1  4  1   2   1
2  4  1   3   1
2  4  1   3   1
2  1  2   4   1
2  1  2   4   1
1  1  2   5   1
1  1  1   5   1
2  2  2   5   1
2  2  2   5   1
1  2  2   4   1
1  2  2   4   1
2  1  1   6   1
```

1	1	2	3	1
1	1	1	3	1
2	1	1	6	1
2	4	2	3	1
2	4	2	7	1
1	4	1	8	1
2	4	1	3	1
2	1	1	1	1
2	2	1	5	1
1	2	1	8	1
1	2	1	4	1
2	1	1	6	1
1	4	2	4	1
1	1	2	3	1
1	1	1	9	1
1	3	2	4	1
1	1	2	9	1
1	1	1	9	1
1	3	1	8	1
2	3	1	9	1
2	3	2	9	1
1	1	1	9	1
2	1	2	4	1
2	3	1	9	1
1	3	1	8	1
2	3	1	9	1
2	3	1	9	1
1	3	2	4	1
1	3	2	4	1
2	3	2	9	1
1	5	2	4	1
1	5	1	8	1
2	3	2	9	1
2	3	2	9	1
1	3	2	4	1
2	1	1	6	1
2	5	2	10	0
2	5	2	10	1
2	1	2	10	1
1	5	1	8	1
1	5	2	4	1
2	1	2	4	1
1	1	1	6	1
1	1	2	10	1

```
2  1  1   2   1
1  1  2  10   1
1  5  1   4   1
1  5  1   8   1
2  5  1  11   1
2  5  1   6   1"

# 2. Read data
sensitive_data <- read.table(text = data_text, header = TRUE)

# 3. Convert variable types
sensitive_data <- sensitive_data %>%
  mutate(
    group = factor(group, levels = c(1, 2), labels = c("telesurgery", "local")),
    hospital = factor(hospital, levels = c(1, 2, 3, 4, 5),
                      labels = c("Beijing", "Harbin", "Hangzhou", "Hefei", "Urumqi")),
    surgery_type = factor(surgery_type, levels = c(1, 2),
                          labels = c("RadicalProstatectomy", "PartialNephrectomy")),
    surgeon = factor(surgeon),
    sensitive = as.integer(success)
  )

# 4. Configure GPU acceleration
configure_gpu_acceleration <- function() {
  # Check if cmdstanr is installed
  if (!requireNamespace("cmdstanr", quietly = TRUE)) {
    message("cmdstanr not installed. Installing now...")
    install.packages("cmdstanr", repos = c("https://mc-stan.org/r-packages/", 
getOption("repos")))
  }

  # Check if CUDA is available
  cuda_available <- tryCatch({
    cmdstanr::install_cmdstan(cores = parallel::detectCores(), overwrite = FALSE, cpp_options 
= list("CUDA" = TRUE))
    TRUE
  }, error = function(e) FALSE)

  if (cuda_available) {
    message("CUDA detected. GPU acceleration will be used.")
    return(list(use_gpu = TRUE, backend = "cmdstanr"))
  } else {
    message("CUDA not available. Using CPU only.")
    return(list(use_gpu = FALSE, backend = "rstan"))
```

```
  }
}

# Configure GPU acceleration
gpu_config <- configure_gpu_acceleration()
use_gpu <- gpu_config$use_gpu
backend <- gpu_config$backend

# 5. Dynamic prior function (with GPU-related parameters)
get_prior_setting <- function(formula) {
  # Basic priors: Fixed effects
  priors <- c(
    prior(normal(0, 5), class = "b", coef = "grouplocal"),
    prior(normal(0, 5), class = "b"),
    prior(normal(0, 5), class = "Intercept")
  )

  # If formula contains random effects, add sd prior
  if (any(grepl("\\|", deparse(formula)))) {
    priors <- c(priors, prior(cauchy(0, 2), class = "sd"))
  }

  return(priors)
}

# 6. Define hierarchical adjustment models (surgeon as random effect)
models <- list(
  unadjusted = list(
    name = "Unadjusted",
    formula = sensitive ~ group,
    description = "No covariates"
  ),
  adjust_surgeon = list(
    name = "Adjusted for Surgeon (RE)",
    formula = sensitive ~ group + (1 | surgeon),  # Random intercept model
    description = "Surgeon as random effect"
  ),
  adjust_hospital = list(
    name = "Adjusted for Hospital",
    formula = sensitive ~ group + hospital,
    description = "Hospital as covariate"
  ),
  adjust_surgery_type = list(
    name = "Adjusted for Surgery Type",
```

```
    formula = sensitive ~ group + surgery_type,
    description = "Surgery type as covariate"
  ),
  adjust_all = list(
    name = "Adjusted for All",
    # Note: Surgeon as random effect, others as fixed effects
    formula = sensitive ~ group + hospital + surgery_type + (1 | surgeon),
    description = "Hospital and type fixed, surgeon random"
  )
)

# 7. Fit all models (with error handling and GPU acceleration)
fit_models <- function(model_list, data) {
  results <- list()

  for (i in seq_along(model_list)) {
    model_name <- names(model_list)[i]
    model_spec <- model_list[[i]]

    cat("\n===== Fitting model:", model_spec$name, "=====\n")

    # Dynamically get priors for this model
    model_prior <- get_prior_setting(model_spec$formula)

    # Configure GPU acceleration parameters
    stan_model_args <- NULL
    if (use_gpu) {
      stan_model_args <- list(
        cpp_options = list(
          STAN_OPENCL = TRUE,
          OPENCL_DEVICE_ID = 0,
          OPENCL_PLATFORM_ID = 0
        )
      )
    }

    tryCatch({
      fit <- brm(
        formula = model_spec$formula,
        data = data,
        family = bernoulli("logit"),
        prior = model_prior,
        chains = 4,
        iter = 5000,
```

```
  warmup = 2000,
  seed = 20250802 + i,  # Changed seed to 20250802
  control = list(adapt_delta = 0.99, max_treedepth = 15),
  refresh = 0,
  silent = TRUE,
  backend = backend,
  stan_model_args = stan_model_args
)

# Extract group effect
post_samples <- as_draws_df(fit)

# Find group effect parameter
param_name <- grep("b_grouplocal", names(post_samples), value = TRUE)
if (length(param_name) == 0) {
  # If parameter name is different, try other common names
  possible_names <- c("b_grouplocal", "b_group2", "b_grouplocal")
  found <- FALSE
  for (pname in possible_names) {
    if (pname %in% names(post_samples)) {
      param_name <- pname
      found <- TRUE
      break
    }
  }
  if (!found) stop("Group effect parameter not found in model: ", model_spec$name)
}

group_effect <- post_samples[[param_name]]

# Calculate group difference statistics
log_or <- mean(group_effect)
log_or_ci <- quantile(group_effect, c(0.025, 0.975))
or <- exp(log_or)
or_ci <- exp(log_or_ci)

# Calculate Bayesian p-values
p_one_sided <- mean(group_effect > 0)
p_two_sided <- 2 * min(p_one_sided, 1 - p_one_sided)
p_two_sided <- min(p_two_sided, 1)

# Calculate success rate difference (relative to non-inferiority margin)
intercept_samples <- post_samples$b_Intercept
p_telesurgery <- plogis(intercept_samples)
```

```
    p_local <- plogis(intercept_samples + group_effect)
    prob_diff <- p_telesurgery - p_local
    mean_diff <- mean(prob_diff)
    diff_ci <- quantile(prob_diff, c(0.025, 0.975))

    # Calculate non-inferiority posterior probability
    prob_non_inferior <- mean(prob_diff > NON_INFERIORITY_MARGIN)

    # Save results
    results[[model_name]] <- list(
      fit = fit,
      model_name = model_spec$name,
      description = model_spec$description,
      log_or = log_or,
      log_or_ci = log_or_ci,
      or = or,
      or_ci = or_ci,
      p_value = p_two_sided,
      mean_diff = mean_diff,
      diff_ci = diff_ci,
      prob_non_inferior = prob_non_inferior,
      rhat = rhat(fit)[[param_name]]
    )

    cat("Successfully completed:", model_spec$name, "\n")
  }, error = function(e) {
    cat("!!! Error fitting model:", model_spec$name, "!!!\n")
    cat("Error message:", e$message, "\n")

    # Save error information
    results[[model_name]] <<- list(
      error = TRUE,
      error_message = e$message,
      model_name = model_spec$name,
      description = model_spec$description
    )
  })
 }

 return(results)
}

# 8. Fit all models (with error handling and GPU acceleration)
model_results <- fit_models(models, sensitive_data)
```

```
# 9. Create results summary table (handling possible errors)
create_results_table <- function(results) {
  res_table <- data.frame(
    Model = character(),
    Covariates = character(),
    Log_OR = numeric(),
    OR = numeric(),
    OR_CI_low = numeric(),
    OR_CI_high = numeric(),
    P_value = numeric(),
    Success_Diff = numeric(),
    Diff_CI_low = numeric(),
    Diff_CI_high = numeric(),
    Prob_Non_Inferior = numeric(),
    Rhat = numeric(),
    stringsAsFactors = FALSE
  )

  for (model_name in names(results)) {
    res <- results[[model_name]]

    # Skip failed models
    if (!is.null(res$error)) {
      cat("Skipping model due to error:", res$model_name, "\n")
      next
    }

    new_row <- data.frame(
      Model = res$model_name,
      Covariates = res$description,
      Log_OR = res$log_or,
      OR = res$or,
      OR_CI_low = res$or_ci[1],
      OR_CI_high = res$or_ci[2],
      P_value = res$p_value,
      Success_Diff = res$mean_diff,
      Diff_CI_low = res$diff_ci[1],
      Diff_CI_high = res$diff_ci[2],
      Prob_Non_Inferior = res$prob_non_inferior,
      Rhat = res$rhat,
      stringsAsFactors = FALSE
    )
```

```
    res_table <- rbind(res_table, new_row)
  }

  return(res_table)
}

# 10. Create results summary table
results_table <- create_results_table(model_results)

# 11. Visualize non-inferiority results
if (nrow(results_table) > 0) {
  # Create forest plot
  forest_plot <- ggplot(results_table, aes(x = Success_Diff, y = Model)) +
    geom_point(aes(color = Prob_Non_Inferior > 0.975), size = 3) +
    geom_errorbarh(aes(xmin = Diff_CI_low, xmax = Diff_CI_high,
                   color = Prob_Non_Inferior > 0.975), height = 0.1) +
    geom_vline(xintercept = 0, linetype = "dashed", color = "black") +
    geom_vline(xintercept = NON_INFERIORITY_MARGIN, linetype = "dashed", color = "red")
+
    annotate("rect", xmin = NON_INFERIORITY_MARGIN, xmax =
max(results_table$Diff_CI_high, na.rm = TRUE) + 0.05,
             ymin = -Inf, ymax = Inf, alpha = 0.1, fill = "green") +
    scale_color_manual(values = c("FALSE" = "gray", "TRUE" = "blue"),
                       name = "Non-inferior",
                       labels = c("No", "Yes")) +
    labs(x = "Success Rate Difference (Telesurgery - Local)",
         y = "Adjustment Model",
         title = paste("Non-inferiority Analysis (Margin =", NON_INFERIORITY_MARGIN, ")"),
         subtitle = paste("GPU Acceleration:", ifelse(use_gpu, "Enabled", "Disabled")),
         caption = "Non-inferiority concluded if posterior probability > 97.5%") +
    theme_minimal() +
    theme(plot.title = element_text(hjust = 0.5, face = "bold"),
          plot.subtitle = element_text(hjust = 0.5))

  # Add non-inferiority probability labels
  forest_plot <- forest_plot +
    geom_text(aes(label = sprintf("P(NI): %.3f", Prob_Non_Inferior),
                  x = max(results_table$Diff_CI_high, na.rm = TRUE) + 0.03),
              hjust = 0, size = 3.5, color = "darkblue")

  # Print and save plot
  print(forest_plot)
  ggsave("non_inferiority_forest_plot.png", forest_plot, width = 10, height = 6, dpi = 300)
```

```
  # Print results table
  cat("\n===== FINAL RESULTS (Non-inferiority Analysis) =====\n")
  print(results_table)

  # Save results
  write.csv(results_table, "non_inferiority_results.csv", row.names = FALSE)
} else {
  cat("No models successfully fitted. Cannot create results table or visualizations.\n")
}

# 12. Output detailed results (only successfully fitted models)
cat("\n===== Detailed Results =====\n")
for (model_name in names(model_results)) {
  res <- model_results[[model_name]]

  if (!is.null(res$error)) {
    cat("\n---", res$model_name, "--- [ERROR]\n")
    cat("Error message:", res$error_message, "\n")
    next
  }

  cat("\n---", res$model_name, "---\n")
  cat("Covariates:", res$description, "\n")
  cat("Log OR:", round(res$log_or, 3), "\n")
  cat("OR:", round(res$or, 3),
      "95% CI: [", round(res$or_ci[1], 3), ",", round(res$or_ci[2], 3), "]\n")
  cat("P-value:", round(res$p_value, 4), "\n")
  cat("Success Rate Difference:", round(res$mean_diff, 3),
      "95% CI: [", round(res$diff_ci[1], 3), ",", round(res$diff_ci[2], 3), "]\n")
  cat("Non-inferiority Probability (P >", NON_INFERIORITY_MARGIN, "):",
      round(res$prob_non_inferior, 3), "\n")
  cat("R-hat:", round(res$rhat, 3), "\n")
}

# 13. Covariate effect analysis
analyze_covariate_effects <- function(model) {
  post_samples <- as_draws_df(model$fit)

  # Identify covariate parameters
  cov_params <- setdiff(
    grep("^b_", colnames(post_samples), value = TRUE),
    c("b_Intercept", "b_grouplocal")
  )
```

```
  # Identify random effect standard deviations
  sd_params <- grep("^sd_", colnames(post_samples), value = TRUE)

  all_params <- c(cov_params, sd_params)

  if (length(all_params) == 0) {
    return(data.frame())
  }

  effects <- map_dfr(all_params, function(param) {
    samples <- post_samples[[param]]

    data.frame(
      Parameter = param,
      Mean = mean(samples),
      CI_low = quantile(samples, 0.025),
      CI_high = quantile(samples, 0.975),
      P_direction = p_direction(samples),
      stringsAsFactors = FALSE
    )
  })

  effects$Model <- model$model_name
  return(effects)
}

# Analyze covariate effects across all models
covariate_effects <- map_dfr(model_results, analyze_covariate_effects, .id = "model_id")

# Output covariate effects
if (nrow(covariate_effects) > 0) {
  cat("\n===== Covariate Effects Across Models =====\n")
  print(covariate_effects)

  # Visualize covariate effects
  cov_plot <- ggplot(covariate_effects, aes(x = Mean, y = Parameter)) +
    geom_point() +
    geom_errorbarh(aes(xmin = CI_low, xmax = CI_high), height = 0.2) +
    geom_vline(xintercept = 0, linetype = "dashed", color = "red") +
    facet_wrap(~ Model, scales = "free_y") +
    labs(x = "Effect Size (Log Odds) / Standard Deviation",
         y = "Covariate/Random Effect",
         title = "Covariate and Random Effects Across Models") +
    theme_minimal() +
```

```
    theme(plot.title = element_text(hjust = 0.5))

  ggsave("covariate_effects.png", cov_plot, width = 12, height = 8, dpi = 300)
  print(cov_plot)
}

# 14. Save all results
saveRDS(model_results, "non_inferiority_model_results.rds")
write.csv(results_table, "non_inferiority_model_results.csv", row.names = FALSE)
```

3. Mixed-effects Linear regression analysis

3.1 Operative time

```
/* Data Input */
/* Data details:
   group: 1=telesurgery group, 2=local surgery group
   hospital: 1=Beijing, 2=Harbin, 3=Hangzhou, 4=Hefei, 5=Urumqi
   surgery_type: 1=Radical Prostatectomy, 2=Partial Nephrectomy
   surgeon codes: 1=Chaozhao Liang, 2=Baojun Wang, 3=Xu Zhang,
4=Sheng Tai,
                  5=Hongzhao Li, 6=Wanghai Xu, 7=Xin Ma, 8=Qingbo
Huang,
                  9=Weijun Fu, 10=Shuo Wang, 11=Mulati Rexiati
*/
data sensitive_data;
input group hospital surgery_type surgeon sensitive;
datalines;
2   3   1   10  141
1   3   1   5   172
2   3   1   10  115
2   3   1   10  210
1   3   1   5   153
2   3   1   10  135
1   3   2   7   125
2   3   2   10  95
2   3   2   10  94
1   3   2   7   94
2   3   2   10  112
1   3   2   7   83
2   3   2   10  85
```

1	3	2	7	83
1	4	1	2	120
2	4	1	4	320
2	4	1	4	225
1	4	1	3	110
1	4	1	5	240
2	4	1	1	260
2	4	2	1	90
2	4	2	1	140
1	4	2	7	75
1	5	1	5	180
1	5	1	5	195
1	5	1	5	235
1	5	1	2	250
2	5	1	2	205
2	5	1	11	320
1	5	2	7	100
2	5	2	8	80
2	5	2	8	150
1	5	2	8	115
2	1	1	9	240
1	1	1	6	180
2	1	1	9	180
1	1	1	1	200
2	1	1	3	157
2	1	1	9	250
1	1	1	10	150
1	1	1	10	120
1	1	1	10	180
2	1	1	9	180
1	1	1	9	180
2	1	1	9	115
2	1	2	7	60
2	1	2	7	75
1	1	2	6	240
1	1	2	1	140
1	1	2	1	60
1	1	2	10	160
2	1	2	7	90
2	1	2	7	120
1	1	2	8	70
2	1	2	7	60
1	1	2	8	60
1	2	1	5	165

```
2   2   1   6   250
1   2   1   7   170
2   2   2   6   100
2   2   2   6   120
1   2   2   7   70
1   2   2   8   170
;
run;


/* 2. Main Analysis: Mixed Model (Surgeon as Random Effect) */
proc mixed data=sensitive_data method=reml noclprint;
  class group surgeon;
  model sensitive = group / solution ddfm=satterthwaite;
  random surgeon;
  lsmeans group / cl pdiff;
  ods output LSMeans=lsmeans_mixed CovParms=covparms;
  title "Main Analysis: Mixed Effects Model with Surgeon as Random";
run;

/* Extract Residual Standard Deviation */
data _null_;
  set covparms;
  if CovParm="Residual" then call symputx("resid_sd_mixed",
sqrt(Estimate));
run;

/* Calculate Cohen's d */
data cohens_d_mixed;
  length label $50;
  set lsmeans_mixed(where=(group in (1,2)) rename=(Estimate=adj_mean
StdErr=adj_se));
  retain mean1 mean2;
  if group=1 then mean1=adj_mean;
  else if group=2 then mean2=adj_mean;
  if _n_=2 then do;
    s_pooled = &resid_sd_mixed;
    cohens_d = (mean1 - mean2) / s_pooled;
    label = "Main Analysis (Mixed Model)";
    output;
  end;
  keep label cohens_d mean1 mean2 s_pooled;
run;
```

```
proc print data=cohens_d_mixed noobs label;
  title "Cohen's d from Main Analysis (Mixed Effects Model)";
run;


/* 3. Sensitivity Analysis 1: Adjusting Hospital (Mixed Model) */
proc mixed data=sensitive_data method=reml noclprint;
  class group surgeon hospital;
  model sensitive = group hospital / solution ddfm=satterthwaite;
  random surgeon;
  lsmeans group / cl pdiff;
  ods output LSMeans=lsmeans_hospital CovParms=covparms_hosp;
  title "Sensitivity Analysis 1: Mixed Model Adjusting Hospital";
run;

/* Extract Residual Standard Deviation */
data _null_;
  set covparms_hosp;
  if CovParm="Residual" then call symputx("resid_sd_hosp",
sqrt(Estimate));
run;

/* Calculate Cohen's d */
data cohens_d_hosp;
  length label $50;
  set lsmeans_hospital(where=(group in (1,2))
rename=(Estimate=adj_mean StdErr=adj_se));
  retain mean1 mean2;
  if group=1 then mean1=adj_mean;
  else if group=2 then mean2=adj_mean;
  if _n_=2 then do;
    s_pooled = &resid_sd_hosp;
    cohens_d = (mean1 - mean2) / s_pooled;
    label = "Sensitivity Analysis Adjusting Hospital";
    output;
  end;
  keep label cohens_d mean1 mean2 s_pooled;
run;

proc print data=cohens_d_hosp noobs label;
  title "Cohen's d from Sensitivity Analysis Adjusting Hospital";
run;
```

```
/* 4. Sensitivity Analysis 2: Adjusting Surgery Type (Mixed Model) */
proc mixed data=sensitive_data method=reml noclprint;
  class group surgeon surgery_type;
  model sensitive = group surgery_type / solution ddfm=satterthwaite;
  random surgeon;
  lsmeans group / cl pdiff;
  ods output LSMeans=lsmeans_surgtype CovParms=covparms_surgtype;
  title "Sensitivity Analysis 2: Mixed Model Adjusting Surgery Type";
run;

/* Extract Residual Standard Deviation */
data _null_;
  set covparms_surgtype;
  if CovParm="Residual" then call symputx("resid_sd_surgtype",
sqrt(Estimate));
run;

/* Calculate Cohen's d */
data cohens_d_surgtype;
  length label $50;
  set lsmeans_surgtype(where=(group in (1,2))
rename=(Estimate=adj_mean StdErr=adj_se));
  retain mean1 mean2;
  if group=1 then mean1=adj_mean;
  else if group=2 then mean2=adj_mean;
  if _n_=2 then do;
    s_pooled = &resid_sd_surgtype;
    cohens_d = (mean1 - mean2) / s_pooled;
    label = "Sensitivity Analysis Adjusting Surgery Type";
    output;
  end;
  keep label cohens_d mean1 mean2 s_pooled;
run;

proc print data=cohens_d_surgtype noobs label;
  title "Cohen's d from Sensitivity Analysis Adjusting Surgery Type";
run;


/* 5. Sensitivity Analysis 3: Adjusting Surgeon (fixed effect) */
proc mixed data=sensitive_data method=reml noclprint;
  class group surgeon;
  model sensitive = group surgeon / solution ddfm=satterthwaite;
  lsmeans group / cl pdiff;
```

```
  ods output LSMeans=lsmeans_surg_fixed CovParms=covparms_surg_fixed;
  title "Sensitivity Analysis 3: Mixed Model Adjusting Surgeon (as
fixed effect)";
run;

/* Extract Residual Standard Deviation */
data _null_;
  set covparms_surg_fixed;
  if CovParm="Residual" then call symputx("resid_sd_surg_fixed",
sqrt(Estimate));
run;

/* Calculate Cohen's d */
data cohens_d_surg_fixed;
  length label $50;
  set lsmeans_surg_fixed(where=(group in (1,2))
rename=(Estimate=adj_mean StdErr=adj_se));
  retain mean1 mean2;
  if group=1 then mean1=adj_mean;
  else if group=2 then mean2=adj_mean;
  if _n_=2 then do;
    s_pooled = &resid_sd_surg_fixed;
    cohens_d = (mean1 - mean2) / s_pooled;
    label = "Sensitivity Analysis Adjusting Surgeon (fixed effect)";
    output;
  end;
  keep label cohens_d mean1 mean2 s_pooled;
run;

proc print data=cohens_d_surg_fixed noobs label;
  title "Cohen's d from Sensitivity Analysis Adjusting Surgeon
(fixed)";
run;
```

3.2 Warm ischemia time

```
/* Data Input */
/* Data details:
   group: 1=telesurgery group, 2=local surgery group
   hospital: 1=Beijing, 2=Harbin, 3=Hangzhou, 4=Hefei, 5=Urumqi
   surgeon codes: 1=Chaozhao Liang, 2=Baojun Wang, 3=Xu Zhang,
4=Sheng Tai,
                  5=Hongzhao Li, 6=Wanghai Xu, 7=Xin Ma, 8=Qingbo
Huang,
```

```
                   9=Weijun Fu, 10=Shuo Wang, 11=Mulati Rexiati
*/
data sensitive_data;
input group hospital surgeon sensitive;
datalines;
1   3   7   26
2   3   10  20
2   3   10  18
1   3   7   0
2   3   10  28
1   3   7   16
2   3   10  18
1   3   7   15
2   4   1   29
2   4   1   22
1   4   7   0
1   5   7   11
2   5   8   14
2   5   8   29
1   5   8   15
2   1   7   15
2   1   7   0
1   1   6   15
1   1   1   17
1   1   1   30
1   1   10  24
2   1   7   22
2   1   7   20
1   1   8   18
2   1   7   23
1   1   8   29
2   2   6   20
2   2   6   15
1   2   7   15
1   2   8   20
;
run;


/* 2. Main Analysis: Mixed Model (Surgeon as Random Effect) */
proc mixed data=sensitive_data method=reml noclprint;
  class group surgeon;
  model sensitive = group / solution ddfm=satterthwaite;
  random surgeon;
```

```
  lsmeans group / cl pdiff;
  ods output LSMeans=lsmeans_mixed CovParms=covparms;
  title "Main Analysis: Mixed Effects Model with Surgeon as Random";
run;

/* Extract Residual Standard Deviation */
data _null_;
  set covparms;
  if CovParm="Residual" then call symputx("resid_sd_mixed",
sqrt(Estimate));
run;

/* Calculate Cohen's d */
data cohens_d_mixed;
  length label $50;
  set lsmeans_mixed(where=(group in (1,2)) rename=(Estimate=adj_mean
StdErr=adj_se));
  retain mean1 mean2;
  if group=1 then mean1=adj_mean;
  else if group=2 then mean2=adj_mean;
  if _n_=2 then do;
    s_pooled = &resid_sd_mixed;
    cohens_d = (mean1 - mean2) / s_pooled;
    label = "Main Analysis (Mixed Model)";
    output;
  end;
  keep label cohens_d mean1 mean2 s_pooled;
run;

proc print data=cohens_d_mixed noobs label;
  title "Cohen's d from Main Analysis (Mixed Effects Model)";
run;


/* 3. Sensitivity Analysis 1: Adjusting Hospital (Mixed Model) */
proc mixed data=sensitive_data method=reml noclprint;
  class group surgeon hospital;
  model sensitive = group hospital / solution ddfm=satterthwaite;
  random surgeon;
  lsmeans group / cl pdiff;
  ods output LSMeans=lsmeans_hospital CovParms=covparms_hosp;
  title "Sensitivity Analysis 1: Mixed Model Adjusting Hospital";
run;
```

```
/* Extract Residual Standard Deviation */
data _null_;
  set covparms_hosp;
  if CovParm="Residual" then call symputx("resid_sd_hosp",
sqrt(Estimate));
run;

/* Calculate Cohen's d */
data cohens_d_hosp;
  length label $50;
  set lsmeans_hospital(where=(group in (1,2))
rename=(Estimate=adj_mean StdErr=adj_se));
  retain mean1 mean2;
  if group=1 then mean1=adj_mean;
  else if group=2 then mean2=adj_mean;
  if _n_=2 then do;
    s_pooled = &resid_sd_hosp;
    cohens_d = (mean1 - mean2) / s_pooled;
    label = "Sensitivity Analysis Adjusting Hospital";
    output;
  end;
  keep label cohens_d mean1 mean2 s_pooled;
run;

proc print data=cohens_d_hosp noobs label;
  title "Cohen's d from Sensitivity Analysis Adjusting Hospital";
run;


/* 4. Sensitivity Analysis 3: Adjusting Surgeon (fixed effect) */
proc mixed data=sensitive_data method=reml noclprint;
  class group surgeon;
  model sensitive = group surgeon / solution ddfm=satterthwaite;
  lsmeans group / cl pdiff;
  ods output LSMeans=lsmeans_surg_fixed CovParms=covparms_surg_fixed;
  title "Sensitivity Analysis 3: Mixed Model Adjusting Surgeon (as
fixed effect)";
run;

/* Extract Residual Standard Deviation */
data _null_;
  set covparms_surg_fixed;
  if CovParm="Residual" then call symputx("resid_sd_surg_fixed",
sqrt(Estimate));
```

```
run;

/* Calculate Cohen's d */
data cohens_d_surg_fixed;
  length label $50;
  set lsmeans_surg_fixed(where=(group in (1,2))
rename=(Estimate=adj_mean StdErr=adj_se));
  retain mean1 mean2;
  if group=1 then mean1=adj_mean;
  else if group=2 then mean2=adj_mean;
  if _n_=2 then do;
    s_pooled = &resid_sd_surg_fixed;
    cohens_d = (mean1 - mean2) / s_pooled;
    label = "Sensitivity Analysis Adjusting Surgeon (fixed effect)";
    output;
  end;
  keep label cohens_d mean1 mean2 s_pooled;
run;

proc print data=cohens_d_surg_fixed noobs label;
  title "Cohen's d from Sensitivity Analysis Adjusting Surgeon
(fixed)";
run;
```

3.3 Blood loss

```
/* Data Input */
/* Data details:
   group: 1=telesurgery group, 2=local surgery group
   hospital: 1=Beijing, 2=Harbin, 3=Hangzhou, 4=Hefei, 5=Urumqi
   surgery_type: 1=Radical Prostatectomy, 2=Partial Nephrectomy
   surgeon codes: 1=Chaozhao Liang, 2=Baojun Wang, 3=Xu Zhang,
4=Sheng Tai,
                  5=Hongzhao Li, 6=Wanghai Xu, 7=Xin Ma, 8=Qingbo
Huang,
                  9=Weijun Fu, 10=Shuo Wang, 11=Mulati Rexiati
*/
data sensitive_data;
input group hospital surgery_type surgeon sensitive;
datalines;
2   3   1   10  50
1   3   1   5   50
2   3   1   10  50
2   3   1   10  50
```

1	3	1	5	50
2	3	1	10	80
1	3	2	7	50
2	3	2	10	50
2	3	2	10	20
1	3	2	7	20
2	3	2	10	20
1	3	2	7	30
2	3	2	10	50
1	3	2	7	50
1	4	1	2	50
2	4	1	4	100
2	4	1	4	150
1	4	1	3	20
1	4	1	5	250
2	4	1	1	100
2	4	2	1	50
2	4	2	1	50
1	4	2	7	20
1	5	1	5	100
1	5	1	5	50
1	5	1	5	200
1	5	1	2	100
2	5	1	2	200
2	5	1	11	100
1	5	2	7	50
2	5	2	8	10
2	5	2	8	400
1	5	2	8	20
2	1	1	9	50
1	1	1	6	200
2	1	1	9	70
1	1	1	1	50
2	1	1	3	59
2	1	1	9	100
1	1	1	10	50
1	1	1	10	100
1	1	1	10	400
2	1	1	9	100
1	1	1	9	30
2	1	1	9	50
2	1	2	7	20
2	1	2	7	50
1	1	2	6	50

```
1   1   2   1   50
1   1   2   1   50
1   1   2   10  100
2   1   2   7   100
2   1   2   7   100
1   1   2   8   30
2   1   2   7
1   1   2   8   50
1   2   1   5   50
2   2   1   6   100
1   2   1   7   50
2   2   2   6   50
2   2   2   6   50
1   2   2   7   50
1   2   2   8   200
;
run;


/* 2. Main Analysis: Mixed Model (Surgeon as Random Effect) */
proc mixed data=sensitive_data method=reml noclprint;
  class group surgeon;
  model sensitive = group / solution ddfm=satterthwaite;
  random surgeon;
  lsmeans group / cl pdiff;
  ods output LSMeans=lsmeans_mixed CovParms=covparms;
  title "Main Analysis: Mixed Effects Model with Surgeon as Random";
run;

/* Extract Residual Standard Deviation */
data _null_;
  set covparms;
  if CovParm="Residual" then call symputx("resid_sd_mixed",
sqrt(Estimate));
run;

/* Calculate Cohen's d */
data cohens_d_mixed;
  length label $50;
  set lsmeans_mixed(where=(group in (1,2)) rename=(Estimate=adj_mean
StdErr=adj_se));
  retain mean1 mean2;
  if group=1 then mean1=adj_mean;
  else if group=2 then mean2=adj_mean;
```

```
  if _n_=2 then do;
    s_pooled = &resid_sd_mixed;
    cohens_d = (mean1 - mean2) / s_pooled;
    label = "Main Analysis (Mixed Model)";
    output;
  end;
  keep label cohens_d mean1 mean2 s_pooled;
run;

proc print data=cohens_d_mixed noobs label;
  title "Cohen's d from Main Analysis (Mixed Effects Model)";
run;


/* 3. Sensitivity Analysis 1: Adjusting Hospital (Mixed Model) */
proc mixed data=sensitive_data method=reml noclprint;
  class group surgeon hospital;
  model sensitive = group hospital / solution ddfm=satterthwaite;
  random surgeon;
  lsmeans group / cl pdiff;
  ods output LSMeans=lsmeans_hospital CovParms=covparms_hosp;
  title "Sensitivity Analysis 1: Mixed Model Adjusting Hospital";
run;

/* Extract Residual Standard Deviation */
data _null_;
  set covparms_hosp;
  if CovParm="Residual" then call symputx("resid_sd_hosp",
sqrt(Estimate));
run;

/* Calculate Cohen's d */
data cohens_d_hosp;
  length label $50;
  set lsmeans_hospital(where=(group in (1,2))
rename=(Estimate=adj_mean StdErr=adj_se));
  retain mean1 mean2;
  if group=1 then mean1=adj_mean;
  else if group=2 then mean2=adj_mean;
  if _n_=2 then do;
    s_pooled = &resid_sd_hosp;
    cohens_d = (mean1 - mean2) / s_pooled;
    label = "Sensitivity Analysis Adjusting Hospital";
    output;
```

```
  end;
  keep label cohens_d mean1 mean2 s_pooled;
run;

proc print data=cohens_d_hosp noobs label;
  title "Cohen's d from Sensitivity Analysis Adjusting Hospital";
run;


/* 4. Sensitivity Analysis 2: Adjusting Surgery Type (Mixed Model) */
proc mixed data=sensitive_data method=reml noclprint;
  class group surgeon surgery_type;
  model sensitive = group surgery_type / solution ddfm=satterthwaite;
  random surgeon;
  lsmeans group / cl pdiff;
  ods output LSMeans=lsmeans_surgtype CovParms=covparms_surgtype;
  title "Sensitivity Analysis 2: Mixed Model Adjusting Surgery Type";
run;

/* Extract Residual Standard Deviation */
data _null_;
  set covparms_surgtype;
  if CovParm="Residual" then call symputx("resid_sd_surgtype",
sqrt(Estimate));
run;

/* Calculate Cohen's d */
data cohens_d_surgtype;
  length label $50;
  set lsmeans_surgtype(where=(group in (1,2))
rename=(Estimate=adj_mean StdErr=adj_se));
  retain mean1 mean2;
  if group=1 then mean1=adj_mean;
  else if group=2 then mean2=adj_mean;
  if _n_=2 then do;
    s_pooled = &resid_sd_surgtype;
    cohens_d = (mean1 - mean2) / s_pooled;
    label = "Sensitivity Analysis Adjusting Surgery Type";
    output;
  end;
  keep label cohens_d mean1 mean2 s_pooled;
run;

proc print data=cohens_d_surgtype noobs label;
```

```
  title "Cohen's d from Sensitivity Analysis Adjusting Surgery Type";
run;


/* 5. Sensitivity Analysis 3: Adjusting Surgeon (fixed effect) */
proc mixed data=sensitive_data method=reml noclprint;
  class group surgeon;
  model sensitive = group surgeon / solution ddfm=satterthwaite;
  lsmeans group / cl pdiff;
  ods output LSMeans=lsmeans_surg_fixed CovParms=covparms_surg_fixed;
  title "Sensitivity Analysis 3: Mixed Model Adjusting Surgeon (as
fixed effect)";
run;

/* Extract Residual Standard Deviation */
data _null_;
  set covparms_surg_fixed;
  if CovParm="Residual" then call symputx("resid_sd_surg_fixed",
sqrt(Estimate));
run;

/* Calculate Cohen's d */
data cohens_d_surg_fixed;
  length label $50;
  set lsmeans_surg_fixed(where=(group in (1,2))
rename=(Estimate=adj_mean StdErr=adj_se));
  retain mean1 mean2;
  if group=1 then mean1=adj_mean;
  else if group=2 then mean2=adj_mean;
  if _n_=2 then do;
    s_pooled = &resid_sd_surg_fixed;
    cohens_d = (mean1 - mean2) / s_pooled;
    label = "Sensitivity Analysis Adjusting Surgeon (fixed effect)";
    output;
  end;
  keep label cohens_d mean1 mean2 s_pooled;
run;

proc print data=cohens_d_surg_fixed noobs label;
  title "Cohen's d from Sensitivity Analysis Adjusting Surgeon
(fixed)";
run;
```

3.4 Postoperative hospitalization days

```
/* Data Input */
/* Data details:
   group: 1=telesurgery group, 2=local surgery group
   hospital: 1=Beijing, 2=Harbin, 3=Hangzhou, 4=Hefei, 5=Urumqi
   surgery_type: 1=Radical Prostatectomy, 2=Partial Nephrectomy
   surgeon codes: 1=Chaozhao Liang, 2=Baojun Wang, 3=Xu Zhang,
4=Sheng Tai,
                  5=Hongzhao Li, 6=Wanghai Xu, 7=Xin Ma, 8=Qingbo
Huang,
                  9=Weijun Fu, 10=Shuo Wang, 11=Mulati Rexiati
*/
data sensitive_data;
input group hospital surgery_type surgeon sensitive;
datalines;
2   3   1   10  4
1   3   1   5   4
2   3   1   10  4
2   3   1   10  11
1   3   1   5   5
2   3   1   10  5
1   3   2   7   7
2   3   2   10  4
2   3   2   10  5
1   3   2   7   5
2   3   2   10  5
1   3   2   7   5
2   3   2   10  5
1   3   2   7   6
1   4   1   2   6
2   4   1   4   7
2   4   1   4   5
1   4   1   3   6
1   4   1   5   5
2   4   1   1   5
2   4   2   1   7
2   4   2   1   7
1   4   2   7   6
1   5   1   5   4
1   5   1   5   6
1   5   1   5   8
1   5   1   2   5
2   5   1   2   5
2   5   1   11  5
```

```
1   5   2   7   5
2   5   2   8   3
2   5   2   8   7
1   5   2   8   5
2   1   1   9   6
1   1   1   6   6
2   1   1   9   6
1   1   1   1   6
2   1   1   3   8
2   1   1   9   6
1   1   1   10  6
1   1   1   10  4
1   1   1   10  5
2   1   1   9   7
1   1   1   9   11
2   1   1   9   3
2   1   2   7   4
2   1   2   7   11
1   1   2   6   3
1   1   2   1   6
1   1   2   1   5
1   1   2   10  4
2   1   2   7   4
2   1   2   7   3
1   1   2   8   3
2   1   2   7   4
1   1   2   8   4
1   2   1   5   8
2   2   1   6   9
1   2   1   7   6
2   2   2   6   8
2   2   2   6   15
1   2   2   7   11
1   2   2   8   10
;
run;


/* 2. Main Analysis: Mixed Model (Surgeon as Random Effect) */
proc mixed data=sensitive_data method=reml noclprint;
  class group surgeon;
  model sensitive = group / solution ddfm=satterthwaite;
  random surgeon;
  lsmeans group / cl pdiff;
```

```
  ods output LSMeans=lsmeans_mixed CovParms=covparms;
  title "Main Analysis: Mixed Effects Model with Surgeon as Random";
run;

/* Extract Residual Standard Deviation */
data _null_;
  set covparms;
  if CovParm="Residual" then call symputx("resid_sd_mixed",
sqrt(Estimate));
run;

/* Calculate Cohen's d */
data cohens_d_mixed;
  length label $50;
  set lsmeans_mixed(where=(group in (1,2)) rename=(Estimate=adj_mean
StdErr=adj_se));
  retain mean1 mean2;
  if group=1 then mean1=adj_mean;
  else if group=2 then mean2=adj_mean;
  if _n_=2 then do;
    s_pooled = &resid_sd_mixed;
    cohens_d = (mean1 - mean2) / s_pooled;
    label = "Main Analysis (Mixed Model)";
    output;
  end;
  keep label cohens_d mean1 mean2 s_pooled;
run;

proc print data=cohens_d_mixed noobs label;
  title "Cohen's d from Main Analysis (Mixed Effects Model)";
run;


/* 3. Sensitivity Analysis 1: Adjusting Hospital (Mixed Model) */
proc mixed data=sensitive_data method=reml noclprint;
  class group surgeon hospital;
  model sensitive = group hospital / solution ddfm=satterthwaite;
  random surgeon;
  lsmeans group / cl pdiff;
  ods output LSMeans=lsmeans_hospital CovParms=covparms_hosp;
  title "Sensitivity Analysis 1: Mixed Model Adjusting Hospital";
run;

/* Extract Residual Standard Deviation */
```

```
data _null_;
  set covparms_hosp;
  if CovParm="Residual" then call symputx("resid_sd_hosp",
sqrt(Estimate));
run;

/* Calculate Cohen's d */
data cohens_d_hosp;
  length label $50;
  set lsmeans_hospital(where=(group in (1,2))
rename=(Estimate=adj_mean StdErr=adj_se));
  retain mean1 mean2;
  if group=1 then mean1=adj_mean;
  else if group=2 then mean2=adj_mean;
  if _n_=2 then do;
    s_pooled = &resid_sd_hosp;
    cohens_d = (mean1 - mean2) / s_pooled;
    label = "Sensitivity Analysis Adjusting Hospital";
    output;
  end;
  keep label cohens_d mean1 mean2 s_pooled;
run;

proc print data=cohens_d_hosp noobs label;
  title "Cohen's d from Sensitivity Analysis Adjusting Hospital";
run;


/* 4. Sensitivity Analysis 2: Adjusting Surgery Type (Mixed Model) */
proc mixed data=sensitive_data method=reml noclprint;
  class group surgeon surgery_type;
  model sensitive = group surgery_type / solution ddfm=satterthwaite;
  random surgeon;
  lsmeans group / cl pdiff;
  ods output LSMeans=lsmeans_surgtype CovParms=covparms_surgtype;
  title "Sensitivity Analysis 2: Mixed Model Adjusting Surgery Type";
run;

/* Extract Residual Standard Deviation */
data _null_;
  set covparms_surgtype;
  if CovParm="Residual" then call symputx("resid_sd_surgtype",
sqrt(Estimate));
run;
```

```
/* Calculate Cohen's d */
data cohens_d_surgtype;
  length label $50;
  set lsmeans_surgtype(where=(group in (1,2))
rename=(Estimate=adj_mean StdErr=adj_se));
  retain mean1 mean2;
  if group=1 then mean1=adj_mean;
  else if group=2 then mean2=adj_mean;
  if _n_=2 then do;
    s_pooled = &resid_sd_surgtype;
    cohens_d = (mean1 - mean2) / s_pooled;
    label = "Sensitivity Analysis Adjusting Surgery Type";
    output;
  end;
  keep label cohens_d mean1 mean2 s_pooled;
run;

proc print data=cohens_d_surgtype noobs label;
  title "Cohen's d from Sensitivity Analysis Adjusting Surgery Type";
run;


/* 5. Sensitivity Analysis 3: Adjusting Surgeon (fixed effect) */
proc mixed data=sensitive_data method=reml noclprint;
  class group surgeon;
  model sensitive = group surgeon / solution ddfm=satterthwaite;
  lsmeans group / cl pdiff;
  ods output LSMeans=lsmeans_surg_fixed CovParms=covparms_surg_fixed;
  title "Sensitivity Analysis 3: Mixed Model Adjusting Surgeon (as
fixed effect)";
run;

/* Extract Residual Standard Deviation */
data _null_;
  set covparms_surg_fixed;
  if CovParm="Residual" then call symputx("resid_sd_surg_fixed",
sqrt(Estimate));
run;

/* Calculate Cohen's d */
data cohens_d_surg_fixed;
  length label $50;
  set lsmeans_surg_fixed(where=(group in (1,2))
```

```
rename=(Estimate=adj_mean StdErr=adj_se));
  retain mean1 mean2;
  if group=1 then mean1=adj_mean;
  else if group=2 then mean2=adj_mean;
  if _n_=2 then do;
    s_pooled = &resid_sd_surg_fixed;
    cohens_d = (mean1 - mean2) / s_pooled;
    label = "Sensitivity Analysis Adjusting Surgeon (fixed effect)";
    output;
  end;
  keep label cohens_d mean1 mean2 s_pooled;
run;

proc print data=cohens_d_surg_fixed noobs label;
  title "Cohen's d from Sensitivity Analysis Adjusting Surgeon
(fixed)";
run;
```

3.5 Length of days in critical care

```
/* Data Input */
/* Data details:
   group: 1=telesurgery group, 2=local surgery group
   hospital: 1=Beijing, 2=Harbin, 3=Hangzhou, 4=Hefei, 5=Urumqi
   surgery_type: 1=Radical Prostatectomy, 2=Partial Nephrectomy
   surgeon codes: 1=Chaozhao Liang, 2=Baojun Wang, 3=Xu Zhang,
4=Sheng Tai,
                  5=Hongzhao Li, 6=Wanghai Xu, 7=Xin Ma, 8=Qingbo
Huang,
                  9=Weijun Fu, 10=Shuo Wang, 11=Mulati Rexiati
*/
data sensitive_data;
input group hospital surgery_type surgeon sensitive;
datalines;
2   3   1   10  0
1   3   1   5   0
2   3   1   10  0
2   3   1   10  0
1   3   1   5   0
2   3   1   10  0
1   3   2   7   0
2   3   2   10  0
2   3   2   10  0
1   3   2   7   0
```

2	3	2	10	0
1	3	2	7	0
2	3	2	10	0
1	3	2	7	0
1	4	1	2	0
2	4	1	4	0
2	4	1	4	0
1	4	1	3	0
1	4	1	5	0
2	4	1	1	0
2	4	2	1	0
2	4	2	1	0
1	4	2	7	0
1	5	1	5	0
1	5	1	5	0
1	5	1	5	0
1	5	1	2	1
2	5	1	2	0
2	5	1	11	1
1	5	2	7	0
2	5	2	8	0
2	5	2	8	0
1	5	2	8	0
2	1	1	9	0
1	1	1	6	0
2	1	1	9	0
1	1	1	1	0
2	1	1	3	0
2	1	1	9	0
1	1	1	10	0
1	1	1	10	0
1	1	1	10	0
2	1	1	9	0
1	1	1	9	0
2	1	1	9	0
2	1	2	7	0
2	1	2	7	0
1	1	2	6	0
1	1	2	1	0
1	1	2	1	0
1	1	2	10	0
2	1	2	7	0
2	1	2	7	0
1	1	2	8	0

```
2   1   2   7   0
1   1   2   8   0
1   2   1   5   0
2   2   1   6   0
1   2   1   7   0
2   2   2   6   0
2   2   2   6   0
1   2   2   7   0
1   2   2   8   0
;
run;


/* 2. Main Analysis: Mixed Model (Surgeon as Random Effect) */
proc mixed data=sensitive_data method=reml noclprint;
  class group surgeon;
  model sensitive = group / solution ddfm=satterthwaite;
  random surgeon;
  lsmeans group / cl pdiff;
  ods output LSMeans=lsmeans_mixed CovParms=covparms;
  title "Main Analysis: Mixed Effects Model with Surgeon as Random";
run;

/* Extract Residual Standard Deviation */
data _null_;
  set covparms;
  if CovParm="Residual" then call symputx("resid_sd_mixed",
sqrt(Estimate));
run;

/* Calculate Cohen's d */
data cohens_d_mixed;
  length label $50;
  set lsmeans_mixed(where=(group in (1,2)) rename=(Estimate=adj_mean
StdErr=adj_se));
  retain mean1 mean2;
  if group=1 then mean1=adj_mean;
  else if group=2 then mean2=adj_mean;
  if _n_=2 then do;
    s_pooled = &resid_sd_mixed;
    cohens_d = (mean1 - mean2) / s_pooled;
    label = "Main Analysis (Mixed Model)";
    output;
  end;
```

```
  keep label cohens_d mean1 mean2 s_pooled;
run;

proc print data=cohens_d_mixed noobs label;
  title "Cohen's d from Main Analysis (Mixed Effects Model)";
run;


/* 3. Sensitivity Analysis 1: Adjusting Hospital (Mixed Model) */
proc mixed data=sensitive_data method=reml noclprint;
  class group surgeon hospital;
  model sensitive = group hospital / solution ddfm=satterthwaite;
  random surgeon;
  lsmeans group / cl pdiff;
  ods output LSMeans=lsmeans_hospital CovParms=covparms_hosp;
  title "Sensitivity Analysis 1: Mixed Model Adjusting Hospital";
run;

/* Extract Residual Standard Deviation */
data _null_;
  set covparms_hosp;
  if CovParm="Residual" then call symputx("resid_sd_hosp",
sqrt(Estimate));
run;

/* Calculate Cohen's d */
data cohens_d_hosp;
  length label $50;
  set lsmeans_hospital(where=(group in (1,2))
rename=(Estimate=adj_mean StdErr=adj_se));
  retain mean1 mean2;
  if group=1 then mean1=adj_mean;
  else if group=2 then mean2=adj_mean;
  if _n_=2 then do;
    s_pooled = &resid_sd_hosp;
    cohens_d = (mean1 - mean2) / s_pooled;
    label = "Sensitivity Analysis Adjusting Hospital";
    output;
  end;
  keep label cohens_d mean1 mean2 s_pooled;
run;

proc print data=cohens_d_hosp noobs label;
  title "Cohen's d from Sensitivity Analysis Adjusting Hospital";
```

```
run;


/* 4. Sensitivity Analysis 2: Adjusting Surgery Type (Mixed Model) */
proc mixed data=sensitive_data method=reml noclprint;
  class group surgeon surgery_type;
  model sensitive = group surgery_type / solution ddfm=satterthwaite;
  random surgeon;
  lsmeans group / cl pdiff;
  ods output LSMeans=lsmeans_surgtype CovParms=covparms_surgtype;
  title "Sensitivity Analysis 2: Mixed Model Adjusting Surgery Type";
run;

/* Extract Residual Standard Deviation */
data _null_;
  set covparms_surgtype;
  if CovParm="Residual" then call symputx("resid_sd_surgtype",
sqrt(Estimate));
run;

/* Calculate Cohen's d */
data cohens_d_surgtype;
  length label $50;
  set lsmeans_surgtype(where=(group in (1,2))
rename=(Estimate=adj_mean StdErr=adj_se));
  retain mean1 mean2;
  if group=1 then mean1=adj_mean;
  else if group=2 then mean2=adj_mean;
  if _n_=2 then do;
    s_pooled = &resid_sd_surgtype;
    cohens_d = (mean1 - mean2) / s_pooled;
    label = "Sensitivity Analysis Adjusting Surgery Type";
    output;
  end;
  keep label cohens_d mean1 mean2 s_pooled;
run;

proc print data=cohens_d_surgtype noobs label;
  title "Cohen's d from Sensitivity Analysis Adjusting Surgery Type";
run;


/* 5. Sensitivity Analysis 3: Adjusting Surgeon (fixed effect) */
proc mixed data=sensitive_data method=reml noclprint;
```

```
  class group surgeon;
  model sensitive = group surgeon / solution ddfm=satterthwaite;
  lsmeans group / cl pdiff;
  ods output LSMeans=lsmeans_surg_fixed CovParms=covparms_surg_fixed;
  title "Sensitivity Analysis 3: Mixed Model Adjusting Surgeon (as
fixed effect)";
run;

/* Extract Residual Standard Deviation */
data _null_;
  set covparms_surg_fixed;
  if CovParm="Residual" then call symputx("resid_sd_surg_fixed",
sqrt(Estimate));
run;

/* Calculate Cohen's d */
data cohens_d_surg_fixed;
  length label $50;
  set lsmeans_surg_fixed(where=(group in (1,2))
rename=(Estimate=adj_mean StdErr=adj_se));
  retain mean1 mean2;
  if group=1 then mean1=adj_mean;
  else if group=2 then mean2=adj_mean;
  if _n_=2 then do;
    s_pooled = &resid_sd_surg_fixed;
    cohens_d = (mean1 - mean2) / s_pooled;
    label = "Sensitivity Analysis Adjusting Surgeon (fixed effect)";
    output;
  end;
  keep label cohens_d mean1 mean2 s_pooled;
run;

proc print data=cohens_d_surg_fixed noobs label;
  title "Cohen's d from Sensitivity Analysis Adjusting Surgeon
(fixed)";
run;
```

3.6 QoR15 Score

3.6.1 Total for renal tumor and prostate cancer patients

3.6.1.1 Baseline

```
/* Data Input */
/* Data details:
   group: 1=telesurgery group, 2=local surgery group
```

```
   hospital: 1=Beijing, 2=Harbin, 3=Hangzhou, 4=Hefei, 5=Urumqi
   surgery_type: 1=Radical Prostatectomy, 2=Partial Nephrectomy
   surgeon codes: 1=Chaozhao Liang, 2=Baojun Wang, 3=Xu Zhang,
4=Sheng Tai,
                 5=Hongzhao Li, 6=Wanghai Xu, 7=Xin Ma, 8=Qingbo
Huang,
                 9=Weijun Fu, 10=Shuo Wang, 11=Mulati Rexiati
*/
data sensitive_data;
input group hospital surgery_type surgeon sensitive;
datalines;
2   3   1   10  139
1   3   1   5   146
2   3   1   10  148
2   3   1   10  139
1   3   1   5   150
2   3   1   10  150
1   3   2   7   149
2   3   2   10  150
2   3   2   10  145
1   3   2   7   150
2   3   2   10  150
1   3   2   7   146
2   3   2   10  150
1   3   2   7   136
1   4   1   2   130
2   4   1   4   147
2   4   1   4   145
1   4   1   3   150
1   4   1   5   141
2   4   1   1   137
2   4   2   1   134
2   4   2   1   138
1   4   2   7   121
1   5   1   5   145
1   5   1   5   145
1   5   1   5   150
1   5   1   2   150
2   5   1   2   150
2   5   1   11  150
1   5   2   7   149
2   5   2   8   146
2   5   2   8   150
1   5   2   8   150
```

```
2   1   1   9   148
1   1   1   6   150
2   1   1   9   141
1   1   1   1   150
2   1   1   3   146
2   1   1   9   143
1   1   1   10  131
1   1   1   10  147
1   1   1   10  138
2   1   1   9   150
1   1   1   9   144
2   1   1   9   150
2   1   2   7   149
2   1   2   7   147
1   1   2   6   150
1   1   2   1   134
1   1   2   1   142
1   1   2   10  132
2   1   2   7   150
2   1   2   7   149
1   1   2   8   150
2   1   2   7   150
1   1   2   8   150
1   2   1   5   148
2   2   1   6   150
1   2   1   7   149
2   2   2   6   150
2   2   2   6   146
1   2   2   7   143
1   2   2   8   149
;
run;


/* 2. Main Analysis: Mixed Model (Surgeon as Random Effect) */
proc mixed data=sensitive_data method=reml noclprint;
  class group surgeon;
  model sensitive = group / solution ddfm=satterthwaite;
  random surgeon;
  lsmeans group / cl pdiff;
  ods output LSMeans=lsmeans_mixed CovParms=covparms;
  title "Main Analysis: Mixed Effects Model with Surgeon as Random";
run;
```

```
/* Extract Residual Standard Deviation */
data _null_;
  set covparms;
  if CovParm="Residual" then call symputx("resid_sd_mixed",
sqrt(Estimate));
run;

/* Calculate Cohen's d */
data cohens_d_mixed;
  length label $50;
  set lsmeans_mixed(where=(group in (1,2)) rename=(Estimate=adj_mean
StdErr=adj_se));
  retain mean1 mean2;
  if group=1 then mean1=adj_mean;
  else if group=2 then mean2=adj_mean;
  if _n_=2 then do;
    s_pooled = &resid_sd_mixed;
    cohens_d = (mean1 - mean2) / s_pooled;
    label = "Main Analysis (Mixed Model)";
    output;
  end;
  keep label cohens_d mean1 mean2 s_pooled;
run;

proc print data=cohens_d_mixed noobs label;
  title "Cohen's d from Main Analysis (Mixed Effects Model)";
run;


/* 3. Sensitivity Analysis 1: Adjusting Hospital (Mixed Model) */
proc mixed data=sensitive_data method=reml noclprint;
  class group surgeon hospital;
  model sensitive = group hospital / solution ddfm=satterthwaite;
  random surgeon;
  lsmeans group / cl pdiff;
  ods output LSMeans=lsmeans_hospital CovParms=covparms_hosp;
  title "Sensitivity Analysis 1: Mixed Model Adjusting Hospital";
run;

/* Extract Residual Standard Deviation */
data _null_;
  set covparms_hosp;
  if CovParm="Residual" then call symputx("resid_sd_hosp",
sqrt(Estimate));
```

```
run;

/* Calculate Cohen's d */
data cohens_d_hosp;
  length label $50;
  set lsmeans_hospital(where=(group in (1,2))
rename=(Estimate=adj_mean StdErr=adj_se));
  retain mean1 mean2;
  if group=1 then mean1=adj_mean;
  else if group=2 then mean2=adj_mean;
  if _n_=2 then do;
    s_pooled = &resid_sd_hosp;
    cohens_d = (mean1 - mean2) / s_pooled;
    label = "Sensitivity Analysis Adjusting Hospital";
    output;
  end;
  keep label cohens_d mean1 mean2 s_pooled;
run;

proc print data=cohens_d_hosp noobs label;
  title "Cohen's d from Sensitivity Analysis Adjusting Hospital";
run;


/* 4. Sensitivity Analysis 2: Adjusting Surgery Type (Mixed Model) */
proc mixed data=sensitive_data method=reml noclprint;
  class group surgeon surgery_type;
  model sensitive = group surgery_type / solution ddfm=satterthwaite;
  random surgeon;
  lsmeans group / cl pdiff;
  ods output LSMeans=lsmeans_surgtype CovParms=covparms_surgtype;
  title "Sensitivity Analysis 2: Mixed Model Adjusting Surgery Type";
run;

/* Extract Residual Standard Deviation */
data _null_;
  set covparms_surgtype;
  if CovParm="Residual" then call symputx("resid_sd_surgtype",
sqrt(Estimate));
run;

/* Calculate Cohen's d */
data cohens_d_surgtype;
  length label $50;
```

```
  set lsmeans_surgtype(where=(group in (1,2))
rename=(Estimate=adj_mean StdErr=adj_se));
  retain mean1 mean2;
  if group=1 then mean1=adj_mean;
  else if group=2 then mean2=adj_mean;
  if _n_=2 then do;
    s_pooled = &resid_sd_surgtype;
    cohens_d = (mean1 - mean2) / s_pooled;
    label = "Sensitivity Analysis Adjusting Surgery Type";
    output;
  end;
  keep label cohens_d mean1 mean2 s_pooled;
run;

proc print data=cohens_d_surgtype noobs label;
  title "Cohen's d from Sensitivity Analysis Adjusting Surgery Type";
run;


/* 5. Sensitivity Analysis 3: Adjusting Surgeon (fixed effect) */
proc mixed data=sensitive_data method=reml noclprint;
  class group surgeon;
  model sensitive = group surgeon / solution ddfm=satterthwaite;
  lsmeans group / cl pdiff;
  ods output LSMeans=lsmeans_surg_fixed CovParms=covparms_surg_fixed;
  title "Sensitivity Analysis 3: Mixed Model Adjusting Surgeon (as
fixed effect)";
run;

/* Extract Residual Standard Deviation */
data _null_;
  set covparms_surg_fixed;
  if CovParm="Residual" then call symputx("resid_sd_surg_fixed",
sqrt(Estimate));
run;

/* Calculate Cohen's d */
data cohens_d_surg_fixed;
  length label $50;
  set lsmeans_surg_fixed(where=(group in (1,2))
rename=(Estimate=adj_mean StdErr=adj_se));
  retain mean1 mean2;
  if group=1 then mean1=adj_mean;
  else if group=2 then mean2=adj_mean;
```

```
  if _n_=2 then do;
    s_pooled = &resid_sd_surg_fixed;
    cohens_d = (mean1 - mean2) / s_pooled;
    label = "Sensitivity Analysis Adjusting Surgeon (fixed effect)";
    output;
  end;
  keep label cohens_d mean1 mean2 s_pooled;
run;

proc print data=cohens_d_surg_fixed noobs label;
  title "Cohen's d from Sensitivity Analysis Adjusting Surgeon
(fixed)";
run;
```

3.6.1.2 At 4w follow-up point

```
/* Data Input */
/* Data details:
   group: 1=telesurgery group, 2=local surgery group
   hospital: 1=Beijing, 2=Harbin, 3=Hangzhou, 4=Hefei, 5=Urumqi
   surgery_type: 1=Radical Prostatectomy, 2=Partial Nephrectomy
   surgeon codes: 1=Chaozhao Liang, 2=Baojun Wang, 3=Xu Zhang,
4=Sheng Tai,
                  5=Hongzhao Li, 6=Wanghai Xu, 7=Xin Ma, 8=Qingbo
Huang,
                  9=Weijun Fu, 10=Shuo Wang, 11=Mulati Rexiati
*/
data sensitive_data;
input group hospital surgery_type surgeon sensitive;
datalines;
2   3   1   10  137
1   3   1   5   148
2   3   1   10  150
2   3   1   10  150
1   3   1   5   150
2   3   1   10  150
1   3   2   7   123
2   3   2   10  150
2   3   2   10  137
1   3   2   7   149
2   3   2   10  150
1   3   2   7   150
2   3   2   10  145
1   3   2   7   146
```

1	4	1	2	135
2	4	1	4	138
2	4	1	4	131
1	4	1	3	121
1	4	1	5	137
2	4	1	1	140
2	4	2	1	141
2	4	2	1	138
1	4	2	7	139
1	5	1	5	149
1	5	1	5	148
1	5	1	5	150
1	5	1	2	149
2	5	1	2	150
2	5	1	11	149
1	5	2	7	149
2	5	2	8	148
2	5	2	8	150
1	5	2	8	150
2	1	1	9	145
1	1	1	6	144
2	1	1	9	118
1	1	1	1	140
2	1	1	3	145
2	1	1	9	141
1	1	1	10	147
1	1	1	10	148
1	1	1	10	137
2	1	1	9	145
1	1	1	9	148
2	1	1	9	147
2	1	2	7	148
2	1	2	7	149
1	1	2	6	148
1	1	2	1	144
1	1	2	1	150
1	1	2	10	134
2	1	2	7	133
2	1	2	7	143
1	1	2	8	129
2	1	2	7	146
1	1	2	8	146
1	2	1	5	123
2	2	1	6	129

```
1   2   1   7   135
2   2   2   6   149
2   2   2   6   138
1   2   2   7   141
1   2   2   8   136
;
run;


/* 2. Main Analysis: Mixed Model (Surgeon as Random Effect) */
proc mixed data=sensitive_data method=reml noclprint;
  class group surgeon;
  model sensitive = group / solution ddfm=satterthwaite;
  random surgeon;
  lsmeans group / cl pdiff;
  ods output LSMeans=lsmeans_mixed CovParms=covparms;
  title "Main Analysis: Mixed Effects Model with Surgeon as Random";
run;

/* Extract Residual Standard Deviation */
data _null_;
  set covparms;
  if CovParm="Residual" then call symputx("resid_sd_mixed",
sqrt(Estimate));
run;

/* Calculate Cohen's d */
data cohens_d_mixed;
  length label $50;
  set lsmeans_mixed(where=(group in (1,2)) rename=(Estimate=adj_mean
StdErr=adj_se));
  retain mean1 mean2;
  if group=1 then mean1=adj_mean;
  else if group=2 then mean2=adj_mean;
  if _n_=2 then do;
    s_pooled = &resid_sd_mixed;
    cohens_d = (mean1 - mean2) / s_pooled;
    label = "Main Analysis (Mixed Model)";
    output;
  end;
  keep label cohens_d mean1 mean2 s_pooled;
run;

proc print data=cohens_d_mixed noobs label;
```

```
  title "Cohen's d from Main Analysis (Mixed Effects Model)";
run;


/* 3. Sensitivity Analysis 1: Adjusting Hospital (Mixed Model) */
proc mixed data=sensitive_data method=reml noclprint;
  class group surgeon hospital;
  model sensitive = group hospital / solution ddfm=satterthwaite;
  random surgeon;
  lsmeans group / cl pdiff;
  ods output LSMeans=lsmeans_hospital CovParms=covparms_hosp;
  title "Sensitivity Analysis 1: Mixed Model Adjusting Hospital";
run;

/* Extract Residual Standard Deviation */
data _null_;
  set covparms_hosp;
  if CovParm="Residual" then call symputx("resid_sd_hosp",
sqrt(Estimate));
run;

/* Calculate Cohen's d */
data cohens_d_hosp;
  length label $50;
  set lsmeans_hospital(where=(group in (1,2))
rename=(Estimate=adj_mean StdErr=adj_se));
  retain mean1 mean2;
  if group=1 then mean1=adj_mean;
  else if group=2 then mean2=adj_mean;
  if _n_=2 then do;
    s_pooled = &resid_sd_hosp;
    cohens_d = (mean1 - mean2) / s_pooled;
    label = "Sensitivity Analysis Adjusting Hospital";
    output;
  end;
  keep label cohens_d mean1 mean2 s_pooled;
run;

proc print data=cohens_d_hosp noobs label;
  title "Cohen's d from Sensitivity Analysis Adjusting Hospital";
run;


/* 4. Sensitivity Analysis 2: Adjusting Surgery Type (Mixed Model) */
```

```
proc mixed data=sensitive_data method=reml noclprint;
  class group surgeon surgery_type;
  model sensitive = group surgery_type / solution ddfm=satterthwaite;
  random surgeon;
  lsmeans group / cl pdiff;
  ods output LSMeans=lsmeans_surgtype CovParms=covparms_surgtype;
  title "Sensitivity Analysis 2: Mixed Model Adjusting Surgery Type";
run;

/* Extract Residual Standard Deviation */
data _null_;
  set covparms_surgtype;
  if CovParm="Residual" then call symputx("resid_sd_surgtype",
sqrt(Estimate));
run;

/* Calculate Cohen's d */
data cohens_d_surgtype;
  length label $50;
  set lsmeans_surgtype(where=(group in (1,2))
rename=(Estimate=adj_mean StdErr=adj_se));
  retain mean1 mean2;
  if group=1 then mean1=adj_mean;
  else if group=2 then mean2=adj_mean;
  if _n_=2 then do;
    s_pooled = &resid_sd_surgtype;
    cohens_d = (mean1 - mean2) / s_pooled;
    label = "Sensitivity Analysis Adjusting Surgery Type";
    output;
  end;
  keep label cohens_d mean1 mean2 s_pooled;
run;

proc print data=cohens_d_surgtype noobs label;
  title "Cohen's d from Sensitivity Analysis Adjusting Surgery Type";
run;


/* 5. Sensitivity Analysis 3: Adjusting Surgeon (fixed effect) */
proc mixed data=sensitive_data method=reml noclprint;
  class group surgeon;
  model sensitive = group surgeon / solution ddfm=satterthwaite;
  lsmeans group / cl pdiff;
  ods output LSMeans=lsmeans_surg_fixed CovParms=covparms_surg_fixed;
```

```
  title "Sensitivity Analysis 3: Mixed Model Adjusting Surgeon (as
fixed effect)";
run;

/* Extract Residual Standard Deviation */
data _null_;
  set covparms_surg_fixed;
  if CovParm="Residual" then call symputx("resid_sd_surg_fixed",
sqrt(Estimate));
run;

/* Calculate Cohen's d */
data cohens_d_surg_fixed;
  length label $50;
  set lsmeans_surg_fixed(where=(group in (1,2))
rename=(Estimate=adj_mean StdErr=adj_se));
  retain mean1 mean2;
  if group=1 then mean1=adj_mean;
  else if group=2 then mean2=adj_mean;
  if _n_=2 then do;
    s_pooled = &resid_sd_surg_fixed;
    cohens_d = (mean1 - mean2) / s_pooled;
    label = "Sensitivity Analysis Adjusting Surgeon (fixed effect)";
    output;
  end;
  keep label cohens_d mean1 mean2 s_pooled;
run;

proc print data=cohens_d_surg_fixed noobs label;
  title "Cohen's d from Sensitivity Analysis Adjusting Surgeon
(fixed)";
run;
```

3.6.1.3 At 6w follow-up point

```
/* Data Input */
/* Data details:
   group: 1=telesurgery group, 2=local surgery group
   hospital: 1=Beijing, 2=Harbin, 3=Hangzhou, 4=Hefei, 5=Urumqi
   surgery_type: 1=Radical Prostatectomy, 2=Partial Nephrectomy
   surgeon codes: 1=Chaozhao Liang, 2=Baojun Wang, 3=Xu Zhang,
4=Sheng Tai,
                  5=Hongzhao Li, 6=Wanghai Xu, 7=Xin Ma, 8=Qingbo
Huang,
```

```
                   9=Weijun Fu, 10=Shuo Wang, 11=Mulati Rexiati
*/
data sensitive_data;
input group hospital surgery_type surgeon sensitive;
datalines;
2   3   1   10  146
1   3   1   5   148
2   3   1   10  150
2   3   1   10  150
1   3   1   5   150
2   3   1   10  150
1   3   2   7   123
2   3   2   10  150
2   3   2   10  138
1   3   2   7   149
2   3   2   10  150
1   3   2   7   150
2   3   2   10  145
1   3   2   7   147
1   4   1   2   141
2   4   1   4   140
2   4   1   4   131
1   4   1   3   139
1   4   1   5   138
2   4   1   1
2   4   2   1
2   4   2   1
1   4   2   7
1   5   1   5   150
1   5   1   5   149
1   5   1   5   150
1   5   1   2   150
2   5   1   2   150
2   5   1   11  150
1   5   2   7   150
2   5   2   8   149
2   5   2   8   150
1   5   2   8   150
2   1   1   9   144
1   1   1   6   130
2   1   1   9   135
1   1   1   1   132
2   1   1   3   144
2   1   1   9   132
```

```
1   1   1   10  143
1   1   1   10  147
1   1   1   10  137
2   1   1   9   150
1   1   1   9   145
2   1   1   9   147
2   1   2   7   150
2   1   2   7
1   1   2   6   149
1   1   2   1   147
1   1   2   1   150
1   1   2   10  136
2   1   2   7   142
2   1   2   7   150
1   1   2   8   126
2   1   2   7   148
1   1   2   8   150
1   2   1   5   143
2   2   1   6   140
1   2   1   7   144
2   2   2   6   150
2   2   2   6   145
1   2   2   7   146
1   2   2   8   146;
run;


/* 2. Main Analysis: Mixed Model (Surgeon as Random Effect) */
proc mixed data=sensitive_data method=reml noclprint;
  class group surgeon;
  model sensitive = group / solution ddfm=satterthwaite;
  random surgeon;
  lsmeans group / cl pdiff;
  ods output LSMeans=lsmeans_mixed CovParms=covparms;
  title "Main Analysis: Mixed Effects Model with Surgeon as Random";
run;

/* Extract Residual Standard Deviation */
data _null_;
  set covparms;
  if CovParm="Residual" then call symputx("resid_sd_mixed",
sqrt(Estimate));
run;
```

```
/* Calculate Cohen's d */
data cohens_d_mixed;
  length label $50;
  set lsmeans_mixed(where=(group in (1,2)) rename=(Estimate=adj_mean
StdErr=adj_se));
  retain mean1 mean2;
  if group=1 then mean1=adj_mean;
  else if group=2 then mean2=adj_mean;
  if _n_=2 then do;
    s_pooled = &resid_sd_mixed;
    cohens_d = (mean1 - mean2) / s_pooled;
    label = "Main Analysis (Mixed Model)";
    output;
  end;
  keep label cohens_d mean1 mean2 s_pooled;
run;

proc print data=cohens_d_mixed noobs label;
  title "Cohen's d from Main Analysis (Mixed Effects Model)";
run;


/* 3. Sensitivity Analysis 1: Adjusting Hospital (Mixed Model) */
proc mixed data=sensitive_data method=reml noclprint;
  class group surgeon hospital;
  model sensitive = group hospital / solution ddfm=satterthwaite;
  random surgeon;
  lsmeans group / cl pdiff;
  ods output LSMeans=lsmeans_hospital CovParms=covparms_hosp;
  title "Sensitivity Analysis 1: Mixed Model Adjusting Hospital";
run;

/* Extract Residual Standard Deviation */
data _null_;
  set covparms_hosp;
  if CovParm="Residual" then call symputx("resid_sd_hosp",
sqrt(Estimate));
run;

/* Calculate Cohen's d */
data cohens_d_hosp;
  length label $50;
  set lsmeans_hospital(where=(group in (1,2))
rename=(Estimate=adj_mean StdErr=adj_se));
```

```
  retain mean1 mean2;
  if group=1 then mean1=adj_mean;
  else if group=2 then mean2=adj_mean;
  if _n_=2 then do;
    s_pooled = &resid_sd_hosp;
    cohens_d = (mean1 - mean2) / s_pooled;
    label = "Sensitivity Analysis Adjusting Hospital";
    output;
  end;
  keep label cohens_d mean1 mean2 s_pooled;
run;

proc print data=cohens_d_hosp noobs label;
  title "Cohen's d from Sensitivity Analysis Adjusting Hospital";
run;


/* 4. Sensitivity Analysis 2: Adjusting Surgery Type (Mixed Model) */
proc mixed data=sensitive_data method=reml noclprint;
  class group surgeon surgery_type;
  model sensitive = group surgery_type / solution ddfm=satterthwaite;
  random surgeon;
  lsmeans group / cl pdiff;
  ods output LSMeans=lsmeans_surgtype CovParms=covparms_surgtype;
  title "Sensitivity Analysis 2: Mixed Model Adjusting Surgery Type";
run;

/* Extract Residual Standard Deviation */
data _null_;
  set covparms_surgtype;
  if CovParm="Residual" then call symputx("resid_sd_surgtype",
sqrt(Estimate));
run;

/* Calculate Cohen's d */
data cohens_d_surgtype;
  length label $50;
  set lsmeans_surgtype(where=(group in (1,2))
rename=(Estimate=adj_mean StdErr=adj_se));
  retain mean1 mean2;
  if group=1 then mean1=adj_mean;
  else if group=2 then mean2=adj_mean;
  if _n_=2 then do;
    s_pooled = &resid_sd_surgtype;
```

```
    cohens_d = (mean1 - mean2) / s_pooled;
    label = "Sensitivity Analysis Adjusting Surgery Type";
    output;
  end;
  keep label cohens_d mean1 mean2 s_pooled;
run;

proc print data=cohens_d_surgtype noobs label;
  title "Cohen's d from Sensitivity Analysis Adjusting Surgery Type";
run;


/* 5. Sensitivity Analysis 3: Adjusting Surgeon (fixed effect) */
proc mixed data=sensitive_data method=reml noclprint;
  class group surgeon;
  model sensitive = group surgeon / solution ddfm=satterthwaite;
  lsmeans group / cl pdiff;
  ods output LSMeans=lsmeans_surg_fixed CovParms=covparms_surg_fixed;
  title "Sensitivity Analysis 3: Mixed Model Adjusting Surgeon (as
fixed effect)";
run;

/* Extract Residual Standard Deviation */
data _null_;
  set covparms_surg_fixed;
  if CovParm="Residual" then call symputx("resid_sd_surg_fixed",
sqrt(Estimate));
run;

/* Calculate Cohen's d */
data cohens_d_surg_fixed;
  length label $50;
  set lsmeans_surg_fixed(where=(group in (1,2))
rename=(Estimate=adj_mean StdErr=adj_se));
  retain mean1 mean2;
  if group=1 then mean1=adj_mean;
  else if group=2 then mean2=adj_mean;
  if _n_=2 then do;
    s_pooled = &resid_sd_surg_fixed;
    cohens_d = (mean1 - mean2) / s_pooled;
    label = "Sensitivity Analysis Adjusting Surgeon (fixed effect)";
    output;
  end;
  keep label cohens_d mean1 mean2 s_pooled;
```

```
run;

proc print data=cohens_d_surg_fixed noobs label;
  title "Cohen's d from Sensitivity Analysis Adjusting Surgeon
(fixed)";
run;
```

3.6.2 For Renal tumor patients

3.6.2.1 Baseline

```
/* Data Input */
/* Data details:
   group: 1=telesurgery group, 2=local surgery group
   hospital: 1=Beijing, 2=Harbin, 3=Hangzhou, 4=Hefei, 5=Urumqi
   surgeon codes: 1=Chaozhao Liang, 2=Baojun Wang, 3=Xu Zhang,
4=Sheng Tai,
                  5=Hongzhao Li, 6=Wanghai Xu, 7=Xin Ma, 8=Qingbo
Huang,
                  9=Weijun Fu, 10=Shuo Wang, 11=Mulati Rexiati
*/
data sensitive_data;
input group hospital surgeon sensitive;
datalines;
1   3   7   149
2   3   10  150
2   3   10  145
1   3   7   150
2   3   10  150
1   3   7   146
2   3   10  150
1   3   7   136
2   4   1   134
2   4   1   138
1   4   7   121
1   5   7   149
2   5   8   146
2   5   8   150
1   5   8   150
2   1   7   149
2   1   7   147
1   1   6   150
1   1   1   134
1   1   1   142
```

```
1   1   10  132
2   1   7   150
2   1   7   149
1   1   8   150
2   1   7   150
1   1   8   150
2   2   6   150
2   2   6   146
1   2   7   143
1   2   8   149
;
run;


/* 2. Main Analysis: Mixed Model (Surgeon as Random Effect) */
proc mixed data=sensitive_data method=reml noclprint;
  class group surgeon;
  model sensitive = group / solution ddfm=satterthwaite;
  random surgeon;
  lsmeans group / cl pdiff;
  ods output LSMeans=lsmeans_mixed CovParms=covparms;
  title "Main Analysis: Mixed Effects Model with Surgeon as Random";
run;

/* Extract Residual Standard Deviation */
data _null_;
  set covparms;
  if CovParm="Residual" then call symputx("resid_sd_mixed",
sqrt(Estimate));
run;

/* Calculate Cohen's d */
data cohens_d_mixed;
  length label $50;
  set lsmeans_mixed(where=(group in (1,2)) rename=(Estimate=adj_mean
StdErr=adj_se));
  retain mean1 mean2;
  if group=1 then mean1=adj_mean;
  else if group=2 then mean2=adj_mean;
  if _n_=2 then do;
    s_pooled = &resid_sd_mixed;
    cohens_d = (mean1 - mean2) / s_pooled;
    label = "Main Analysis (Mixed Model)";
    output;
```

```
  end;
  keep label cohens_d mean1 mean2 s_pooled;
run;

proc print data=cohens_d_mixed noobs label;
  title "Cohen's d from Main Analysis (Mixed Effects Model)";
run;


/* 3. Sensitivity Analysis 1: Adjusting Hospital (Mixed Model) */
proc mixed data=sensitive_data method=reml noclprint;
  class group surgeon hospital;
  model sensitive = group hospital / solution ddfm=satterthwaite;
  random surgeon;
  lsmeans group / cl pdiff;
  ods output LSMeans=lsmeans_hospital CovParms=covparms_hosp;
  title "Sensitivity Analysis 1: Mixed Model Adjusting Hospital";
run;

/* Extract Residual Standard Deviation */
data _null_;
  set covparms_hosp;
  if CovParm="Residual" then call symputx("resid_sd_hosp",
sqrt(Estimate));
run;

/* Calculate Cohen's d */
data cohens_d_hosp;
  length label $50;
  set lsmeans_hospital(where=(group in (1,2))
rename=(Estimate=adj_mean StdErr=adj_se));
  retain mean1 mean2;
  if group=1 then mean1=adj_mean;
  else if group=2 then mean2=adj_mean;
  if _n_=2 then do;
    s_pooled = &resid_sd_hosp;
    cohens_d = (mean1 - mean2) / s_pooled;
    label = "Sensitivity Analysis Adjusting Hospital";
    output;
  end;
  keep label cohens_d mean1 mean2 s_pooled;
run;

proc print data=cohens_d_hosp noobs label;
```

```
  title "Cohen's d from Sensitivity Analysis Adjusting Hospital";
run;


/* 4. Sensitivity Analysis 3: Adjusting Surgeon (fixed effect) */
proc mixed data=sensitive_data method=reml noclprint;
  class group surgeon;
  model sensitive = group surgeon / solution ddfm=satterthwaite;
  lsmeans group / cl pdiff;
  ods output LSMeans=lsmeans_surg_fixed CovParms=covparms_surg_fixed;
  title "Sensitivity Analysis 3: Mixed Model Adjusting Surgeon (as
fixed effect)";
run;

/* Extract Residual Standard Deviation */
data _null_;
  set covparms_surg_fixed;
  if CovParm="Residual" then call symputx("resid_sd_surg_fixed",
sqrt(Estimate));
run;

/* Calculate Cohen's d */
data cohens_d_surg_fixed;
  length label $50;
  set lsmeans_surg_fixed(where=(group in (1,2))
rename=(Estimate=adj_mean StdErr=adj_se));
  retain mean1 mean2;
  if group=1 then mean1=adj_mean;
  else if group=2 then mean2=adj_mean;
  if _n_=2 then do;
    s_pooled = &resid_sd_surg_fixed;
    cohens_d = (mean1 - mean2) / s_pooled;
    label = "Sensitivity Analysis Adjusting Surgeon (fixed effect)";
    output;
  end;
  keep label cohens_d mean1 mean2 s_pooled;
run;

proc print data=cohens_d_surg_fixed noobs label;
  title "Cohen's d from Sensitivity Analysis Adjusting Surgeon
(fixed)";
run;
```

3.6.2.2 At 4w follow-up point

```
/* Data Input */
/* Data details:
   group: 1=telesurgery group, 2=local surgery group
   hospital: 1=Beijing, 2=Harbin, 3=Hangzhou, 4=Hefei, 5=Urumqi
   surgeon codes: 1=Chaozhao Liang, 2=Baojun Wang, 3=Xu Zhang,
4=Sheng Tai,
                  5=Hongzhao Li, 6=Wanghai Xu, 7=Xin Ma, 8=Qingbo
Huang,
                  9=Weijun Fu, 10=Shuo Wang, 11=Mulati Rexiati
*/
data sensitive_data;
input group hospital surgeon sensitive;
datalines;
1   3   7   149
2   3   10  150
2   3   10  145
1   3   7   150
2   3   10  150
1   3   7   146
2   3   10  150
1   3   7   136
2   4   1   134
2   4   1   138
1   4   7   121
1   5   7   149
2   5   8   146
2   5   8   150
1   5   8   150
2   1   7   149
2   1   7   147
1   1   6   150
1   1   1   134
1   1   1   142
1   1   10  132
2   1   7   150
2   1   7   149
1   1   8   150
2   1   7   150
1   1   8   150
2   2   6   150
2   2   6   146
1   2   7   143
1   2   8   149
```

```
;
run;


/* 2. Main Analysis: Mixed Model (Surgeon as Random Effect) */
proc mixed data=sensitive_data method=reml noclprint;
  class group surgeon;
  model sensitive = group / solution ddfm=satterthwaite;
  random surgeon;
  lsmeans group / cl pdiff;
  ods output LSMeans=lsmeans_mixed CovParms=covparms;
  title "Main Analysis: Mixed Effects Model with Surgeon as Random";
run;

/* Extract Residual Standard Deviation */
data _null_;
  set covparms;
  if CovParm="Residual" then call symputx("resid_sd_mixed",
sqrt(Estimate));
run;

/* Calculate Cohen's d */
data cohens_d_mixed;
  length label $50;
  set lsmeans_mixed(where=(group in (1,2)) rename=(Estimate=adj_mean
StdErr=adj_se));
  retain mean1 mean2;
  if group=1 then mean1=adj_mean;
  else if group=2 then mean2=adj_mean;
  if _n_=2 then do;
    s_pooled = &resid_sd_mixed;
    cohens_d = (mean1 - mean2) / s_pooled;
    label = "Main Analysis (Mixed Model)";
    output;
  end;
  keep label cohens_d mean1 mean2 s_pooled;
run;

proc print data=cohens_d_mixed noobs label;
  title "Cohen's d from Main Analysis (Mixed Effects Model)";
run;


/* 3. Sensitivity Analysis 1: Adjusting Hospital (Mixed Model) */
```

```
proc mixed data=sensitive_data method=reml noclprint;
  class group surgeon hospital;
  model sensitive = group hospital / solution ddfm=satterthwaite;
  random surgeon;
  lsmeans group / cl pdiff;
  ods output LSMeans=lsmeans_hospital CovParms=covparms_hosp;
  title "Sensitivity Analysis 1: Mixed Model Adjusting Hospital";
run;

/* Extract Residual Standard Deviation */
data _null_;
  set covparms_hosp;
  if CovParm="Residual" then call symputx("resid_sd_hosp",
sqrt(Estimate));
run;

/* Calculate Cohen's d */
data cohens_d_hosp;
  length label $50;
  set lsmeans_hospital(where=(group in (1,2))
rename=(Estimate=adj_mean StdErr=adj_se));
  retain mean1 mean2;
  if group=1 then mean1=adj_mean;
  else if group=2 then mean2=adj_mean;
  if _n_=2 then do;
    s_pooled = &resid_sd_hosp;
    cohens_d = (mean1 - mean2) / s_pooled;
    label = "Sensitivity Analysis Adjusting Hospital";
    output;
  end;
  keep label cohens_d mean1 mean2 s_pooled;
run;

proc print data=cohens_d_hosp noobs label;
  title "Cohen's d from Sensitivity Analysis Adjusting Hospital";
run;


/* 4. Sensitivity Analysis 3: Adjusting Surgeon (fixed effect) */
proc mixed data=sensitive_data method=reml noclprint;
  class group surgeon;
  model sensitive = group surgeon / solution ddfm=satterthwaite;
  lsmeans group / cl pdiff;
  ods output LSMeans=lsmeans_surg_fixed CovParms=covparms_surg_fixed;
```

```
  title "Sensitivity Analysis 3: Mixed Model Adjusting Surgeon (as
fixed effect)";
run;

/* Extract Residual Standard Deviation */
data _null_;
  set covparms_surg_fixed;
  if CovParm="Residual" then call symputx("resid_sd_surg_fixed",
sqrt(Estimate));
run;

/* Calculate Cohen's d */
data cohens_d_surg_fixed;
  length label $50;
  set lsmeans_surg_fixed(where=(group in (1,2))
rename=(Estimate=adj_mean StdErr=adj_se));
  retain mean1 mean2;
  if group=1 then mean1=adj_mean;
  else if group=2 then mean2=adj_mean;
  if _n_=2 then do;
    s_pooled = &resid_sd_surg_fixed;
    cohens_d = (mean1 - mean2) / s_pooled;
    label = "Sensitivity Analysis Adjusting Surgeon (fixed effect)";
    output;
  end;
  keep label cohens_d mean1 mean2 s_pooled;
run;

proc print data=cohens_d_surg_fixed noobs label;
  title "Cohen's d from Sensitivity Analysis Adjusting Surgeon
(fixed)";
run;
```

3.6.2.3 At 6w follow-up point

```
/* Data Input */
/* Data details:
   group: 1=telesurgery group, 2=local surgery group
   hospital: 1=Beijing, 2=Harbin, 3=Hangzhou, 4=Hefei, 5=Urumqi
   surgeon codes: 1=Chaozhao Liang, 2=Baojun Wang, 3=Xu Zhang,
4=Sheng Tai,
                  5=Hongzhao Li, 6=Wanghai Xu, 7=Xin Ma, 8=Qingbo
Huang,
                  9=Weijun Fu, 10=Shuo Wang, 11=Mulati Rexiati
```

```
*/
data sensitive_data;
input group hospital surgeon sensitive;
datalines;
1   3   7   123
2   3   10  150
2   3   10  138
1   3   7   149
2   3   10  150
1   3   7   150
2   3   10  145
1   3   7   147
2   4   1
2   4   1
1   4   7
1   5   7   150
2   5   8   149
2   5   8   150
1   5   8   150
2   1   7   150
2   1   7
1   1   6   149
1   1   1   147
1   1   1   150
1   1   10  136
2   1   7   142
2   1   7   150
1   1   8   126
2   1   7   148
1   1   8   150
2   2   6   150
2   2   6   145
1   2   7   146
1   2   8   146
;
run;


/* 2. Main Analysis: Mixed Model (Surgeon as Random Effect) */
proc mixed data=sensitive_data method=reml noclprint;
  class group surgeon;
  model sensitive = group / solution ddfm=satterthwaite;
  random surgeon;
  lsmeans group / cl pdiff;
```

```
  ods output LSMeans=lsmeans_mixed CovParms=covparms;
  title "Main Analysis: Mixed Effects Model with Surgeon as Random";
run;

/* Extract Residual Standard Deviation */
data _null_;
  set covparms;
  if CovParm="Residual" then call symputx("resid_sd_mixed",
sqrt(Estimate));
run;

/* Calculate Cohen's d */
data cohens_d_mixed;
  length label $50;
  set lsmeans_mixed(where=(group in (1,2)) rename=(Estimate=adj_mean
StdErr=adj_se));
  retain mean1 mean2;
  if group=1 then mean1=adj_mean;
  else if group=2 then mean2=adj_mean;
  if _n_=2 then do;
    s_pooled = &resid_sd_mixed;
    cohens_d = (mean1 - mean2) / s_pooled;
    label = "Main Analysis (Mixed Model)";
    output;
  end;
  keep label cohens_d mean1 mean2 s_pooled;
run;

proc print data=cohens_d_mixed noobs label;
  title "Cohen's d from Main Analysis (Mixed Effects Model)";
run;


/* 3. Sensitivity Analysis 1: Adjusting Hospital (Mixed Model) */
proc mixed data=sensitive_data method=reml noclprint;
  class group surgeon hospital;
  model sensitive = group hospital / solution ddfm=satterthwaite;
  random surgeon;
  lsmeans group / cl pdiff;
  ods output LSMeans=lsmeans_hospital CovParms=covparms_hosp;
  title "Sensitivity Analysis 1: Mixed Model Adjusting Hospital";
run;

/* Extract Residual Standard Deviation */
```

```
data _null_;
  set covparms_hosp;
  if CovParm="Residual" then call symputx("resid_sd_hosp",
sqrt(Estimate));
run;

/* Calculate Cohen's d */
data cohens_d_hosp;
  length label $50;
  set lsmeans_hospital(where=(group in (1,2))
rename=(Estimate=adj_mean StdErr=adj_se));
  retain mean1 mean2;
  if group=1 then mean1=adj_mean;
  else if group=2 then mean2=adj_mean;
  if _n_=2 then do;
    s_pooled = &resid_sd_hosp;
    cohens_d = (mean1 - mean2) / s_pooled;
    label = "Sensitivity Analysis Adjusting Hospital";
    output;
  end;
  keep label cohens_d mean1 mean2 s_pooled;
run;

proc print data=cohens_d_hosp noobs label;
  title "Cohen's d from Sensitivity Analysis Adjusting Hospital";
run;


/* 4. Sensitivity Analysis 3: Adjusting Surgeon (fixed effect) */
proc mixed data=sensitive_data method=reml noclprint;
  class group surgeon;
  model sensitive = group surgeon / solution ddfm=satterthwaite;
  lsmeans group / cl pdiff;
  ods output LSMeans=lsmeans_surg_fixed CovParms=covparms_surg_fixed;
  title "Sensitivity Analysis 3: Mixed Model Adjusting Surgeon (as
fixed effect)";
run;

/* Extract Residual Standard Deviation */
data _null_;
  set covparms_surg_fixed;
  if CovParm="Residual" then call symputx("resid_sd_surg_fixed",
sqrt(Estimate));
run;
```

```
/* Calculate Cohen's d */
data cohens_d_surg_fixed;
  length label $50;
  set lsmeans_surg_fixed(where=(group in (1,2))
rename=(Estimate=adj_mean StdErr=adj_se));
  retain mean1 mean2;
  if group=1 then mean1=adj_mean;
  else if group=2 then mean2=adj_mean;
  if _n_=2 then do;
    s_pooled = &resid_sd_surg_fixed;
    cohens_d = (mean1 - mean2) / s_pooled;
    label = "Sensitivity Analysis Adjusting Surgeon (fixed effect)";
    output;
  end;
  keep label cohens_d mean1 mean2 s_pooled;
run;

proc print data=cohens_d_surg_fixed noobs label;
  title "Cohen's d from Sensitivity Analysis Adjusting Surgeon
(fixed)";

run;
```

3.6.3 For Prostate cancer patients

3.6.3.1 Baseline

```
/* Data Input */
/* Data details:
   group: 1=telesurgery group, 2=local surgery group
   hospital: 1=Beijing, 2=Harbin, 3=Hangzhou, 4=Hefei, 5=Urumqi
   surgeon codes: 1=Chaozhao Liang, 2=Baojun Wang, 3=Xu Zhang,
4=Sheng Tai,
                  5=Hongzhao Li, 6=Wanghai Xu, 7=Xin Ma, 8=Qingbo
Huang,
                  9=Weijun Fu, 10=Shuo Wang, 11=Mulati Rexiati
*/
data sensitive_data;
input group hospital surgeon sensitive;
datalines;
2   3   10  139
1   3   5   146
2   3   10  148
2   3   10  139
```

```
1   3   5   150
2   3   10  150
1   4   2   130
2   4   4   147
2   4   4   145
1   4   3   150
1   4   5   141
2   4   1   137
1   5   5   145
1   5   5   145
1   5   5   150
1   5   2   150
2   5   2   150
2   5   11  150
2   1   9   148
1   1   6   150
2   1   9   141
1   1   1   150
2   1   3   146
2   1   9   143
1   1   10  131
1   1   10  147
1   1   10  138
2   1   9   150
1   1   9   144
2   1   9   150
1   2   5   148
2   2   6   150
1   2   7   149
;
run;


/* 2. Main Analysis: Mixed Model (Surgeon as Random Effect) */
proc mixed data=sensitive_data method=reml noclprint;
  class group surgeon;
  model sensitive = group / solution ddfm=satterthwaite;
  random surgeon;
  lsmeans group / cl pdiff;
  ods output LSMeans=lsmeans_mixed CovParms=covparms;
  title "Main Analysis: Mixed Effects Model with Surgeon as Random";
run;

/* Extract Residual Standard Deviation */
```

```
data _null_;
  set covparms;
  if CovParm="Residual" then call symputx("resid_sd_mixed",
sqrt(Estimate));
run;

/* Calculate Cohen's d */
data cohens_d_mixed;
  length label $50;
  set lsmeans_mixed(where=(group in (1,2)) rename=(Estimate=adj_mean
StdErr=adj_se));
  retain mean1 mean2;
  if group=1 then mean1=adj_mean;
  else if group=2 then mean2=adj_mean;
  if _n_=2 then do;
    s_pooled = &resid_sd_mixed;
    cohens_d = (mean1 - mean2) / s_pooled;
    label = "Main Analysis (Mixed Model)";
    output;
  end;
  keep label cohens_d mean1 mean2 s_pooled;
run;

proc print data=cohens_d_mixed noobs label;
  title "Cohen's d from Main Analysis (Mixed Effects Model)";
run;


/* 3. Sensitivity Analysis 1: Adjusting Hospital (Mixed Model) */
proc mixed data=sensitive_data method=reml noclprint;
  class group surgeon hospital;
  model sensitive = group hospital / solution ddfm=satterthwaite;
  random surgeon;
  lsmeans group / cl pdiff;
  ods output LSMeans=lsmeans_hospital CovParms=covparms_hosp;
  title "Sensitivity Analysis 1: Mixed Model Adjusting Hospital";
run;

/* Extract Residual Standard Deviation */
data _null_;
  set covparms_hosp;
  if CovParm="Residual" then call symputx("resid_sd_hosp",
sqrt(Estimate));
run;
```

```
/* Calculate Cohen's d */
data cohens_d_hosp;
  length label $50;
  set lsmeans_hospital(where=(group in (1,2))
rename=(Estimate=adj_mean StdErr=adj_se));
  retain mean1 mean2;
  if group=1 then mean1=adj_mean;
  else if group=2 then mean2=adj_mean;
  if _n_=2 then do;
    s_pooled = &resid_sd_hosp;
    cohens_d = (mean1 - mean2) / s_pooled;
    label = "Sensitivity Analysis Adjusting Hospital";
    output;
  end;
  keep label cohens_d mean1 mean2 s_pooled;
run;

proc print data=cohens_d_hosp noobs label;
  title "Cohen's d from Sensitivity Analysis Adjusting Hospital";
run;


/* 4. Sensitivity Analysis 3: Adjusting Surgeon (fixed effect) */
proc mixed data=sensitive_data method=reml noclprint;
  class group surgeon;
  model sensitive = group surgeon / solution ddfm=satterthwaite;
  lsmeans group / cl pdiff;
  ods output LSMeans=lsmeans_surg_fixed CovParms=covparms_surg_fixed;
  title "Sensitivity Analysis 3: Mixed Model Adjusting Surgeon (as
fixed effect)";
run;

/* Extract Residual Standard Deviation */
data _null_;
  set covparms_surg_fixed;
  if CovParm="Residual" then call symputx("resid_sd_surg_fixed",
sqrt(Estimate));
run;

/* Calculate Cohen's d */
data cohens_d_surg_fixed;
  length label $50;
  set lsmeans_surg_fixed(where=(group in (1,2))
```

```
rename=(Estimate=adj_mean StdErr=adj_se));
  retain mean1 mean2;
  if group=1 then mean1=adj_mean;
  else if group=2 then mean2=adj_mean;
  if _n_=2 then do;
    s_pooled = &resid_sd_surg_fixed;
    cohens_d = (mean1 - mean2) / s_pooled;
    label = "Sensitivity Analysis Adjusting Surgeon (fixed effect)";
    output;
  end;
  keep label cohens_d mean1 mean2 s_pooled;
run;

proc print data=cohens_d_surg_fixed noobs label;
  title "Cohen's d from Sensitivity Analysis Adjusting Surgeon
(fixed)";
run;
```

3.6.3.2 At 4w follow-up point

```
/* Data Input */
/* Data details:
   group: 1=telesurgery group, 2=local surgery group
   hospital: 1=Beijing, 2=Harbin, 3=Hangzhou, 4=Hefei, 5=Urumqi
   surgeon codes: 1=Chaozhao Liang, 2=Baojun Wang, 3=Xu Zhang,
4=Sheng Tai,
                  5=Hongzhao Li, 6=Wanghai Xu, 7=Xin Ma, 8=Qingbo
Huang,
                  9=Weijun Fu, 10=Shuo Wang, 11=Mulati Rexiati
*/
data sensitive_data;
input group hospital surgeon sensitive;
datalines;
2   3   10  137
1   3   5   148
2   3   10  150
2   3   10  150
1   3   5   150
2   3   10  150
1   4   2   135
2   4   4   138
2   4   4   131
1   4   3   121
1   4   5   137
```

```
2   4   1   140
1   5   5   149
1   5   5   148
1   5   5   150
1   5   2   149
2   5   2   150
2   5   11  149
2   1   9   145
1   1   6   144
2   1   9   118
1   1   1   140
2   1   3   145
2   1   9   141
1   1   10  147
1   1   10  148
1   1   10  137
2   1   9   145
1   1   9   148
2   1   9   147
1   2   5   123
2   2   6   129
1   2   7   135
;
run;


/* 2. Main Analysis: Mixed Model (Surgeon as Random Effect) */
proc mixed data=sensitive_data method=reml noclprint;
  class group surgeon;
  model sensitive = group / solution ddfm=satterthwaite;
  random surgeon;
  lsmeans group / cl pdiff;
  ods output LSMeans=lsmeans_mixed CovParms=covparms;
  title "Main Analysis: Mixed Effects Model with Surgeon as Random";
run;

/* Extract Residual Standard Deviation */
data _null_;
  set covparms;
  if CovParm="Residual" then call symputx("resid_sd_mixed",
sqrt(Estimate));
run;

/* Calculate Cohen's d */
```

```
data cohens_d_mixed;
  length label $50;
  set lsmeans_mixed(where=(group in (1,2)) rename=(Estimate=adj_mean
StdErr=adj_se));
  retain mean1 mean2;
  if group=1 then mean1=adj_mean;
  else if group=2 then mean2=adj_mean;
  if _n_=2 then do;
    s_pooled = &resid_sd_mixed;
    cohens_d = (mean1 - mean2) / s_pooled;
    label = "Main Analysis (Mixed Model)";
    output;
  end;
  keep label cohens_d mean1 mean2 s_pooled;
run;

proc print data=cohens_d_mixed noobs label;
  title "Cohen's d from Main Analysis (Mixed Effects Model)";
run;


/* 3. Sensitivity Analysis 1: Adjusting Hospital (Mixed Model) */
proc mixed data=sensitive_data method=reml noclprint;
  class group surgeon hospital;
  model sensitive = group hospital / solution ddfm=satterthwaite;
  random surgeon;
  lsmeans group / cl pdiff;
  ods output LSMeans=lsmeans_hospital CovParms=covparms_hosp;
  title "Sensitivity Analysis 1: Mixed Model Adjusting Hospital";
run;

/* Extract Residual Standard Deviation */
data _null_;
  set covparms_hosp;
  if CovParm="Residual" then call symputx("resid_sd_hosp",
sqrt(Estimate));
run;

/* Calculate Cohen's d */
data cohens_d_hosp;
  length label $50;
  set lsmeans_hospital(where=(group in (1,2))
rename=(Estimate=adj_mean StdErr=adj_se));
  retain mean1 mean2;
```

```
  if group=1 then mean1=adj_mean;
  else if group=2 then mean2=adj_mean;
  if _n_=2 then do;
    s_pooled = &resid_sd_hosp;
    cohens_d = (mean1 - mean2) / s_pooled;
    label = "Sensitivity Analysis Adjusting Hospital";
    output;
  end;
  keep label cohens_d mean1 mean2 s_pooled;
run;

proc print data=cohens_d_hosp noobs label;
  title "Cohen's d from Sensitivity Analysis Adjusting Hospital";
run;


/* 4. Sensitivity Analysis 3: Adjusting Surgeon (fixed effect) */
proc mixed data=sensitive_data method=reml noclprint;
  class group surgeon;
  model sensitive = group surgeon / solution ddfm=satterthwaite;
  lsmeans group / cl pdiff;
  ods output LSMeans=lsmeans_surg_fixed CovParms=covparms_surg_fixed;
  title "Sensitivity Analysis 3: Mixed Model Adjusting Surgeon (as
fixed effect)";
run;

/* Extract Residual Standard Deviation */
data _null_;
  set covparms_surg_fixed;
  if CovParm="Residual" then call symputx("resid_sd_surg_fixed",
sqrt(Estimate));
run;

/* Calculate Cohen's d */
data cohens_d_surg_fixed;
  length label $50;
  set lsmeans_surg_fixed(where=(group in (1,2))
rename=(Estimate=adj_mean StdErr=adj_se));
  retain mean1 mean2;
  if group=1 then mean1=adj_mean;
  else if group=2 then mean2=adj_mean;
  if _n_=2 then do;
    s_pooled = &resid_sd_surg_fixed;
    cohens_d = (mean1 - mean2) / s_pooled;
```

```
    label = "Sensitivity Analysis Adjusting Surgeon (fixed effect)";
    output;
  end;
  keep label cohens_d mean1 mean2 s_pooled;
run;

proc print data=cohens_d_surg_fixed noobs label;
  title "Cohen's d from Sensitivity Analysis Adjusting Surgeon
(fixed)";
run;
```

3.6.3.3 At 6w follow-up point

```
/* Data Input */
/* Data details:
   group: 1=telesurgery group, 2=local surgery group
   hospital: 1=Beijing, 2=Harbin, 3=Hangzhou, 4=Hefei, 5=Urumqi
   surgeon codes: 1=Chaozhao Liang, 2=Baojun Wang, 3=Xu Zhang,
4=Sheng Tai,
                  5=Hongzhao Li, 6=Wanghai Xu, 7=Xin Ma, 8=Qingbo
Huang,
                  9=Weijun Fu, 10=Shuo Wang, 11=Mulati Rexiati
*/
data sensitive_data;
input group hospital surgeon sensitive;
datalines;
2   3   10  146
1   3   5   148
2   3   10  150
2   3   10  150
1   3   5   150
2   3   10  150
1   4   2   141
2   4   4   140
2   4   4   131
1   4   3   139
1   4   5   138
2   4   1
1   5   5   150
1   5   5   149
1   5   5   150
1   5   2   150
2   5   2   150
2   5   11  150
```

```
2   1   9   144
1   1   6   130
2   1   9   135
1   1   1   132
2   1   3   144
2   1   9   132
1   1   10  143
1   1   10  147
1   1   10  137
2   1   9   150
1   1   9   145
2   1   9   147
1   2   5   143
2   2   6   140
1   2   7   144
;
run;


/* 2. Main Analysis: Mixed Model (Surgeon as Random Effect) */
proc mixed data=sensitive_data method=reml noclprint;
  class group surgeon;
  model sensitive = group / solution ddfm=satterthwaite;
  random surgeon;
  lsmeans group / cl pdiff;
  ods output LSMeans=lsmeans_mixed CovParms=covparms;
  title "Main Analysis: Mixed Effects Model with Surgeon as Random";
run;

/* Extract Residual Standard Deviation */
data _null_;
  set covparms;
  if CovParm="Residual" then call symputx("resid_sd_mixed",
sqrt(Estimate));
run;

/* Calculate Cohen's d */
data cohens_d_mixed;
  length label $50;
  set lsmeans_mixed(where=(group in (1,2)) rename=(Estimate=adj_mean
StdErr=adj_se));
  retain mean1 mean2;
  if group=1 then mean1=adj_mean;
  else if group=2 then mean2=adj_mean;
```

```
  if _n_=2 then do;
    s_pooled = &resid_sd_mixed;
    cohens_d = (mean1 - mean2) / s_pooled;
    label = "Main Analysis (Mixed Model)";
    output;
  end;
  keep label cohens_d mean1 mean2 s_pooled;
run;

proc print data=cohens_d_mixed noobs label;
  title "Cohen's d from Main Analysis (Mixed Effects Model)";
run;


/* 3. Sensitivity Analysis 1: Adjusting Hospital (Mixed Model) */
proc mixed data=sensitive_data method=reml noclprint;
  class group surgeon hospital;
  model sensitive = group hospital / solution ddfm=satterthwaite;
  random surgeon;
  lsmeans group / cl pdiff;
  ods output LSMeans=lsmeans_hospital CovParms=covparms_hosp;
  title "Sensitivity Analysis 1: Mixed Model Adjusting Hospital";
run;

/* Extract Residual Standard Deviation */
data _null_;
  set covparms_hosp;
  if CovParm="Residual" then call symputx("resid_sd_hosp",
sqrt(Estimate));
run;

/* Calculate Cohen's d */
data cohens_d_hosp;
  length label $50;
  set lsmeans_hospital(where=(group in (1,2))
rename=(Estimate=adj_mean StdErr=adj_se));
  retain mean1 mean2;
  if group=1 then mean1=adj_mean;
  else if group=2 then mean2=adj_mean;
  if _n_=2 then do;
    s_pooled = &resid_sd_hosp;
    cohens_d = (mean1 - mean2) / s_pooled;
    label = "Sensitivity Analysis Adjusting Hospital";
    output;
```

```
  end;
  keep label cohens_d mean1 mean2 s_pooled;
run;

proc print data=cohens_d_hosp noobs label;
  title "Cohen's d from Sensitivity Analysis Adjusting Hospital";
run;


/* 4. Sensitivity Analysis 3: Adjusting Surgeon (fixed effect) */
proc mixed data=sensitive_data method=reml noclprint;
  class group surgeon;
  model sensitive = group surgeon / solution ddfm=satterthwaite;
  lsmeans group / cl pdiff;
  ods output LSMeans=lsmeans_surg_fixed CovParms=covparms_surg_fixed;
  title "Sensitivity Analysis 3: Mixed Model Adjusting Surgeon (as
fixed effect)";
run;

/* Extract Residual Standard Deviation */
data _null_;
  set covparms_surg_fixed;
  if CovParm="Residual" then call symputx("resid_sd_surg_fixed",
sqrt(Estimate));
run;

/* Calculate Cohen's d */
data cohens_d_surg_fixed;
  length label $50;
  set lsmeans_surg_fixed(where=(group in (1,2))
rename=(Estimate=adj_mean StdErr=adj_se));
  retain mean1 mean2;
  if group=1 then mean1=adj_mean;
  else if group=2 then mean2=adj_mean;
  if _n_=2 then do;
    s_pooled = &resid_sd_surg_fixed;
    cohens_d = (mean1 - mean2) / s_pooled;
    label = "Sensitivity Analysis Adjusting Surgeon (fixed effect)";
    output;
  end;
  keep label cohens_d mean1 mean2 s_pooled;
run;

proc print data=cohens_d_surg_fixed noobs label;
```

```
  title "Cohen's d from Sensitivity Analysis Adjusting Surgeon
(fixed)";
run;
```

3.7 '30 Second Chair to Stand test

3.7.1 Total for renal tumor and prostate cancer patients

3.7.1.1 Baseline

```
/* Data Input */
/* Data details:
   group: 1=telesurgery group, 2=local surgery group
   hospital: 1=Beijing, 2=Harbin, 3=Hangzhou, 4=Hefei, 5=Urumqi
   surgery_type: 1=Radical Prostatectomy, 2=Partial Nephrectomy
   surgeon codes: 1=Chaozhao Liang, 2=Baojun Wang, 3=Xu Zhang,
4=Sheng Tai,
                  5=Hongzhao Li, 6=Wanghai Xu, 7=Xin Ma, 8=Qingbo
Huang,
                  9=Weijun Fu, 10=Shuo Wang, 11=Mulati Rexiati
*/
data sensitive_data;
input group hospital surgery_type surgeon sensitive;
datalines;
2   3   1   10  21
1   3   1   5   29
2   3   1   10  30
2   3   1   10  10
1   3   1   5   17
2   3   1   10  18
1   3   2   7   9
2   3   2   10  20
2   3   2   10  19
1   3   2   7   12
2   3   2   10  15
1   3   2   7   14
2   3   2   10  20
1   3   2   7   23
1   4   1   2   15
2   4   1   4   20
2   4   1   4   12
1   4   1   3   16
1   4   1   5   15
2   4   1   1   14
```

2	4	2	1	16
2	4	2	1	17
1	4	2	7	13
1	5	1	5	13
1	5	1	5	17
1	5	1	5	16
1	5	1	2	12
2	5	1	2	16
2	5	1	11	14
1	5	2	7	15
2	5	2	8	14
2	5	2	8	14
1	5	2	8	16
2	1	1	9	12
1	1	1	6	15
2	1	1	9	15
1	1	1	1	15
2	1	1	3	13
2	1	1	9	15
1	1	1	10	4
1	1	1	10	10
1	1	1	10	16
2	1	1	9	12
1	1	1	9	15
2	1	1	9	28
2	1	2	7	14
2	1	2	7	14
1	1	2	6	15
1	1	2	1	8
1	1	2	1	12
1	1	2	10	15
2	1	2	7	16
2	1	2	7	14
1	1	2	8	13
2	1	2	7	12
1	1	2	8	10
1	2	1	5	14
2	2	1	6	13
1	2	1	7	8
2	2	2	6	12
2	2	2	6	16
1	2	2	7	16
1	2	2	8	15

;

```
run;


/* 2. Main Analysis: Mixed Model (Surgeon as Random Effect) */
proc mixed data=sensitive_data method=reml noclprint;
  class group surgeon;
  model sensitive = group / solution ddfm=satterthwaite;
  random surgeon;
  lsmeans group / cl pdiff;
  ods output LSMeans=lsmeans_mixed CovParms=covparms;
  title "Main Analysis: Mixed Effects Model with Surgeon as Random";
run;

/* Extract Residual Standard Deviation */
data _null_;
  set covparms;
  if CovParm="Residual" then call symputx("resid_sd_mixed",
sqrt(Estimate));
run;

/* Calculate Cohen's d */
data cohens_d_mixed;
  length label $50;
  set lsmeans_mixed(where=(group in (1,2)) rename=(Estimate=adj_mean
StdErr=adj_se));
  retain mean1 mean2;
  if group=1 then mean1=adj_mean;
  else if group=2 then mean2=adj_mean;
  if _n_=2 then do;
    s_pooled = &resid_sd_mixed;
    cohens_d = (mean1 - mean2) / s_pooled;
    label = "Main Analysis (Mixed Model)";
    output;
  end;
  keep label cohens_d mean1 mean2 s_pooled;
run;

proc print data=cohens_d_mixed noobs label;
  title "Cohen's d from Main Analysis (Mixed Effects Model)";
run;


/* 3. Sensitivity Analysis 1: Adjusting Hospital (Mixed Model) */
proc mixed data=sensitive_data method=reml noclprint;
```

```
  class group surgeon hospital;
  model sensitive = group hospital / solution ddfm=satterthwaite;
  random surgeon;
  lsmeans group / cl pdiff;
  ods output LSMeans=lsmeans_hospital CovParms=covparms_hosp;
  title "Sensitivity Analysis 1: Mixed Model Adjusting Hospital";
run;

/* Extract Residual Standard Deviation */
data _null_;
  set covparms_hosp;
  if CovParm="Residual" then call symputx("resid_sd_hosp",
sqrt(Estimate));
run;

/* Calculate Cohen's d */
data cohens_d_hosp;
  length label $50;
  set lsmeans_hospital(where=(group in (1,2))
rename=(Estimate=adj_mean StdErr=adj_se));
  retain mean1 mean2;
  if group=1 then mean1=adj_mean;
  else if group=2 then mean2=adj_mean;
  if _n_=2 then do;
    s_pooled = &resid_sd_hosp;
    cohens_d = (mean1 - mean2) / s_pooled;
    label = "Sensitivity Analysis Adjusting Hospital";
    output;
  end;
  keep label cohens_d mean1 mean2 s_pooled;
run;

proc print data=cohens_d_hosp noobs label;
  title "Cohen's d from Sensitivity Analysis Adjusting Hospital";
run;


/* 4. Sensitivity Analysis 2: Adjusting Surgery Type (Mixed Model) */
proc mixed data=sensitive_data method=reml noclprint;
  class group surgeon surgery_type;
  model sensitive = group surgery_type / solution ddfm=satterthwaite;
  random surgeon;
  lsmeans group / cl pdiff;
  ods output LSMeans=lsmeans_surgtype CovParms=covparms_surgtype;
```

```
  title "Sensitivity Analysis 2: Mixed Model Adjusting Surgery Type";
run;

/* Extract Residual Standard Deviation */
data _null_;
  set covparms_surgtype;
  if CovParm="Residual" then call symputx("resid_sd_surgtype",
sqrt(Estimate));
run;

/* Calculate Cohen's d */
data cohens_d_surgtype;
  length label $50;
  set lsmeans_surgtype(where=(group in (1,2))
rename=(Estimate=adj_mean StdErr=adj_se));
  retain mean1 mean2;
  if group=1 then mean1=adj_mean;
  else if group=2 then mean2=adj_mean;
  if _n_=2 then do;
    s_pooled = &resid_sd_surgtype;
    cohens_d = (mean1 - mean2) / s_pooled;
    label = "Sensitivity Analysis Adjusting Surgery Type";
    output;
  end;
  keep label cohens_d mean1 mean2 s_pooled;
run;

proc print data=cohens_d_surgtype noobs label;
  title "Cohen's d from Sensitivity Analysis Adjusting Surgery Type";
run;


/* 5. Sensitivity Analysis 3: Adjusting Surgeon (fixed effect) */
proc mixed data=sensitive_data method=reml noclprint;
  class group surgeon;
  model sensitive = group surgeon / solution ddfm=satterthwaite;
  lsmeans group / cl pdiff;
  ods output LSMeans=lsmeans_surg_fixed CovParms=covparms_surg_fixed;
  title "Sensitivity Analysis 3: Mixed Model Adjusting Surgeon (as
fixed effect)";
run;

/* Extract Residual Standard Deviation */
data _null_;
```

```
  set covparms_surg_fixed;
  if CovParm="Residual" then call symputx("resid_sd_surg_fixed", 
sqrt(Estimate));
run;

/* Calculate Cohen's d */
data cohens_d_surg_fixed;
  length label $50;
  set lsmeans_surg_fixed(where=(group in (1,2)) 
rename=(Estimate=adj_mean StdErr=adj_se));
  retain mean1 mean2;
  if group=1 then mean1=adj_mean;
  else if group=2 then mean2=adj_mean;
  if _n_=2 then do;
    s_pooled = &resid_sd_surg_fixed;
    cohens_d = (mean1 - mean2) / s_pooled;
    label = "Sensitivity Analysis Adjusting Surgeon (fixed effect)";
    output;
  end;
  keep label cohens_d mean1 mean2 s_pooled;
run;

proc print data=cohens_d_surg_fixed noobs label;
  title "Cohen's d from Sensitivity Analysis Adjusting Surgeon 
(fixed)";
run;
```

3.7.1.2 At 4w follow-up point

```
/* Data Input */
/* Data details:
   group: 1=telesurgery group, 2=local surgery group
   hospital: 1=Beijing, 2=Harbin, 3=Hangzhou, 4=Hefei, 5=Urumqi
   surgery_type: 1=Radical Prostatectomy, 2=Partial Nephrectomy
   surgeon codes: 1=Chaozhao Liang, 2=Baojun Wang, 3=Xu Zhang, 
4=Sheng Tai,
                  5=Hongzhao Li, 6=Wanghai Xu, 7=Xin Ma, 8=Qingbo 
Huang,
                  9=Weijun Fu, 10=Shuo Wang, 11=Mulati Rexiati
*/
data sensitive_data;
input group hospital surgery_type surgeon sensitive;
datalines;
2   3   1   10  12
```

1	3	1	5	
2	3	1	10	23
2	3	1	10	
1	3	1	5	
2	3	1	10	20
1	3	2	7	6
2	3	2	10	15
2	3	2	10	13
1	3	2	7	29
2	3	2	10	
1	3	2	7	
2	3	2	10	20
1	3	2	7	25
1	4	1	2	14
2	4	1	4	14
2	4	1	4	14
1	4	1	3	14
1	4	1	5	13
2	4	1	1	13
2	4	2	1	14
2	4	2	1	16
1	4	2	7	12
1	5	1	5	12
1	5	1	5	16
1	5	1	5	16
1	5	1	2	8
2	5	1	2	16
2	5	1	11	14
1	5	2	7	15
2	5	2	8	13
2	5	2	8	15
1	5	2	8	15
2	1	1	9	7
1	1	1	6	15
2	1	1	9	16
1	1	1	1	15
2	1	1	3	16
2	1	1	9	25
1	1	1	10	4
1	1	1	10	16
1	1	1	10	18
2	1	1	9	16
1	1	1	9	
2	1	1	9	16

```
2   1   2   7   16
2   1   2   7   13
1   1   2   6   15
1   1   2   1   10
1   1   2   1   12
1   1   2   10  11
2   1   2   7   25
2   1   2   7   15
1   1   2   8   14
2   1   2   7   12
1   1   2   8   6
1   2   1   5   7
2   2   1   6   8
1   2   1   7   5
2   2   2   6   10
2   2   2   6   18
1   2   2   7   10
1   2   2   8   12
;
run;


/* 2. Main Analysis: Mixed Model (Surgeon as Random Effect) */
proc mixed data=sensitive_data method=reml noclprint;
  class group surgeon;
  model sensitive = group / solution ddfm=satterthwaite;
  random surgeon;
  lsmeans group / cl pdiff;
  ods output LSMeans=lsmeans_mixed CovParms=covparms;
  title "Main Analysis: Mixed Effects Model with Surgeon as Random";
run;

/* Extract Residual Standard Deviation */
data _null_;
  set covparms;
  if CovParm="Residual" then call symputx("resid_sd_mixed",
sqrt(Estimate));
run;

/* Calculate Cohen's d */
data cohens_d_mixed;
  length label $50;
  set lsmeans_mixed(where=(group in (1,2)) rename=(Estimate=adj_mean
StdErr=adj_se));
```

```
  retain mean1 mean2;
  if group=1 then mean1=adj_mean;
  else if group=2 then mean2=adj_mean;
  if _n_=2 then do;
    s_pooled = &resid_sd_mixed;
    cohens_d = (mean1 - mean2) / s_pooled;
    label = "Main Analysis (Mixed Model)";
    output;
  end;
  keep label cohens_d mean1 mean2 s_pooled;
run;

proc print data=cohens_d_mixed noobs label;
  title "Cohen's d from Main Analysis (Mixed Effects Model)";
run;


/* 3. Sensitivity Analysis 1: Adjusting Hospital (Mixed Model) */
proc mixed data=sensitive_data method=reml noclprint;
  class group surgeon hospital;
  model sensitive = group hospital / solution ddfm=satterthwaite;
  random surgeon;
  lsmeans group / cl pdiff;
  ods output LSMeans=lsmeans_hospital CovParms=covparms_hosp;
  title "Sensitivity Analysis 1: Mixed Model Adjusting Hospital";
run;

/* Extract Residual Standard Deviation */
data _null_;
  set covparms_hosp;
  if CovParm="Residual" then call symputx("resid_sd_hosp",
sqrt(Estimate));
run;

/* Calculate Cohen's d */
data cohens_d_hosp;
  length label $50;
  set lsmeans_hospital(where=(group in (1,2))
rename=(Estimate=adj_mean StdErr=adj_se));
  retain mean1 mean2;
  if group=1 then mean1=adj_mean;
  else if group=2 then mean2=adj_mean;
  if _n_=2 then do;
    s_pooled = &resid_sd_hosp;
```

```
    cohens_d = (mean1 - mean2) / s_pooled;
    label = "Sensitivity Analysis Adjusting Hospital";
    output;
  end;
  keep label cohens_d mean1 mean2 s_pooled;
run;

proc print data=cohens_d_hosp noobs label;
  title "Cohen's d from Sensitivity Analysis Adjusting Hospital";
run;


/* 4. Sensitivity Analysis 2: Adjusting Surgery Type (Mixed Model) */
proc mixed data=sensitive_data method=reml noclprint;
  class group surgeon surgery_type;
  model sensitive = group surgery_type / solution ddfm=satterthwaite;
  random surgeon;
  lsmeans group / cl pdiff;
  ods output LSMeans=lsmeans_surgtype CovParms=covparms_surgtype;
  title "Sensitivity Analysis 2: Mixed Model Adjusting Surgery Type";
run;

/* Extract Residual Standard Deviation */
data _null_;
  set covparms_surgtype;
  if CovParm="Residual" then call symputx("resid_sd_surgtype",
sqrt(Estimate));
run;

/* Calculate Cohen's d */
data cohens_d_surgtype;
  length label $50;
  set lsmeans_surgtype(where=(group in (1,2))
rename=(Estimate=adj_mean StdErr=adj_se));
  retain mean1 mean2;
  if group=1 then mean1=adj_mean;
  else if group=2 then mean2=adj_mean;
  if _n_=2 then do;
    s_pooled = &resid_sd_surgtype;
    cohens_d = (mean1 - mean2) / s_pooled;
    label = "Sensitivity Analysis Adjusting Surgery Type";
    output;
  end;
  keep label cohens_d mean1 mean2 s_pooled;
```

```
run;

proc print data=cohens_d_surgtype noobs label;
  title "Cohen's d from Sensitivity Analysis Adjusting Surgery Type";
run;


/* 5. Sensitivity Analysis 3: Adjusting Surgeon (fixed effect) */
proc mixed data=sensitive_data method=reml noclprint;
  class group surgeon;
  model sensitive = group surgeon / solution ddfm=satterthwaite;
  lsmeans group / cl pdiff;
  ods output LSMeans=lsmeans_surg_fixed CovParms=covparms_surg_fixed;
  title "Sensitivity Analysis 3: Mixed Model Adjusting Surgeon (as
fixed effect)";
run;

/* Extract Residual Standard Deviation */
data _null_;
  set covparms_surg_fixed;
  if CovParm="Residual" then call symputx("resid_sd_surg_fixed",
sqrt(Estimate));
run;

/* Calculate Cohen's d */
data cohens_d_surg_fixed;
  length label $50;
  set lsmeans_surg_fixed(where=(group in (1,2))
rename=(Estimate=adj_mean StdErr=adj_se));
  retain mean1 mean2;
  if group=1 then mean1=adj_mean;
  else if group=2 then mean2=adj_mean;
  if _n_=2 then do;
    s_pooled = &resid_sd_surg_fixed;
    cohens_d = (mean1 - mean2) / s_pooled;
    label = "Sensitivity Analysis Adjusting Surgeon (fixed effect)";
    output;
  end;
  keep label cohens_d mean1 mean2 s_pooled;
run;

proc print data=cohens_d_surg_fixed noobs label;
  title "Cohen's d from Sensitivity Analysis Adjusting Surgeon
(fixed)";
```

```
run;
```

3.7.1.3 At 6w follow-up point

```
/* Data Input */
/* Data details:
   group: 1=telesurgery group, 2=local surgery group
   hospital: 1=Beijing, 2=Harbin, 3=Hangzhou, 4=Hefei, 5=Urumqi
   surgery_type: 1=Radical Prostatectomy, 2=Partial Nephrectomy
   surgeon codes: 1=Chaozhao Liang, 2=Baojun Wang, 3=Xu Zhang,
4=Sheng Tai,
                  5=Hongzhao Li, 6=Wanghai Xu, 7=Xin Ma, 8=Qingbo
Huang,
                  9=Weijun Fu, 10=Shuo Wang, 11=Mulati Rexiati
*/
data sensitive_data;
input group hospital surgery_type surgeon sensitive;
datalines;
2   3   1   10  14
1   3   1   5
2   3   1   10  29
2   3   1   10
1   3   1   5
2   3   1   10  20
1   3   2   7   6
2   3   2   10  20
2   3   2   10  15
1   3   2   7   30
2   3   2   10
1   3   2   7
2   3   2   10  20
1   3   2   7   28
1   4   1   2
2   4   1   4   16
2   4   1   4
1   4   1   3   15
1   4   1   5   16
2   4   1   1
2   4   2   1
2   4   2   1   18
1   4   2   7   13
1   5   1   5   13
1   5   1   5   17
1   5   1   5   16
```

```
1   5   1   2   10
2   5   1   2   16
2   5   1   11  14
1   5   2   7   15
2   5   2   8   13
2   5   2   8   15
1   5   2   8   16
2   1   1   9   8
1   1   1   6   14
2   1   1   9   14
1   1   1   1   16
2   1   1   3   16
2   1   1   9   20
1   1   1   10  5
1   1   1   10  13
1   1   1   10  20
2   1   1   9   16
1   1   1   9
2   1   1   9   20
2   1   2   7   18
2   1   2   7   16
1   1   2   6   23
1   1   2   1   12
1   1   2   1   14
1   1   2   10  14
2   1   2   7   20
2   1   2   7   20
1   1   2   8   9
2   1   2   7   12
1   1   2   8   9
1   2   1   5   9
2   2   1   6   10
1   2   1   7   7
2   2   2   6   13
2   2   2   6   10
1   2   2   7   12
1   2   2   8   12
;
run;


/* 2. Main Analysis: Mixed Model (Surgeon as Random Effect) */
proc mixed data=sensitive_data method=reml noclprint;
  class group surgeon;
```

```
  model sensitive = group / solution ddfm=satterthwaite;
  random surgeon;
  lsmeans group / cl pdiff;
  ods output LSMeans=lsmeans_mixed CovParms=covparms;
  title "Main Analysis: Mixed Effects Model with Surgeon as Random";
run;

/* Extract Residual Standard Deviation */
data _null_;
  set covparms;
  if CovParm="Residual" then call symputx("resid_sd_mixed",
sqrt(Estimate));
run;

/* Calculate Cohen's d */
data cohens_d_mixed;
  length label $50;
  set lsmeans_mixed(where=(group in (1,2)) rename=(Estimate=adj_mean
StdErr=adj_se));
  retain mean1 mean2;
  if group=1 then mean1=adj_mean;
  else if group=2 then mean2=adj_mean;
  if _n_=2 then do;
    s_pooled = &resid_sd_mixed;
    cohens_d = (mean1 - mean2) / s_pooled;
    label = "Main Analysis (Mixed Model)";
    output;
  end;
  keep label cohens_d mean1 mean2 s_pooled;
run;

proc print data=cohens_d_mixed noobs label;
  title "Cohen's d from Main Analysis (Mixed Effects Model)";
run;


/* 3. Sensitivity Analysis 1: Adjusting Hospital (Mixed Model) */
proc mixed data=sensitive_data method=reml noclprint;
  class group surgeon hospital;
  model sensitive = group hospital / solution ddfm=satterthwaite;
  random surgeon;
  lsmeans group / cl pdiff;
  ods output LSMeans=lsmeans_hospital CovParms=covparms_hosp;
  title "Sensitivity Analysis 1: Mixed Model Adjusting Hospital";
```

```
run;

/* Extract Residual Standard Deviation */
data _null_;
  set covparms_hosp;
  if CovParm="Residual" then call symputx("resid_sd_hosp",
sqrt(Estimate));
run;

/* Calculate Cohen's d */
data cohens_d_hosp;
  length label $50;
  set lsmeans_hospital(where=(group in (1,2))
rename=(Estimate=adj_mean StdErr=adj_se));
  retain mean1 mean2;
  if group=1 then mean1=adj_mean;
  else if group=2 then mean2=adj_mean;
  if _n_=2 then do;
    s_pooled = &resid_sd_hosp;
    cohens_d = (mean1 - mean2) / s_pooled;
    label = "Sensitivity Analysis Adjusting Hospital";
    output;
  end;
  keep label cohens_d mean1 mean2 s_pooled;
run;

proc print data=cohens_d_hosp noobs label;
  title "Cohen's d from Sensitivity Analysis Adjusting Hospital";
run;


/* 4. Sensitivity Analysis 2: Adjusting Surgery Type (Mixed Model) */
proc mixed data=sensitive_data method=reml noclprint;
  class group surgeon surgery_type;
  model sensitive = group surgery_type / solution ddfm=satterthwaite;
  random surgeon;
  lsmeans group / cl pdiff;
  ods output LSMeans=lsmeans_surgtype CovParms=covparms_surgtype;
  title "Sensitivity Analysis 2: Mixed Model Adjusting Surgery Type";
run;

/* Extract Residual Standard Deviation */
data _null_;
  set covparms_surgtype;
```

```
  if CovParm="Residual" then call symputx("resid_sd_surgtype",
sqrt(Estimate));
run;

/* Calculate Cohen's d */
data cohens_d_surgtype;
  length label $50;
  set lsmeans_surgtype(where=(group in (1,2))
rename=(Estimate=adj_mean StdErr=adj_se));
  retain mean1 mean2;
  if group=1 then mean1=adj_mean;
  else if group=2 then mean2=adj_mean;
  if _n_=2 then do;
    s_pooled = &resid_sd_surgtype;
    cohens_d = (mean1 - mean2) / s_pooled;
    label = "Sensitivity Analysis Adjusting Surgery Type";
    output;
  end;
  keep label cohens_d mean1 mean2 s_pooled;
run;

proc print data=cohens_d_surgtype noobs label;
  title "Cohen's d from Sensitivity Analysis Adjusting Surgery Type";
run;


/* 5. Sensitivity Analysis 3: Adjusting Surgeon (fixed effect) */
proc mixed data=sensitive_data method=reml noclprint;
  class group surgeon;
  model sensitive = group surgeon / solution ddfm=satterthwaite;
  lsmeans group / cl pdiff;
  ods output LSMeans=lsmeans_surg_fixed CovParms=covparms_surg_fixed;
  title "Sensitivity Analysis 3: Mixed Model Adjusting Surgeon (as
fixed effect)";
run;

/* Extract Residual Standard Deviation */
data _null_;
  set covparms_surg_fixed;
  if CovParm="Residual" then call symputx("resid_sd_surg_fixed",
sqrt(Estimate));
run;

/* Calculate Cohen's d */
```

```
data cohens_d_surg_fixed;
  length label $50;
  set lsmeans_surg_fixed(where=(group in (1,2))
rename=(Estimate=adj_mean StdErr=adj_se));
  retain mean1 mean2;
  if group=1 then mean1=adj_mean;
  else if group=2 then mean2=adj_mean;
  if _n_=2 then do;
    s_pooled = &resid_sd_surg_fixed;
    cohens_d = (mean1 - mean2) / s_pooled;
    label = "Sensitivity Analysis Adjusting Surgeon (fixed effect)";
    output;
  end;
  keep label cohens_d mean1 mean2 s_pooled;
run;

proc print data=cohens_d_surg_fixed noobs label;
  title "Cohen's d from Sensitivity Analysis Adjusting Surgeon
(fixed)";
run;
```

3.7.2 For Renal tumor patients

3.7.2.1 Baseline

3.7.2.2 At 4w follow-up point

3.7.2.3 At 6w follow-up point

3.7.3 For Prostate cancer patients

3.7.3.1 Baseline

3.7.3.2 At 4w follow-up point

3.7.3.3 At 6w follow-up point

3.8 EPIC-26 Score

3.8.1 Total

3.8.1.1 Baseline

```
/* Data Input */
/* Data details:
   group: 1=telesurgery group, 2=local surgery group
   hospital: 1=Beijing, 2=Harbin, 3=Hangzhou, 4=Hefei, 5=Urumqi
   surgeon codes: 1=Chaozhao Liang, 2=Baojun Wang, 3=Xu Zhang,
4=Sheng Tai,
                  5=Hongzhao Li, 6=Wanghai Xu, 7=Xin Ma, 8=Qingbo
Huang,
                  9=Weijun Fu, 10=Shuo Wang, 11=Mulati Rexiati
*/
data sensitive_data;
input group hospital surgeon sensitive;
datalines;
2   3   10  53
1   3   5   42
2   3   10  60
2   3   10  50
1   3   5   97
2   3   10  48
1   4   2   31
2   4   4   58
2   4   4   53
1   4   3   82
1   4   5   44
2   4   1   48
1   5   5   40
1   5   5   80
1   5   5   37
1   5   2   52
2   5   2   29
2   5   11  64
2   1   9   71
1   1   6   96
2   1   9   60
1   1   1   55
2   1   3   27
2   1   9   46
1   1   10  58
```

```
1   1   10  57
1   1   10  44
2   1   9   38
1   1   9   55
2   1   9   52
1   2   5   26
2   2   6   26
1   2   7   27
;
run;


/* 2. Main Analysis: Mixed Model (Surgeon as Random Effect) */
proc mixed data=sensitive_data method=reml noclprint;
  class group surgeon;
  model sensitive = group / solution ddfm=satterthwaite;
  random surgeon;
  lsmeans group / cl pdiff;
  ods output LSMeans=lsmeans_mixed CovParms=covparms;
  title "Main Analysis: Mixed Effects Model with Surgeon as Random";
run;

/* Extract Residual Standard Deviation */
data _null_;
  set covparms;
  if CovParm="Residual" then call symputx("resid_sd_mixed",
sqrt(Estimate));
run;

/* Calculate Cohen's d */
data cohens_d_mixed;
  length label $50;
  set lsmeans_mixed(where=(group in (1,2)) rename=(Estimate=adj_mean
StdErr=adj_se));
  retain mean1 mean2;
  if group=1 then mean1=adj_mean;
  else if group=2 then mean2=adj_mean;
  if _n_=2 then do;
    s_pooled = &resid_sd_mixed;
    cohens_d = (mean1 - mean2) / s_pooled;
    label = "Main Analysis (Mixed Model)";
    output;
  end;
  keep label cohens_d mean1 mean2 s_pooled;
```

```
run;

proc print data=cohens_d_mixed noobs label;
  title "Cohen's d from Main Analysis (Mixed Effects Model)";
run;


/* 3. Sensitivity Analysis 1: Adjusting Hospital (Mixed Model) */
proc mixed data=sensitive_data method=reml noclprint;
  class group surgeon hospital;
  model sensitive = group hospital / solution ddfm=satterthwaite;
  random surgeon;
  lsmeans group / cl pdiff;
  ods output LSMeans=lsmeans_hospital CovParms=covparms_hosp;
  title "Sensitivity Analysis 1: Mixed Model Adjusting Hospital";
run;

/* Extract Residual Standard Deviation */
data _null_;
  set covparms_hosp;
  if CovParm="Residual" then call symputx("resid_sd_hosp",
sqrt(Estimate));
run;

/* Calculate Cohen's d */
data cohens_d_hosp;
  length label $50;
  set lsmeans_hospital(where=(group in (1,2))
rename=(Estimate=adj_mean StdErr=adj_se));
  retain mean1 mean2;
  if group=1 then mean1=adj_mean;
  else if group=2 then mean2=adj_mean;
  if _n_=2 then do;
    s_pooled = &resid_sd_hosp;
    cohens_d = (mean1 - mean2) / s_pooled;
    label = "Sensitivity Analysis Adjusting Hospital";
    output;
  end;
  keep label cohens_d mean1 mean2 s_pooled;
run;

proc print data=cohens_d_hosp noobs label;
  title "Cohen's d from Sensitivity Analysis Adjusting Hospital";
run;
```

```
/* 4. Sensitivity Analysis 3: Adjusting Surgeon (fixed effect) */
proc mixed data=sensitive_data method=reml noclprint;
  class group surgeon;
  model sensitive = group surgeon / solution ddfm=satterthwaite;
  lsmeans group / cl pdiff;
  ods output LSMeans=lsmeans_surg_fixed CovParms=covparms_surg_fixed;
  title "Sensitivity Analysis 3: Mixed Model Adjusting Surgeon (as
fixed effect)";
run;

/* Extract Residual Standard Deviation */
data _null_;
  set covparms_surg_fixed;
  if CovParm="Residual" then call symputx("resid_sd_surg_fixed",
sqrt(Estimate));
run;

/* Calculate Cohen's d */
data cohens_d_surg_fixed;
  length label $50;
  set lsmeans_surg_fixed(where=(group in (1,2))
rename=(Estimate=adj_mean StdErr=adj_se));
  retain mean1 mean2;
  if group=1 then mean1=adj_mean;
  else if group=2 then mean2=adj_mean;
  if _n_=2 then do;
    s_pooled = &resid_sd_surg_fixed;
    cohens_d = (mean1 - mean2) / s_pooled;
    label = "Sensitivity Analysis Adjusting Surgeon (fixed effect)";
    output;
  end;
  keep label cohens_d mean1 mean2 s_pooled;
run;

proc print data=cohens_d_surg_fixed noobs label;
  title "Cohen's d from Sensitivity Analysis Adjusting Surgeon
(fixed)";
run;
```

3.8.1.2 At 4w follow-up point

```
/* Data Input */
```

```
/* Data details:
   group: 1=telesurgery group, 2=local surgery group
   hospital: 1=Beijing, 2=Harbin, 3=Hangzhou, 4=Hefei, 5=Urumqi
   surgeon codes: 1=Chaozhao Liang, 2=Baojun Wang, 3=Xu Zhang,
4=Sheng Tai,
                  5=Hongzhao Li, 6=Wanghai Xu, 7=Xin Ma, 8=Qingbo
Huang,
                  9=Weijun Fu, 10=Shuo Wang, 11=Mulati Rexiati
*/
data sensitive_data;
input group hospital surgeon sensitive;
datalines;
2   3   10  46
1   3   5   68
2   3   10  73
2   3   10  68
1   3   5   46
2   3   10  55
1   4   2   65
2   4   4   61
2   4   4   57
1   4   3   63
1   4   5   58
2   4   1   57
1   5   5   56
1   5   5   75
1   5   5   46
1   5   2   50
2   5   2   36
2   5   11  50
2   1   9   58
1   1   6   86
2   1   9   79
1   1   1   69
2   1   3   77
2   1   9   68
1   1   10  77
1   1   10  56
1   1   10  84
2   1   9   68
1   1   9   55
2   1   9   67
1   2   5   70
2   2   6   55
```

```
1   2   7   64
;
run;


/* 2. Main Analysis: Mixed Model (Surgeon as Random Effect) */
proc mixed data=sensitive_data method=reml noclprint;
  class group surgeon;
  model sensitive = group / solution ddfm=satterthwaite;
  random surgeon;
  lsmeans group / cl pdiff;
  ods output LSMeans=lsmeans_mixed CovParms=covparms;
  title "Main Analysis: Mixed Effects Model with Surgeon as Random";
run;

/* Extract Residual Standard Deviation */
data _null_;
  set covparms;
  if CovParm="Residual" then call symputx("resid_sd_mixed",
sqrt(Estimate));
run;

/* Calculate Cohen's d */
data cohens_d_mixed;
  length label $50;
  set lsmeans_mixed(where=(group in (1,2)) rename=(Estimate=adj_mean
StdErr=adj_se));
  retain mean1 mean2;
  if group=1 then mean1=adj_mean;
  else if group=2 then mean2=adj_mean;
  if _n_=2 then do;
    s_pooled = &resid_sd_mixed;
    cohens_d = (mean1 - mean2) / s_pooled;
    label = "Main Analysis (Mixed Model)";
    output;
  end;
  keep label cohens_d mean1 mean2 s_pooled;
run;

proc print data=cohens_d_mixed noobs label;
  title "Cohen's d from Main Analysis (Mixed Effects Model)";
run;
```

```
/* 3. Sensitivity Analysis 1: Adjusting Hospital (Mixed Model) */
proc mixed data=sensitive_data method=reml noclprint;
  class group surgeon hospital;
  model sensitive = group hospital / solution ddfm=satterthwaite;
  random surgeon;
  lsmeans group / cl pdiff;
  ods output LSMeans=lsmeans_hospital CovParms=covparms_hosp;
  title "Sensitivity Analysis 1: Mixed Model Adjusting Hospital";
run;

/* Extract Residual Standard Deviation */
data _null_;
  set covparms_hosp;
  if CovParm="Residual" then call symputx("resid_sd_hosp",
sqrt(Estimate));
run;

/* Calculate Cohen's d */
data cohens_d_hosp;
  length label $50;
  set lsmeans_hospital(where=(group in (1,2))
rename=(Estimate=adj_mean StdErr=adj_se));
  retain mean1 mean2;
  if group=1 then mean1=adj_mean;
  else if group=2 then mean2=adj_mean;
  if _n_=2 then do;
    s_pooled = &resid_sd_hosp;
    cohens_d = (mean1 - mean2) / s_pooled;
    label = "Sensitivity Analysis Adjusting Hospital";
    output;
  end;
  keep label cohens_d mean1 mean2 s_pooled;
run;

proc print data=cohens_d_hosp noobs label;
  title "Cohen's d from Sensitivity Analysis Adjusting Hospital";
run;


/* 4. Sensitivity Analysis 3: Adjusting Surgeon (fixed effect) */
proc mixed data=sensitive_data method=reml noclprint;
  class group surgeon;
  model sensitive = group surgeon / solution ddfm=satterthwaite;
  lsmeans group / cl pdiff;
```

```
  ods output LSMeans=lsmeans_surg_fixed CovParms=covparms_surg_fixed;
  title "Sensitivity Analysis 3: Mixed Model Adjusting Surgeon (as
fixed effect)";
run;

/* Extract Residual Standard Deviation */
data _null_;
  set covparms_surg_fixed;
  if CovParm="Residual" then call symputx("resid_sd_surg_fixed",
sqrt(Estimate));
run;

/* Calculate Cohen's d */
data cohens_d_surg_fixed;
  length label $50;
  set lsmeans_surg_fixed(where=(group in (1,2))
rename=(Estimate=adj_mean StdErr=adj_se));
  retain mean1 mean2;
  if group=1 then mean1=adj_mean;
  else if group=2 then mean2=adj_mean;
  if _n_=2 then do;
    s_pooled = &resid_sd_surg_fixed;
    cohens_d = (mean1 - mean2) / s_pooled;
    label = "Sensitivity Analysis Adjusting Surgeon (fixed effect)";
    output;
  end;
  keep label cohens_d mean1 mean2 s_pooled;
run;

proc print data=cohens_d_surg_fixed noobs label;
  title "Cohen's d from Sensitivity Analysis Adjusting Surgeon
(fixed)";
run;
```

3.8.1.3 At 6w follow-up point

```
/* Data Input */
/* Data details:
   group: 1=telesurgery group, 2=local surgery group
   hospital: 1=Beijing, 2=Harbin, 3=Hangzhou, 4=Hefei, 5=Urumqi
   surgeon codes: 1=Chaozhao Liang, 2=Baojun Wang, 3=Xu Zhang,
4=Sheng Tai,
                  5=Hongzhao Li, 6=Wanghai Xu, 7=Xin Ma, 8=Qingbo
Huang,
```

```
                   9=Weijun Fu, 10=Shuo Wang, 11=Mulati Rexiati
*/
data sensitive_data;
input group hospital surgeon sensitive;
datalines;
2   3   10  46
1   3   5   72
2   3   10  73
2   3   10  68
1   3   5   46
2   3   10  55
1   4   2   64
2   4   4   54
2   4   4   53
1   4   3   63
1   4   5   56
2   4   1
1   5   5   46
1   5   5   79
1   5   5   34
1   5   2   47
2   5   2   34
2   5   11  46
2   1   9   64
1   1   6   89
2   1   9   73
1   1   1   70
2   1   3   51
2   1   9   62
1   1   10  66
1   1   10  54
1   1   10  66
2   1   9   64
1   1   9   56
2   1   9   62
1   2   5   47
2   2   6   56
1   2   7   56
;
run;


/* 2. Main Analysis: Mixed Model (Surgeon as Random Effect) */
proc mixed data=sensitive_data method=reml noclprint;
```

```
  class group surgeon;
  model sensitive = group / solution ddfm=satterthwaite;
  random surgeon;
  lsmeans group / cl pdiff;
  ods output LSMeans=lsmeans_mixed CovParms=covparms;
  title "Main Analysis: Mixed Effects Model with Surgeon as Random";
run;

/* Extract Residual Standard Deviation */
data _null_;
  set covparms;
  if CovParm="Residual" then call symputx("resid_sd_mixed",
sqrt(Estimate));
run;

/* Calculate Cohen's d */
data cohens_d_mixed;
  length label $50;
  set lsmeans_mixed(where=(group in (1,2)) rename=(Estimate=adj_mean
StdErr=adj_se));
  retain mean1 mean2;
  if group=1 then mean1=adj_mean;
  else if group=2 then mean2=adj_mean;
  if _n_=2 then do;
    s_pooled = &resid_sd_mixed;
    cohens_d = (mean1 - mean2) / s_pooled;
    label = "Main Analysis (Mixed Model)";
    output;
  end;
  keep label cohens_d mean1 mean2 s_pooled;
run;

proc print data=cohens_d_mixed noobs label;
  title "Cohen's d from Main Analysis (Mixed Effects Model)";
run;


/* 3. Sensitivity Analysis 1: Adjusting Hospital (Mixed Model) */
proc mixed data=sensitive_data method=reml noclprint;
  class group surgeon hospital;
  model sensitive = group hospital / solution ddfm=satterthwaite;
  random surgeon;
  lsmeans group / cl pdiff;
  ods output LSMeans=lsmeans_hospital CovParms=covparms_hosp;
```

```
  title "Sensitivity Analysis 1: Mixed Model Adjusting Hospital";
run;

/* Extract Residual Standard Deviation */
data _null_;
  set covparms_hosp;
  if CovParm="Residual" then call symputx("resid_sd_hosp",
sqrt(Estimate));
run;

/* Calculate Cohen's d */
data cohens_d_hosp;
  length label $50;
  set lsmeans_hospital(where=(group in (1,2))
rename=(Estimate=adj_mean StdErr=adj_se));
  retain mean1 mean2;
  if group=1 then mean1=adj_mean;
  else if group=2 then mean2=adj_mean;
  if _n_=2 then do;
    s_pooled = &resid_sd_hosp;
    cohens_d = (mean1 - mean2) / s_pooled;
    label = "Sensitivity Analysis Adjusting Hospital";
    output;
  end;
  keep label cohens_d mean1 mean2 s_pooled;
run;

proc print data=cohens_d_hosp noobs label;
  title "Cohen's d from Sensitivity Analysis Adjusting Hospital";
run;


/* 4. Sensitivity Analysis 3: Adjusting Surgeon (fixed effect) */
proc mixed data=sensitive_data method=reml noclprint;
  class group surgeon;
  model sensitive = group surgeon / solution ddfm=satterthwaite;
  lsmeans group / cl pdiff;
  ods output LSMeans=lsmeans_surg_fixed CovParms=covparms_surg_fixed;
  title "Sensitivity Analysis 3: Mixed Model Adjusting Surgeon (as
fixed effect)";
run;

/* Extract Residual Standard Deviation */
data _null_;
```

```
  set covparms_surg_fixed;
  if CovParm="Residual" then call symputx("resid_sd_surg_fixed",
sqrt(Estimate));
run;

/* Calculate Cohen's d */
data cohens_d_surg_fixed;
  length label $50;
  set lsmeans_surg_fixed(where=(group in (1,2))
rename=(Estimate=adj_mean StdErr=adj_se));
  retain mean1 mean2;
  if group=1 then mean1=adj_mean;
  else if group=2 then mean2=adj_mean;
  if _n_=2 then do;
    s_pooled = &resid_sd_surg_fixed;
    cohens_d = (mean1 - mean2) / s_pooled;
    label = "Sensitivity Analysis Adjusting Surgeon (fixed effect)";
    output;
  end;
  keep label cohens_d mean1 mean2 s_pooled;
run;

proc print data=cohens_d_surg_fixed noobs label;
  title "Cohen's d from Sensitivity Analysis Adjusting Surgeon
(fixed)";

run;
```

3.8.2 For Sexual function

3.8.2.1 Baseline

```
/* Data Input */
data epicss_data;
input group hospital epics; /* group 1=telesurgery group,  group
2=local surgery group; hospital 1=Beijing, hospital 2= Harbin,
hospital 3=Hangzhou, hospital 4=Hefei, hospital 5=Urumqi*/
datalines;
1   4   11
1   4   21
2   4   20
1   4   19
2   4   20
2   4   20
1   2   6
```

```
2   2   6
1   2   6
1   1   29
1   1   27
2   1   25
2   1   17
2   1   28
1   1   18
1   1   26
1   1   25
1   1   27
2   1   17
2   3   18
1   3   19
2   3   21
2   3   25
1   3   13
2   3   25
2   1   20
2   5   9
2   5   26
1   5   14
1   5   26
1   5   25
1   5   17
2   1   6
;
run;

/* Main Analysis: Linear Regression Model (with Hospital and Surgery
Type as Fixed Effects) */
proc glm data= epicss_data;
class group hospital;
model epics = group hospital / solution;
lsmeans group / pdiff=control('2') cl;  /* Using the Local Surgery
Group (2) as the Reference */
ods output LSMeanDiffCL=effect_est;     /* Output Effect Estimates */
title "Main Analysis: ANCOVA with Fixed Effects";
run;

/* Sensitivity Analysis 1: Mixed Effects Model (with Hospital as a
Random Effect) */
proc mixed data= epicss_data;
class group hospital;
```

```
model epics = group/ solution ddfm=satterthwaite;
random hospital;
lsmeans group / pdiff cl;
ods output Estimates=mixed_effects;
title "Sensitivity Analysis: Mixed Effects Model";
run;


/* Sensitivity Analysis 2: Only Adjusting for Hospital*/
proc glm data= epicss_data;
class group hospital;
model epics = group hospital / solution;
lsmeans group / pdiff cl;
title "Sensitivity Analysis: Adjusting Only hospital";
run;

/* Calculate Effect Size (Cohen's d) */
/* Main Analysis: Linear Regression Model Adjusted for Hospital and
Surgery Type */
proc glm data=epicss_data;
  class group hospital;
  model Epics = group hospital/ solution;
  lsmeans group / pdiff cl;
  ods output ParameterEstimates=params FitStatistics=fit;
  title 'Main Analysis: Model Adjusted for Hospital and Surgery Type';
run;

/* Calculate Descriptive Statistics for Each Group (Mean, Standard
Deviation, Sample Size) */
proc means data=epicss_data noprint;
  class group;
  var Epics;
  output out=group_stats mean=mean std=std n=n;
run;

/* Only Retain Group-wise Statistical Results (Exclude Overall
Statistics Row) */
data group_stats;
  set group_stats;
  where _TYPE_ = 1;  /* _TYPE_=1 Indicates Statistical Results
Grouped by group */
run;

/* Combine the Statistics of the Two Groups and Calculate Cohen's d
```

```
*/
data cohens_d;
/* Initialize Variables */
  retain group1_mean group1_std group1_n group2_mean group2_std 
group2_n;

/* Read Statistics for the First Group (group=1) */
  set group_stats(where=(group=1) 
    rename=(mean=group1_mean std=group1_std n=group1_n));

/* Read Statistics for the Second Group (group=2) */
  set group_stats(where=(group=2) 
    rename=(mean=group2_mean std=group2_std n=group2_n));

/* Calculate Pooled Standard Deviation */
  s_pooled = sqrt(
    ((group1_n - 1) * group1_std**2 + (group2_n - 1) * group2_std**2)
    / (group1_n + group2_n - 2)
  );

  /* Calculate Cohen's d */
  cohens_d = (group1_mean - group2_mean) / s_pooled;

/* Only Retain Key Results */
  keep cohens_d s_pooled group1_mean group2_mean;
run;

/* Print d value */
proc print data=cohens_d;
  title "Cohen's d Effect Size Calculation Result (Without Covariate 
Adjustment)";
  format cohens_d 8.2;
run;
```

3.8.2.2 At 4w follow-up point

```
/* Data Input */
data epicss_data;
input group hospital epics; /* group 1=telesurgery group,  group 
2=local surgery group; hospital 1=Beijing, hospital 2= Harbin, 
hospital 3=Hangzhou, hospital 4=Hefei, hospital 5=Urumqi */
datalines;
1   4   29
1   4   26
```

```
2   4   19
1   4   25
2   4   26
2   4   26
1   2   22
2   2   13
1   2   16
1   1   27
1   1   29
2   1   29
2   1   27
2   1   29
1   1   29
1   1   25
1   1   25
1   1   26
2   1   27
2   3   26
1   3   28
2   3   28
2   3   28
1   3   26
2   3   26
2   1   28
2   5   16
2   5   26
1   5   26
1   5   22
1   5   25
1   5   23
2   1   25
;
run;

/* Main Analysis: Linear Regression Model (with Hospital and Surgery
Type as Fixed Effects) */
proc glm data= epicss_data;
class group hospital;
model epics = group hospital / solution;
lsmeans group / pdiff=control('2') cl;  /* Using the Local Surgery
Group (2) as the Reference */
ods output LSMeanDiffCL=effect_est;     /* Output Effect Estimates */
title "Main Analysis: ANCOVA with Fixed Effects";
run;
```

```
/* Sensitivity Analysis 1: Mixed Effects Model (with Hospital as a
Random Effect) */
proc mixed data= epicss_data;
class group hospital;
model epics = group/ solution ddfm=satterthwaite;
random hospital;
lsmeans group / pdiff cl;
ods output Estimates=mixed_effects;
title "Sensitivity Analysis: Mixed Effects Model";
run;


/* Sensitivity Analysis 2: Only Adjusting for Hospital*/
proc glm data= epicss_data;
class group hospital;
model epics = group hospital / solution;
lsmeans group / pdiff cl;
title "Sensitivity Analysis: Adjusting Only hospital";
run;

/* Calculate Effect Size (Cohen's d) */
/* Main Analysis: Linear Regression Model Adjusted for Hospital and
Surgery Type */
proc glm data=epicss_data;
  class group hospital;
  model Epics = group hospital/ solution;
  lsmeans group / pdiff cl;
  ods output ParameterEstimates=params FitStatistics=fit;
  title 'Main Analysis: Model Adjusted for Hospital and Surgery Type';
run;

/* Calculate Descriptive Statistics for Each Group (Mean, Standard
Deviation, Sample Size) */
proc means data=epicss_data noprint;
  class group;
  var Epics;
  output out=group_stats mean=mean std=std n=n;
run;

/* Only Retain Group-wise Statistical Results (Exclude Overall
Statistics Row) */
data group_stats;
  set group_stats;
```

```
  where _TYPE_ = 1;  /* _TYPE_=1 Indicates Statistical Results
Grouped by group */
run;

/* Combine the Statistics of the Two Groups and Calculate Cohen's d
*/
data cohens_d;
/* Initialize Variables */
  retain group1_mean group1_std group1_n group2_mean group2_std
group2_n;

/* Read Statistics for the First Group (group=1) */
  set group_stats(where=(group=1)
    rename=(mean=group1_mean std=group1_std n=group1_n));

/* Read Statistics for the Second Group (group=2) */
  set group_stats(where=(group=2)
    rename=(mean=group2_mean std=group2_std n=group2_n));

/* Calculate Pooled Standard Deviation */
  s_pooled = sqrt(
    ((group1_n - 1) * group1_std**2 + (group2_n - 1) * group2_std**2)
    / (group1_n + group2_n - 2)
  );

  /* Calculate Cohen's d */
  cohens_d = (group1_mean - group2_mean) / s_pooled;

/* Only Retain Key Results */
  keep cohens_d s_pooled group1_mean group2_mean;
run;

/* Print d value */
proc print data=cohens_d;
  title "Cohen's d Effect Size Calculation Result (Without Covariate
Adjustment)";
  format cohens_d 8.2;
run;
```

3.8.2.3 At 6w follow-up point

```
/* Data Input */
data epicss_data;
```

```
input group hospital epics; /* group 1=telesurgery group,  group
2=local surgery group; hospital 1=Beijing, hospital 2= Harbin,
hospital 3=Hangzhou, hospital 4=Hefei, hospital 5=Urumqi */
datalines;
1   4   29
1   4   25
2   4   26
1   4   26
2   4
2   4   22
1   2   16
2   2   19
1   2   23
1   1   27
1   1   29
2   1   25
2   1   24
2   1   29
1   1   26
1   1   27
1   1   27
1   1   27
2   1   28
2   3   26
1   3   28
2   3   28
2   3   28
1   3   26
2   3   26
2   1   24
2   5   14
2   5   26
1   5   14
1   5   26
1   5   25
1   5   19
2   1   26
;
run;

/* Main Analysis: Linear Regression Model (with Hospital and Surgery
Type as Fixed Effects) */
proc glm data= epicss_data;
class group hospital;
```

```
model epics = group hospital / solution;
lsmeans group / pdiff=control('2') cl;  /* Using the Local Surgery
Group (2) as the Reference */
ods output LSMeanDiffCL=effect_est;     /* Output Effect Estimates */
title "Main Analysis: ANCOVA with Fixed Effects";
run;

/* Sensitivity Analysis 1: Mixed Effects Model (with Hospital as a
Random Effect) */
proc mixed data= epicss_data;
class group hospital;
model epics = group/ solution ddfm=satterthwaite;
random hospital;
lsmeans group / pdiff cl;
ods output Estimates=mixed_effects;
title "Sensitivity Analysis: Mixed Effects Model";
run;


/* Sensitivity Analysis 2: Only Adjusting for Hospital*/
proc glm data= epicss_data;
class group hospital;
model epics = group hospital / solution;
lsmeans group / pdiff cl;
title "Sensitivity Analysis: Adjusting Only hospital";
run;

/* Calculate Effect Size (Cohen's d) */
/* Main Analysis: Linear Regression Model Adjusted for Hospital and
Surgery Type */
proc glm data=epicss_data;
  class group hospital;
  model Epics = group hospital/ solution;
  lsmeans group / pdiff cl;
  ods output ParameterEstimates=params FitStatistics=fit;
  title 'Main Analysis: Model Adjusted for Hospital and Surgery Type';
run;

/* Calculate Descriptive Statistics for Each Group (Mean, Standard
Deviation, Sample Size) */
proc means data=epicss_data noprint;
  class group;
  var Epics;
  output out=group_stats mean=mean std=std n=n;
```

```
run;

/* Only Retain Group-wise Statistical Results (Exclude Overall
Statistics Row) */
data group_stats;
  set group_stats;
  where _TYPE_ = 1;  /* _TYPE_=1 Indicates Statistical Results
Grouped by group */
run;

/* Combine the Statistics of the Two Groups and Calculate Cohen's d
*/
data cohens_d;
/* Initialize Variables */
  retain group1_mean group1_std group1_n group2_mean group2_std
group2_n;

/* Read Statistics for the First Group (group=1) */
  set group_stats(where=(group=1)
    rename=(mean=group1_mean std=group1_std n=group1_n));

/* Read Statistics for the Second Group (group=2) */
  set group_stats(where=(group=2)
    rename=(mean=group2_mean std=group2_std n=group2_n));

/* Calculate Pooled Standard Deviation */
  s_pooled = sqrt(
    ((group1_n - 1) * group1_std**2 + (group2_n - 1) * group2_std**2)
    / (group1_n + group2_n - 2)
  );

  /* Calculate Cohen's d */
  cohens_d = (group1_mean - group2_mean) / s_pooled;

/* Only Retain Key Results */
  keep cohens_d s_pooled group1_mean group2_mean;
run;

/* Print d value */
proc print data=cohens_d;
  title "Cohen's d Effect Size Calculation Result (Without Covariate
Adjustment)";
  format cohens_d 8.2;
run;
```

3.8.3 For Urine control

3.8.3.1 Baseline

```
/* Data Input */
data epicus_data;
input group hospital epicu; /* group 1=telesurgery group,  group
2=local surgery group; hospital 1=Beijing, hospital 2= Harbin,
hospital 3=Hangzhou, hospital 4=Hefei, hospital 5=Urumqiy*/
datalines;
1   4   4
1   4   7
2   4   8
1   4   3
2   4   4
2   4   4
1   2   4
2   2   4
1   2   4
1   1   12
1   1   4
2   1   5
2   1   4
2   1   10
1   1   4
1   1   6
1   1   4
1   1   5
2   1   4
2   3   6
1   3   4
2   3   4
2   3   4
1   3   8
2   3   5
2   1   5
2   5   4
2   5   8
1   5   4
1   5   7
```

```
1   5   4
1   5   4
2   1   4
;
run;

/* Main Analysis: Linear Regression Model (with Hospital and Surgery
Type as Fixed Effects) */
proc glm data= epicus_data;
class group hospital;
model epicu = group hospital / solution;
lsmeans group / pdiff=control('2') cl;  /* Using the Local Surgery
Group (2) as the Reference */
ods output LSMeanDiffCL=effect_est;     /* Output Effect Estimates */
title "Main Analysis: ANCOVA with Fixed Effects";
run;

/* Sensitivity Analysis 1: Mixed Effects Model (with Hospital as a
Random Effect) */
proc mixed data= epicus_data;
class group hospital;
model epicu = group/ solution ddfm=satterthwaite;
random hospital;
lsmeans group / pdiff cl;
ods output Estimates=mixed_effects;
title "Sensitivity Analysis: Mixed Effects Model";
run;


/* Sensitivity Analysis 2: Only Adjusting for Hospital*/
proc glm data= epicus_data;
class group hospital;
model epicu = group hospital / solution;
lsmeans group / pdiff cl;
title "Sensitivity Analysis: Adjusting Only hospital";
run;

/* Calculate Effect Size (Cohen's d) */
/* Main Analysis: Linear Regression Model Adjusted for Hospital and
Surgery Type */
proc glm data=epicus_data;
  class group hospital;
  model Epicu = group hospital/ solution;
  lsmeans group / pdiff cl;
```

```
  ods output ParameterEstimates=params FitStatistics=fit;
  title 'Main Analysis: Model Adjusted for Hospital and Surgery Type';
run;

/* Calculate Descriptive Statistics for Each Group (Mean, Standard
Deviation, Sample Size) */
proc means data=epicus_data noprint;
  class group;
  var Epicu;
  output out=group_stats mean=mean std=std n=n;
run;

/* Only Retain Group-wise Statistical Results (Exclude Overall
Statistics Row) */
data group_stats;
  set group_stats;
  where _TYPE_ = 1;  /* _TYPE_=1 Indicates Statistical Results
Grouped by group */
run;

/* Combine the Statistics of the Two Groups and Calculate Cohen's d
*/
data cohens_d;
/* Initialize Variables */
  retain group1_mean group1_std group1_n group2_mean group2_std
group2_n;

/* Read Statistics for the First Group (group=1) */
  set group_stats(where=(group=1)
    rename=(mean=group1_mean std=group1_std n=group1_n));

/* Read Statistics for the Second Group (group=2) */
  set group_stats(where=(group=2)
    rename=(mean=group2_mean std=group2_std n=group2_n));

/* Calculate Pooled Standard Deviation */
  s_pooled = sqrt(
    ((group1_n - 1) * group1_std**2 + (group2_n - 1) * group2_std**2)
    / (group1_n + group2_n - 2)
  );

  /* Calculate Cohen's d */
  cohens_d = (group1_mean - group2_mean) / s_pooled;
```

```
/* Only Retain Key Results */
  keep cohens_d s_pooled group1_mean group2_mean;
run;

/* Print d value */
proc print data=cohens_d;
  title "Cohen's d Effect Size Calculation Result (Without Covariate
Adjustment)";
  format cohens_d 8.2;
run;
```

3.8.3.2 At 4w follow-up point

```
/* Data Input */
data epicus_data;
input group hospital epicu; /* group 1=telesurgery group,  group
2=local surgery group; hospital 1=Beijing, hospital 2= Harbin,
hospital 3=Hangzhou, hospital 4=Hefei, hospital 5=Urumqi */
datalines;
1   4   14
1   4   14
2   4   15
1   4   15
2   4   13
2   4   13
1   2   16
2   2   14
1   2   16
1   1   17
1   1   16
2   1   16
2   1   17
2   1   9
1   1   18
1   1   15
1   1   9
1   1   10
2   1   17
2   3   4
1   3   11
2   3   18
2   3   12
1   3   4
2   3   10
```

```
2   1   11
2   5   4
2   5   4
1   5   4
1   5   6
1   5   7
1   5   10
2   1   9

;
run;

/* Main Analysis: Linear Regression Model (with Hospital and Surgery
Type as Fixed Effects) */
proc glm data= epicus_data;
class group hospital;
model epicu = group hospital / solution;
lsmeans group / pdiff=control('2') cl;  /* Using the Local Surgery
Group (2) as the Reference */
ods output LSMeanDiffCL=effect_est;     /* Output Effect Estimates */
title "Main Analysis: ANCOVA with Fixed Effects";
run;

/* Sensitivity Analysis 1: Mixed Effects Model (with Hospital as a
Random Effect) */
proc mixed data= epicus_data;
class group hospital;
model epicu = group/ solution ddfm=satterthwaite;
random hospital;
lsmeans group / pdiff cl;
ods output Estimates=mixed_effects;
title "Sensitivity Analysis: Mixed Effects Model";
run;


/* Sensitivity Analysis 2: Only Adjusting for Hospital*/
proc glm data= epicus_data;
class group hospital;
model epicu = group hospital / solution;
lsmeans group / pdiff cl;
title "Sensitivity Analysis: Adjusting Only hospital";
run;

/* Calculate Effect Size (Cohen's d) */
```

```
/* Main Analysis: Linear Regression Model Adjusted for Hospital and
Surgery Type */
proc glm data=epicus_data;
  class group hospital;
  model Epicu = group hospital/ solution;
  lsmeans group / pdiff cl;
  ods output ParameterEstimates=params FitStatistics=fit;
  title 'Main Analysis: Model Adjusted for Hospital and Surgery Type';
run;

/* Calculate Descriptive Statistics for Each Group (Mean, Standard
Deviation, Sample Size) */
proc means data=epicus_data noprint;
  class group;
  var Epicu;
  output out=group_stats mean=mean std=std n=n;
run;

/* Only Retain Group-wise Statistical Results (Exclude Overall
Statistics Row) */
data group_stats;
  set group_stats;
  where _TYPE_ = 1;  /* _TYPE_=1 Indicates Statistical Results
Grouped by group */
run;

/* Combine the Statistics of the Two Groups and Calculate Cohen's d
*/
data cohens_d;
/* Initialize Variables */
  retain group1_mean group1_std group1_n group2_mean group2_std
group2_n;

/* Read Statistics for the First Group (group=1) */
  set group_stats(where=(group=1)
    rename=(mean=group1_mean std=group1_std n=group1_n));

/* Read Statistics for the Second Group (group=2) */
  set group_stats(where=(group=2)
    rename=(mean=group2_mean std=group2_std n=group2_n));

/* Calculate Pooled Standard Deviation */
  s_pooled = sqrt(
    ((group1_n - 1) * group1_std**2 + (group2_n - 1) * group2_std**2)
```

```
    / (group1_n + group2_n - 2)
  );

  /* Calculate Cohen's d */
  cohens_d = (group1_mean - group2_mean) / s_pooled;

/* Only Retain Key Results */
  keep cohens_d s_pooled group1_mean group2_mean;
run;

/* Print d value */
proc print data=cohens_d;
  title "Cohen's d Effect Size Calculation Result (Without Covariate
Adjustment)";
  format cohens_d 8.2;
run;
```

3.8.3.3 At 6w follow-up point

```
/* Data Input */
data epicus_data;
input group hospital epicu; /* group 1=telesurgery group,  group
2=local surgery group; hospital 1=Beijing, hospital 2= Harbin,
hospital 3=Hangzhou, hospital 4=Hefei, hospital 5=Urumqi */
datalines;
1   4   13
1   4   15
2   4   12
1   4   12
2   4
2   4   13
1   2   8
2   2   10
1   2   10
1   1   16
1   1   15
2   1   17
2   1   12
2   1   9
1   1   17
1   1   12
1   1   6
1   1   6
2   1   14
```

```
2   3   4
1   3   11
2   3   18
2   3   12
1   3   4
2   3   10
2   1   12
2   5   4
2   5   4
1   5   4
1   5   4
1   5   8
1   5   7
2   1   8
;
run;

/* Main Analysis: Linear Regression Model (with Hospital and Surgery
Type as Fixed Effects) */
proc glm data= epicus_data;
class group hospital;
model epicu = group hospital / solution;
lsmeans group / pdiff=control('2') cl;  /* Using the Local Surgery
Group (2) as the Reference */
ods output LSMeanDiffCL=effect_est;     /* Output Effect Estimates */
title "Main Analysis: ANCOVA with Fixed Effects";
run;

/* Sensitivity Analysis 1: Mixed Effects Model (with Hospital as a
Random Effect) */
proc mixed data= epicus_data;
class group hospital;
model epicu = group/ solution ddfm=satterthwaite;
random hospital;
lsmeans group / pdiff cl;
ods output Estimates=mixed_effects;
title "Sensitivity Analysis: Mixed Effects Model";
run;


/* Sensitivity Analysis 2: Only Adjusting for Hospital*/
proc glm data= epicus_data;
class group hospital;
model epicu = group hospital / solution;
```

```
lsmeans group / pdiff cl;
title "Sensitivity Analysis: Adjusting Only hospital";
run;

/* Calculate Effect Size (Cohen's d) */
/* Main Analysis: Linear Regression Model Adjusted for Hospital and
Surgery Type */
proc glm data=epicus_data;
  class group hospital;
  model Epicu = group hospital/ solution;
  lsmeans group / pdiff cl;
  ods output ParameterEstimates=params FitStatistics=fit;
  title 'Main Analysis: Model Adjusted for Hospital and Surgery Type';
run;

/* Calculate Descriptive Statistics for Each Group (Mean, Standard
Deviation, Sample Size) */
proc means data=epicus_data noprint;
  class group;
  var Epicu;
  output out=group_stats mean=mean std=std n=n;
run;

/* Only Retain Group-wise Statistical Results (Exclude Overall
Statistics Row) */
data group_stats;
  set group_stats;
  where _TYPE_ = 1;  /* _TYPE_=1 Indicates Statistical Results
Grouped by group */
run;

/* Combine the Statistics of the Two Groups and Calculate Cohen's d
*/
data cohens_d;
/* Initialize Variables */
  retain group1_mean group1_std group1_n group2_mean group2_std
group2_n;

/* Read Statistics for the First Group (group=1) */
  set group_stats(where=(group=1)
    rename=(mean=group1_mean std=group1_std n=group1_n));

/* Read Statistics for the Second Group (group=2) */
  set group_stats(where=(group=2)
```

```
    rename=(mean=group2_mean std=group2_std n=group2_n));

/* Calculate Pooled Standard Deviation */
  s_pooled = sqrt(
    ((group1_n - 1) * group1_std**2 + (group2_n - 1) * group2_std**2)
    / (group1_n + group2_n - 2)
  );

  /* Calculate Cohen's d */
  cohens_d = (group1_mean - group2_mean) / s_pooled;

/* Only Retain Key Results */
  keep cohens_d s_pooled group1_mean group2_mean;
run;

/* Print d value */
proc print data=cohens_d;
  title "Cohen's d Effect Size Calculation Result (Without Covariate
Adjustment)";
  format cohens_d 8.2;
run;
```

3.9 NASA-TXL Score

3.9.1 Surgeon

```
/* Data Input */
/* Data details:
   group: 1=telesurgery group, 2=local surgery group
   hospital: 1=Beijing, 2=Harbin, 3=Hangzhou, 4=Hefei, 5=Urumqi
   surgery_type: 1=Radical Prostatectomy, 2=Partial Nephrectomy
   surgeon codes: 1=Chaozhao Liang, 2=Baojun Wang, 3=Xu Zhang,
4=Sheng Tai,
                  5=Hongzhao Li, 6=Wanghai Xu, 7=Xin Ma, 8=Qingbo
Huang,
                  9=Weijun Fu, 10=Shuo Wang, 11=Mulati Rexiati
*/
data sensitive_data;
input group hospital surgery_type surgeon sensitive;
datalines;
2   3   1   10  55
1   3   1   5   28
2   3   1   10  48
```

2	3	1	10	47
1	3	1	5	24
2	3	1	10	43
1	3	2	7	16
2	3	2	10	47
2	3	2	10	46
1	3	2	7	30
2	3	2	10	49
1	3	2	7	31
2	3	2	10	52
1	3	2	7	
1	4	1	2	68
2	4	1	4	40
2	4	1	4	37
1	4	1	3	30
1	4	1	5	26
2	4	1	1	33
2	4	2	1	42
2	4	2	1	42
1	4	2	7	30
1	5	1	5	24
1	5	1	5	24
1	5	1	5	24
1	5	1	2	28
2	5	1	2	54
2	5	1	11	57
1	5	2	7	29
2	5	2	8	23
2	5	2	8	24
1	5	2	8	24
2	1	1	9	96
1	1	1	6	55
2	1	1	9	76
1	1	1	1	
2	1	1	3	
2	1	1	9	81
1	1	1	10	
1	1	1	10	49
1	1	1	10	45
2	1	1	9	
1	1	1	9	95
2	1	1	9	
2	1	2	7	31
2	1	2	7	32

```
1   1   2   6   95
1   1   2   1
1   1   2   1
1   1   2   10  44
2   1   2   7   76
2   1   2   7
1   1   2   8   25
2   1   2   7   80
1   1   2   8   24
1   2   1   5
2   2   1   6   56
1   2   1   7   28
2   2   2   6   83
2   2   2   6   64
1   2   2   7   39
1   2   2   8
;
run;


/* 2. Main Analysis: Mixed Model (Surgeon as Random Effect) */
proc mixed data=sensitive_data method=reml noclprint;
  class group surgeon;
  model sensitive = group / solution ddfm=satterthwaite;
  random surgeon;
  lsmeans group / cl pdiff;
  ods output LSMeans=lsmeans_mixed CovParms=covparms;
  title "Main Analysis: Mixed Effects Model with Surgeon as Random";
run;

/* Extract Residual Standard Deviation */
data _null_;
  set covparms;
  if CovParm="Residual" then call symputx("resid_sd_mixed",
sqrt(Estimate));
run;

/* Calculate Cohen's d */
data cohens_d_mixed;
  length label $50;
  set lsmeans_mixed(where=(group in (1,2)) rename=(Estimate=adj_mean
StdErr=adj_se));
  retain mean1 mean2;
  if group=1 then mean1=adj_mean;
```

```
  else if group=2 then mean2=adj_mean;
  if _n_=2 then do;
    s_pooled = &resid_sd_mixed;
    cohens_d = (mean1 - mean2) / s_pooled;
    label = "Main Analysis (Mixed Model)";
    output;
  end;
  keep label cohens_d mean1 mean2 s_pooled;
run;

proc print data=cohens_d_mixed noobs label;
  title "Cohen's d from Main Analysis (Mixed Effects Model)";
run;


/* 3. Sensitivity Analysis 1: Adjusting Hospital (Mixed Model) */
proc mixed data=sensitive_data method=reml noclprint;
  class group surgeon hospital;
  model sensitive = group hospital / solution ddfm=satterthwaite;
  random surgeon;
  lsmeans group / cl pdiff;
  ods output LSMeans=lsmeans_hospital CovParms=covparms_hosp;
  title "Sensitivity Analysis 1: Mixed Model Adjusting Hospital";
run;

/* Extract Residual Standard Deviation */
data _null_;
  set covparms_hosp;
  if CovParm="Residual" then call symputx("resid_sd_hosp",
sqrt(Estimate));
run;

/* Calculate Cohen's d */
data cohens_d_hosp;
  length label $50;
  set lsmeans_hospital(where=(group in (1,2))
rename=(Estimate=adj_mean StdErr=adj_se));
  retain mean1 mean2;
  if group=1 then mean1=adj_mean;
  else if group=2 then mean2=adj_mean;
  if _n_=2 then do;
    s_pooled = &resid_sd_hosp;
    cohens_d = (mean1 - mean2) / s_pooled;
    label = "Sensitivity Analysis Adjusting Hospital";
```

```
    output;
  end;
  keep label cohens_d mean1 mean2 s_pooled;
run;

proc print data=cohens_d_hosp noobs label;
  title "Cohen's d from Sensitivity Analysis Adjusting Hospital";
run;


/* 4. Sensitivity Analysis 2: Adjusting Surgery Type (Mixed Model) */
proc mixed data=sensitive_data method=reml noclprint;
  class group surgeon surgery_type;
  model sensitive = group surgery_type / solution ddfm=satterthwaite;
  random surgeon;
  lsmeans group / cl pdiff;
  ods output LSMeans=lsmeans_surgtype CovParms=covparms_surgtype;
  title "Sensitivity Analysis 2: Mixed Model Adjusting Surgery Type";
run;

/* Extract Residual Standard Deviation */
data _null_;
  set covparms_surgtype;
  if CovParm="Residual" then call symputx("resid_sd_surgtype",
sqrt(Estimate));
run;

/* Calculate Cohen's d */
data cohens_d_surgtype;
  length label $50;
  set lsmeans_surgtype(where=(group in (1,2))
rename=(Estimate=adj_mean StdErr=adj_se));
  retain mean1 mean2;
  if group=1 then mean1=adj_mean;
  else if group=2 then mean2=adj_mean;
  if _n_=2 then do;
    s_pooled = &resid_sd_surgtype;
    cohens_d = (mean1 - mean2) / s_pooled;
    label = "Sensitivity Analysis Adjusting Surgery Type";
    output;
  end;
  keep label cohens_d mean1 mean2 s_pooled;
run;
```

```
proc print data=cohens_d_surgtype noobs label;
  title "Cohen's d from Sensitivity Analysis Adjusting Surgery Type";
run;


/* 5. Sensitivity Analysis 3: Adjusting Surgeon (fixed effect) */
proc mixed data=sensitive_data method=reml noclprint;
  class group surgeon;
  model sensitive = group surgeon / solution ddfm=satterthwaite;
  lsmeans group / cl pdiff;
  ods output LSMeans=lsmeans_surg_fixed CovParms=covparms_surg_fixed;
  title "Sensitivity Analysis 3: Mixed Model Adjusting Surgeon (as
fixed effect)";
run;

/* Extract Residual Standard Deviation */
data _null_;
  set covparms_surg_fixed;
  if CovParm="Residual" then call symputx("resid_sd_surg_fixed",
sqrt(Estimate));
run;

/* Calculate Cohen's d */
data cohens_d_surg_fixed;
  length label $50;
  set lsmeans_surg_fixed(where=(group in (1,2))
rename=(Estimate=adj_mean StdErr=adj_se));
  retain mean1 mean2;
  if group=1 then mean1=adj_mean;
  else if group=2 then mean2=adj_mean;
  if _n_=2 then do;
    s_pooled = &resid_sd_surg_fixed;
    cohens_d = (mean1 - mean2) / s_pooled;
    label = "Sensitivity Analysis Adjusting Surgeon (fixed effect)";
    output;
  end;
  keep label cohens_d mean1 mean2 s_pooled;
run;

proc print data=cohens_d_surg_fixed noobs label;
  title "Cohen's d from Sensitivity Analysis Adjusting Surgeon
(fixed)";
run;
```

3.9.2 First Assistant

3.9.3 Instrument nurse

```
/* Data Input */
data sensitive_data;
input group hospital surgery_type surgeon sensitive; /* group
1=telesurgery group,  group 2=local surgery group; hospital 1=Beijing,
hospital 2= Harbin, hospital 3=Hangzhou, hospital 4=Hefei, hospital
5=Urumqi; surgery_type 1=Radical Prostatectomy, surgery_type
2=Partial Nephrectomy; Chaozhao Liang=1, Baojun Wang=2, Xu Zhang=3,
Sheng Tai=4, Hongzhao Li=5, Wanghai Xu=6, Xin Ma=7, Qingbo Huang=8,
Weijun Fu=9, Shuo Wang=10, Mulati Rexiati=11*/
datalines;
2   3   1   10  67
1   3   1   5   59
2   3   1   10  61
2   3   1   10  55
1   3   1   5   59
2   3   1   10  64
1   3   2   7   60
2   3   2   10  62
2   3   2   10  57
1   3   2   7
2   3   2   10  60
1   3   2   7
2   3   2   10  56
1   3   2   7
1   4   1   2   24
2   4   1   4   36
2   4   1   4   28
1   4   1   3   34
1   4   1   5   34
2   4   1   1   33
2   4   2   1   40
2   4   2   1   32
1   4   2   7   44
1   5   1   5   66
1   5   1   5   51
1   5   1   5   99
1   5   1   2   92
2   5   1   2   53
2   5   1   11  56
1   5   2   7   60
```

```
2   5   2   8   60
2   5   2   8   54
1   5   2   8   37
2   1   1   9   90
1   1   1   6   30
2   1   1   9   34
1   1   1   1   31
2   1   1   3
2   1   1   9   68
1   1   1   10  66
1   1   1   10  49
1   1   1   10  21
2   1   1   9   42
1   1   1   9   61
2   1   1   9
2   1   2   7   49
2   1   2   7   50
1   1   2   6   39
1   1   2   1
1   1   2   1   48
1   1   2   10  47
2   1   2   7   47
2   1   2   7
1   1   2   8   40
2   1   2   7   32
1   1   2   8   65
1   2   1   5   56
2   2   1   6   49
1   2   1   7   51
2   2   2   6   80
2   2   2   6   50
1   2   2   7   45
1   2   2   8   47
run;

/* Main Analysis: Linear Regression Model (with Hospital, Surgery
Type and Surgeon as Fixed Effects) */
proc glm data= sensitive_data;
class group hospital surgery_type surgeon;
model sensitive = group hospital surgery_type surgeon/ solution;
lsmeans group / pdiff=control('2') cl;  /* Using the Local Surgery
Group (2) as the Reference */
ods output LSMeanDiffCL=effect_est;     /* Output Effect Estimates */
title "Main Analysis: ANCOVA with Fixed Effects";
```

```
run;

/* Sensitivity Analysis 1: Mixed Effects Model (with Surgeon as a
Random Effect) */
proc mixed data= sensitive_data;
class group surgeon;
model sensitive = group/ solution ddfm=satterthwaite;
random surgeon;
lsmeans group / pdiff cl;
ods output Estimates=mixed_effects;
title "Sensitivity Analysis: Mixed Effects Model";
run;

/* Sensitivity Analysis 2: Only Adjusting for Hospital*/
proc glm data= sensitive_data;
class group hospital;
model sensitive = group hospital / solution;
lsmeans group / pdiff cl;
title "Sensitivity Analysis: Adjusting Only hospital";
run;

/* Sensitivity Analysis 3: Only Adjusting for Surge Type*/
proc glm data= sensitive_data;
class group surgery_type;
model sensitive = group surgery_type / solution;
lsmeans group / pdiff cl;
title "Sensitivity Analysis: Adjusting Only Surge Type";
run;

/* Calculate Effect Size (Cohen's d) */
/* Main Analysis: Linear Regression Model Adjusted for Surgeon */
proc glm data=sensitive_data;
  class group surgeon;
  model sensitive = group surgeon/ solution;
  lsmeans group / pdiff cl;
  ods output ParameterEstimates=params FitStatistics=fit;
  title 'Main Analysis: Model Adjusted for Surgeon';
run;

/* Calculate Descriptive Statistics for Each Group (Mean, Standard
Deviation, Sample Size) */
proc means data=sensitive_data noprint;
  class group;
  var sensitive;
```

```
  output out=group_stats mean=mean std=std n=n;
run;

/* Only Retain Group-wise Statistical Results (Exclude Overall
Statistics Row) */
data group_stats;
  set group_stats;
  where _TYPE_ = 1;  /* _TYPE_=1 Indicates Statistical Results
Grouped by group */
run;

/* Combine the Statistics of the Two Groups and Calculate Cohen's d
*/
data cohens_d;
/* Initialize Variables */
  retain group1_mean group1_std group1_n group2_mean group2_std
group2_n;

/* Read Statistics for the First Group (group=1) */
  set group_stats(where=(group=1)
    rename=(mean=group1_mean std=group1_std n=group1_n));

/* Read Statistics for the Second Group (group=2) */
  set group_stats(where=(group=2)
    rename=(mean=group2_mean std=group2_std n=group2_n));

/* Calculate Pooled Standard Deviation */
  s_pooled = sqrt(
    ((group1_n - 1) * group1_std**2 + (group2_n - 1) * group2_std**2)
    / (group1_n + group2_n - 2)
  );

  /* Calculate Cohen's d */
  cohens_d = (group1_mean - group2_mean) / s_pooled;

/* Only Retain Key Results */
  keep cohens_d s_pooled group1_mean group2_mean;
run;

/* Print d value */
proc print data=cohens_d;
  title "Cohen's d Effect Size Calculation Result (Without Covariate
Adjustment)";
  format cohens_d 8.2;
```

```
run;
```

4. Mixed-effects Logistic regression with Firth's correction (Sensitive analysis for Positive surgical margin)

```
/* Import data; Surgeon,hospital or surgery type as fixed effects */
data sensitive_data;
input group hospital surgery_type surgeon sensitive;
datalines;
2   3   1   10  1
1   3   1   5   0
2   3   1   10  1
2   3   1   10  0
1   3   1   5   0
2   3   1   10  0
1   3   2   7   0
2   3   2   10  0
2   3   2   10  0
1   3   2   7   0
2   3   2   10  0
1   3   2   7   0
2   3   2   10  0
1   3   2   7   0
1   4   1   2   0
2   4   1   4   0
2   4   1   4   0
1   4   1   3   0
1   4   1   5   1
2   4   1   1   1
2   4   2   1   0
2   4   2   1   0
1   4   2   7   0
1   5   1   5   0
1   5   1   5   0
1   5   1   5   0
1   5   1   2   0
2   5   1   2   0
2   5   1   11  0
1   5   2   7   0
2   5   2   8   0
2   5   2   8   0
1   5   2   8   0
2   1   1   9   0
1   1   1   6   0
```

```
2   1   1   9   1
1   1   1   1   0
2   1   1   3   0
2   1   1   9   0
1   1   1   10  0
1   1   1   10  0
1   1   1   10  0
2   1   1   9   0
1   1   1   9   0
2   1   1   9   1
2   1   2   7   0
2   1   2   7   0
1   1   2   6   0
1   1   2   1   0
1   1   2   1   0
1   1   2   10  0
2   1   2   7   0
2   1   2   7   0
1   1   2   8   0
2   1   2   7   0
1   1   2   8   0
1   2   1   5   0
2   2   1   6   0
1   2   1   7   0
2   2   2   6   0
2   2   2   6   0
1   2   2   7   0
1   2   2   8   0
;
run;

/*==================================================*/
/* Main analysis: joint adjustment for hospital, surgery_type,
surgeon; Firth correction */
/*==================================================*/
ods output ParameterEstimates=param_est_main;
title "Main Analysis: Firth-corrected Logistic Regression - Joint
Adjustment for hospital, surgery_type, surgeon";
proc logistic data=sensitive_data;
   class group(ref='2') hospital(ref='1') surgery_type(ref='1')
surgeon(ref='1') / param=ref;
   model sensitive(event='1') = group hospital surgery_type surgeon /
firth clodds=pl maxiter=100;
run;
```

```
title;
ods output close;

/* Calculate OR and 95% CI */
data param_est_main2;
   set param_est_main;
   OR = exp(Estimate);
   LowerCL = exp(Estimate - 1.96*StdErr);
   UpperCL = exp(Estimate + 1.96*StdErr);
run;

/* Print main analysis OR and 95% CI */
title "Main Analysis: OR and 95% Confidence Interval Adjusted for
hospital, surgery_type, surgeon";
proc print data=param_est_main2 noobs label;
   var Variable Estimate StdErr OR LowerCL UpperCL ProbChiSq;
   label
      Variable = "Variable"
      Estimate = "Estimate"
      StdErr = "Standard Error"
      OR = "Odds Ratio"
      LowerCL = "OR Lower Limit (95% CI)"
      UpperCL = "OR Upper Limit (95% CI)"
      ProbChiSq = "P-value"
   ;
run;

/*==================================================*/
/* Sensitivity analysis 1: adjustment for hospital only; Firth
correction */
/*==================================================*/
ods output ParameterEstimates=param_est_sens1;
title "Sensitivity Analysis 1: Firth-corrected Logistic Regression -
Adjustment for hospital only";
proc logistic data=sensitive_data;
   class group(ref='2') hospital(ref='1') / param=ref;
   model sensitive(event='1') = group hospital / firth clodds=pl
maxiter=100;
run;
title;
ods output close;

/* Calculate OR and 95% CI */
data param_est_sens1_2;
```

```
   set param_est_sens1;
   OR = exp(Estimate);
   LowerCL = exp(Estimate - 1.96*StdErr);
   UpperCL = exp(Estimate + 1.96*StdErr);
run;

/* Print sensitivity analysis 1 results */
title "Sensitivity Analysis 1: OR and 95% Confidence Interval
Adjusted for hospital only";
proc print data=param_est_sens1_2 noobs label;
   var Variable Estimate StdErr OR LowerCL UpperCL ProbChiSq;
   label
      Variable = "Variable"
      Estimate = "Estimate"
      StdErr = "Standard Error"
      OR = "Odds Ratio"
      LowerCL = "OR Lower Limit (95% CI)"
      UpperCL = "OR Upper Limit (95% CI)"
      ProbChiSq = "P-value"
   ;
run;

/*================================================*/
/* Sensitivity analysis 2: adjustment for surgery_type only; Firth
correction */
/*================================================*/
ods output ParameterEstimates=param_est_sens2;
title "Sensitivity Analysis 2: Firth-corrected Logistic Regression -
Adjustment for surgery_type only";
proc logistic data=sensitive_data;
   class group(ref='2') surgery_type(ref='1') / param=ref;
   model sensitive(event='1') = group surgery_type / firth clodds=pl
maxiter=100;
run;
title;
ods output close;

/* Calculate OR and 95% CI */
data param_est_sens2_2;
   set param_est_sens2;
   OR = exp(Estimate);
   LowerCL = exp(Estimate - 1.96*StdErr);
   UpperCL = exp(Estimate + 1.96*StdErr);
run;
```

```
/* Print sensitivity analysis 2 results */
title "Sensitivity Analysis 2: OR and 95% Confidence Interval
Adjusted for surgery_type only";
proc print data=param_est_sens2_2 noobs label;
   var Variable Estimate StdErr OR LowerCL UpperCL ProbChiSq;
   label
      Variable = "Variable"
      Estimate = "Estimate"
      StdErr = "Standard Error"
      OR = "Odds Ratio"
      LowerCL = "OR Lower Limit (95% CI)"
      UpperCL = "OR Upper Limit (95% CI)"
      ProbChiSq = "P-value"
   ;
run;

/*===============================================*/
/* Sensitivity analysis 3: adjustment for surgeon only; Firth
correction */
/*===============================================*/
ods output ParameterEstimates=param_est_sens3;
title "Sensitivity Analysis 3: Firth-corrected Logistic Regression -
Adjustment for surgeon only";
proc logistic data=sensitive_data;
   class group(ref='2') surgeon(ref='1') / param=ref;
   model sensitive(event='1') = group surgeon / firth clodds=pl
maxiter=100;
run;
title;
ods output close;

/* Calculate OR and 95% CI */
data param_est_sens3_2;
   set param_est_sens3;
   OR = exp(Estimate);
   LowerCL = exp(Estimate - 1.96*StdErr);
   UpperCL = exp(Estimate + 1.96*StdErr);
run;

/* Print sensitivity analysis 3 results */
title "Sensitivity Analysis 3: OR and 95% Confidence Interval
Adjusted for surgeon only";
proc print data=param_est_sens3_2 noobs label;
```

```
   var Variable Estimate StdErr OR LowerCL UpperCL ProbChiSq;
   label
      Variable = "Variable"
      Estimate = "Estimate"
      StdErr = "Standard Error"
      OR = "Odds Ratio"
      LowerCL = "OR Lower Limit (95% CI)"
      UpperCL = "OR Upper Limit (95% CI)"
      ProbChiSq = "P-value"
   ;
run;
```

5. Exact Fisher's test for Clavin-Dindo Complications

5.1 Over Level III complications in 6 weeks

```
/* Create dataset mydata */
data mydata;
    input y x $;
    /*y is for trail group, 1=telesurgery, 2=localsurgery group; x is
for event, 1=occurrence, 2=non- occurrence */
    datalines;
1   A
1   B
1   B
1   B
1   B
1   B
1   B
1   B
1   B
1   B
1   B
1   B
1   B
1   B
1   B
1   B
1   B
1   B
1   B
1   B
1   B
1   B
1   B
```

```
1   B
1   B
1   B
1   B
1   B
1   B
1   B
1   B
1   B
2   B
2   B
2   B
2   B
2   B
2   B
2   B
2   B
2   B
2   B
2   B
2   B
2   B
2   B
2   B
2   B
2   B
2   B
2   B
2   B
2   B
2   B
2   B
2   B
2   B
2   B
2   B
2   B
2   B
2   B
2   B
;
run;
proc freq data=mydata;
    tables x*y / chisq exact out=cross_tab; /* Output the crosstab to
```

```
the dataset cross_tab */
    run;

/* View the cross_tab dataset to obtain frequencies */
proc print data=cross_tab;
run;

/* Assuming the following frequencies are read from the cross_tab or
PROC FREQ output, /
/ but for this example, we will use hypothetical values directly */
%let a = ; /* Number of observations where A and y=0 */
%let b = ; /* Number of observations where A and y=1 */
%let c = ; / Number of observations where B and y=0 */
%let d = ; / Number of observations where B and y=1 */


/* Calculate the Odds Ratio */
data odds_ratio;
    odds_ratio = (&a * &d) / (&b * &c);
    log_odds_ratio = log(odds_ratio);
    put "Odds Ratio: " odds_ratio; /* Print the Odds Ratio in the log
*/
        output;
run;

/* Use PROC PRINT to view the results */
proc print data=odds_ratio;
    title "Calculated Odds Ratio and Log Odds Ratio";
run;
```

5.2 At 4-week follow up timepoint（all level complications）

```
/* Create dataset mydata */
data mydata;
        input y x $;  /*y is for trail group, 1=telesurgery,
2=localsurgery group; x is for event, 1=occurrence, 2=non- occurrence
*/
    datalines;
1   A
1   A
1   A
1   A
1   B
1   B
```

1	B
1	B
1	B
1	B
1	B
1	B
1	B
1	B
1	B
1	B
1	B
1	B
1	B
1	B
1	B
1	B
1	B
1	B
1	B
1	B
1	B
1	B
1	B
1	B
1	B
2	A
2	B
2	B
2	B
2	B
2	B
2	B
2	B
2	B
2	B
2	B
2	B
2	B
2	B
2	B
2	B
2	B
2	B
2	B

```
2   B
2   B
2   B
2   B
2   B
2   B
2   B
2   B
2   B
;
run;
proc freq data=mydata;
    tables x*y / chisq exact out=cross_tab; /* Output the crosstab to
the dataset cross_tab */
    run;

/* View the cross_tab dataset to obtain frequencies */
proc print data=cross_tab;
run;

/* Assuming the following frequencies are read from the cross_tab or
PROC FREQ output, /
/ but for this example, we will use hypothetical values directly */
%let a = ; /* Number of observations where A and y=0 */
%let b = ; /* Number of observations where A and y=1 */
%let c = ; / Number of observations where B and y=0 */
%let d = ; / Number of observations where B and y=1 */


/* Calculate the Odds Ratio */
data odds_ratio;
    odds_ratio = (&a * &d) / (&b * &c);
    log_odds_ratio = log(odds_ratio);
    put "Odds Ratio: " odds_ratio; /* Print the Odds Ratio in the log
*/
        output;
run;

/* Use PROC PRINT to view the results */
proc print data=odds_ratio;
    title "Calculated Odds Ratio and Log Odds Ratio";
run;
```

5.3 At 6-week follow up timepoint

```
/* Create dataset mydata */
data mydata;
        input y x $;  /*y is for trail group, 1=telesurgery, 2=localsurgery group; x is for event, 1=occurrence, 2=non- occurrence */
    datalines;
1   A
1   A
1   A
1   A
1   B
1   B
1   B
1   B
1   B
1   B
1   B
1   B
1   B
1   B
1   B
1   B
1   B
1   B
1   B
1   B
1   B
1   B
1   B
1   B
1   B
1   B
1   B
1   B
1   B
1   B
1   B
2   A
2   B
2   B
2   B
2   B
2   B
```

```
2   B
2   B
2   B
2   B
2   B
2   B
2   B
2   B
2   B
2   B
2   B
2   B
2   B
2   B
2   B
2   B
2   B
2   B
2   B
2   B
2   B
2   B
;
run;
proc freq data=mydata;
    tables x*y / chisq exact out=cross_tab; /* Output the crosstab to
the dataset cross_tab */
    run;

/* View the cross_tab dataset to obtain frequencies */
proc print data=cross_tab;
run;

/* Assuming the following frequencies are read from the cross_tab or
PROC FREQ output, /
/ but for this example, we will use hypothetical values directly */
%let a = ; /* Number of observations where A and y=0 */
%let b = ; /* Number of observations where A and y=1 */
%let c = ; / Number of observations where B and y=0 */
%let d = ; / Number of observations where B and y=1 */


/* Calculate the Odds Ratio */
data odds_ratio;
```

```
    odds_ratio = (&a * &d) / (&b * &c);
    log_odds_ratio = log(odds_ratio);
    put "Odds Ratio: " odds_ratio; /* Print the Odds Ratio in the log
*/
        output;
run;

/* Use PROC PRINT to view the results */
proc print data=odds_ratio;
    title "Calculated Odds Ratio and Log Odds Ratio";
run;
```

6. Bayesian mixed-effects logistic regression (Main analysis for the Positive margin;The R Programming Language)

```
# Surgeon as Random effect
library(brms)
library(dplyr)
library(tibble)
library(posterior)  # Dependency for brms, processes posterior draws

# 1. Construct data (manually created from your data)
data_text <- "
group hospital surgery_type surgeon sensitive
2 3 1 10 1
1 3 1 5 0
2 3 1 10 1
2 3 1 10 0
1 3 1 5 0
2 3 1 10 0
1 3 2 7 0
2 3 2 10 0
2 3 2 10 0
1 3 2 7 0
2 3 2 10 0
1 3 2 7 0
2 3 2 10 0
1 3 2 7 0
1 4 1 2 0
2 4 1 4 0
```

2 4 1 4 0
1 4 1 3 0
1 4 1 5 1
2 4 1 1 1
2 4 2 1 0
2 4 2 1 0
1 4 2 7 0
1 5 1 5 0
1 5 1 5 0
1 5 1 5 0
1 5 1 2 0
2 5 1 2 0
2 5 1 11 0
1 5 2 7 0
2 5 2 8 0
2 5 2 8 0
1 5 2 8 0
2 1 1 9 0
1 1 1 6 0
2 1 1 9 1
1 1 1 1 0
2 1 1 3 0
2 1 1 9 0
1 1 1 10 0
1 1 1 10 0
1 1 1 10 0
2 1 1 9 0
1 1 1 9 0
2 1 1 9 1
2 1 2 7 0
2 1 2 7 0
1 1 2 6 0
1 1 2 1 0
1 1 2 1 0
1 1 2 10 0
2 1 2 7 0
2 1 2 7 0
1 1 2 8 0
2 1 2 7 0
1 1 2 8 0
1 2 1 5 0
2 2 1 6 0
1 2 1 7 0
2 2 2 6 0

```
2 2 2 6 0
1 2 2 7 0
1 2 2 8 0
"

# Read data
sensitive_data <- read.table(text = data_text, header = TRUE)

# Conversion factor
sensitive_data <- sensitive_data %>%
  mutate(
    group = factor(group),
    hospital = factor(hospital),
    surgery_type = factor(surgery_type),
    surgeon = factor(surgeon),
    sensitive = as.integer(sensitive)
  )

# Set penalizing priors
priors <- c(
  prior(normal(0, 5), class = "b"),             # Prior for fixed
effect coefficients
  prior(cauchy(0, 2), class = "sd")              # Prior for random
effect sd
)

# Fit the model
fit_main <- brm(
  formula = sensitive ~ group + hospital + surgery_type + (1 |
surgeon),
  data = sensitive_data,
  family = bernoulli("logit"),
  prior = priors,
  chains = 4,
  iter = 4000,
  seed = 1234,
  control = list(adapt_delta = 0.95),
  refresh = 0
)

# Posterior extraction and results
post_samples <- as_draws_df(fit_main)

param_name <- "b_group2"
```

```r
post_param <- post_samples[[param_name]]

est_mean <- mean(post_param)
est_ci <- quantile(post_param, c(0.025, 0.975))

OR <- exp(est_mean)
OR_low <- exp(est_ci[1])
OR_high <- exp(est_ci[2])

p_one_sided <- mean(post_param > 0)
p_two_sided <- 2 * min(p_one_sided, 1 - p_one_sided)
p_two_sided <- ifelse(p_two_sided > 1, 1, p_two_sided)

result <- data.frame(
  Parameter = "group2 vs group1",
  Estimate_logOR = est_mean,
  OR = OR,
  OR_low = OR_low,
  OR_high = OR_high,
  p_value = p_two_sided
)

print(result)
```

9. Univariate linear regression (The R Programming Language)

```r
# --------------------- Environment Preparation ---------------------
options(repos = c(CRAN =
"https://mirrors.tuna.tsinghua.edu.cn/CRAN/"))
install.packages(c("readr", "dplyr", "ggplot2", "broom"))
library(readr)
library(dplyr)
library(ggplot2)

# --------------------- Data Reading ---------------------
file_path <- file.choose()
df <- read_csv(
  file_path,
  col_types = cols(
    Distance = col_double(),
    Latency = col_double()
  ),
```

```
  na = c("", "NA", "NULL")
) %>%
  rename(Distance = 1, Latency = 2)  # Forcefully specify the first
two columns

# --------------------- Data Cleaning ---------------------
clean_df <- df %>%
  mutate(
   Distance = Distance / 1000  # Assume original data is in meters,
convert to kilometers
  ) %>%
  filter(
    Distance >= 0,
    Latency >= 0,
    !is.na(Distance),
    !is.na(Latency)
  ) %>%
  distinct() %>%
  arrange(Distance)

# --------------------- Regression Analysis ---------------------
model <- lm(Latency ~ Distance, data = clean_df)
regression_summary <- summary(model)

# --------------------- Visualization Optimization ------------------
---
ggplot(clean_df, aes(x = Distance, y = Latency)) +
 geom_point(color = "#1f77b4", alpha = 0.6) +
  geom_smooth(method = "lm", color = "#ff7f0e") +
  annotate(
    "text",
    x = max(clean_df$Distance) * 0.8,
    y = min(clean_df$Latency),
    label = paste(
      sprintf("y = %.2f + %.2fx", coef(model)[1], coef(model)[2]),
      sprintf("R² = %.3f", regression_summary$r.squared),
      sprintf("p = %.1e", coef(summary(model))[2, 4]),
      sep = "\n"
    ),
    color = "#2ca02c",
    size = 5,
    hjust = 0
  ) +
  labs(
```

```
    x = "Physical Distance (km)",
    y = "Network Latency (ms)",
    title = "Relationship Between Network Latency and Distance (After
Unit Correction)"
  ) +
  theme_bw()

# --------------------- Results Saving ---------------------
ggsave("network_latency_corrected.png", width = 8, height = 6, dpi =
300)
```

10. Box-plot (The R Programming Language)

```
# Load the ggbeeswarm library, which is necessary to use the
geom_quasirandom function
library(ggbeeswarm)

# Load the ggplot2 library for data visualization
library(ggplot2)

# Load the dplyr library for data manipulation
library(dplyr)

# Ensure the ggbeeswarm library is loaded
library(ggbeeswarm)

# Load the showtext library to use Chinese fonts in ggplot2
library(showtext)

# ====== 1. Initialize Chinese Font ======
# Add the SimHei font to the system
font_add("SimHei", "simhei.ttf")
# Automatically use the showtext package for text rendering
showtext_auto()

# ====== 2. Data Reading and Cleaning ======
# Read the CSV file from the specified path and perform data
transformations
df <- read.csv("C:/Users/123/Desktop/data.csv") %>%
  mutate(
    # Convert the group variable to a factor with custom labels
    group = factor(group, labels = paste0("group", 1:4)),
    # Convert the loss variable to numeric
```

```
    loss = as.numeric(loss)
  ) %>%
  # Filter out rows where the loss variable is NA
  filter(!is.na(loss))

# ====== 3. Plot an Enhanced Boxplot ======
# Create a ggplot with customized aesthetics and layers
ggplot(df, aes(x = group, y = loss, fill = group)) +
  # Add a boxplot layer with adjusted width and no outliers displayed
  geom_boxplot(width = 0.6, outlier.shape = NA) +
  # Add a quasi-random (jittered) point layer for better
visualization of overlapping points
  geom_quasirandom(
    # Specify the Tukey method for quasi-random jittering
    method = "tukey",
    # Adjust the width of the jittered points
    width = 0.2,
    # Set the transparency level of the points
    alpha = 0.6,
    # Set the size of the points
    size = 2,
    # Set the color of the points
    color = "#333333"
  ) +
  # Add a title and y-axis label
  labs(title = "frame loss analysis", y = "number of frame loss") +
  # Apply a clean theme
  theme_bw()

# ====== 4. Save the Plot as an Image ======
# Save the ggplot as a PNG file with specified dimensions and
resolution
ggsave("data_analysis.png", width = 10, height = 7, dpi = 300)
```

11. Violin Plot combined with Box-plot (The R Programming Language)

```
# Load necessary libraries
library(ggplot2)
library(tidyr)
library(dplyr)
library(stringr)

# ========== 1. Data Reading ==========
```

```
# 1. Read the CSV file (please replace with your actual file path)
data <- read.csv("C:/Users/---/Desktop/data.csv")  # This line is
redundant if you're not using 'data' later
raw_data <- read.csv("C:/Users/---/Desktop/data.csv")  # Corrected
file path string

# ========== 2. Data Preprocessing ==========
# Convert to long format (from wide to long)
data_long <- raw_data %>%
  pivot_longer(
    cols = everything(),
    names_to = "Sample",
    values_to = "Latency"
  )

# Automatically correct sample ordering (crucial step)
data_long <- data_long %>%
  mutate(
    # Extract numeric part of sample ID (supports any length of
digits)
    sample_num = as.numeric(str_extract(Sample, "\\d+")),
    # Create factor by ordering numerically
    Sample = factor(Sample, levels =
unique(Sample[order(sample_num)]))
  ) %>%
  select(-sample_num)  # Remove temporary column

# Verify sorting order (optional)
cat("Verify sample order:\n")
print(levels(data_long$Sample))

# ========== 3. Visualization Plotting ==========
ggplot(data_long, aes(x = Sample, y = Latency, fill = Sample)) +
  # Violin layer
  geom_violin(
    trim = TRUE,
    scale = "width",
    alpha = 0.8,
    width = 0.85,
    color = "black"
  ) +
  # Boxplot layer
  geom_boxplot(
    width = 0.15,
```

```
    alpha = 0.6,
    outlier.shape = NA,
    fill = "white"
  ) +
  # Color configuration
  scale_fill_viridis_d(
    option = "D",
    begin = 0.1,
    end = 0.9,
    guide = "none"
  ) +
  # Labels and theme
  labs(
    title = "Network Latency Distribution Analysis",
    x = "Sample ID",
    y = "Latency (ms)",
    caption = "Data Source: Actual Network Measurement Data"
  ) +
  theme_minimal(base_size = 12) +
  theme(
    axis.text.x = element_text(
      angle = 45,
      hjust = 1,
      vjust = 1,
      face = "bold"
    ),
    plot.title = element_text(
      size = 16,
      hjust = 0.5,
      margin = margin(b = 15)
    )
  ) +
  # Y-axis expansion
  scale_y_continuous(expand = expansion(mult = c(0.02, 0.1)))

# Note: The 'n_distinct' function should be from 'dplyr' but is not
directly used here in ggsave.
# Instead, we'll calculate the number of distinct samples and use it
in the width calculation.
num_samples <- n_distinct(data_long$Sample)

# ========== 4. Save the Image ==========
ggsave(
  "network_latency_plot.png",
```

```
  width = 6 + num_samples * 0.4,  # Automatically adjust width based
on number of samples
  height = 6,
  dpi = 300
)
```
